# Structure-based design of stabilized recombinant influenza neuraminidase tetramers

**DOI:** 10.1038/s41467-022-29416-z

**Published:** 2022-04-05

**Authors:** Daniel Ellis, Julia Lederhofer, Oliver J. Acton, Yaroslav Tsybovsky, Sally Kephart, Christina Yap, Rebecca A. Gillespie, Adrian Creanga, Audrey Olshefsky, Tyler Stephens, Deleah Pettie, Michael Murphy, Claire Sydeman, Maggie Ahlrichs, Sidney Chan, Andrew J. Borst, Young-Jun Park, Kelly K. Lee, Barney S. Graham, David Veesler, Neil P. King, Masaru Kanekiyo

**Affiliations:** 1 Institute for Protein Design, University of Washington, Seattle, WA 98195 USA; 2 Department of Biochemistry, University of Washington, Seattle, WA 98195 USA; 3 Graduate Program in Molecular and Cellular Biology, University of Washington, Seattle, WA 98195 USA; 4 Vaccine Research Center, National Institute of Allergy and Infectious Diseases, National Institutes of Health, Bethesda, MD 20892 USA; 5 Electron Microscopy Laboratory, Cancer Research Technology Program, Frederick National Laboratory for Cancer Research sponsored by the National Cancer Institute, Frederick, MD 21702 USA; 6 Department of Medicinal Chemistry, University of Washington, Seattle, WA 98195 USA; 7 Department of Bioengineering, University of Washington, Seattle, WA 98195 USA; 8 Howard Hughes Medical Institute, University of Washington, Seattle, WA 98195 USA; 9 Present Address: Icosavax Inc., Seattle, WA 98102 USA

**Keywords:** Cryoelectron microscopy, Protein design, Influenza virus

## Abstract

Influenza virus neuraminidase (NA) is a major antiviral drug target and has recently reemerged as a key target of antibody-mediated protective immunity. Here we show that recombinant NAs across non-bat subtypes adopt various tetrameric conformations, including an “open” state that may help explain poorly understood variations in NA stability across viral strains and subtypes. We use homology-directed protein design to uncover the structural principles underlying these distinct tetrameric conformations and stabilize multiple recombinant NAs in the “closed” state, yielding two near-atomic resolution structures of NA by cryo-EM. In addition to enhancing thermal stability, conformational stabilization improves affinity to protective antibodies elicited by viral infection, including antibodies targeting a quaternary epitope and the broadly conserved catalytic site. Stabilized NAs can also be integrated into viruses without affecting fitness. Our findings provide a deeper understanding of NA structure, stability, and antigenicity, and establish design strategies for reinforcing the conformational integrity of recombinant NA proteins.

## Abstract (web summary)

Influenza virus neuraminidase (NA) is a drug target and a potential vaccine antigen. Here, the authors provide a detailed analysis of the conformational stability of NA, and show how expression and stability of recombinant NA antigens can be strengthened through structure-based design.

## Introduction

Influenza viruses are negative-sense RNA viruses in the family *Orthomyxoviridae*. The two major viral glycoproteins, hemagglutinin (HA) and neuraminidase (NA), facilitate viral entry and egress from host cells, respectively. NA is an enzyme that binds and cleaves sialosides from glycans on the host cell surface to facilitate the release of nascent viral particles from infected cells, and is also proposed to assist other stages in the virus replication cycle^1,2^. Like HA, influenza A NAs are divided into subtypes that are clustered into the phylogenetically defined groups 1 (N1, N4, N5, and N8) and 2 (N2, N3, N6, N7, and N9), whereas influenza B NAs are clustered in a separate branch^3^. NA is a homotetrameric, type II integral membrane protein with a short cytoplasmic N-terminal domain. The C-terminal catalytic domain folds into a disulfide-stabilized and glycosylated six-bladed beta propeller and is supported by a hypervariable stalk domain^4,5^. Crystal structures of NA catalytic domains, either proteolytically released from virions or produced recombinantly, have consistently shown that four identical subunits interact non-covalently to form a globular “head” with four-fold symmetry^2,6^. The head features deep pockets on the exterior face of each protomer comprising the catalytic residues. NA contains several Ca^2+^ binding sites, one of which is highly conserved and supports the periphery of the catalytic pocket on each protomer, while another site at the four-fold symmetry axis is frequently, but not universally, observed across various subtypes^7^. Ca^2+^ ions have been shown to assist catalytic activity^2^ and have been resolved to varying degrees in crystal structures, with reports and predictions of increased structural flexibility in their absence^8,9^. These observations, combined with remote allosteric effects that impact sialoside binding and resistance to catalytic inhibitors, highlight the relatively plastic conformational landscape of NA^10–14^.

While multiple studies have shown that antibodies against NA correlate with immunity against influenza viruses^15–20^, substantial gaps remain in our knowledge of NA-directed immunity, particularly in terms of the relationships between NA structure, conformational stability, and antigenicity. The antigenicity of NA has long been detailed using monoclonal antibodies (mAbs) isolated from immunized and/or infected mice, which provided an initial understanding of its distinct antigenic regions—some of which span adjacent protomers—as well as mechanisms of both enzymatic inhibition and protection^21–25^. Although an NA-based vaccine, extracted from viral membranes and inactivated by formalin, improved humoral NA-directed enzymatic inhibition compared to commercial influenza vaccines in humans in a Phase I clinical trial more than 25 years ago^26,27^, only preclinical studies of NA-based vaccines have been reported since^28–32^. More recently, studies comparing antibodies elicited by infection or immunization with split virus vaccines have clearly demonstrated that infection results in substantially more robust NA-directed humoral responses in humans^33^. Protective human mAbs have therefore most frequently been isolated from infected rather than immunized individuals, with the recent exception of mAbs isolated from a minority of subjects that generated detectable anti-NA responses after receiving H7N9 vaccines^33–36^. These studies have further defined NA epitopes targeted by protective antibodies, most notably leading to the isolation of broadly cross-reactive and protective antibodies from an H3N2-infected individual that bind the catalytic site^36^.

The disconnect between infection- and vaccine-elicited responses against NA highlights our incomplete knowledge of the structural and antigenic stability of NA outside its native context in the viral membrane. Conformational differences between membrane-bound and recombinant forms of several viral antigens have been noted previously, and the design of conformationally stabilized recombinant antigens has provided useful tools for basic and translational research^37–40^. In agreement with crystal structures, tomographic models of NA on the surface of virions have shown a compact, “closed” four-fold symmetric arrangement of the catalytic domains^41,42^. Yet in the absence of natural or exogenous tetramerization domains, soluble recombinant NA heads can dissociate into monomers^43–45^, suggesting that interactions between NA heads are relatively weak. Nevertheless, in contrast to other sialidases that share the same six-bladed propeller fold and can function as monomers^46^, it has frequently been reported that tetramer formation is a prerequisite for the function of influenza NA, and a correlation between enzymatic activity and immunogenicity of recombinant NAs has been observed^43,45,47–49^. Consequently, catalytic activity is often considered an indicator of the biologically relevant conformation^50^. However, enzymatic activity is neither a direct nor complete readout of the native antigenic structure of NA^45^. Recent attempts to better understand and improve the conformational stability of NA have shown some promise, and have suggested connections between Ca^2+^ binding, antigenicity, oligomerization, and both thermal and conformational stability^44,45,51^.

Here we characterize in detail distinct tetrameric conformations adopted by recombinant soluble NAs, including an “open” tetrameric conformation that does not appear to represent the native membrane-bound conformation, and use this information to develop a general structure-based design strategy for stabilizing the closed tetrameric state. Closed versions of recombinant N1 NAs have improved stability and affinity to infection-elicited protective antibodies relative to their wild-type counterparts. Conformationally stabilized NAs may be valuable reagents for facilitating the design and discovery of NA-directed vaccines and therapeutics.

## Results

### Tetrameric conformations of recombinant NA proteins vary across influenza strains and subtypes

**Fig. 1:**
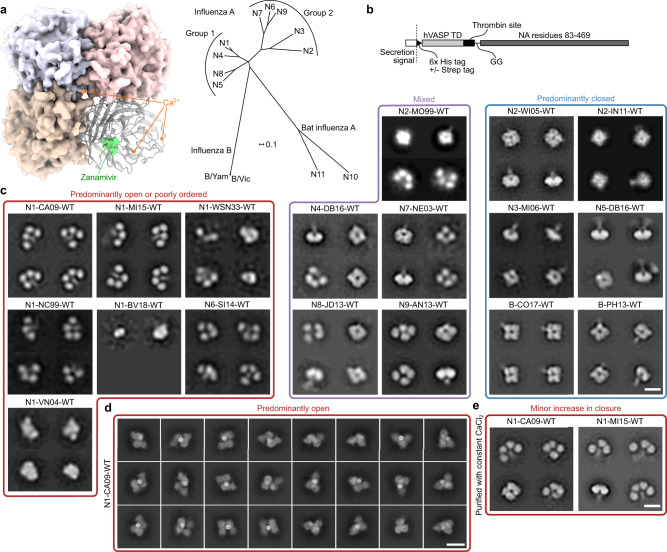
Tetrameric conformations of recombinant NA proteins vary across influenza strains and subtypes. **a** NA structure and phylogeny. (Left) Model of NA structure as observed crystallographically, featuring a bound NA inhibitor (Zanamivir, green) and Ca^2+^ ions (orange). Model from PDB ID 4B7Q with the Ca^2+^ ion near the four-fold axis placed using an alignment with PDB ID 3NSS. (Right) Phylogenetic tree of NA of influenza A and B viruses. Scale bar, 0.1 amino acid substitutions per site. **b** Construct diagram for recombinant NA proteins used in this work. The globular head domain of NA (residues 83–469, N1 numbering) was genetically fused to the hVASP tetramerization domain (TD) by a flexible GG linker and a thrombin protease cleavage site. All constructs contained an N-terminal hexahistidine tag, and some constructs additionally contained a Strep Tag. See Supplementary Data 1 for amino acid sequences. **c** NS-EM 2D class averages of NA (scale bar, 10 nm). (Left) NA preparations from strains that show predominantly open or poorly ordered tetramers. (Middle) NA preparations from strains that show mixtures of closed and open tetramers. (Right) NA preparations from strains that show predominantly closed tetramers similar to those classically observed in NA crystal structures. **d** Cryo-EM 2D class averages of recombinant N1-CA09-WT (scale bar, 10 nm). **e** NS-EM 2D class averages of recombinant N1-CA09-WT and N1-MI15-WT maintained in the presence of 1 mM CaCl_2_ throughout purification (scale bar, 10 nm).

We expressed and purified recombinant soluble NA proteins from every non-bat influenza A subtype and two influenza B viruses using a previously described construct design^52^ in which the NA head domain is genetically fused to the hVASP tetramerization domain^53^ (Fig. 1a, b). The proteins were secreted from mammalian cells and purified from clarified supernatants, yielding homogeneous size exclusion chromatography (SEC) profiles featuring a major peak corresponding to the estimated elution volume of a tetramer, with minimal aggregation (Supplementary Fig. 1a). We then analyzed the purified NAs by negative stain electron microscopy (NS-EM) with two-dimensional (2D) class averaging, a technique commonly used to evaluate the structural integrity of recombinant viral glycoproteins^54–58^. This analysis revealed substantial structural heterogeneity among the various recombinant NAs (Fig. 1c). Two tested NAs each from the N2 subtype and influenza B viruses, as well as representative NAs from N3 and N5, formed “closed” tetrameric structures in which the head domains packed tightly and resembled the four-fold symmetric structure classically observed by X-ray crystallography. In contrast, representative NAs from the N1 and N6 subtypes formed “open” tetramers in which the head domains did not form a single, compact structure. These NAs instead exhibited a clover-like appearance characterized by a thin stalk tethered to independent densities corresponding to the four globular head domains. This result is consistent with the observation that many NAs form monomers when produced recombinantly without a tetramerization domain^44,45^, and suggests that the strength of the tetrameric interface between head domains varies across NA strains and subtypes. Representative NAs from the N4, N7, N8, and N9 subtypes, in addition to a third tested N2 strain, formed mixtures of open and closed tetramers. We also produced and analyzed a previously reported N9 NA construct containing the stabilizing mutation Y170H (Y169aH in N2 numbering)^44^, which, despite showing an improved proportion of the closed state compared to its WT counterpart, still formed a significant population of open tetramers (Supplementary Fig. 1b). Finally, we characterized two N1 and one N2 recombinant NAs from the BEI Resources Repository at the NIH that were also genetically fused to hVASP. The N2 NA was found to adopt the closed conformation and further form rosettes through the hVASP domains, while both N1 NAs appeared open or disordered (Supplementary Fig. 1c). We found no clear correlation between SEC profiles (Supplementary Fig. 1a) or catalytic activity (Supplementary Fig. 1d) and the conformations observed by NS-EM for several recombinant NAs: samples showing predominantly open tetramers remained catalytically active.

To determine whether destabilization of the closed tetrameric conformation was due to the conditions the proteins experienced during NS-EM, the WT NA from the H1N1 strain A/California/07/2009 (abbreviated as N1-CA09-WT) was vitrified and imaged by cryoelectron microscopy (cryo-EM). While 2D classification of the data revealed slightly more compact structures than those obtained by NS-EM, the samples still showed substantial heterogeneity compared to the closed tetrameric structures seen in other subtypes (Fig. 1d). Several of the classes suggested that adjacent head domains formed dimers reminiscent of conformations observed in distantly related proteins with the same six-bladed propeller fold, such as the hemagglutinin (H), hemagglutinin-neuraminidase (HN), and G proteins of paramyxoviruses^59,60^.

We also determined the thermal stability of multiple NAs using nano differential scanning fluorimetry (nanoDSF) by monitoring intrinsic tryptophan fluorescence in buffers containing either 1 mM CaCl_2_ or 5 mM ethylenediaminetetraacetic acid (EDTA) (Supplementary Fig. 2a, b). NAs from representative N3 and N5 (identified as closed by NS-EM) and N8 (mixed) strains were found to have the highest melting temperatures (T_m_), all of which were above 60 °C regardless of buffer additives (Fig. 1f). N8-JD13-WT (A/Jiangxi Donghu/346/2013) had an additional clear yet incomplete transition between 46 and 52 °C depending on the buffer additive, suggesting a partial unfolding event or the unfolding of a minor population of less stable species. Another closed NA, N2-WI05-WT (A/Wisconsin/67/2005), was less stable but also relatively insensitive to Ca^2+^, with T_m_s of 55.1 °C in the presence of Ca^2+^ and 49.0 °C in 5 mM EDTA. In contrast, the open N1-CA09-WT and N1-MI15-WT (A/Michigan/45/2015), and particularly the mixed N4-DB16-WT (A/Redknot/Delaware Bay/310/2016), showed much greater sensitivity to Ca^2+^, with T_m_s below 50 °C in the presence of EDTA becoming 12.5–23 °C higher in the presence of Ca^2+^. Despite their relative phylogenetic similarity, N1-CA09-WT was found to be less thermally stable than N1-MI15-WT, particularly in the presence of Ca^2+^. These data indicate that the thermal stability of recombinant NAs can vary over a wide range, and suggest that Ca^2+^ contributes less to the stability of NAs that tend to form closed tetramers, possibly because head tetramerization has an independent stabilizing effect.

We then evaluated the effects of added Ca^2+^ and the native stalk domain on the tetrameric conformations of soluble recombinant N1 NAs. Although NA contains an interprotomeric Ca^2+^ binding site that thermally stabilizes the protein (ref. ^51^ and above), the addition of 1 mM CaCl_2_ to purified N1-CA09-WT prior to NS-EM did not result in noticeable changes in tetramer closure (Supplementary Fig. 2c). To test whether exposure to Ca^2+^ throughout the entire purification process has an influence on tetramer closure, additional samples of N1-CA09-WT and N1-MI15-WT were purified in the constant presence of 1 mM CaCl_2_ (Fig. 1e). NS-EM of these samples again showed predominantly open tetrameric structures, although a minor population of closed tetrameric species was also observed. Inclusion of the native stalk domain in N1-CA09-WT using a previously described design that also includes hVASP^61^ did not result in closed tetramers in the absence of added Ca^2+^ (Supplementary Fig. 2d), although a partial improvement in the closure was again seen when Ca^2+^ was provided throughout the purification process. Interestingly, nanoDSF of samples treated with Ca^2+^ throughout the purification process (Supplementary Fig. 2e) showed blueshifted baselines relative to samples without Ca^2+^ throughout purification (Supplementary Fig. 2a), suggesting improved burial of tryptophan residues. This was particularly noticeable for N1-CA09-WT with the native stalk, which also showed poorly-defined melting transitions in the absence of added Ca^2+^ (compare Supplementary Fig. 2f to Supplementary Fig. 2a). Together, these data indicate that the open tetrameric state observed by NS-EM in various recombinant NAs, including those with and without the native stalk, cannot be attributed to Ca^2+^ deficiency alone.

In summary, NS-EM analysis was required to recognize distinct tetrameric conformations adopted by different NA antigens, with recombinant N1 NAs in particular showing a tendency to form open tetramers. Treatment with Ca^2+^ was helpful for enhancing the thermal stability of recombinant soluble NAs, but it was not sufficient for fully restoring the closed quaternary conformation.

### Stabilization of the closed tetrameric state of N1 CA09 NA

Although high-resolution structures for NA in its native context in the viral membrane are not yet available, cryoelectron tomographic reconstructions at modest resolution suggest that NA on the surface of H1N1 CA09 virions forms closed tetramers^42^. This observation is further supported by the protective mAb CD6, isolated from a mouse infected with H1N1 virus, which binds a quaternary epitope spanning two subunits of the NA tetramer^62^. Inspired by these indications that NA forms closed tetramers on the virion surface and the potential utility of recombinant NA antigens with improved stability, homogeneity, and native-like structure, we set out to stabilize N1 NA in the closed tetrameric state using structure-based design.

**Fig. 2:**
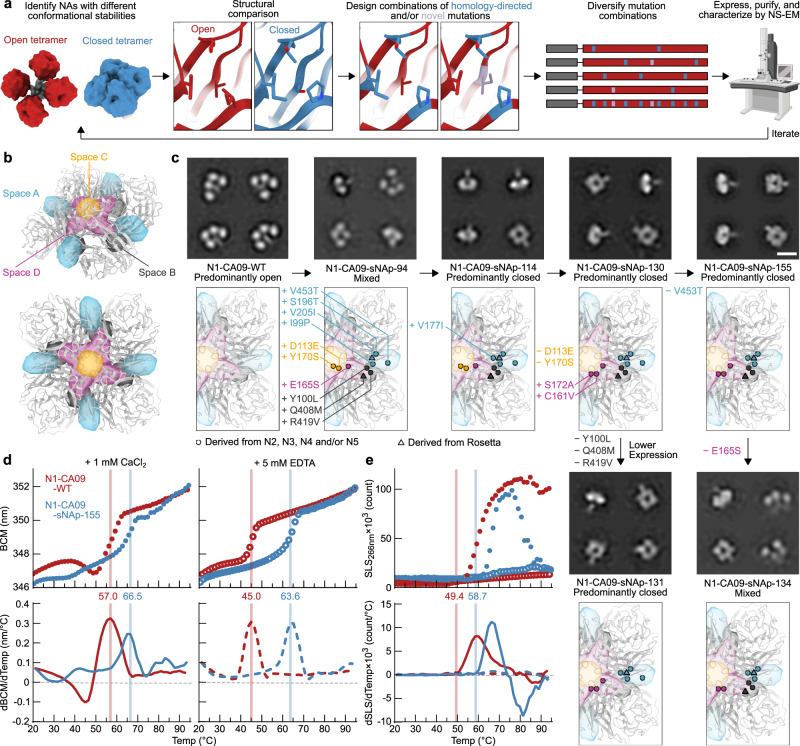
Stabilization of the closed tetrameric state of N1 CA09 NA. **a** Design and analysis pipeline for stabilization of closed tetrameric NAs using homology-directed mutations and computational protein design. Models constructed using PDB entries 4B7Q, 6BR5, and 1USE. Electron microscope image was generated by BioRender. **b** Structural depiction of the four different spaces in NA targeted for design. Two views of the CA09 NA tetramer are shown. **c** NS-EM 2D class averages (top) and designed mutations (bottom) in a series of CA09 NA mutants that exhibit varying degrees of tetramer closure (scale bar, 10 nm), and explore the roles of spaces A, B, and D in tetramer closure and expression (scale bar, 10 nm). 2D class averages for N1-CA09-WT are the same as presented in Fig. 1c. **d** Thermal denaturation of N1-CA09-WT and N1-CA09-sNAp-155 in the presence of 1 mM CaCl_2_ (closed circles and solid lines) or 5 mM EDTA (open circles and dashed lines), monitored by intrinsic tryptophan fluorescence. Top panels show raw data, while lower panels show smoothed first derivatives used to calculate melting temperatures. The barycentric mean (BCM) of the fluorescence emission spectra is plotted. **e** SLS during thermal denaturation of N1-CA09-WT and N1-CA09-sNAp-155 in the presence of 1 mM CaCl_2_ or 5 mM EDTA, with aggregation temperatures shown for CaCl_2_-treated samples. Data are plotted as in panel d.

The inter-protomer interface of N1-CA09-WT (PDB ID 4B7Q) has 3,172 Å^2^ of buried surface area between each protomer, but features many aqueous cavities and non-ideal polar contacts. We attempted to stabilize the inter-protomeric interface of N1-CA09 tetramers using homology-directed mutations inspired by NAs we identified as closed, as well as novel mutations identified purely by structural modeling (Fig. 2a). Based on our data indicating that recombinant N2, N3, and N5 NAs form predominantly closed tetramers, we analyzed amino acid differences at the interface between representative NAs from each of these subtypes and N1-CA09-WT. We also included N4 NA in this analysis even though it was characterized as only partially closed, as its conformational stability was better than N1-CA09-WT and the N4 subtype is phylogenetically closest to N1. To organize our design strategy, we identified residues involved in inter-protomer interactions by visual inspection and categorized them into four “spaces” (A, B, C, and D) that spanned the entire interface (Fig. 2b, see Supplementary Table 1 for lists of residues in each space). We developed a computational pipeline for homology-directed design using the Rosetta macromolecular modeling software^63,64^ in which potential interface-stabilizing mutations derived from closed NAs were symmetrically modeled both individually and in combination within each space. In parallel, design trajectories that allowed Rosetta to identify novel mutations without restriction to naturally observed substitutions were performed. Thirty-two designs were selected for experimental characterization, comprising designs with mutations restricted to each individual space as well as combined across multiple spaces.

Genes for each stabilized NA protein (sNAp) were synthesized as genetic fusions to the hVASP tetramerization domain (Fig. 1b) and transfected into mammalian cells to assess expression. Nine designs that were detectable in cell culture supernatants by SDS-PAGE were purified by affinity chromatography and analyzed by NS-EM (see Supplementary Data 1 for amino acid sequences). Of these nine designs, only one (N1-CA09-sNAp-94) contained a substantial population of closed structures, with approximately half of the particles classified as closed based on the 2D class averages (Fig. 2c). To improve this design, one additional N2- and N3-inspired mutation was added that appended an individual methyl group to a valine in space A (V177I; N1-CA09-sNAp-114), which is distal to the C4 axis and near the CD6 binding site, resulting in predominantly closed particles by NS-EM with only a minor fraction of particles in the open state still detectable (Fig. 2c, Supplementary Data 2).

Two additional rounds of design were performed to revert unproductive mutations from N1-CA09-sNAp-114 while maintaining closure and maximizing expression levels (see Supplementary Table 2 for summaries of mutations and expression levels). Space C, which includes residues immediately surrounding the C4 axis, featured mutations derived from N4 NA that could be reverted without deleterious effects to the closed conformation. Furthermore, the N5-inspired combination of C161V and S172A was added to remove an unpaired cysteine and slightly repack space D, adjacent to space C on the “underside” of NA, which greatly improved yield (N1-CA09-sNAp-130). N1-CA09-sNAp-155, which reverted an extra mutation at the surface-exposed position 453 of N1-CA09-sNAp-130, maintained closure with the similar yield to N1-CA09-WT and had its stabilizing mutations maximally buried to maintain proper antigenicity (Fig. 2c). Relative to N1-CA09-WT, N1-CA09-sNAp-155 features four mutations in space A, three in space B (on the underside of NA distal to the C4 axis), and three in space D. sNAp-130 and sNAp-155 were henceforth used interchangeably as representative sNAps for N1 CA09.

While the V177I mutation suggested that improved packing in space A assists closure, the specific contributions of other mutations were less clear since they were introduced simultaneously in N1-CA09-sNAp-94. Reversion of the three space B mutations in N1-CA09-sNAp-130 did not decrease tetrameric closure (N1-CA09-sNAp-131), although it did reveal a role for these mutations in increasing sNAp yield (Fig. 2c, Supplementary Table 2). Strikingly, reversion of the single N5-derived space D mutation E165S from N1-CA09-sNAp-155 resulted in a roughly equal mixture of open and closed tetramers (N1-CA09-sNAp-134). Together, these data demonstrate that combining mutations in spaces A and D can drive the complete closure of CA09 NA.

We found that the tetramer-stabilizing mutations in N1-CA09-sNAp-155 increased the T_m_ by 9.5 and 19.1 °C in the presence of CaCl_2_ and EDTA, respectively, when compared to N1-CA09-WT (Fig. 2d). Sensitivity to Ca^2+^ was substantially decreased, with only a 2.9 °C difference between Ca^2+^- and EDTA-containing conditions. This finding is consistent with our earlier observation that the thermal stability of closed tetramers tended to be less dependent on Ca^2+^ (Supplementary Fig. 2a, b). Monitoring static light scattering (SLS) also revealed an increase in aggregation temperature for N1-CA09-sNAp-155 of 9.7 °C in the presence of Ca^2+^ (Fig. 2e). Interestingly, neither protein aggregated in the presence of EDTA. To assess conformational stability over time, N1-CA09-sNAp-155 was analyzed by NS-EM after storage for 6, 10, and 15 days at 4 °C and showed retention of the closed state during this period (Supplementary Fig. 3a). Hydrogen-deuterium exchange mass spectrometry (HDX-MS) was used to collect data on a small set of deuterium-exposed peptides from both N1-CA09-WT and N1-CA09-sNAp-130, which indicated multiple regions of differential stability (Supplementary Fig. 3b). Several peptides were identified near the inter-protomer interface and the catalytic pocket that showed decreased deuterium uptake for the sNAp, including one peptide that includes the main catalytic residue (Y402) and another peptide containing the substrate-binding residue R368. One peptide (residues 215–224) was more dynamic in the sNAp, which includes a solvent-exposed loop and part of a beta-strand that is adjacent to two stabilizing mutations (S196T and V205I). Overall, most of the changes observed by HDX-MS showed a reduction in solvent accessibility or an increase in local ordering consistent with the sNAp adopting a more “closed” conformation compared to N1-CA09-WT. Both N1-CA09-sNAp-130 and N1-CA09-sNAp-155 retained catalytic activity, although they were less active than N1-CA09-WT (Supplementary Fig. 3c). Finally, the stabilizing mutations in N1-CA09-sNAp-130 also resulted in predominantly closed tetramers when transferred to recombinant constructs containing the complete stalk domain (Supplementary Fig. 3d). Compared to the equivalent design lacking stabilizing mutations (Supplementary Fig. 2f), nanoDSF showed significantly higher T_m_s and more defined melting transitions, with 13.5 °C and 18.3 °C increases in T_m_ in the presence of Ca^2+^ and EDTA, respectively (Supplementary Fig. 3e).

In all, our stabilization of N1 CA09 showed that a combination of structure- and homology-based design can both conformationally stabilize the closed tetrameric state and improve thermal stability and local ordering. The designed stabilizing mutations were functional with or without the inclusion of the native NA stalk, and mutations in both spaces A and D seemed most important for conformational stability.

### Structural and bioinformatic analysis of closed state stabilizing mutations

**Fig. 3:**
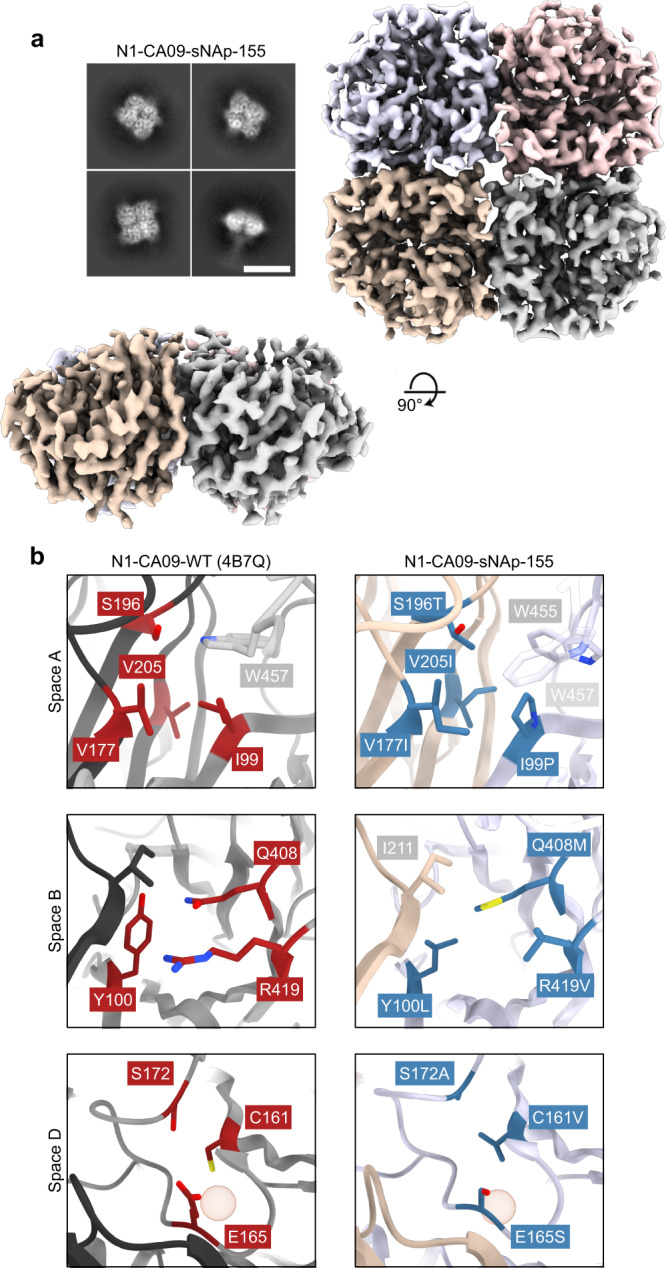
Cryo-EM structure of N1-CA09-sNAp-155. **a** Cryo-EM 2D class averages for N1-CA09-sNAp-155 showing fully closed tetramers (scale bar, 10 nm) (top left). Cryo-EM map, colored by individual head domain, viewed down the four-fold symmetry axis (top right) and an orthogonal view (bottom left). **b** The three major regions targeted for mutation: WT N1-CA09 (PDB 4B7Q, left), with residues targeted for mutation indicated (red). Space A (top), Space B (middle), and space D (bottom). Corresponding regions in the N1-CA09-sNAp-155 single-particle reconstruction (right), with mutated residues indicated (blue) and non-mutated residues involved in packing colored by chain as in panel **a**. The red sphere highlights the position of a cavity in space D.

**Fig. 4:**
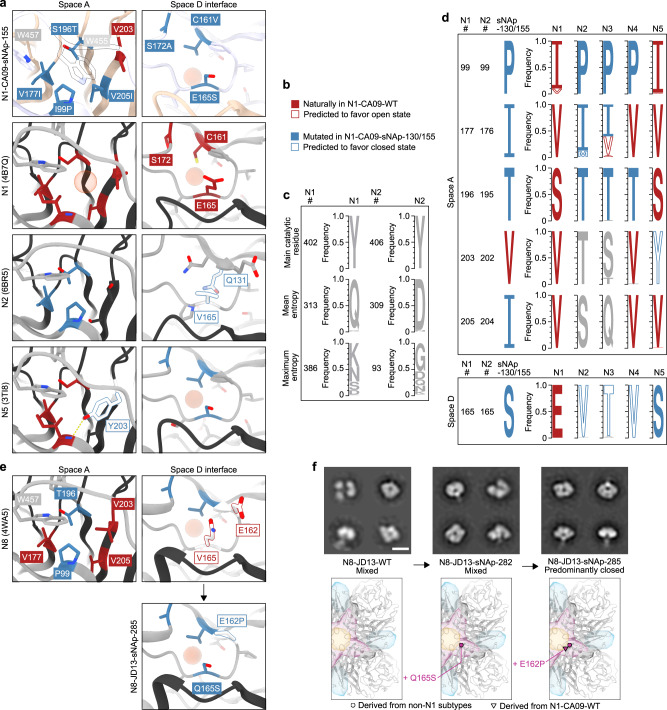
Structural and bioinformatic analysis of closed state-stabilizing mutations. **a** Zoomed-in views of space A- and D-stabilizing mutations in the N1-CA09-sNAp-155 cryo-EM structure and corresponding regions in predominantly open N1 (PDB ID 4B7Q) and predominantly closed N2 (PDB ID 6BR5) and N5 (PDB ID 3TI8) NAs. The red spheres highlight the positions of cavities in space A or space D. **b** Descriptions of color labels which apply to panels a and d. **c** Sequence logos of a highly conserved residue (the main catalytic residue), a representative position with an entropy near the mean for the whole protein, and the position of maximum entropy within both the N1 and N2 subtypes are shown for comparison with panel d. **d** Sequence logos for N1-CA09-sNAp-155 and naturally occurring NAs at positions that can alter NA tetrameric conformation. All data for panels a and d is derived from a compiled database of all unique sequences from each subtype prior to December 31st, 2019. **e** (Top) Spaces A and D in a representative crystal structure of an N8 NA (PDB ID 4WA5) and (bottom) design model for space D of N8-JD13-sNAp-285. The red sphere highlights the position of a cavity in space D. **f** Mutation schematics and NS-EM comparing N8-JD13-WT, N8-JD13-sNAp-282, and N8-JD13-sNAp-285 (scale bar, 10 nm). NS-EM was performed and analyzed multiple times and representative images are shown.

To better understand the roles of our stabilizing mutations, we determined a cryo-EM structure of N1-CA09-sNAp-155 at 3.2 Å resolution (Fig. 3, Supplementary Fig. 4a–g, and Supplementary Table 3). 2D class averages obtained from samples that were purified and vitrified in the absence of supplemental Ca^2+^ exclusively showed closed NA tetramers resembling those observed in crystal structures, in contrast to our earlier cryo-EM 2D class averages of N1-CA09-WT (Fig. 1d). The 3D reconstruction of N1-CA09-sNAp-155 confirmed its adoption of the closed tetramer in solution (Fig. 3a). The structure is highly similar to N1 CA09 crystal structures (1.18 and 1.83 Å backbone root-mean-square deviation (RMSD) to PDB 4B7Q over one and all four chains of the tetramer, respectively). The main differences were in a few solvent-exposed loops that were poorly ordered in N1-CA09-sNAp-155, including the C-terminal region from residues 458–469 (Supplementary Fig. 4h), the ‘150-loop’ consisting of residues 145–150, and the ‘430-loop’ consisting of residues 429–437 (Supplementary Fig. 4i). The apparent flexibility of the 150- and 430-loops is consistent with previous structural observations and computational modeling of these regions^65–67^. Additionally, a backbone segment adjacent to the space A mutations was slightly rearranged such that W455 packed closely against the introduced mutations and W457 moved away from its native position at the interface (Fig. 3b). Despite this rearrangement, none of the mutated positions in spaces A, B, and D showed substantial changes in their main chain coordinates and, other than their interactions with W455 and W457, the intended interactions were formed. For example, P99 and T196 (T195 in N2 numbering) in space A form a central hydrophobic contact across the interface that closely matches the same interaction seen in the NA structures used for homology-directed design (Figs. 3b and 4a). Likewise, I177 (I176 in N2 numbering) provides additional hydrophobic packing in both N1-CA09-sNAp-155 and the NAs of other subtypes relative to N1 CA09, while I205 adds further hydrophobic packing that was favored by Rosetta. In space B, the Rosetta-derived Q408M and the homology-inspired Y100L and R419V simultaneously provide improved hydrophobic packing and decrease the number of partially buried polar groups at the interface.

In contrast to intuitive stabilization mechanisms such as the cavity-filling mutations employed in space A, the N5-inspired space D mutation E165S does not have an obvious structural explanation. Crystal structures of N2, N3, N4, and N5 NAs all show less bulky residues at position 165, including S, T, and V (Fig. 4a). Interestingly, in all known NA structures a substantial cavity exists adjacent to the 160-loop and near the inter-protomeric interface (Fig. 4a), raising the possibility that the E165S mutation may function by helping preorganize this region during tetramer assembly. Several N2 NA structures showed partial filling of this cavity through a T131Q mutation (e.g., PDB 6BR5; Fig. 4a), which could also partially explain the improved conformational stability of NAs from this subtype.

To better understand NA sequence-structure relationships, a comprehensive dataset of unique NA sequences from all available influenza A viruses isolated before December 31, 2019, was compiled, and the mutational frequencies of key positions in spaces A and D were evaluated within each subtype. As expected, some variation was observed in the amount of sequence diversity at each position, ranging from universal conservation of the tyrosine nucleophile in the catalytic site (Y402 and Y406 in N1 and N2 numbering, respectively) to high diversity at several surface-exposed positions (e.g., position 386 in N1 NAs) (Fig. 4c). However, sequence diversity within each subtype was generally low: positions of mean entropy in N1 and N2 NAs, for which far greater numbers of unique sequences were available compared to the animal-derived N3, N4, and N5 subtypes, showed ~97% conservation of the predominant amino acid.

Fewer than 0.05% of the 4,422 animal and 13,735 human N1 NA sequences included in our dataset contained any of the five stabilizing mutations at positions 99, 165, 177, 196, and 205 (Fig. 4d). The vast majority of sequences instead possessed the less stable identities observed in N1-CA09-WT. Position 99 was the most variable, with valine present in ~9.8% of the sequences. We predict that both isoleucine and valine at position 99 favor the open state, and 99.9% of N1 NA sequences contain one of these two residues. NAs from N2 (dataset of 8,888 animal and 23,121 human sequences), N3 (942 animal sequences), and N4 (242 animal sequences) are also highly conserved at most of these positions and share multiple features that are distinct from N1 NAs, despite the close phylogenetic relationship between N1 and N4 (Fig. 1a). For example, P99 and T196 (195 in N2 numbering) are nearly completely conserved in all three subtypes. These residues form a consistent hydrophobic contact and play key roles in the sNAp designs. In contrast, position 177 (176 in N2 numbering) in space A is more variable in N2 and N3 NAs, with multiple identities appearing frequently, while N4 has a universally conserved valine at this position. N2-IN11-WT (A/Indiana/10/2011), which we found to be predominantly closed (Fig. 1c), contains a methionine at position 176, showing that this substitution can also favor the closed state similarly to isoleucine. N5 NAs have converged on a different solution in space A, maintaining complete conservation of the distinctive Y203, which both fills the cavity and accepts a hydrogen bond from the backbone amide of residue 99, thereby achieving the same outcome as P99 and T196 (Fig. 4a, d). In space D, further similarities are observed among the more stable subtypes (N2, N3, N4, and N5) at position 165, with less flexible smaller or beta-branched amino acids (A/S/T/V/I) found in >99.9% of all sequences in these subtypes, with valine, serine, and threonine most prevalent by far.

These structural and bioinformatic analyses provide sequence-based signatures that appear to explain the conformational differences we observed in recombinant NAs by NS- and cryo-EM. Specifically, the combination of a small side chain at position 165 and a well-packed space A correlates with the stability of the closed tetrameric conformation.

### Design of stabilized closed tetrameric N8 NA

Although our finding that recombinant N8-JD13-WT forms a mixture of open and closed tetramers (Fig. 1c) is consistent with a previous report showing that N8 NAs can form monomers and dimers, a number of infection-elicited N8 NA-specific antibodies recognized a quaternary epitope^23^, providing an incentive for tetramer stabilization. We applied the lessons learned from stabilizing N1 CA09 to generate closed N8 NA tetramers. Analysis of existing N8 NA crystal structures showed hydrophobic packing in space A similar to that of N4 NAs, featuring P99 and T196. However, space D contained the large, polar Q165 and a notable difference from other subtypes with a glutamic acid at position 162 instead of a proline (Fig. 4e). This combination of a well-packed space A and a more “open-like” space D appeared to explain its heterogeneous conformational phenotype. Although Q165S alone (N8-JD13-sNAp-282) was insufficient, the combination of E162P and Q165S (N8-JD13-sNAp-285) provided complete stabilization of the closed state of N8 as assessed by NS-EM, further emphasizing the cooperative effects of spaces A and D on stabilization (Fig. 4f). While N8-JD13-sNAp-285 showed only small improvements in its primary T_m_ in the presence of either Ca^2+^ or EDTA relative to N8-JD13-WT, the stabilizing mutations minimized an earlier partial unfolding transition at 45–55 °C, consistent with its more homogeneous adoption of the closed state (Supplementary Fig. 5a). A similar effect on T_agg_ was observed (Supplementary Fig. 5b). However, conformational evaluation over time by NS-EM showed that, in contrast to N1-CA09-sNAp-155, tetramer closure of N8-JD13-sNAp-285 decreased over the course of a week at 4 °C, indicating that further conformational stabilization should be possible (Supplementary Fig. 5c). Finally, N8-JD13-sNAp-285 was still catalytically active, although less so than N8-JD-WT (Supplementary Fig. 5d).

### Mutation of stabilizing residues in N2 NA prevents closed tetramer formation

Given the clear stabilizing effects of key positions in spaces A and D, we hypothesized that mutating them to amino acids observed in more open subtypes would destabilize the closed N2-WI05-WT tetramer. Mutations were made separately in each space to make space A more strongly resemble WT N1 NAs and space D resemble N1 or N8 NAs, while avoiding steric clashes (Supplementary Fig. 5e). The V165E mutation in space D alone was sufficient to induce global structural changes including dissociation of the heads, but also led to the formation of soluble aggregates. V165Q resulted in a more subtle effect, causing about half of the tetrameric particles to adopt the open state while the rest remained closed. In space A, an I177V/T196S (N1 numbering, see Supplementary Table 1 for conversions to N2 numbering) double mutant remained closed, but the addition of P99I led to the formation of unassembled monomers and aggregates. We next combined V165Q with I177V and T196S and found that the protein formed either open tetramers or lower-order oligomers (Supplementary Fig. 5e). Thermal denaturation of this triple mutant destabilized NA protein (desNAp), N2-WI05-desNAp-255, showed a decreased T_m_, a strongly redshifted baseline in its intrinsic fluorescence emission spectrum, and no apparent stabilization by Ca^2+^ (Supplementary Fig. 5f). Enzymatic activity was also abolished in N2-WI05-desNAp-255 (Supplementary Fig. 5g). Together, these data indicate that the same design rules that enabled stabilization of the closed tetrameric state of N1 CA09 and N8 JD13 could be applied in reverse to destabilize the closed state of N2 WI05.

### Design of additional closed recombinant N1 NA tetramers

**Fig. 5:**
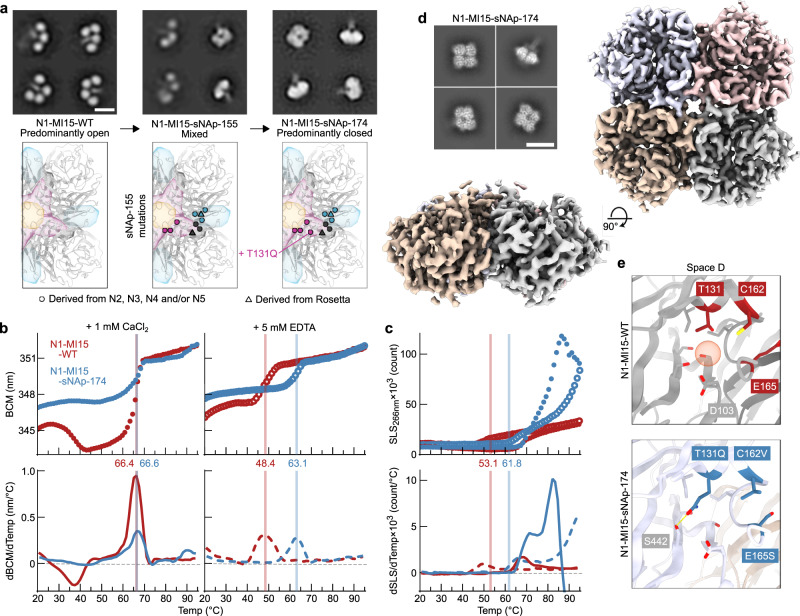
Design and Structural Characterization of N1-MI15-sNAp-174. **a** Application of sNAp-155 mutations to N1 MI15 and addition of the space D stabilizing mutation T131Q resulted in fully closed N1-MI15-sNAp-174 tetramers (scale bar, 10 nm). NS-EM was performed and analyzed multiple times and representative images are shown. **b** Thermal denaturation of N1-MI15-WT and N1-MI15-sNAp-174 in the presence of 1 mM CaCl_2_ (closed circles and solid lines) or 5 mM EDTA (open circles and dashed lines), monitored by intrinsic tryptophan fluorescence. Top panels show raw data, while lower panels show smoothed first derivatives used to calculate melting temperatures. The barycentric mean (BCM) of the fluorescence emission spectra is plotted. **c** SLS during thermal denaturation of N1-MI15-WT and N1-MI15-sNAp-174 in the presence of 1 mM CaCl_2_ or 5 mM EDTA, with aggregation temperatures shown for CaCl_2_-treated samples. Data are plotted as in panel B. **d** Representative 2D class averages (scale bar, 10 nm) (top left) and final 3D density (bottom left and right) from cryo-EM of N1-MI15-sNAp-174. **e** Space D cavity in N1-MI15-WT (top; from PDB ID 4B7Q) and N1-MI15-sNAp-174 (bottom; cryo-EM reconstruction), with the cavity marked by a red sphere. All residues shown as sticks in the WT model are conserved between N1-CA09-WT and N1-MI15-WT.

To assess the portability of the closed state-stabilizing mutations, mutations from N1-CA09-sNAp-155 were introduced into the NA of N1 MI15, from the same post-2009 human lineage as CA09. We found that the sNAp-155 mutations clearly assisted stabilization of the closed state but were insufficient for complete closure, as N1-MI15-sNAp-155 adopted a mixture of the open and closed states (Fig. 5a).

Given our hypothesis that E165S favors closure by making space D more ordered, we focused on redesigning the aqueous cavity surrounding D103, which is directly adjacent to residue 165 and another potential source of structural instability, in order to stabilize N1 MI15. This cavity is adjacent to, but not immediately at, the interprotomer interface. After generating a homology model of N1-MI15 using PDB ID 4B7Q as a template, we applied our structure- and homology-guided design strategy to this space. Among 20 designs building on N1-MI15-sNAp-155, four were found to predominantly favor the closed state, with three showing exclusively closed particles in NS-EM 2D class averages (Fig. 5a and Supplementary Fig. 6a). These designs utilized an N2-inspired V166P mutation along with complementary cavity filling from V114I (N1-MI15-sNAp-165 and -176), an N2-inspired T131Q mutation that provides both improved packing and hydrogen bonding in the cavity (N1-MI15-sNAp-174 and -176), and homology-directed hydrophobic filling of the cavity (N1-MI15-sNAp-183). Due to the higher yield of N1-MI15-sNAp-174 compared to the other designs and the simplicity of the single additional stabilizing mutation (T131Q; Fig. 5a and Supplementary Table 2), this design was selected for further analyses. Mirroring the results obtained with other sNAps, the stability of N1-MI15-sNAp-174 was relatively insensitive to Ca^2+^ and the T_m_ of the sNAp was 14.7 °C higher than N1-MI15-WT in the presence of EDTA (Fig. 5b). The T_agg_ was also higher in the presence of either Ca^2+^ or EDTA (Fig. 5c), and N1-MI15-sNAp-174 maintained the closed tetrameric state over a period of 15 days (Supplementary Fig. 6b). It remained catalytically active, although at reduced levels compared to its open WT counterpart (Supplementary Fig. 6c). The success of these designs established multiple sets of mutations that stabilize closed N1 NA tetramers—including several that do not make direct contact across the interface—and highlight the cavity in space D as a key determinant of closed tetramer stability.

We determined the structure of the N1-MI15-sNAp-174 tetramer at 3.2 Å resolution using cryo-EM (Fig. 5d, Supplementary Fig. 7a–g, and Supplementary Table 3). The structure, which strongly resembled the overall structure of N1-CA09-sNAp-155 (backbone RMSD of 0.77 and 0.80 Å over one and all four subunits of the tetramer, respectively), confirmed that N1-MI15-sNAp-174 adopts the closed tetrameric state in solution. The backbone rearrangement in space A observed in the N1-CA09-sNAp-155 structure was also found in N1-MI15-sNAp-174, along with a similar lack of density for the C-terminal regions, the 150-loop, and the 430-loop (Supplementary Fig. 7h, i). The new T131Q mutation helps fill the space D cavity as intended while forming a hydrogen bond to the side chain of S442 (Fig. 5e).

We next applied the sNAp-155 mutations to more distantly related N1 NAs from the avian H5N1 strain A/Vietnam/1203/2004 (VN04) and a pre-pandemic lineage of human H1N1, A/WSN/1933 (WSN33). The resultant N1-VN04-sNAp-155 and N1-WSN33-sNAp-155 formed predominantly open tetramers (Supplementary Fig. 6d, e). We reasoned that making the sequences of these sNAps more like N1-CA09-sNAp-155 and N1-MI15-sNAp-174 overall should stabilize them in the closed tetrameric state. We therefore made substitutions at other buried positions in N1 VN04 and N1 WSN33 that differed from N1 CA09 and N1 MI15, an approach similar to the Repair-and-Stabilize strategy recently developed for stabilization of HIV-1 Env trimers^68^, and combined these with mutations used to stabilize N1 CA09 and N1 MI15. N1-VN04-sNAp-354, which combined the T131Q substitution in space D that stabilized N1-MI15-sNAps with mutation of three nearby residues to the amino acids in N1 CA09 and N1 MI15 (I106V, V163I, and A166V), formed exclusively closed tetramers as determined by NS-EM (Supplementary Fig. 6d).

The sequence of N1 WSN33 NA differs from N1 CA09 and N1 MI15 at many positions in both the space D cavity and the rest of the tetrameric interface. In addition to the sNAp-155 mutation set, the substitution of six residues across spaces A, B, and D to the identities of N1 CA09 and N1 MI15 (G105S, I106V, A157T, V163I, A166V, and R210G; N1-WSN33-sNAp-366) substantially improved tetramer closure (Supplementary Fig. 6e), demonstrating that there are inherent differences in interface stability between N1 WSN33 and N1 CA09/N1 MI15 that can be altered by focusing purely on the interface and the space D cavity. The addition of T131Q to this set of mutations resulted in a sNAp (N1-WSN33-sNAp-367) that further improved tetramer closure, while hydrophobic packing mutations in the cavity (N1-WSN33-sNAp-375) were less effective. To determine if sequence differences distant to the interface contribute to closed tetramer formation, we changed eight buried residues distributed throughout the globular head of N1-WSN-sNAp-375 which differed from N1 CA09 to the amino acids in N1 CA09, resulting in the predominantly closed N1-WSN-sNAp-378 (Supplementary Fig. 6e). These data suggest that while spaces A and D are key for closure, packing interactions distant from the interface can remotely influence interface-stabilizing mutations. Transfer of general packing residues from one strain to another thus offers an additional strategy for stabilization while maintaining native surface antigenicity. Together, the strategies we provide should be widely applicable across many N1 NAs as well as NAs from other subtypes.

### Effects of N1 NA Tetramer Stabilization on Viral Fitness and Antigenicity

**Fig. 6:**
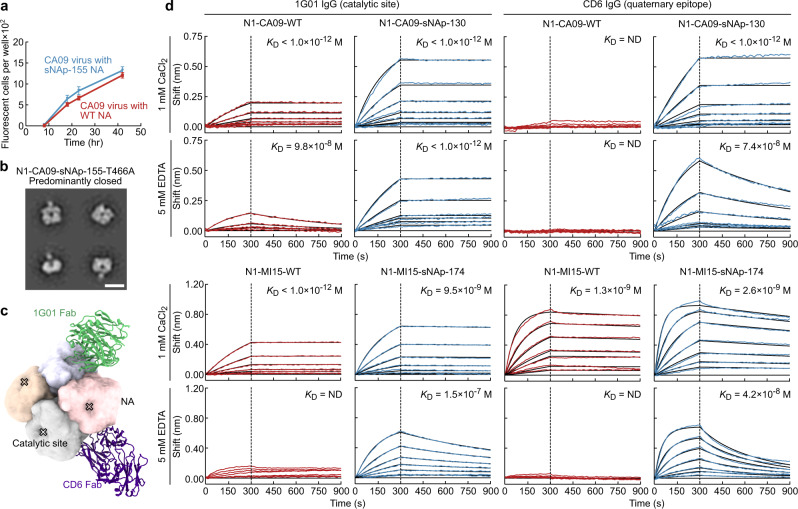
Effects of N1 NA tetramer stabilization on viral fitness and antigenicity. **a** Growth curve of A/California 07/2009 H1N1 influenza reporter viruses with WT NA and NA with sNAP-155 mutations on MDCK-SIAT1-PB1 cells. Error bars denote the mean and error of the standard deviation (SD) of measurements from *n* = 64 individual wells at each time point. The experiment was repeated twice, and representative results are shown. **b** NS-EM of a recombinant soluble N1-CA09-sNAp-155 with the T466A mutation acquired during growth of the R3ΔPB1 A/California 07/2009 H1N1 sNAp-155 virus (scale bar, 10 nm). NS-EM preparation was performed once. **c** Models of Fabs of murine CD6 (purple) and human 1G01 (green) bound to CA09 NA. CD6 binds a quaternary epitope that spans two protomers, while 1G01 binds within and around the catalytic pocket. Models were generated by aligning crystal structures of CD6 (PDB ID 4QNP) and 1G01 (PDB ID 6Q23) Fabs to a single tetramer of CA09 NA (PDB ID 4B7Q). **d** Binding kinetics of anti-NA mAbs to N1-CA09-WT, N1-CA09-sNAp-130, N1-MI15-WT, and N1-MI15-sNAp-174. Biolayer interferometry sensorgrams of 1G01 IgG (left) and CD6 IgG (right) binding to N1-CA09-WT (red, top row), N1-CA09-sNAp-130 (blue, top row), N1-MI15-WT (red, bottom row), and N1-MI15-sNAp-174 (blue, bottom row). Upper and lower sensorgrams within each row were measured in buffers containing 1 mM CaCl_2_ and 5 mM EDTA, respectively. Experimental data (colored traces) were fitted (black lines) with the binding equations describing a 2:1 interaction.

We next tested whether mutations that stabilize the closed tetrameric state impact viral fitness by generating CA09 H1N1 reporter influenza viruses^69^ with and without the N1-CA09-sNAp-155 mutations in the NA gene by reverse genetics, and observed similar growth kinetics over 42 hours postinfection (Fig. 6a). Sequencing revealed that the virus carrying N1-CA09-sNAp-155 acquired a T466A mutation in the stabilized NA, and both viruses acquired mutations in HA (Supplementary Table 4). We found that recombinant soluble N1-CA09-sNAp-155 with the T466A mutation adopted the closed tetrameric state, confirming that this mutation does not affect the tetramer closure (Fig. 6b). These results demonstrate that tetramer-stabilizing mutations are compatible with virus growth, which is consistent with the closed tetramer being the biologically relevant form of the protein on the viral surface.

To further understand the relevance of the closed and open states, we compared the antigenicity of WT NA proteins and sNAps. The murine mAb CD6, isolated from a mouse infected with a sublethal dose of A/California/07/2009 (H1N1) virus, recognizes a quaternary epitope that spans two neighboring NA protomers^62^. CD6 is highly protective and potently inhibits viral replication in vitro. The second mAb, 1G01, isolated from a human immediately after H3N2 virus infection, is the broadest anti-NA antibody described to date, providing protection against many influenza A and B viruses by targeting the highly conserved catalytic site^36^ (Fig. 6c). We first used flow cytometry to assess the binding of fluorescently labeled CD6 and 1G01 to HEK293F cells transfected with plasmids encoding full-length N1-CA09-WT and N1-CA09-sNAp-130 constructs with native N-terminal cytosolic domains, transmembrane domains, and stalks that bore C-terminal myc tags to enable assessment of surface expression levels. Both CD6 and 1G01 unambiguously bound to both the WT and stabilized membrane-anchored NAs in the presence or absence of 5 mM EDTA (Supplementary Fig. 8). The similar binding of CD6—which targets a quaternary epitope—to both NAs is again consistent with the closed tetramer being the biologically relevant form that is present on viral membranes.

We next measured antibody binding affinities to the soluble versions of N1-CA09-WT and N1-CA09-sNAp-130 by biolayer interferometry. While both proteins were recognized by 1G01 IgG with similar affinity in the presence of CaCl_2_, the antibody bound to N1-CA09-WT with much lower affinity than to N1-CA09-sNAp-130 in the presence of EDTA (Fig. 6d). Strikingly, the quaternary epitope-specific CD6 IgG minimally bound N1-CA09-WT regardless of the presence of Ca^2+^, indicating that the CD6 epitope was not properly formed in the open N1-CA09-WT. By contrast, the same antibody readily and avidly bound to N1-CA09-sNAp-130 in the presence or absence of Ca^2+^. The binding data for the N1 CA09 NAs are consistent with our NS-EM data, which showed that Ca^2+^ alone was insufficient for stabilizing the closed states of these antigens. WT and stabilized soluble versions of N1 MI15 were also analyzed. Although 1G01 and CD6 binding to the WT and stabilized versions of N1-MI15 was not substantially different in the presence of Ca^2+^, we again observed strong binding of both mAbs to only N1-MI15-sNAp-174 in the presence of EDTA (Fig. 6d). In comparison with our earlier NS-EM analysis, the data for the N1-MI15 antigens indicate that N1-MI15-WT can adopt the closed tetrameric state—or perhaps dimers of adjacent protomers—in the presence of both Ca^2+^ and CD6, but not either of the two alone, while the stabilized N1-MI15-sNAp-174 homogeneously adopts the closed tetrameric state regardless of Ca^2+^.

Together, these data suggest that the head dissociation observed in recombinant soluble NAs is far less frequently sampled in membrane-anchored NAs. Stabilization of the closed tetrameric state of NA through structure-based design therefore enables the generation of recombinant antigens that more accurately reflect the biologically relevant conformation of the protein.

## Discussion

Influenza NA has been an attractive yet temperamental target for universal influenza countermeasures. We have shown that recombinant soluble NA tetramers from several different influenza strains and subtypes adopt an open tetrameric conformation despite the presence of an exogenous tetramerization domain and excess Ca^2+^, while others form the crystallographically observed closed tetramer. These data may help explain reports of different stabilities among NAs from various subtypes^50^ and are consistent with observations that NA protomers have a tendency to dissociate^23,44^. However, to our knowledge, no previously published work has characterized the open tetrameric conformation adopted by many recombinant soluble NAs. The open conformation we observed is similar to those found in distantly related tetrameric viral glycoproteins, leaving open the possibility that this conformation has biological relevance^59,60^. Nevertheless, several lines of evidence suggest that NA forms closed tetramers on the surface of influenza virions, and our work provides additional evidence in support of this view while also highlighting the importance of the closed state for stability and binding to protective infection-elicited antibodies. Such differences between the native transmembrane and recombinant soluble forms of glycoproteins are not uncommon. For example, many class I fusion proteins require stabilizing mutations to maintain their native—and often metastable—prefusion conformations when produced recombinantly^70^.

Inspired by knowledge of the sequences and structural phenotypes of homologous NAs, we stabilized the closed tetrameric state using a homology-directed design strategy similar to the recently reported Repair-and-Stabilize approach applied to HIV-1 envelope trimers^68,71^. We identified several structural features that work together to stabilize the closed NA tetramer: direct interactions between subunits at the tetrameric interface, preorganization of the tetrameric interface within each subunit by intra-protomeric interactions near the interface, and general stabilization of the structure of each subunit by packing interactions distal to the interface. Two regions, which we refer to as spaces A and D, appear to have a particularly strong influence on NA stability and conformation. Multiple naturally occurring stabilizing and destabilizing mutations in space D have been described previously^10,51^, and comparisons to homologous NAs led to a single stabilizing mutation in space C in an N9 NA which facilitated structure determination of NA–antibody complexes^44^. Whereas mutations in space A and the space D cavity stabilize the closed tetrameric state by improving atomic packing in these interfaces or interface-proximal regions, the mechanism of other mutations is less clear. For example, there is no obvious structural rationale for the stabilizing effect of the E/Q165S mutation, yet this position—which stood out when sequence alignments of several NA subtypes were compared—is a critical determinant of tetramer closure. The success of homology-based mutations reflects the sensitivity and complexity of the determinants of NA stability and provides a roadmap towards the design of additional stabilizing mutations to NAs from diverse influenza strains and subtypes.

Our findings have several implications for previous and future studies of influenza NA. We found that several commonly used assays—SEC, enzymatic activity, and thermal stability—were generally unable to distinguish between the open and closed forms of the enzyme due to inherent differences between NAs from different strains. Like several previous studies of other viral glycoprotein antigens^39,40,54–58,72–74^, structural characterization by EM was required to establish the conformational state of most samples. Although antigenic characterization using protective mAbs that bind a quaternary epitope or the catalytic site also clearly distinguished the open and closed forms, only a few such mAbs are currently available, limiting the diversity of NAs that can be characterized in this way. We anticipate that with the identification of additional NA-specific mAbs with the desired properties, antigenic characterization could become a rapid, widely accessible, and powerful tool for assessing NA tetrameric conformation.

Furthermore, our data suggest that the recombinant soluble NA proteins used in many previous studies, while possibly presumed to be in the crystallographically observed closed tetrameric state based on biochemical assays, may have been partially or wholly in the open conformation. This disconnect could lead to erroneous interpretations of experimental outcomes, most particularly for serological measurements, and could limit the discovery of valuable mAbs. If so, our understanding of NA antigenicity and immunogenicity may be incomplete or compromised, particularly for NAs from the highly relevant but less stable N1 subtype. While Ca^2+^ treatment has been frequently used for NA stabilization^45,51,75,76^, our work emphasizes that Ca^2+^ alone is likely insufficient for completely repairing the loss of conformational integrity in many recombinant soluble NAs. Stabilizing mutations provide an alternative approach to improving conformational integrity, resulting here in several recombinant soluble NAs that homogeneously adopt the closed tetrameric conformation that, to the best of our knowledge, is present on influenza virions. These improvements are maintained even in the presence of EDTA, which could prove particularly useful for proper maintenance of conformational epitopes when sNAps are exposed to assay or biological conditions that lack adequate Ca^2+^ concentrations.

Our work provides a design-based strategy for overcoming the inherent conformational instabilities of recombinant soluble NA proteins to improve their conformational and antigenic profiles. As highlighted by the transformational effects of the development of prefusion-stabilized class I viral fusion proteins^39,70,77,78^, the availability of stable, native-like recombinant soluble glycoproteins can broadly catalyze research and development efforts, including serological assays, monoclonal antibody isolation, structure determination of antigen-antibody complexes, immunogen design, and vaccine manufacturing.

## Methods

### Cell lines

Expi293F cells, a female human embryonic kidney cell line adapted to grow in suspension, were obtained from Thermo Fisher Scientific (Catalog A14527). Expi293F cells were cultured in Expi293 Expression Medium (Life Technologies) at 37 °C, 8% CO_2_, and shaking at 150 RPM. 293T-PB1 cells used for virus rescue were kindly donated from Jesse Bloom (Fred Hutchinson Cancer Research Center). MDCK-SIAT1-PB1 cells used for virus propagation were described previously^69^. The parental MDCK-SIAT1 cells were purchased from Millipore Sigma (Catalog 05071502). Cell lines were maintained in DMEM supplemented with 10% FBS, and for MDCK-SIAT1-PB1 cells, geneticin (1 mg ml^−1^) and puromycin (0.25 µg ml^−1^) was further added.

### Protein expression and purification

Sequences for all NA proteins are provided in Supplementary Data 1. The cytoplasmic, transmembrane, and stalk domains of the wild-type (WT) NA (residues 1–82, N1 numbering) were replaced by affinity purification tags, a tetramerization domain derived from human vasodilator-stimulated phosphoprotein (hVASP)^53^, a thrombin protease cleavage site, and a two-residue GG linker. NA constructs were expressed by transient transfection in Expi293F cells (ThermoFisher Scientific) at a density of 2.5 × 10^6^ cells/ml using the ExpiFectamine^TM^ 293 Transfection Kit (ThermoFisher Scientific), or alternatively at a density of 3.0 × 10^6^ cells/ml using PEI-MAX (Polyscience). Prior to protein purification, the supernatants were harvested 5 days post-transfection and centrifuged at 3,700 x g to remove cell debris. Proteins were purified from clarified supernatants by immobilized metal affinity chromatography (IMAC) using either Ni^2+^- or Co^2+^-containing resin. For Ni^2+^-based IMAC, clarified supernatants were incubated for 2 h at room temperature with Ni^2+^ Sepharose High-Performance histidine-tagged protein purification resin (Cytiva) and bound protein eluted using 50 mM Tris pH 8.0, 0.5 M NaCl, 300 mM imidazole. For Co^2+^-based resin, clarified supernatants were flowed over Talon resin (Takara), and bound protein eluted in 20 mM Tris pH 8.0, 300 mM NaCl, 300 mM imidazole. Eluted proteins were further purified by SEC into phosphate-buffered saline (PBS) or 25 mM Tris pH 8.0, 150 mM NaCl, 5% glycerol using a Superdex 200 Increase 10/300 column (Cytiva). For proteins that were purified in the constant presence of CaCl_2_, 1 mM CaCl_2_ was added to the wash and elution buffer. Eluted proteins were further purified by SEC into a buffer containing either 25 mM Tris pH 8.0, 150 mM NaCl, 5% glycerol and 1 mM CaCl_2_, or 50 mM Tris pH 8.0, 0.5 M NaCl and 1 mM CaCl_2_. Recombinant NA proteins derived from A/California/7/2009 (H1N1) (Catalog #NR-19234), A/New Caledonia/20/1999 (Catalog #NR-43779) and A/Wisconsin/67/2005 (Catalog #NR19237) were obtained from BEI Resources

### Negative stain EM sample preparation and analysis

NS-EM and particle image averaging were used to assess whether the head domains of recombinant NA proteins adopted the open or closed tetrameric structure. Proteins were diluted to between 0.01–0.02 mg mL^−1^ using either 10 mM HEPES pH 7.0, 150 mM NaCl or 10 mM Tris pH 7.5, 150 mM NaCl, either with or without 1 mM CaCl_2_. Samples were adsorbed to glow-discharged carbon-coated copper grids. The grids were either washed with a drop of the same buffer three times and stained with 0.75% uranyl formate, or blotted and stained directly with 0.75% uranyl formate. Images were recorded with sampling ranging between 1.9 Å/pixel and 2.2 Å/pixel, depending on the microscope. Data were collected on either an FEI Tecnai T20 electron microscope equipped with an FEI Eagle CCD camera and operated at 200 kV using SerialEM^79^, or on an FEI Tecnai 12 Spirit 120 kV electron microscope equipped with a Gatan Ultrascan 4000 CCD camera. Particles were selected from the micrographs automatically using either in-house software (Yaroslav Tsybovsky, unpublished) or were picked in a reference-free manner in cisTEM^80^. For the latter datasets, particles were extracted after correcting for the effect of the CTF for each micrograph with cisTEM^80^. Depending on user, dataset, and microscope, particles were either extracted into 120 × 120-pixel boxes with a final pixel size of 2.2 Å/pixel, or extracted into 230 × 230-pixel boxes with a final pixel size of 2.2 Å/pixel, or extracted with a box size of 176 × 176 pixels and binned to a final box size of 44 × 44 pixels (to a pixel size of 6.4 Å/pixel). Reference-free 2D classification was performed using either Relion 1.4 or Relion 3.1 (refs. ^81,82^). Representative 2D class averages for all datasets were chosen based on the unambiguous identification of NA particle quality and conformational state, with qualitative descriptions based on overall class average appearance and/or semiquantitative measurements of particles assigned to each class when distinct populations could be reliably measured. A designation of “predominantly closed” NA variants were assigned to samples with 2D class averages of the closed state comprising >80% of well-resolved NA tetramers, with open or poorly resolvable classes containing the remaining <20% of particles. “Mostly closed” was assigned to NA variants where roughly three-quarters of particles were classified into the closed conformation. “Mostly open” was assigned when roughly one-quarter of particles were observed as closed. “Predominantly open” was assigned for samples with clearly >80% of resolvable particles being classified in an open conformation. “Mixed” was assigned to any sample in between “mostly closed” and “mostly open”. Representative electron micrographs and complete sets of 2D class averages are provided in Supplementary Data 2 for each NA.

### Thermal denaturation and static light scattering

Non-equilibrium melting temperatures were determined using an UNcle (UNchained Labs) based on the barycentric mean of intrinsic tryptophan fluorescence emission spectra collected from 20–95 °C using a thermal ramp of 1 °C per minute in buffers of 25 mM Tris pH 8.0, 150 mM NaCl and 5% glycerol supplemented with either 1 mM CaCl_2_ or 5 mM EDTA. Static light scattering (SLS) data were simultaneously collected. Melting temperatures were defined as the maximum point of the first derivative of the melting curve, with first derivatives calculated using GraphPad Prism software after smoothing with four neighboring points using 2nd order polynomial settings. Aggregation temperatures were defined as the first positive data point above baseline of the first derivative of SLS, with the first derivative similarly calculated using GraphPad Prism.

### Enzymatic activity

Neuraminidase activity was measured with the NA-Fluor^TM^ Influenza Neuraminidase Assay Kit (ThermoFisher Scientific) according to the manufacturer’s protocol, which contains 4 mM CaCl_2_ in final preparations used for measurement. Briefly, two-fold serial dilutions of each NA protein were made in a black 96-well, flat bottom plate, starting from protein stocks at concentrations of 25–50 µg/mL. The wells in column 12 were left empty for background controls. NA-Fluor Substrate was prepared according to the manufacturer’s protocol and added to each well. Plates were incubated for 1 h at 37 °C and reactions were stopped with NA-Fluor Stop Solution. Plates were read using an excitation wavelength range of 350–365 nm and an emission wavelength range of 440–460 nm. Background control wells were subtracted for each protein serial dilution. Protein concentrations were plotted versus relative fluorescence unit (RFU) values.

### Structure-based design using Rosetta

All calculations in Rosetta were made using versions v2017.18-dev59451, v2019.21-dev60746, or v2019.45-dev61026. For design of N1 CA09 sNAps, residue positions were manually sorted into spaces A, B, C, and D based on perceived local interactions in the inter-protomeric interface of N1-CA09-WT (PDB ID 4B7Q). The sequence of PDB ID 4B7Q contains an additional Y351F mutation compared to the sequence used for N1-CA09-WT, which was maintained in all designs. Based on either amino acid appearing at this position in multiple closed and open constructs described in this manuscript, we do not expect that this mutation has an impact on closure. Representative structures of N2 (PDB ID 6BR5), N3 (PDB ID 4HZV), N4 (PDB ID 2HTV), and N5 (PDB ID 3TI8) NAs were analyzed and residue identities at each of the positions were recorded. The four-fold symmetry axis of N1-CA09-WT was aligned with [0,0,1] and a single protomer was saved in.pdb format. A design protocol was written using RosettaScripts^63^ that takes the aligned protomer and a custom resfile as inputs, with the resfile dictating the side chain identities and conformations sampled during design (Supplementary Data 3). Briefly, the protocol applies two rounds of design based on the input resfile, with side-chain and backbone energy minimization applied after each design step. Both design and minimization steps were allowed to repack or minimize residues within 5 Å of all mutable or packable residues listed in the resfile. Multiple resfiles were set up for each space. One set of resfiles was designed to place specific substitutions from N2 (PDB ID 4H52), N3, N4, or N5 NA structures into N1-CA09-WT using the ‘PIKAA’ option. Other sets used the ‘PIKAA’ option to allow Rosetta’s packer to choose between residue identities from the N1, N2, N3, N4, or N5 NAs. A final set used the ‘PIKAA’ option to add further residue identities that were not observed in the N1, N2, N3, N4, or N5 structures to identify novel mutations. Design models and scores were manually inspected to identify interactions across the interface that appeared structurally feasible. Mutations were discarded if they buried polar groups that were natively solvent-exposed or involved in hydrogen bonds. To prevent undesired alterations to antigenicity, mutations to surface-exposed residues were not frequently considered. Favorable interactions were iteratively retested in resfiles and manually refined to finalize a diverse set of designs for each space. Twenty-six designs targeted individual spaces, while six designs consisted of combinations of designs to all four spaces that were manually picked and combined.

A slightly modified protocol was used to design N1 MI15 sNAps that contained additional mutations in space D. Due to the lack of a crystal structure for N1 MI15, N1 CA09 (PDB-ID 4B7Q) was used as a template for a homology model. All design trajectories were performed while adding sNAp-155 mutations (I99P, Y100L, C161V, E165S, S172A, V177I, S196T, V205I, Q408M, R419V) in addition to mutational differences between N1 CA09 and N1 MI15 (N200S, V241I, N248D, V264I, N270K, I314M, I321V, N369K, N386K, K432E). Beyond those mutations, the design process focused entirely on residues within space D with resfile inputs drawn from either crystal structures of other non-bat NA subtypes or other identities not naturally observed in all subtypes. Disulfide mutations were designed outside of Rosetta based on distances between pairs of residues.

### Hydrogen-deuterium exchange mass spectrometry

For each time point, 40 pmol of N1-CA09-WT and N1-CA09-sNAp-130 were incubated in deuterated buffer (85% D_2_O, pH* 7.4) for 3, 60, 1,800, or 72,000 s at room temperature and subsequently mixed with an equal volume of ice-cold quench buffer (4 M urea, 200 mM tris(2-chlorethyl) phosphate (TCEP), 0.2% formic acid) to a final pH* of 2.5. Samples were immediately frozen in liquid nitrogen and stored at 80 °C until analysis. Fully deuterated samples were prepared by digesting 40 pmol of undeuterated sample over a pepsin column, followed by concentration under vacuum, resuspension in deuterated buffer at 65 °C for 1 hour, and then quenching/freezing. Zero time point samples were prepared as previously described^83^. A 3 s exchange was performed at the beginning and end of the longest exchange reaction to monitor protein stability under the experiment conditions. Online pepsin digestion was performed and analyzed by LC-MS-IMS utilizing a Waters Synapt G2-Si Q-TOF mass spectrometer as previously described^83^. Deuterium uptake analysis was performed using HX-Express v2 (refs. ^84,85^). The relative deuterium exchange was corrected for in-exchange using the zero time point. Internal exchange standards (Pro-Pro-Pro-Ile [PPPI] and Pro-Pro-Pro-Phe [PPPF]) were included in each reaction to ensure that conditions were consistent throughout all of the labeling reactions.

### Cryo-electron microscopy sample preparation, data collection, and image processing

sNAp proteins were diluted to 1–1.5 μM in buffer (10 mM Tris, pH 7.5, 150 mM NaCl) and 3 μL sample loaded onto a freshly glow-discharged 1.2/1.2 UltrAuFoil grid (300 mesh) prior to plunge freezing using a vitrobot Mark IV (ThermoFisher Scientific) with a blot force of −1 and 3.5–4.5 s blot time at 100% humidity and 4 °C.

Data were acquired on an FEI Glacios transmission electron microscope operated at 200 kV and equipped with a Gatan K2 Summit direct detector. Automated data collection was carried out using Leginon^86^ at a nominal magnification of 36,000× with a pixel size of 1.16 Å. The dose rate was adjusted to 8 counts/pixel/s, and each movie was acquired in counting mode fractionated in 50 frames of 200 ms. For the N1-CA09-sNAp-c155 and N1-MI15-sNAp-174 complexes, 1,034 and 1,099 micrographs were collected, respectively, with a defocus range between −0.5 and −2.5 µm. For the N1-CA09-WT complex, 605 micrographs were collected with a defocus range between −1.0 and −3.0 µm. Movie frame alignment, estimation of microscope CTF parameters, and automatic particle picking and extraction were carried out using Warp^87^. Particles were extracted unbinned into a box size of 204 pixels.

For the N1-CA09-WT dataset, three rounds of reference-free 2D classification were performed using cryoSPARC^88^. After each round, well-defined images corresponding to NA tetramers were selected and re-classified until 2D classification stabilized. For the N1-CA09-sNAp-c155 and N1-MI15-sNAp-c174 datasets, two rounds of reference-free 2D classification were performed in cryoSPARC, again selecting for well-defined images. Particles selected post-2D classification were subjected to two rounds of 3D classification in Relion^89^ without imposing symmetry (angular sampling 7.5° for 25 iterations followed by 1.8° with local searches for a further 25 iterations). For each dataset, ab initio models were generated in cryoSPARC as reference maps for 3D classification^88^. Refinements of 3D maps were carried out using non-uniform refinement along with per-particle defocus refinement as implemented in cryoSPARC^90^. Particles were transferred back into Relion to perform Bayesian polishing^81,91^ before an additional round of non-uniform refinement, per-particle defocus refinement, and finally one more round of non-uniform refinement imposing four-fold symmetry. Local resolution estimation, filtering, and sharpening were carried out in cryoSPARC^92^. All reported resolutions were determined using gold-standard Fourier shell correlation (FSC) calculations with a cutoff criterion of 0.143 (ref. ^93^) and FSC curves were corrected for the effects of soft masking by high-resolution noise substitution^94^.

### Cryo-EM model building and analysis

UCSF Chimera^95^ and Coot^96^ were used to fit atomic models (PDB ID 4B7Q) into the cryoEM maps and point mutations were made manually in Coot. Models were refined and relaxed using Rosetta using both sharpened and unsharpened maps^97,98^ and validated using Molprobity^99^, Phenix^100^, and EMRinger^101^. Figures were generated using UCSF ChimeraX^102^.

### Collection of NA sequences and entropy calculations

Comprehensive sequence datasets for each NA subtype were downloaded from GISAID (www.gisaid.org). Each dataset included animal and human sequences longer than 1,350 nucleotides deposited before January 14, 2020. Sequences were aligned using Mafft v7 for large numbers of short sequences^103^. Sequences with more than 135 ambiguous bases or large gaps were eliminated. To calculate amino acid frequencies, we truncated NA sequences to retain only the globular head of NA ectodomain. Unique amino acid sequences of the NA head domain were selected using CD-HIT with the “Sequence identity cut-off” set at 1.0 (ref. ^104^). Sequence logos were generated using WebLogo 3 (ref. ^105^), http://weblogo.threeplusone.com/). From aligned sequences containing gaps, entropy calculations were measured using the dms_tools2 python library (https://jbloomlab.github.io/dms_tools2/). Representative positions for maximum and mean entropies were the individual positions with entropy values that were highest or closest to the mean calculated using all positions in a given sequence, respectively.

### Biolayer interferometry

All biosensors were hydrated in PBS prior to use. Recombinant NA tetramers were immobilized on HIS2 biosensors (fortéBio) through their hexahistidine tags. After briefly dipping in assay buffer (25 mM Tris pH 8, 150 mM NaCl, 5% glycerol, 1% BSA) supplemented with either 1 mM CaCl_2_ or 5 mM EDTA, the biosensors were dipped in a two-fold dilution series of IgG for 5 min. Starting concentration of 1G01 and CD6 was 400 nM and 100 nM, respectively. Biosensors were then dipped in the assay buffer (with either CaCl_2_ or EDTA) to allow IgG to dissociate from NA for 10 min. All assay steps were performed at 30 °C with agitation set at 1,000 rpm in the Octet HTX instrument (fortéBio). Correction to subtract non-specific baseline drift was carried out by subtracting the measurements recorded for a sensor loaded with the NA in the same buffer with no antibody. Data analysis and curve fitting were carried out using Octet analysis software (version 11). Experimental data were fitted with the binding equations describing a 2:1 (bivalent binding) interaction. Global analyses of the complete data sets assuming binding was reversible (full dissociation) were carried out using nonlinear least-squares fitting allowing a single set of binding parameters to be obtained simultaneously for all concentrations used in each experiment.

### Virus generation

A/California 07/2009 H1N1 influenza reporter virus was rescued by reverse genetics of eight-plasmid system with HA and NA segments of CA09 and internal gene segments of A/WSN/1933 except for PB1 segment which was replaced with reporter gene (tdKatushka2). To generate the virus with stabilized NA, NA segment was modified to incorporate sNAP-155 mutations and used the modified NA segment for virus rescue in 293 T cells transiently expressing viral PB1 and TMPRSS2. Both viruses with WT NA and with stabilized NA were propagated in MDCK-SIAT1 cells constitutively expressing PB1 as described^69^. Both viruses were passaged ten times prior to assessing growth kinetics. For virus growth kinetics, pretitrated viruses were added to MDCK-SIAT1-PB1 cells (preseeded at 1 × 10^5^ cells/ml) in OptiMEM with 1 µg/ml of TPCK-treated trypsin in a 384-well black plate (final 50 µl/well) with the transparent bottom (Greiner). Viral growth was measured at 8, 18, 21, 23, and 42 hours postinfection by counting fluorescent foci using Celigo Image Cytometer (Nexcelom) with a customized red channel for optimal detection of the tdKatushka2 reporter. In Celigo operation and analysis software v4.1, Target 1 protocol was used to detect and count fluorescent foci.

### Flow cytometry

Cells were transfected as described earlier in Methods using PEI-MAX and harvested 24 hours post-transfection. Cells were collected, passed through a 70 µm nylon mesh filter, and counted for concentration and viability according to the manufacturer’s instructions (ThermoFisher Countess). Cells were split into two groups per sample and washed twice with buffer (25 mM HEPES, 150 mM NaCl, 2.5% glycerol) or the same buffer supplemented with 5 mM EDTA (buffer-EDTA). Cells were incubated with 1 µL ZombieViolet (Biolegend #423114) per 1 million cells for 10 minutes at room temperature. Cells were washed twice in buffer or buffer-EDTA, both containing 1% w/v BSA. Cells were aliquoted into 96-well flow cytometry plates (2 × 10^5^ cells per well) and centrifuged. The supernatant was removed and the cells were resuspended with buffer containing 1% BSA. A 1:400 dilution of antimyc antibody (9E10; Abcam ab202008), 200 nM CD6 or 1G01 antibody fluorescently labeled according to the manufacturer’s instructions (Thermo Fisher A20188), or both antimyc antibody and one of the anti-NA antibodies was added to the resuspended cells. Samples were incubated at 4 degrees C for 30 minutes, washed twice, incubated in 4% paraformaldehyde for 10 minutes at room temperature, washed twice, and stored on ice until analysis on an Attune NxT flow cytometer. The flow cytometry data was analyzed with FlowJo version 10.

### Reporting summary

Further information on research design is available in the Nature Research Reporting Summary linked to this article.

## Supplementary information

Supplementary Information

Description of Additional Supplementary Files

Supplementary Data 1

Supplementary Data 2

Supplementary Data 3

Reporting Summary

## Data Availability

All images and data were generated and analyzed by the authors, and will be made available by the corresponding authors (N.P.K., and M.K.) upon reasonable request. Raw negative-stain EM images and complete set of 2D class averages are provided in Supplementary Item 2. Structural models and density maps are deposited in the Protein Data Bank and Electron Microscopy Data Bank under accession numbers PDB 7U2Q and EMD-26318 (N1-CA09-sNAp-155) and PDB 7U2T and EMD-26319 (N1-MI15-sNAp-174). Source data are provided with this paper.

## Source data

Source Data
