# Supplementary material for: Structure-based design of stabilized recombinant influenza neuraminidase tetramers
Source: Nat Commun. 2022 Apr 5;13:1825. doi: 10.1038/s41467-022-29416-z (PMC8983682; doi:10.1038/s41467-022-29416-z)
Supplement: Supplementary file 1 — Supplementary Information [file 41467_2022_29416_MOESM1_ESM.pdf]

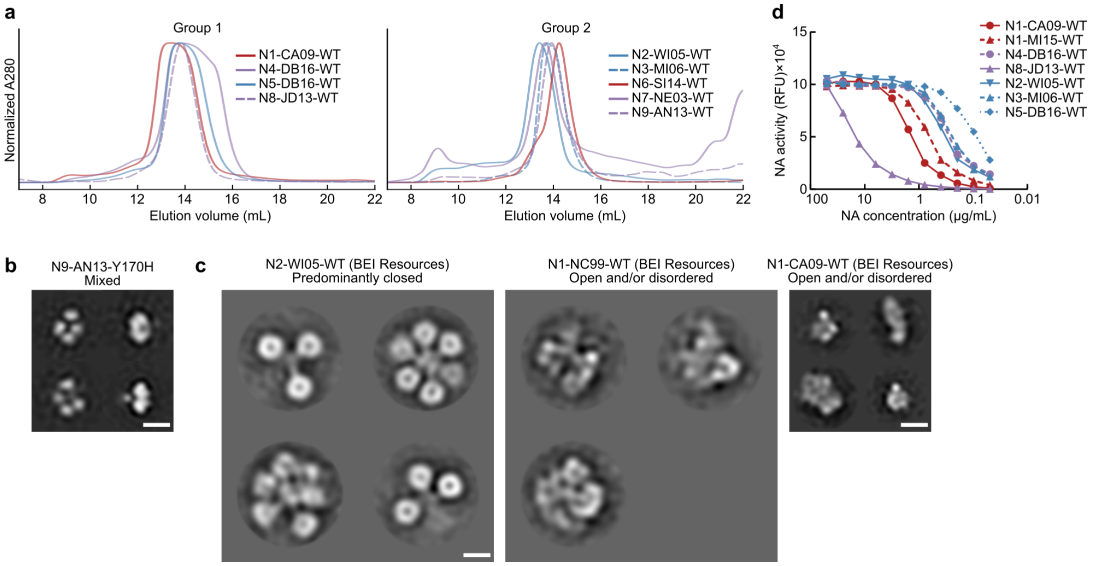

Supplementary Fig. 1. NS-EM, Preparative SEC, and enzymatic activity of recombinant WT and Y170H-containing NA tetramers. **a** Preparative SEC of recombinant NAs from all nine non-bat influenza A subtypes on a Superdex 200 Increase 10/300 GL column. **b** NS-EM 2D class averages of recombinant N9-AN13 NA containing the previously identified Y170H stabilizing mutation (scale bar, 10 nm). **c** NS-EM 2D class averages of recombinant N2-WI05-WT, N1-NC99-WT, and N1-CA09-WT NAs obtained from BEI Resources (scale bar, 10 nm). **d** Enzymatic activity of purified recombinant NAs was determined with the NA-star assay. RFU, relative fluorescence units. Experiment was repeated at least twice, with a representative result shown.

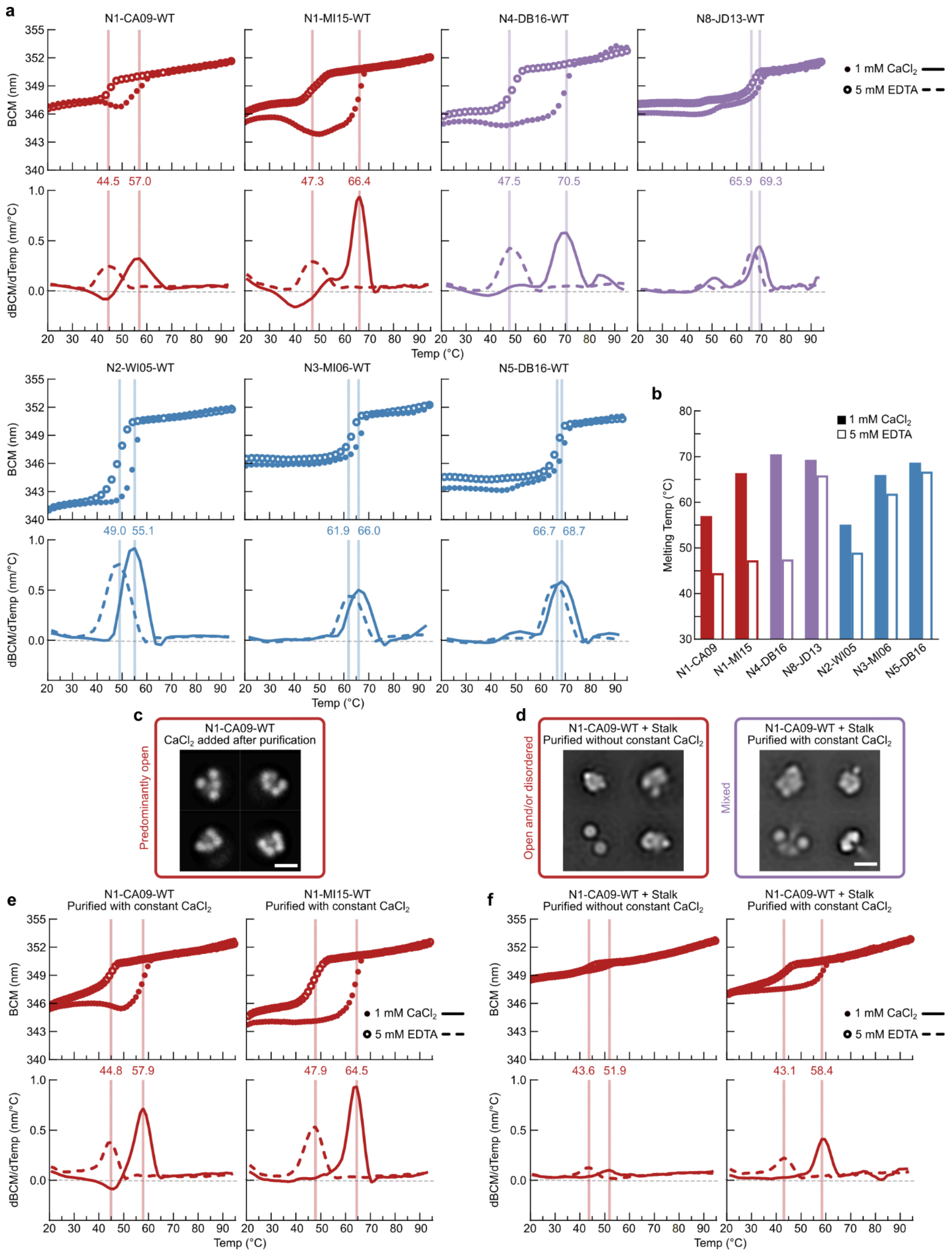

Supplementary Fig. 2. Analysis of recombinant NAs by nanoDSF, and NS-EM analysis of effects of supplemented $CaCl_2$ and/or the inclusion of the native stalk domain. **a** Thermal denaturation of WT NA tetramers in the presence of 1 mM $CaCl_2$ (closed circles and solid lines) or 5 mM EDTA (open circles and dashed lines), monitored by intrinsic tryptophan fluorescence. The barycentric mean (BCM) of the fluorescence emission spectra is plotted. Top panels show raw data, while lower panels show smoothed first derivatives used to calculate melting temperatures, which are indicated by vertical lines. **b** Bar graph representation of melting temperatures shown in **a**. **c** NS-EM 2D class averages of recombinant N1-CA09-WT in the presence of 1 mM $CaCl_2$ which was added after protein purification (scale bar, 10 nm). **d** NS-EM 2D class averages of recombinant N1-CA09-WT with the native stalk added, either after purification with or without 1 mM $CaCl_2$ (scale bar, 10 nm). **e** Thermal denaturation of N1-CA09-WT and N1-MI15-WT samples which were purified in the constant presence of 1 mM $CaCl_2$ (related

to Fig. 1e). Samples containing 5 mM EDTA also contain residual $CaCl_2$ at 0.9 mM. Data are presented as in **a**. **f** Thermal denaturation of N1-CA09-WT with the native stalk added, after purification with or without 1 mM $CaCl_2$. Samples containing 5 mM EDTA also contain residual $CaCl_2$ at 0.9 mM. Data are presented as in **a**.

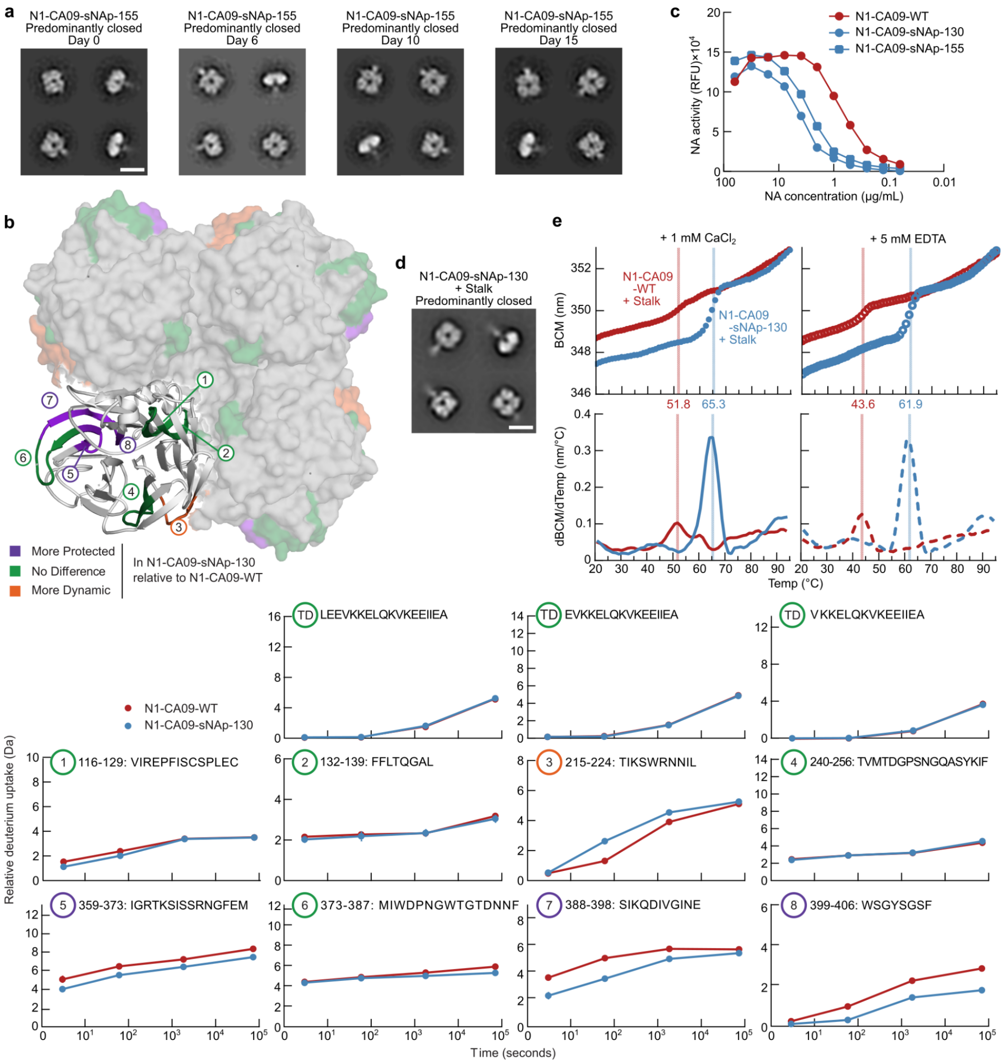

Supplementary Fig. 3. Shelf-life stability, hydrogen-deuterium exchange, NS-EM, nanoDSF, and catalytic activity of unmodified and stabilized N1 CA09 NA tetramers. **a** Shelf-life stability of N1-CA09-sNAp-155 over 15 days at 4°C as assessed by NS-EM (scale bar, 10 nm). **b** (Top left) Peptide segments, numbered 1 to 8, mapped onto the structure of N1 NA (PDB ID 4B7Q). Colors indicate whether the peptide is more protected (purple) or more dynamic (orange) in N1-CA09-sNAp-130 compared to N1-CA09-WT. Green indicates peptides where there is no difference in deuterium exchange between variants. (Bottom) Kinetics of hydrogen-deuterium exchange for numbered peptides and peptides from the tetramerization domain (TD) at multiple timepoints up to 20 h. Each point is an average of duplicate measurements (N1-CA09-WT) and triplicate measurements (N1-CA09-sNAp-130), except for a limited number of replicates that were discarded due to low signal to noise. Standard deviations for all measurements were smaller than the points plotted. **c** Enzymatic activity of N1-CA09-WT, N1-CA09-sNAp-130, and N1-CA09-sNAp-155 determined with the NA-star assay. RFU, relative fluorescence units. Experiment was repeated at least twice, with a representative result shown. Performed in duplicate, with error bars smaller than the plotted points and not shown. **d** NS-EM of N1-CA09-sNAp-130 with the native stalk domain added (scale bar, 10 nm). NS-EM was prepared and analyzed multiple times, representative images are shown. **e** Thermal denaturation of N1-CA09-WT and N1-CA09-sNAp-130 with native stalk domains. Data are presented as in Fig. 2b.

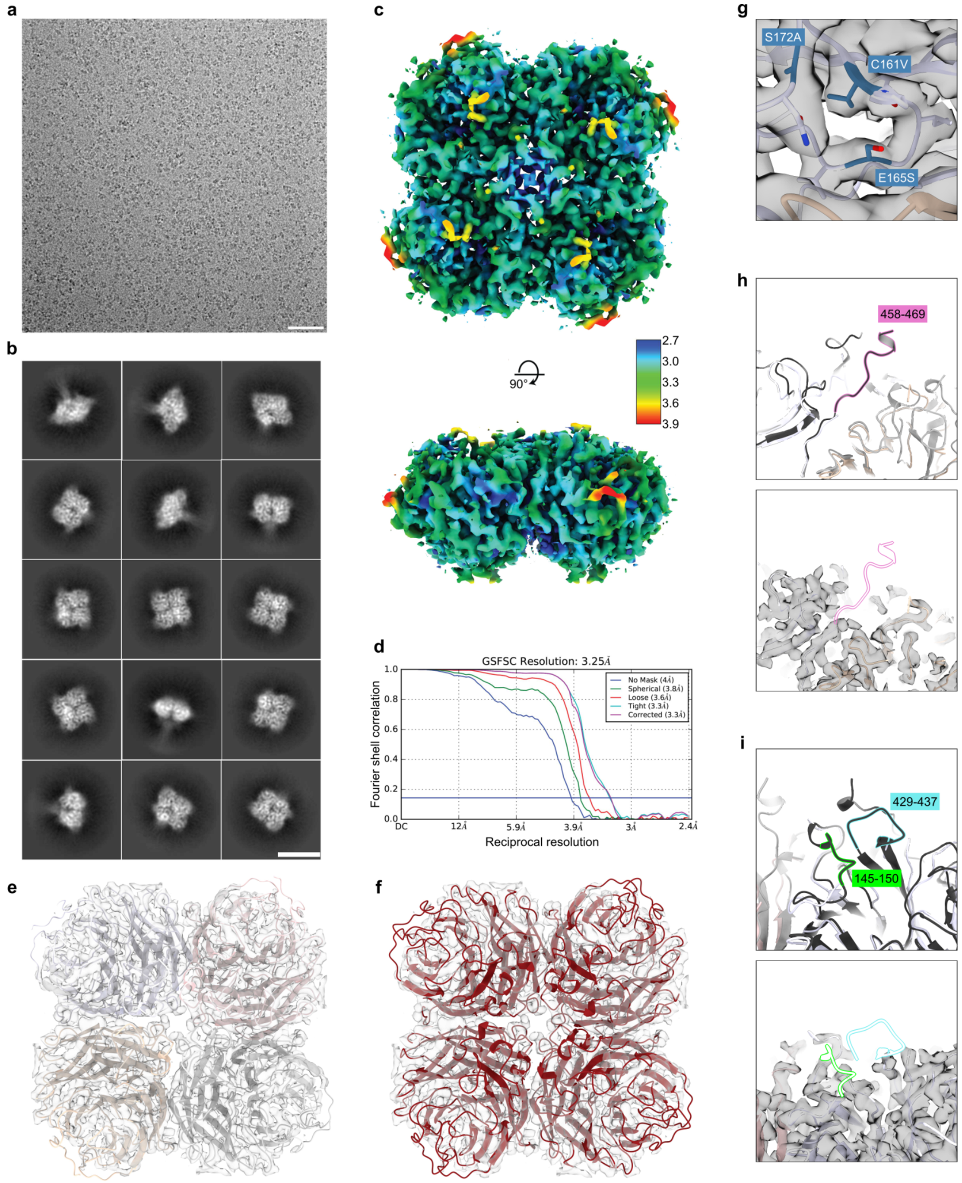

Supplementary Fig. 4. Cryo-EM analysis of N1-CA09-sNAp-155. **a** Representative micrograph of N1-CA09-sNAp-155. Scale bar, 50 nm. **b** 2D class averages of N1-CA09-sNAp-155. Scale bar, 10 nm. **c** Orthogonal views of the cryo-EM reconstruction of N1-CA09-sNAp-155. Map is colored according to local resolution as indicated by color scale (in Å). **d** Gold standard FSC for cryo-EM reconstruction. **e** N1-CA09-sNAp-155 model coloured by chain shown in transparent density showing overall tetramer conformation of map. Cryo-EM structure was determined once. **f** N1-CA09-WT (4B7Q, red) rigid-body docked into the N1-CA09-sNAp-155 map, showing overall agreement with tetramers observed by X-ray crystallography. **g** Density for the N1-CA09-sNAp-155 map overlaid on the generated model, with residues in space D shown. **h** Snapshots of the C-terminal region of the N1-CA09-sNAp-155 model (colored by chain in light blue and brown), either aligned with the crystal structure for N1-CA09-WT (top panel, 4B7Q, colored by chain in dark gray and light gray) or fit into the density used to generate the model (bottom panel). Both images are taken from the exact same angle. The region from residues 458-469 that is ordered in the N1-CA09-WT crystal structure but not in N1-CA09-sNAp-155 is highlighted in pink in the top panel. The same highlighted outline from N1-CA09-WT is shown in the lower panel. **i** Snapshots of the 150- and 430-loop regions of the N1-CA09-sNAp-155 model (colored

by chain in light blue and brown), either aligned with the crystal structure for N1-CA09-WT (top panel, 4B7Q, colored by chain in dark gray and light gray) or fit into the density used to generate the model (bottom panel). Both images are taken from the exact same angle. The regions from residues 145-150 and 429-437 that are ordered in the N1-CA09-WT crystal structure but not in N1-CA09-sNAp-155 are highlighted in green and blue in the top panel, respectively. The same highlighted outlines from N1-CA09-WT are shown in the lower panel.

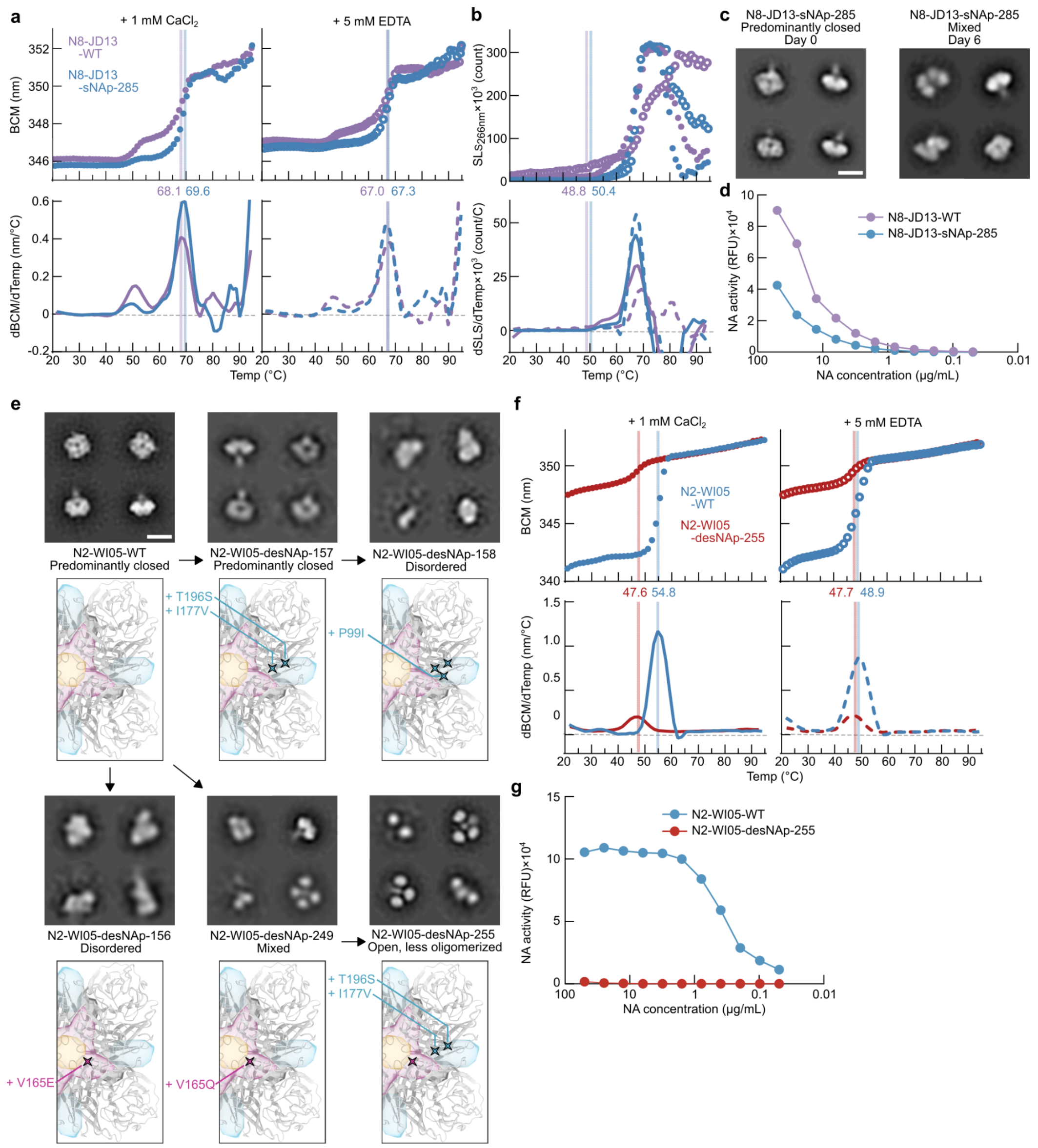

Supplementary Fig. 5. Structural, biophysical, and biochemical characterization of a stabilized N8 NA tetramer and destabilized N2 NA tetramers. **a** Thermal denaturation of N8-JD18-WT and N8-JD13-sNAp-285 in the presence of 1 mM $CaCl_2$ (closed circles and solid lines) or 5 mM EDTA (open circles and dashed lines), monitored by intrinsic tryptophan fluorescence. Data are presented as in Fig. 2d. **b** SLS during thermal denaturation of N8-JD18-WT and N8-JD13-sNAp-285 in the presence of 1 mM $CaCl_2$ or 5 mM EDTA. Data are presented as in Fig. 2e. **c** Shelf-life stability of N8-JD13-sNAp-285 over 6 days at 4°C as assessed by NS-EM (scale bar, 10 nm). **d** Enzymatic activity of N8-JD13-WT and N8-JD13-sNAp-285 determined with the NA-star assay. RFU, relative fluorescence units. Experiment was repeated at least twice, with a representative result shown. **e** Mutation of predicted stabilizing residues in N2 NA to less stable counterparts observed in N1 or N8 and corresponding NS-EM 2D class averages. All numbers are listed in N2 numbering in which positions 99, 165, 176 and 195 are equivalent to positions 99, 165, 177 and 196 in N1 numbering, respectively (scale bar, 10 nm). **f** Thermal denaturation of N2-WI05-WT and N2-WI05-desNAp-255 in the presence of 1 mM $CaCl_2$ or 5 mM EDTA, monitored by intrinsic tryptophan fluorescence. Data are presented as in Fig. 2d. **g** Enzymatic activity of N2-WI05-WT and N2-WI05-desNAp-255 determined with the NA-star assay. RFU, relative fluorescence units. Experiment was repeated at least twice, with a representative result shown.

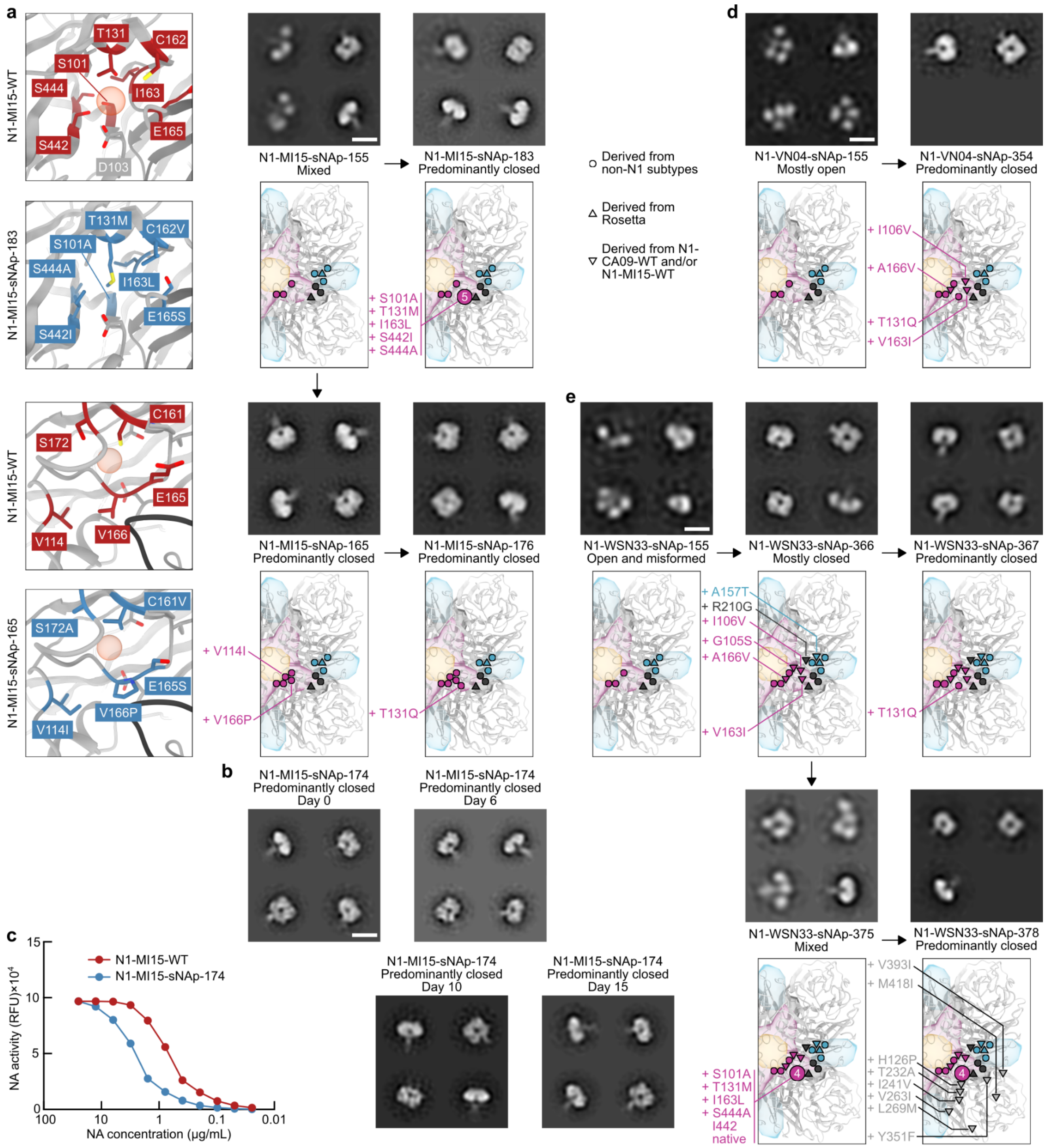

Supplementary Fig. 6. Design of additional closed recombinant N1 NA tetramers. **a** Design model snapshots (left) and NS-EM 2D class averages (right) of successfully designed sNAps for N1 MI15. The red sphere indicates the cavity within space D. Structural models were derived from a Rosetta-generated homology model of N1 MI15 NA based on PDB ID 47BQ (scale bar, 10nm). 2D class averages for N1-MI15-sNAp-155 are the same as presented in Fig. 5a. **b** Shelf-life stability of N1-MI15-sNAp-174 over 15 days at 4°C as assessed by NS-EM (scale bar, 10 nm). Experiment was performed once. **c** Representative enzymatic activity of N1-MI15-WT compared to N1-MI15-sNAp-174 determined with the NA-star assay. RFU, relative fluorescence units. Experiment was repeated twice. **d** Design of a predominantly closed N1 VN04 sNAp using sNAp-155 mutations in addition to T131Q and three other mutations in space D that are derived from N1 CA09 and N1 MI15 (scale bar, 10 nm). NS-EM was prepared and analyzed multiple times, with representative images shown. **e** Design of multiple N1 WSN33 sNAps using homology-directed mutations in combination with strain-specific mutations derived from N1

CA09 and N1 MI15. The mutations added to finalize N1-WSN33-sNAp-375 utilize the same mutation set featured in N1-MI15-sNAp-183. I442 was already native to N1 WSN33 (scale bar, 10 nm). NS-EM was prepared and analyzed multiple times, with representative images shown.

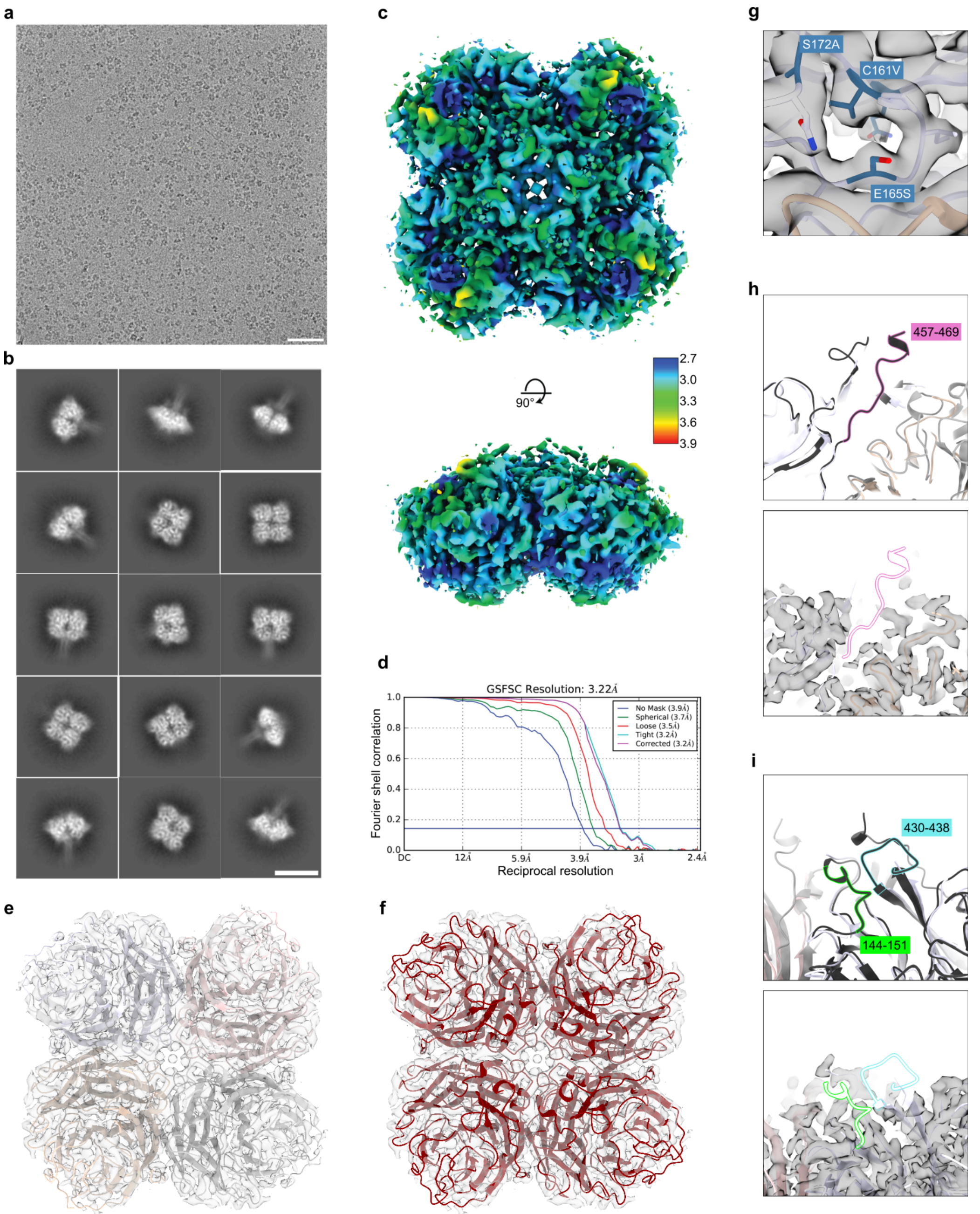

Supplementary Fig. 7. Cryo-EM analysis of N1-MI15-sNAp-174. **a** Representative micrograph of N1-MI15-sNAp-174. Scale bar, 50 nm. **b** 2D class averages of N1-MI15-sNAp-174. Scale bar, 10 nm. **c** Orthogonal views of the cryo-EM reconstruction of N1-CA09-sNAp-155. Map is colored according to local resolution as indicated by color scale (in Å). **d** Gold standard FSC for cryo-EM reconstruction. **e** N1-CA09-sNAp-174 model coloured by chain shown in transparent density showing overall tetramer conformation of map. Cryo-EM structure was determined once. **f** N1-CA09-WT (4B7Q, red) rigid-body docked into the N1-MI15-sNAp-174 map, showing overall agreement with tetramers observed by X-ray crystallography. **g** Density for the N1-MI15-sNAp-174 map overlaid on the generated model, with residues in space D shown. **h** Snapshots of the C-terminal region of the N1-MI15-sNAp-174 model (colored by chain in light blue and brown), either aligned with the crystal structure for N1-CA09-WT (top panel, 4B7Q, colored by chain in dark gray and light gray) or fit into the density used to generate the model (bottom panel). Both images are taken from the exact same angle. The

region from residues 457-469 that is ordered in the N1-CA09-WT crystal structure but not in N1-MI15-sNAp-174 is highlighted in pink in the top panel. The same highlighted outline from N1-CA09-WT is shown in the lower panel. **i** Snapshots of the 150- and 430-loop regions of the N1-MI15-sNAp-174 model (colored by chain in light blue and brown), either aligned with the crystal structure for N1-CA09-WT (top panel, 4B7Q, colored by chain in dark gray and light gray) or fit into the density used to generate the model (bottom panel). Both images are taken from the exact same angle. The regions from residues 145-150 and 429-437 that are ordered in the N1-CA09-WT crystal structure but not in N1-MI15-sNAp-174 are highlighted in green and blue in the top panel, respectively. The same highlighted outlines from N1-CA09-WT are shown in the lower panel.

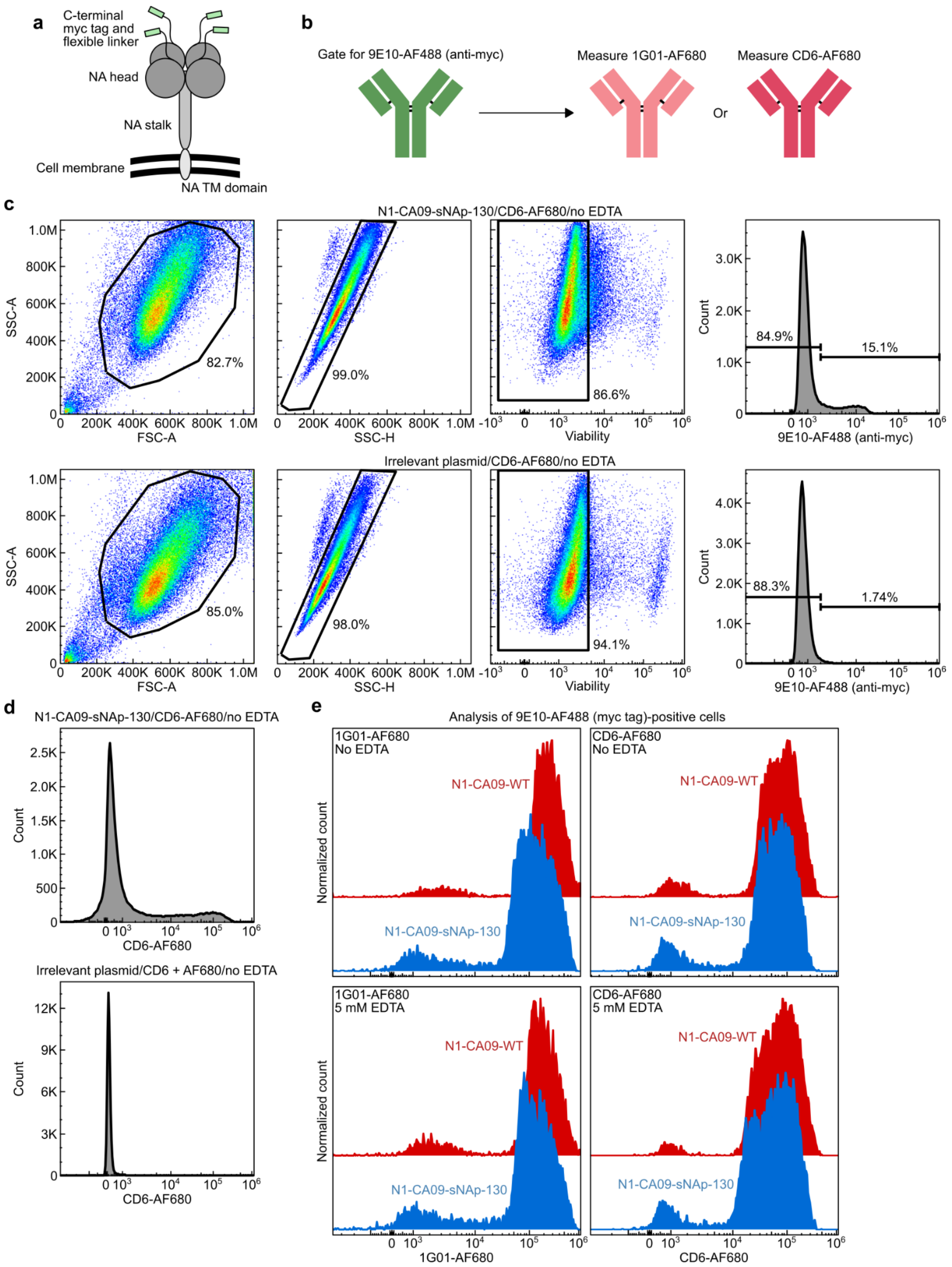

Supplementary Fig. 8. Flow cytometry analysis of Expi293F cells displaying membrane-anchored N1-CA09-WT and N1-CA09-sNAp-130 and their binding to fluorescently-labeled antibodies. **a** Cartoon representation of NA constructs, in which the full sequence of N1 CA09 was used for both WT and stabilized designs. A flexible linker

and myc tag were added to the exposed C-terminal end of the NA head domain to allow for general recognition of expression regardless of foldedness. **b** Scheme for antigenic analysis of cells displaying membrane-anchored NAs by fluorescently-labeled antibodies. Cells were gated for general expression of NA on the cell surface based on anti-myc (9E10) labeled with Alexa Fluor 488, then binding to 1G01 or CD6 labeled with Alexa Fluor 680 (1G01-AF680 and CD6-AF680, respectively). **c** Representative examples of gating strategies used for identifying viable cells displaying myc tags. The top row shows an example of the gating strategy performed on cells transfected with plasmids expressing NA, while the bottom row shows an example of the gating strategy performed on cells transfected with an irrelevant plasmid (solubly-expressed hemagglutinin genetically fused to T4 fibritin foldon). Viability was measured using Zombie Violet. **d** Examples of viable cells measured for CD6-AF680 signal without prior gating for positive 9E10-AF488 signal, using the same samples analyzed in panel c. **e** 1G01-AF680 and CD6-AF680 signals for cells gated on 9810-AF488 signal. Data was collected for N1 CA09 containing either the WT sequence (N1-CA09-WT) or stabilizing mutations from N1-CA09-sNAp-130. All measurements were performed in technical and biological duplicate both in the presence or absence of 5 mM EDTA, with representative samples shown.

Supplementary Table 1. Residues considered in spaces A, B, C and D, as well as other mutated residues which were not included in the four spaces ("Other"). Conversions between N1 and N2 numbering are also shown. All residues in this manuscript are listed in N1 numbering unless otherwise noted.

	**N1 numbering**	**N2 numbering**
**Space A**	99<br>157<br>177<br>196<br>198<br>201<br>203<br>205<br>215<br>217<br>451<br>453	99<br>157<br>176<br>195<br>197<br>200<br>202<br>204<br>214<br>216<br>452<br>454
**Space B**	100<br>207<br>210<br>211<br>212<br>408<br>412<br>419	100<br>206<br>209<br>210<br>211<br>412<br>414b<br>419
**Space C**	113<br>168<br>169<br>170<br>171	113<br>168<br>169<br>169a<br>170
**Space D**	101<br>103<br>105<br>106<br>114<br>122<br>131<br>161<br>162<br>163<br>164<br>165<br>166<br>172<br>442<br>444	101<br>103<br>105<br>106<br>114<br>122<br>131<br>161<br>162<br>163<br>164<br>165<br>166<br>172<br>443<br>445
**Other**	126<br>232<br>241	126<br>231<br>240

	263<br>269<br>351<br>393<br>418	260<br>268<br>354<br>397<br>418

Supplementary Table 2. Summary of conformational and yield data for all stabilized or destabilized constructs and WT counterparts, along with mutations included. All mutations are listed in N1 numbering. For yields, all values were recorded after IMAC purification: * <1 mg/L, ** 1-2 mg/L, *** 2-5 mg/L, **** 5-10 mg/L, ***** 10-30 mg/L.

Construct	Conformational state	Yield	Stabilizing mutations identified from outside of N1	Stabilizing mutations identified from N1-CA09 and/or N1-MI15	Destabilizing mutations	Stabilizing mutations identified by Rosetta
N1-CA09-WT	Predominantly open	***	None	None	None	None
N1-CA09-WT + Stalk	Open and disordered	**	None	None	None	None
N1-CA09-sNAp-94	Mixed	**	I99P (N2/N3/N4), Y100L (N4), D113E (N4), E165S (N5), Y170S (N4), S196T (N2/N3/N4), R419V (N4/N5), V453T (N4)	None	None	V205I, Q408M
N1-CA09-sNAp-114	Predominantly closed	*	I99P (N2/N3/N4), Y100L (N4), D113E (N4), E165S (N5), Y170S (N4), V177I (N2/N3), S196T (N2/N3/N4), R419V (N4/N5), V453T (N4)	None	None	V205I, Q408M
N1-CA09-sNAp-130	Predominantly closed	**	I99P (N2/N3/N4), Y100L (N4), C161V (N5), E165S (N5), S172A (N5), V177I (N2/N3), S196T (N2/N3/N4), R419V (N4/N5), V453T (N4)	None	None	V205I, Q408M
N1-CA09-sNAp-155	Predominantly closed	***	I99P (N2/N3/N4), Y100L (N4), C161V (N5), E165S (N5), S172A (N5), V177I (N2/N3), S196T (N2/N3/N4), R419V (N4/N5)	None	None	V205I, Q408M
N1-CA09-sNAp-130 + Stalk	Predominantly closed	**	I99P (N2/N3/N4), Y100L (N4), C161V (N5), E165S (N5), S172A (N5), V177I (N2/N3), S196T (N2/N3/N4), R419V (N4/N5), V453T (N4)	None	None	V205I, Q408M

N1-CA09-sNAp-131	Predominantly closed	*	I99P (N2/N3/N4), C161V (N5), E165S (N5), S172A (N5), V177I (N2/N3), S196T (N2/N3/N4), V453T (N4)	None	None	V205I
N1-CA09-sNAp-134	Mixed	***	I99P (N2/N3/N4), Y100L (N4), C161V (N5), S172A (N5), V177I (N2/N3), S196T (N2/N3/N4), R419V (N4/N5)	None	None	V205I, Q408M
N8-JD13-WT	Mixed	****	None	None	None	None
N8-JD13-sNAp-282	Mixed	****	Q165S (N5)	None	None	None
N8-JD13-sNAp-285	Predominantly closed	*****	E162P (N1/N3/N4/N5), Q165S (N5)	None	None	None
N2-WI05-WT	Predominantly closed	****	None	None	None	None
N2-WI05-desNAp-156	Disordered	***	None	None	V165E (N1)	None
N2-WI05-desNAp-157	Predominantly closed	*****	None	None	I177V (N1), T196S (N1)	None
N2-WI05-desNAp-158	Disordered	***	None	None	P99I (N1), I177V (N1), T196S (N1)	None
N2-WI05-desNAp-249	Mixed	***	V165Q (N8)	None	V165Q (N8)	None
N2-WI05-desNAp-255	Open, less oligomerized	***	V165Q (N8)	None	V165Q (N8), I177V (N1), T196S (N1)	None
N1-MI15-WT	Predominantly open	***	None	None	None	None
N1-MI15-sNAp-155	Mixed	*****	I99P (N2/N3/N4), Y100L (N4), C161V (N5), E165S (N5), S172A (N5), V177I (N2/N3), S196T (N2/N3/N4), R419V (N4/N5)	None	None	V205I, Q408M
N1-MI15-sNAp-174	Predominantly closed	*****	I99P (N2/N3/N4), Y100L (N4), T131Q (N2), C161V (N5), E165S (N5), S172A (N5), V177I (N2/N3), S196T (N2/N3/N4), R419V (N4/N5)	None	None	V205I, Q408M

N1-MI15-sNAp-165	Predominantly closed	***	I99P (N2/N3/N4), Y100L (N4), V114I (N2), C161V (N5), E165S (N5), V166P (N2), S172A (N5), V177I (N2/N3), S196T (N2/N3/N4), R419V (N4/N5)	None	None	V205I, Q408M
N1-MI15-sNAp-176	Predominantly closed	***	I99P (N2/N3/N4), Y100L (N4), V114I (N2), T131Q (N2), C161V (N5), E165S (N5), V166P (N2), S172A (N5), V177I (N2/N3), S196T (N2/N3/N4), R419V (N4/N5)	None	None	V205I, Q408M
N1-MI15-sNAp-183	Predominantly closed	****	I99P (N2/N3/N4), Y100L (N4), S101A (N7), T131M (N9), C161V (N5), I163L (N2/N5/N7/N9), E165S (N5), S172A (N5), V177I (N2/N3), S196T (N2/N3/N4), R419V (N4/N5), S442I (N2), S444A (N4/N6/N7)	None	None	V205I, Q408M
N1-VN04-WT	Disordered	***	None	None	None	None
N1-VN04-sNAp-155	Mostly open	*****	I99P (N2/N3/N4), Y100L (N4), C161V (N5), E165S (N5), S172A (N5), V177I (N2/N3), S196T (N2/N3/N4), R419V (N4/N5)	None	None	V205I, Q408M
N1-VN04-sNAp-354	Predominantly closed	*****	I99P (N2/N3/N4), Y100L (N4), T131Q (N2), C161V (N5), E165S (N5), S172A (N5), V177I (N2/N3), S196T (N2/N3/N4), R419V (N4/N5)	I106V, V163I, A166V	None	V205I, Q408M
N1-WSN33-WT	Open and misformed	***	None	None	None	None
N1-WSN33-sNAp-155	Open and misformed	**	I99P (N2/N3/N4), Y100L (N4), C161V (N5), E165S (N5), S172A (N5), V177I (N2/N3), S196T (N2/N3/N4), R419V (N4/N5)	None	None	V205I, Q408M
N1-WSN33-sNAp-366	Mostly closed	****	I99P (N2/N3/N4), Y100L (N4), C161V (N5), E165S (N5), S172A (N5), V177I (N2/N3), S196T (N2/N3/N4), R419V (N4/N5)	G105S, I106V, A157T, V163I, A166V, R210G	None	V205I, Q408M

N1-WSN33-sNAp-367	Predominantly closed	****	I99P (N2/N3/N4), Y100L (N4), T131Q (N2), C161V (N5), E165S (N5), S172A (N5), V177I (N2/N3), S196T (N2/N3/N4), R419V (N4/N5)	G105S, I106V, A157T, V163I, A166V, R210G	None	V205I, Q408M
N1-WSN33-sNAp-375	Mixed	****	I99P (N2/N3/N4), Y100L (N4), S101A (N7), T131M (N9), C161V (N5), V163L (N2/N5/N7/N9), E165S (N5), S172A (N5), V177I (N2/N3), S196T (N2/N3/N4), R419V (N4/N5), S444A (N4/N6/N7)	G105S, I106V, A157T, A166V, R210G	None	V205I, Q408M
N1-WSN33-sNAp-378	Predominantly closed	****	I99P (N2/N3/N4), Y100L (N4), S101A (N7), T131M (N9), C161V (N5), V163L (N2/N5/N7/N9), E165S (N5), S172A (N5), V177I (N2/N3), S196T (N2/N3/N4), R419V (N4/N5), S444A (N4/N6/N7)	G105S, I106V, H126P, A157T, A166V, R210G, T232A, I241V, V263I, L269M, Y351F, V393I, M418I	None	V205I, Q408M

Supplementary Table 3. Cryo-EM data collection, refinement and validation statistics.

	**N1-CA09-sNAp-155 (EMD-26318) (PDB 7U2Q)**	**N1-MI15-sNAp-174 (EMD-26319) (PDB 7U2T)**
**Data collection and processing**		
Magnification	36,000x	36,000x
Voltage (kV)	200	200
Electron exposure (e–/Å^2)	60	60
Defocus range (μm)	-0.8 to -2.5	-0.8 to -2.5
Pixel size (Å)	1.16	1.16
Symmetry imposed	C4	C4
Initial particle images (no.)	496,303	177,620
Final particle images (no.)	67,444	72,106
Map resolution (Å)	3.2	3.2
FSC threshold	0.143	0.143
Map resolution range (Å)	2.7-3.9	2.7-3.6
**Refinement**		
Initial model used (PDB code)	N1-MI15-sNAp-174 model (with point mutations)	4B7Q (with point mutations)
Model resolution (Å)	3.4	3.4
FSC threshold	0.5	0.5
Map sharpening *B* factor (Å^2)	-143	-150
Model composition		
Non-hydrogen atoms	10536	10300
Protein residues	1352	1336
Ligands	8 (NAG)	8 (NAG)
B factors (Å^2)		
Protein	35.37	34.03
Ligand	20.00	50.00
R.m.s. deviations		
Bond lengths (Å)	0.013	0.013
Bond angles (°)	1.413	1.411
Validation		
MolProbity score	1.71	1.29
Clashscore	1.51	1.44
Poor rotamers (%)	4.83	1.77
Ramachandran plot		
Favored (%)	95.73	96.62
Allowed (%)	3.96	3.11
Disallowed (%)	0.30	0.31

Supplementary Table 4. Mutations to Rescued H1N1 CA09 Viruses with and without N1-CA09-sNAp-155 Mutations.

	**CA09 with WT NA**	**CA09 with sNAp-155 NA**
**HA substitutions***	V41I, G148S	K171N
**NA substitution***	None	T466A

*Non-synonymous mutations in HA and NA genes of R3ΔPB1 A/California/07/2009 H1N1 virus and R3ΔPB1 A/California/07/2009 H1N1 sNAp-155 virus on MDCK-SIAT1 PB1 cells. Both HA and NA numbering start from the methionine at the beginning of each open reading frame.
