# Supplementary material for: Structure-based design of stabilized recombinant influenza neuraminidase tetramers
Source: Nat Commun. 2022 Apr 5;13:1825. doi: 10.1038/s41467-022-29416-z (PMC8983682; doi:10.1038/s41467-022-29416-z)
Supplement: Supplementary file 2 — Description of Additional Supplementary Files [file 41467_2022_29416_MOESM2_ESM.pdf]

File Name: Supplementary Data 1
Description: Amino acid sequences of NA constructs used in the study

File Name: Supplementary Data 2
Description: Representative raw negative-stain EM images and 2D class averages of NA constructs used in the study

File Name: Supplementary Data 3
Description: Rosetta design scripts and their instructions used in the study
