# Supplementary material for: Structure-based design of stabilized recombinant influenza neuraminidase tetramers
Source: Nat Commun. 2022 Apr 5;13:1825. doi: 10.1038/s41467-022-29416-z (PMC8983682; doi:10.1038/s41467-022-29416-z)
Supplement: Supplementary file 3 — Supplementary Data 1 [file 41467_2022_29416_MOESM3_ESM.pdf]

All soluble NA head sequences shown below were preceded with either of the following sequences containing secretion signal, affinity tags, hVASP tetramerization domain, thrombin cleavage site and GG linker:

<u>Only Hexa-His tag</u>

MEFGLSWIFLAAILKGVQCADPHHHHHHSSSDYSDLQRVKQELLEEVKKELQKVKEEIIEAFVQ
ELRKRGSLVPRGSGG

<u>Both Hexa-His tag and Strep tag</u>

MEFGLSWIFLAAILKGVQCADPHHHHHHGSAWSHPQFEKGGSSSDYSDLQRVKQELLEEVKKEL
QKVKEEIIEAFVQELRKRGSLVPRGSGG

<u>N1-CA09-WT</u>

H1N1 A/California/07/2009

VKLAGNSSLCPVSGWAIYSKDNSVRIGSKGDVFVIREPFISCSPLECRTFFLTQGALLNDKHSN
GTIKDRSPYRTLMSCPIGEVPSPYNSRFESVAWSASACHDGINWLTIGISGPDNGAVAVLKYNG
IITDTIKSWRNNILRTQESECACVNGSCFTVMTDGPSNGQASYKIFRIEKGKIVKSVEMNAPNY
HYEECSCYPDSSEITCVCRDNWHGSNRPWVSFNQNLEYQIGYICSGIFGDNPRPNDKTGSCGPV
SSNGANGVKGFSYKYGNGVWIGRTKSISSRNGFEMIWDPNGWTGTDNNFSIKQDIVGINEWSGY
SGSFVQHPELTGLDCIRPCFWVELIRGRPKENTIWTSGSSISFCGVNSDTVGWSWPDGAELPFT
IDK

<u>N1-MI15-WT</u>

H1N1 A/Michigan/45/2015

VKLAGNSSLCPVSGWAIYSKDNSVRIGSKGDVFVIREPFISCSPLECRTFFLTQGALLNDKHSN
GTIKDRSPYRTLMSCPIGEVPSPYNSRFESVAWSASACHDGINWLTIGISGPDSGAVAVLKYNG
IITDTIKSWRNNILRTQESECACVNGSCFTIMTDGPSDGQASYKIFRIEKGKIIKSVEMKAPNY
HYEECSCYPDSSEITCVCRDNWHGSNRPWVSFNQNLEYQMGYICSGVFGDNPRPNDKTGSCGPV
SSNGANGVKGFSFKYGNGVWIGRTKSISSRKGFEMIWDPNGWTGTDNKFSIKQDIVGINEWSGY
SGSFVQHPELTGLDCIRPCFWVELIRGRPEENTIWTSGSSISFCGVNSDTVGWSWPDGAELPFT
IDK

<u>N1-NC99-WT</u>

H1N1 A/New_Caledonia/20/1999

VTLAGNSSLCSISGWAIYTKDNSIRIGSKGDVFVIREPFISCSHLECRTFFLTQGALLNDKHSN
GTVKDRSPYRALMSCPLGEAPSPYNSKFESVAWSASACHDGMGWLTIGISGPDNGAVAVLKYNG
IITETIKSWKKRILRTQESECVCVNGSCFTIMTDGPSNGAASYKIFKIEKGKVTKSIELNAPNF
HYEECSCYPDTGTVMCVCRDNWHGSNRPWVSFNQNLDYQIGYICSGVFGDNPRPKDGEGSCNPV
TVDGADGVKGFSYKYGNGVWIGRTKSNRLRKGFEMIWDPNGWTDTDSDFSVKQDVVAITDWSGY
SGSFVQHPELTGLDCIRPCFWVELVRGLPRENTTIWTSGSSISFCGVNSDTANWSWPDGAELPF
TIDK

<u>N1-WSN33-WT</u>

H1N1 A/WSN/1933

VILTGNSSLCPIRGWAIHSKDNGIRIGSKGDVFVIREPFISCSHLECRTFFLTQGALLNDKHSR
GTFKDRSPYRALMSCPVGEAPSPYNSRFESVAWSASACHDGMGWLTIGISGPDDGAVAVLKYNR
IITETIKSWRKNILRTQESECTCVNGSCFTIMTDGPSDGLASYKIFKIEKGKVTKSIELNAPNS

HYEECSCYPDTGKVMCVCRDNWHGSNRPWVSFDQNLDYKIGYICSGVFGDNPRPKDGTGSCGPV
SADGANGVKGFSYKYGNGVWIGRTKSDSSRHGFEMIWDPNGWTETDSRFSMRQDVVAITNRSGY
SGSFVQHPELTGLDCMRPCFWVELIRGLPEEDAIWTSGSIISFCGVNSDTVDWSWPDGAELPFT
IDK

N1-BV18-WT
H1N1 A/Brevig_Mission/1/1918
VILTGNSSLCPISGWAIYSKDNGIRIGSKGDVFVIREPFISCSHLECRTFFLTQGALLNDKHSN
GTVKDRSPYRTLMSCPVGEAPSPYNSRFESVAWSASACHDGMGWLTIGISGPDNGAVAVLKYNG
IITDTIKSWRNNILRTQESECACVNGSCFTIMTDGPSNGQASYKILKIEKGKVTKSIELNAPNY
HYEECSCYPDTGKVMCVCRDNWHGSNRPWVSFDQNLDYQIGYICSGVFGDNPRPNDGTGSCGPV
SSNGANGIKGFSFRYDNGVWIGRTKSTSSRSGFEMIWDPNGWTETDSSFSVRQDIVAITDWSGY
SGSFVQHPELTGLDCMRPCFWVELIRGQPKENTIWTSGSSISFCGVNSDTVGWSWPDGAELPFS
IDK

N1-VN04-WT
H5N1 A/Vietnam/1203/2004
VKLAGNSSLCPINGWAVYSKDNSIRIGSKGDVFVIREPFISCSHLECRTFFLTQGALLNDKHSN
GTVKDRSPHRTLMSCPVGEAPSPYNSRFESVAWSASACHDGTSWLTIGISGPDNGAVAVLKYNG
IITDTIKSWRNNILRTQESECACVNGSCFTVMTDGPSNGQASYKIFKMEKGKVVKSVELDAPNY
HYEECSCYPNAGEITCVCRDNWHGSNRPWVSFNQNLEYQIGYICSGVFGDNPRPNDGTGSCGPV
SSNGAYGVKGFSFKYGNGVWIGRTKSTNSRSGFEMIWDPNGWTETDSSFSVKQDIVAITDWSGY
SGSFVQHPELTGLDCIRPCFWVELIRGRPKESTIWTSGSSISFCGVNSDTVGWSWPDGAELPFT
IDK

N6-SI14-WT
H5N6 A/Sichuan/26221/2014
HLLNLTKPLCEVNSWHILSKDNAIRIGEDAHIIVTREPYLSCDPQGCRMFALSQGTTLRGKHAN
GTIHDRSPFRALVSWEMGQAPSPYNTRVECIGWSSTSCHDGISRMSICISGPNNNASAVVWYGG
RPVTEIPSWAGNILRTQESECVCHGGICPVVMTDGPANNRAETKIIYFKEGKIKKIEELKGDAQ
HIEECSCYGASEMIKCICRDNWKGANRPVITIDPEMMTHTSKYLCSKILTDTSRPNDPTNGKCE
APITGGSPDPGVKGFAFLDGENSWLGRTISKDSRSGYEMLKVPNAETDTQSGAISHQIIVNNQN
WSGYSGAFIDYWANKECFNPCFYVELIRGRPKESSVLWTSNSIVALCGSKERLGSWSWHDGAEI
IYFK

N2-MO99-WT
H3N2 A/Moscow/10/1999
EYRNWSKPQCNITGFAPFSKDNSIRLSAGGDIWVTREPYVSCDPDKCYQFALGQGTTLNNGHSN
DTVHDRTPYRTLLMNELGVPFHLGTKQVCIAWSSSSCHDGKAWLHVCVTGDDENATASFIYNGR
LVDSIGSWSKKILRTQESECVCINGTCTVVMTDGSASGKADTKILFIEEGKIVHTSPLSGSAQH
VEECSCYPRYPGVRCVCRDNWKGSNRPIVDINVKDYSIVSSYVCSGLVGDTPRKNDSSSSSHCL
DPNNEEGGHGVKGWAFDDGNDVWMGRTISEKLRSGYETFKVIEGWSKPNSKLQINRQVIVDRGN
RSGYSGIFSVEGKSCINRCFYVELIRGRKQETEVLWTSNSIVVFCGTSGTYGTGSWPDGADINL
MPI

N4-DB16-WT
H10N4 A/Red_knot/Delaware_Bay/310/2016
VHYSSGRDLCPIRGWAPLSKDNGIRIGSRGEVFVIREPFISCSISECRTFFLTQGALLNDKHSN
GTVKDRSPFRTLMSCPIGVAPSPSNSRFESVAWSATACSDGPGWLTLGITGPDSTAVAVLKYNG
IITDTLKSWKGNIMRTQESECVCQDEFCYTLVTDGPSDAQAFYKILKIRKGKIVSMKDVDATGF
HFEECSCYPSGTEIECVCRDNWRGSNRPWIRFNSDLDYQIGYVCSGIFGDNPRPVDGTGSCNGP
VNNGKGRYGVKGFSFRYGDGVWIGRTKSLESRSGFEMVWDANGWVSTDKDSNGVQDIIDNDNWS
GYSGSFSIRGETTGKNCTVPCFWVEMIRGQPKEKTIWTSGSSIAFCGVNSDTTGWSWPDGALLP
FDIDK

N7-NE03-WT
H7N7 A/Netherlands/219/03
SYLLLNKSLCNVEGWVVIAKDNAVRFGESEQIIVTREPYVSCDPTGCKMYALHQGTTIRNKHSN
GTIHDRTAFRGLISTPLGTPPTVSNSDFMCVGWSSTTCHDGIARMTICIQGNNDNATATVYYNR
RLTTTIKTWARNILRTQESECVCHNGTCAVVMTDGSASSQAYTKVMYFHKGLVVKEEELRGSAR
HIEECSCYGHNQKVTCVCRDNWQGANRPIIEIDMSTLEHTSRYVCTGILTDTSRPGDKSSGDCS
NPITGSPGVPGVKGFGFLNGDNTWLGRTISPRSRSGFEMLKIPNAGTDPNSRIAERQEIVDNNN
WSGYSGSFIDYWNDNSECYNPCFYVELIRGRPEEAKYVWWASNSLIALCGSPFPVGSGSFPDGA
QIQYFS

N8-JD13-WT
H10N8 A/Jiangxi-Donghu/346-2/2013
HFMNNTEALCDAKGFAPFSKDNGIRIGSRGHVFVIREPFVSCSPTECRTFFLTQGSLLNDKHSN
GTVKDRSPYRTLMSVEIGQSPNVYQARFEAVAWSATACHDGKKWMTIGVTGPDAKAVAVVHYGG
IPTDVINSWAGDILRTQESSCTCIQGECFWVMTDGPANRQAQYRAFKAKQGKIVGQAEISFNGG
HIEECSCYPNEGKVECVCKDNWTGTNRPVLVISPDLSYRVGYLCAGLPSDTPRGEDSQFTGSCT
SPMGNQGYGVKGFGFRQGNDVWMGRTISRTSRSGFEILKVRNGWVQNSKEQIKRQVVVDNLNWS
GYSGSFTLPAELTKRNCLVPCFWVEMIRGNPEEKTIWTSSSSIVMCGVDHEIADWSWHDGAILP
FDIDKM

N9-AN13-WT
H7N9 A/Anhui/1/2013
NFNNLTKGLCTINSWHIYGKDNAVRIGESSDVLVTREPYVSCDPDECRFYALSQGTTIRGKHSN
GTIHDRSQYRALISWPLSSPPTVYNSRVECIGWSSTSCHDGKSRMSICISGPNNNASAVVWYNR
RPVAEINTWARNILRTQESECVCHNGVCPVVFTDGSATGPADTRIYYFKEGKILKWESLTGTAK
HIEECSCYGERTGITCTCRDNWQGSNRPVIQIDPVAMTHTSQYICSPVLTDNPRPNDPNIGKCN
DPYPGNNNNGVKGFSYLDGANTWLGRTISTASRSGYEMLKVPNALTDDRSKPIQGQTIVLNADW
SGYSGSFMDYWAEGDCYRACFYVELIRGRPKEDKVWWTSNSIVSMCSSTEFLGQWNWPDGAKIE
YFL

N2-WI05-WT
H3N2 A/Wisconsin/67/2005
EYRNWSKPQCNITGFAPFSKDNSIRLSAGGDIWVTREPYVSCDPDKCYQFALGQGTTLNNVHSN
DTVHDRTPYRTLLMNELGVPFHLGTKQVCIAWSSSSCHDGKAWLHVCVTGDDKNATASFIYNGR
LVDSIVSWSKEILRTQESECVCINGTCTVVMTDGSASGKADTKILFIEEGKIVHTSTLSGSAQH

VEECSCYPRYLGVRCVCRDNWKGSNRPIVDINIKDYSIVSSYVCSGLVGDTPRKNDSSSSSHCL
DPNNEEGGHGVKGWAFDDGNDVWMGRTISEKLRSGYETFKVIEGWSNPNSKLQINRQVIVDRGN
RSGYSGIFSVEGKSCINRCFYVELIRGRKEETEVLWTSNSIVVFCGTSGTYGTGSWPDGADINL
MPI

N2-IN11-WT

H3N2 A/Indiana/10/2011

EYRNWSKPQCNITGFAPFSKDNSIRLSAGGDIWVTREPYVSCDPDKCYQFALGQGTTLNNGHSN
NTVHDRTPYRTLLMNELGVPFHLGTRQVCMAWSSSSCHDGKAWLHVCITGNDNNATASFIYNGR
LVDSIGSWSKNILRTQESECVCINGTCTVVMTDGSASGKADTKILFVEEGKIVHISTLSGSAQH
VEECSCYPRFPGVRCVCRDNWKGSNRPIVDINVKNYSIVSSYVCSGLVGDTPRKSDSVSSSYCL
DPNNEKGGHGVKGWAFDDGNDVWMGRTINETLRLGYETFKVIEGWSKANSKLQTNRQVIVEKGD
RSGYSGIFSVEGKSCINRCFYVELIRGRKEETKVWWTSNSIVVFCGTSGTYGTGSWPDGADINL
MPI

N3-MI06-WT

H2N3 A/Swine/Missouri/2124514/2006

EEERPFKSPLPLCPFRGFFPFHKDNAIRLGENKDVIVTREPYVSCDNDNCWSFALAQGALLGTK
HSNGTIKDRTPYRSLIRFPIGTAPVLGNYKEICIAWSSSSCFDGKEWMHVCMTGNDNDASAQII
YGGRMTDSIKSWRKDILRTQESECQCIDGTCVVAVTDGPAANSADYRVYWIREGKIIKYENVPK
TKIQHLEECSCYVDIDVYCICRDNWKGSNRPWMRINNETILETGYVCSKFHSDTPRPADPSTMS
CDSPSNVNGGPGVKGFGFKAGDDVWLGRTVSTSGRSGFEIIKVTEGWINSPNHVKSITQTLVSN
NDWSGYSGSFIVKAKDCFQPCFYVELIRGRPNKNDDVSWTSNSIVTFCGLDNEPGSGNWPDGSN
IGFMPK

N5-DB16-WT

H10N5 A/Shorebird/Delaware_Bay/309/2016

EFLNNTEPLCNVSGFAIVSKDNGIRIGSRGHVFVIREPFVACGPTECRTFFLTQGALLNDKHSN
NTVKDRSPYRALMSVPLGSSPNAYQAKFESVAWSATACHDGKRWLAVGISGADDDAYAVIHYGG
MPTDVVRSWRKQILRTQESSCVCMKGNCYWVMTDGPANSQASYKIFKSHKGMVTNEREVSFQGG
HIEECSCYPNLGKVECVCRDNWNGMNRPVLTFDEDLNYEVGYLCAGIPTDTPRVQDNSFIGSCT
NAVGGSGTNNYGVKGFGFRQGNSVWAGRTVSISSRSGFEILLVEDGWVKTSKNVVKKVEVLNNK
NWSGYSGAFTIPITMTSKQCLVPCFWLEMIRGKPEERTSIWTSSSSTVFCGVSSEVPGWSWDDG
AILPFDIDKM

B-CO17-WT

B-Victoria B/Colorado/06/2017

PEPEWTYPRLSCPGSTFQKALLISPHRFGETKGNSAPLIIREPFVACGPNECKHFALTHYAAQP
GGYYNGTRGDRNKLRHLISVKLGKIPTVENSIFHMAAWSGSACHDGKEWTYIGVDGPDNNALLK
VKYGEAYTDTYHSYANNILRTQESACNCIGGNCYLMITDGSASGVSECRFLKIREGRIIKEIFP
TGRVKHTEECTCGFASNKTIECACRDNRYTAKRPFVKLNVETDTAEIRLMCTDTYLDTPRPNDG
SITGPCESDGDKGSGGIKGGFVHQRMKSKIGRWYSRTMSQTERMGMGLYVKYGGDPWADSDALA
FSGVMVSMKEPGWYSFGFEIKDKKCDVPCIGIEMVHDGGKETWHSAATAIYCLMGSGQLLWDTV
TGVDMAL

B-PH13-WT
B-Yamagata B/Phuket/3073/2013
PEPEWTYPRLSCPGSTFQKALLISPHRFGETKGNSAPLIIREPFIACGPKECKHFALTHYAAQP
GGYYNGTREDRNKLRHLISVKLGKIPTVENSIFHMAAWSGSACHDGREWTYIGVDGPDSNALLK
IKYGEAYTDTYHSYAKNILRTQESACNCIGGDCYLMITDGPASGISECRFLKIREGRIIKEIFP
TGRVKHTEECTCGFASNKTIECACRDNSYTAKRPFVKLNVETDTAEIRLMCTKTYLDTPRPNDG
SITGPCESDGDEGSGGIKGGFVHQRMASKIGRWYSRTMSKTKRMGMGLYVKYDGDPWTDSEALA
LSGVMVSMEEPGWYSFGFEIKDKKCDVPCIGIEMVHDGGKTTWHSAATAIYCLMGSGQLLWDTV
TGVNMTL

N9-AN13-Y170H
H7N9 A/Anhui/1/2013
NFNNLTKGLCTINSWHIYGKDNAVRIGESSDVLVTREPYVSCDPDECRFYALSQGTTIRGKHSN
GTIHDRSQYRALISWPLSSPPTVHNSRVECIGWSSTSCHDGKSRMSICISGPNNNASAVVWYNR
RPVAEINTWARNILRTQESECVCHNGVCPVVFTDGSATGPADTRIYYFKEGKILKWESLTGTAK
HIEECSCYGERTGITCTCRDNWQGSNRPVIQIDPVAMTHTSQYICSPVLTDNPRPNDPNIGKCN
DPYPGNNNNGVKGFSYLDGANTWLGRTISTASRSGYEMLKVPNALTDDRSKPIQGQTIVLNADW
SGYSGSFMDYWAEGDCYRACFYVELIRGRPKEDKVWWTSNSIVSMCSSTEFLGQWNWPDGAKIE
YFL

N1-CA09-WT + Stalk
H1N1 A/California/07/2009
SHSIQLGNQNQIETCNQSVITYENNTWVNQTYVNISNTNFAAGQSVVSVKLAGNSSLCPVSGWA
IYSKDNSVRIGSKGDVFVIREPFISCSPLECRTFFLTQGALLNDKHSNGTIKDRSPYRTLMSCP
IGEVPSPYNSRFESVAWSASACHDGINWLTIGISGPDNGAVAVLKYNGIITDTIKSWRNNILRT
QESECACVNGSCFTVMTDGPSNGQASYKIFRIEKGKIVKSVEMNAPNYHYEECSCYPDSSEITC
VCRDNWHGSNRPWVSFNQNLEYQIGYICSGIFGDNPRPNDKTGSCGPVSSNGANGVKGFSYKYG
NGVWIGRTKSISSRNGFEMIWDPNGWTGTDNNFSIKQDIVGINEWSGYSGSFVQHPELTGLDCI
RPCFWVELIRGRPKENTIWTSGSSISFCGVNSDTVGWSWPDGAELPFTIDK

N1-CA09-sNAp-94
H1N1 A/California/07/2009
VKLAGNSSLCPVSGWAPLSKDNSVRIGSKGEVFVIREPFISCSPLECRTFFLTQGALLNDKHSN
GTIKDRSPYRTLMSCPIGSVPSPSNSRFESVAWSASACHDGINWLTIGITGPDNGAVAILKYNG
IITDTIKSWRNNILRTQESECACVNGSCFTVMTDGPSNGQASYKIFRIEKGKIVKSVEMNAPNY
HYEECSCYPDSSEITCVCRDNWHGSNRPWVSFNQNLEYQIGYICSGIFGDNPRPNDKTGSCGPV
SSNGANGVKGFSFKYGNGVWIGRTKSISSRNGFEMIWDPNGWTGTDNNFSIKQDIVGINEWSGY
SGSFVMHPELTGLDCIVPCFWVELIRGRPKENTIWTSGSSISFCGVNSDTTGWSWPDGAELPFT
IDK

N1-CA09-sNAp-114
H1N1 A/California/07/2009
VKLAGNSSLCPVSGWAPLSKDNSVRIGSKGEVFVIREPFISCSPLECRTFFLTQGALLNDKHSN
GTIKDRSPYRTLMSCPIGSVPSPSNSRFESIAWSASACHDGINWLTIGITGPDNGAVAILKYNG
IITDTIKSWRNNILRTQESECACVNGSCFTVMTDGPSNGQASYKIFRIEKGKIVKSVEMNAPNY

HYEECSCYPDSSEITCVCRDNWHGSNRPWVSFNQNLEYQIGYICSGIFGDNPRPNDKTGSCGPV
SSNGANGVKGFSFKYGNGVWIGRTKSISSRNGFEMIWDPNGWTGTDNNFSIKQDIVGINEWSGY
SGSFVMHPELTGLDCIVPCFWVELIRGRPKENTIWTSGSSISFCGVNSDTTGWSWPDGAELPFT
IDK

<u>N1-CA09-sNAp-130</u>

H1N1 A/California/07/2009

VKLAGNSSLCPVSGWAPLSKDNSVRIGSKGDVFVIREPFISCSPLECRTFFLTQGALLNDKHSN
GTIKDRSPYRTLMSVPIGSVPSPYNARFESIAWSASACHDGINWLTIGITGPDNGAVAILKYNG
IITDTIKSWRNNILRTQESECACVNGSCFTVMTDGPSNGQASYKIFRIEKGKIVKSVEMNAPNY
HYEECSCYPDSSEITCVCRDNWHGSNRPWVSFNQNLEYQIGYICSGIFGDNPRPNDKTGSCGPV
SSNGANGVKGFSFKYGNGVWIGRTKSISSRNGFEMIWDPNGWTGTDNNFSIKQDIVGINEWSGY
SGSFVMHPELTGLDCIVPCFWVELIRGRPKENTIWTSGSSISFCGVNSDTTGWSWPDGAELPFT
IDK

<u>N1-CA09-sNAp-155</u>

H1N1 A/California/07/2009

VKLAGNSSLCPVSGWAPLSKDNSVRIGSKGDVFVIREPFISCSPLECRTFFLTQGALLNDKHSN
GTIKDRSPYRTLMSVPIGSVPSPYNARFESIAWSASACHDGINWLTIGITGPDNGAVAILKYNG
IITDTIKSWRNNILRTQESECACVNGSCFTVMTDGPSNGQASYKIFRIEKGKIVKSVEMNAPNY
HYEECSCYPDSSEITCVCRDNWHGSNRPWVSFNQNLEYQIGYICSGIFGDNPRPNDKTGSCGPV
SSNGANGVKGFSFKYGNGVWIGRTKSISSRNGFEMIWDPNGWTGTDNNFSIKQDIVGINEWSGY
SGSFVMHPELTGLDCIVPCFWVELIRGRPKENTIWTSGSSISFCGVNSDTVGWSWPDGAELPFT
IDK

<u>N1-CA09-sNAp-131</u>

H1N1 A/California/07/2009

VKLAGNSSLCPVSGWAPYSKDNSVRIGSKGDVFVIREPFISCSPLECRTFFLTQGALLNDKHSN
GTIKDRSPYRTLMSVPIGSVPSPYNARFESIAWSASACHDGINWLTIGITGPDNGAVAILKYNG
IITDTIKSWRNNILRTQESECACVNGSCFTVMTDGPSNGQASYKIFRIEKGKIVKSVEMNAPNY
HYEECSCYPDSSEITCVCRDNWHGSNRPWVSFNQNLEYQIGYICSGIFGDNPRPNDKTGSCGPV
SSNGANGVKGFSFKYGNGVWIGRTKSISSRNGFEMIWDPNGWTGTDNNFSIKQDIVGINEWSGY
SGSFVQHPELTGLDCIRPCFWVELIRGRPKENTIWTSGSSISFCGVNSDTTGWSWPDGAELPFT
IDK

<u>N1-CA09-sNAp-134</u>

H1N1 A/California/07/2009

VKLAGNSSLCPVSGWAPLSKDNSVRIGSKGDVFVIREPFISCSPLECRTFFLTQGALLNDKHSN
GTIKDRSPYRTLMSVPIGEVPSPYNARFESIAWSASACHDGINWLTIGITGPDNGAVAILKYNG
IITDTIKSWRNNILRTQESECACVNGSCFTVMTDGPSNGQASYKIFRIEKGKIVKSVEMNAPNY
HYEECSCYPDSSEITCVCRDNWHGSNRPWVSFNQNLEYQIGYICSGIFGDNPRPNDKTGSCGPV
SSNGANGVKGFSFKYGNGVWIGRTKSISSRNGFEMIWDPNGWTGTDNNFSIKQDIVGINEWSGY
SGSFVMHPELTGLDCIVPCFWVELIRGRPKENTIWTSGSSISFCGVNSDTVGWSWPDGAELPFT
IDK

N1-CA09-sNAp-130 + Stalk
H1N1 A/California/07/2009
SHSIQLGNQNQIETCNQSVITYENNTWVNQTYVNISNTNFAAGQSVVSVKLAGNSSLCPVSGWA
PLSKDNSVRIGSKGDVFVIREPFISCSPLECRTFFLTQGALLNDKHSNGTIKDRSPYRTLMSVP
IGSVPSPYNARFESIAWSASACHDGINWLTIGITGPDNGAVAILKYNGIITDTIKSWRNNILRT
QESECACVNGSCFTVMTDGPSNGQASYKIFRIEKGKIVKSVEMNAPNYHYEECSCYPDSSEITC
VCRDNWHGSNRPWVSFNQNLEYQIGYICSGIFGDNPRPNDKTGSCGPVSSNGANGVKGFSFKYG
NGVWIGRTKSISSRNGFEMIWDPNGWTGTDNNFSIKQDIVGINEWSGYSGSFVMHPELTGLDCI
VPCFWVELIRGRPKENTIWTSGSSISFCGVNSDTTGWSWPDGAELPFTIDK

N8-JD13-sNAp-282
H10N8 A/Jiangxi-Donghu/346-2/2013
HFMNNTEALCDAKGFAPFSKDNGIRIGSRGHVFVIREPFVSCSPTECRTFFLTQGSLLNDKHSN
GTVKDRSPYRTLMSVEIGSSPNVYQARFEAVAWSATACHDGKKWMTIGVTGPDAKAVAVVHYGG
IPTDVINSWAGDILRTQESSCTCIQGECFWVMTDGPANRQAQYRAFKAKQGKIVGQAEISFNGG
HIEECSCYPNEGKVECVCKDNWTGTNRPVLVISPDLSYRVGYLCAGLPSDTPRGEDSQFTGSCT
SPMGNQGYGVKGFGFRQGNDVWMGRTISRTSRSGFEILKVRNGWVQNSKEQIKRQVVVDNLNWS
GYSGSFTLPAELTKRNCLVPCFWVEMIRGNPEEKTIWTSSSSIVMCGVDHEIADWSWHDGAILP
FDIDKM

N8-JD13-sNAp-285
H10N8 A/Jiangxi-Donghu/346-2/2013
HFMNNTEALCDAKGFAPFSKDNGIRIGSRGHVFVIREPFVSCSPTECRTFFLTQGSLLNDKHSN
GTVKDRSPYRTLMSVPIGSSPNVYQARFEAVAWSATACHDGKKWMTIGVTGPDAKAVAVVHYGG
IPTDVINSWAGDILRTQESSCTCIQGECFWVMTDGPANRQAQYRAFKAKQGKIVGQAEISFNGG
HIEECSCYPNEGKVECVCKDNWTGTNRPVLVISPDLSYRVGYLCAGLPSDTPRGEDSQFTGSCT
SPMGNQGYGVKGFGFRQGNDVWMGRTISRTSRSGFEILKVRNGWVQNSKEQIKRQVVVDNLNWS
GYSGSFTLPAELTKRNCLVPCFWVEMIRGNPEEKTIWTSSSSIVMCGVDHEIADWSWHDGAILP
FDIDKM

N2-WI05-desNAp-156
H3N2 A/Wisconsin/67/2005
EYRNWSKPQCNITGFAPFSKDNSIRLSAGGDIWVTREPYVSCDPDKCYQFALGQGTTLNNVHSN
DTVHDRTPYRTLLMNELGEPFHLGTKQVCIAWSSSSCHDGKAWLHVCVTGDDKNATASFIYNGR
LVDSIVSWSKEILRTQESECVCINGTCTVVMTDGSASGKADTKILFIEEGKIVHTSTLSGSAQH
VEECSCYPRYLGVRCVCRDNWKGSNRPIVDINIKDYSIVSSYVCSGLVGDTPRKNDSSSSSHCL
DPNNEEGGHGVKGWAFDDGNDVWMGRTISEKLRSGYETFKVIEGWSNPNSKLQINRQVIVDRGN
RSGYSGIFSVEGKSCINRCFYVELIRGRKEETEVLWTSNSIVVFCGTSGTYGTGSWPDGADINL
MPI

N2-WI05-desNAp-157
H3N2 A/Wisconsin/67/2005
EYRNWSKPQCNITGFAPFSKDNSIRLSAGGDIWVTREPYVSCDPDKCYQFALGQGTTLNNVHSN
DTVHDRTPYRTLLMNELGVPFHLGTKQVCVAWSSSSCHDGKAWLHVCVSGDDKNATASFIYNGR
LVDSIVSWSKEILRTQESECVCINGTCTVVMTDGSASGKADTKILFIEEGKIVHTSTLSGSAQH

VEECSCYPRYLGVRCVCRDNWKGSNRPIVDINIKDYSIVSSYVCSGLVGDTPRKNDSSSSSHCL
DPNNEEGGHGVKGWAFDDGNDVWMGRTISEKLRSGYETFKVIEGWSNPNSKLQINRQVIVDRGN
RSGYSGIFSVEGKSCINRCFYVELIRGRKEETEVLWTSNSIVVFCGTSGTYGTGSWPDGADINL
MPI

<u>N2-WI05-desNAp-158</u>
H3N2 A/Wisconsin/67/2005
EYRNWSKPQCNITGFAIFSKDNSIRLSAGGDIWVTREPYVSCDPDKCYQFALGQGTTLNNVHSN
DTVHDRTPYRTLLMNELGVPFHLGTKQVCVAWSSSSCHDGKAWLHVCVSGDDKNATASFIYNGR
LVDSIVSWSKEILRTQESECVCINGTCTVVMTDGSASGKADTKILFIEEGKIVHTSTLSGSAQH
VEECSCYPRYLGVRCVCRDNWKGSNRPIVDINIKDYSIVSSYVCSGLVGDTPRKNDSSSSSHCL
DPNNEEGGHGVKGWAFDDGNDVWMGRTISEKLRSGYETFKVIEGWSNPNSKLQINRQVIVDRGN
RSGYSGIFSVEGKSCINRCFYVELIRGRKEETEVLWTSNSIVVFCGTSGTYGTGSWPDGADINL
MPI

<u>N2-WI05-desNAp-249</u>
H3N2 A/Wisconsin/67/2005
EYRNWSKPQCNITGFAPFSKDNSIRLSAGGDIWVTREPYVSCDPDKCYQFALGQGTTLNNVHSN
DTVHDRTPYRTLLMNELGQPFHLGTKQVCIAWSSSSCHDGKAWLHVCVTGDDKNATASFIYNGR
LVDSIVSWSKEILRTQESECVCINGTCTVVMTDGSASGKADTKILFIEEGKIVHTSTLSGSAQH
VEECSCYPRYLGVRCVCRDNWKGSNRPIVDINIKDYSIVSSYVCSGLVGDTPRKNDSSSSSHCL
DPNNEEGGHGVKGWAFDDGNDVWMGRTISEKLRSGYETFKVIEGWSNPNSKLQINRQVIVDRGN
RSGYSGIFSVEGKSCINRCFYVELIRGRKEETEVLWTSNSIVVFCGTSGTYGTGSWPDGADINL
MP

<u>N2-WI05-desNAp-255</u>
H3N2 A/Wisconsin/67/2005
EYRNWSKPQCNITGFAPFSKDNSIRLSAGGDIWVTREPYVSCDPDKCYQFALGQGTTLNNVHSN
DTVHDRTPYRTLLMNELGQPFHLGTKQVCVAWSSSSCHDGKAWLHVCVSGDDKNATASFIYNGR
LVDSIVSWSKEILRTQESECVCINGTCTVVMTDGSASGKADTKILFIEEGKIVHTSTLSGSAQH
VEECSCYPRYLGVRCVCRDNWKGSNRPIVDINIKDYSIVSSYVCSGLVGDTPRKNDSSSSSHCL
DPNNEEGGHGVKGWAFDDGNDVWMGRTISEKLRSGYETFKVIEGWSNPNSKLQINRQVIVDRGN
RSGYSGIFSVEGKSCINRCFYVELIRGRKEETEVLWTSNSIVVFCGTSGTYGTGSWPDGADINL
MPI

<u>N1-MI15-sNAp-155</u>
VKLAGNSSLCPVSGWAPLSKDNSVRIGSKGDVFVIREPFISCSPLECRTFFLTQGALLNDKHSN
GTIKDRSPYRTLMSVPIGSVPSPYNARFESIAWSASACHDGINWLTIGITGPDSGAVAILKYNG
IITDTIKSWRNNILRTQESECACVNGSCFTIMTDGPSDGQASYKIFRIEKGKIIKSVEMKAPNY
HYEECSCYPDSSEITCVCRDNWHGSNRPWVSFNQNLEYQMGYICSGVFGDNPRPNDKTGSCGPV
SSNGANGVKGFSFKYGNGVWIGRTKSISSRKGFEMIWDPNGWTGTDNKFSIKQDIVGINEWSGY
SGSFVMHPELTGLDCIVPCFWVELIRGRPEENTIWTSGSSISFCGVNSDTVGWSWPDGAELPFT
IDK

N1-MI15-sNAp-174

H1N1 A/Michigan/45/2015

VKLAGNSSLCPVSGWAPLSKDNSVRIGSKGDVFVIREPFISCSPLECRQFFLTQGALLNDKHSN
GTIKDRSPYRTLMSVPIGSVPSPYNARFESIAWSASACHDGINWLTIGITGPDSGAVAILKYNG
IITDTIKSWRNNILRTQESECACVNGSCFTIMTDGPSDGQASYKIFRIEKGKIIKSVEMKAPNY
HYEECSCYPDSSEITCVCRDNWHGSNRPWVSFNQNLEYQMGYICSGVFGDNPRPNDKTGSCGPV
SSNGANGVKGFSFKYGNGVWIGRTKSISSRKGFEMIWDPNGWTGTDNKFSIKQDIVGINEWSGY
SGSFVMHPELTGLDCIVPCFWVELIRGRPEENTIWTSGSSISFCGVNSDTVGWSWPDGAELPFT
IDK

N1-MI15-sNAp-165

H1N1 A/Michigan/45/2015

VKLAGNSSLCPVSGWAPLSKDNSVRIGSKGDIFVIREPFISCSPLECRTFFLTQGALLNDKHSN
GTIKDRSPYRTLMSVPIGSPPSPYNARFESIAWSASACHDGINWLTIGITGPDSGAVAILKYNG
IITDTIKSWRNNILRTQESECACVNGSCFTIMTDGPSDGQASYKIFRIEKGKIIKSVEMKAPNY
HYEECSCYPDSSEITCVCRDNWHGSNRPWVSFNQNLEYQMGYICSGVFGDNPRPNDKTGSCGPV
SSNGANGVKGFSFKYGNGVWIGRTKSISSRKGFEMIWDPNGWTGTDNKFSIKQDIVGINEWSGY
SGSFVMHPELTGLDCIVPCFWVELIRGRPEENTIWTSGSSISFCGVNSDTVGWSWPDGAELPFT
IDK

N1-MI15-sNAp-176

H1N1 A/Michigan/45/2015

VKLAGNSSLCPVSGWAPLSKDNSVRIGSKGDIFVIREPFISCSPLECRQFFLTQGALLNDKHSN
GTIKDRSPYRTLMSVPIGSPPSPYNARFESIAWSASACHDGINWLTIGITGPDSGAVAILKYNG
IITDTIKSWRNNILRTQESECACVNGSCFTIMTDGPSDGQASYKIFRIEKGKIIKSVEMKAPNY
HYEECSCYPDSSEITCVCRDNWHGSNRPWVSFNQNLEYQMGYICSGVFGDNPRPNDKTGSCGPV
SSNGANGVKGFSFKYGNGVWIGRTKSISSRKGFEMIWDPNGWTGTDNKFSIKQDIVGINEWSGY
SGSFVMHPELTGLDCIVPCFWVELIRGRPEENTIWTSGSSISFCGVNSDTVGWSWPDGAELPFT
IDK

N1-MI15-sNAp-183

H1N1 A/Michigan/45/2015

VKLAGNSSLCPVSGWAPLAKDNSVRIGSKGDVFVIREPFISCSPLECRMFFLTQGALLNDKHSN
GTIKDRSPYRTLMSVPLGSVPSPYNARFESIAWSASACHDGINWLTIGITGPDSGAVAILKYNG
IITDTIKSWRNNILRTQESECACVNGSCFTIMTDGPSDGQASYKIFRIEKGKIIKSVEMKAPNY
HYEECSCYPDSSEITCVCRDNWHGSNRPWVSFNQNLEYQMGYICSGVFGDNPRPNDKTGSCGPV
SSNGANGVKGFSFKYGNGVWIGRTKSISSRKGFEMIWDPNGWTGTDNKFSIKQDIVGINEWSGY
SGSFVMHPELTGLDCIVPCFWVELIRGRPEENTIWTSGSIIAFCGVNSDTVGWSWPDGAELPFT
IDK

N1-VN04-sNAp-155

H5N1 A/Vietnam/1203/2004

VKLAGNSSLCPINGWAPLSKDNSIRIGSKGDVFVIREPFISCSHLECRTFFLTQGALLNDKHSN
GTVKDRSPHRTLMSVPVGSAPSPYNARFESIAWSASACHDGTSWLTIGITGPDNGAVAILKYNG

IITDTIKSWRNNILRTQESECACVNGSCFTVMTDGPSNGQASYKIFKMEKGKVVKSVELDAPNY
HYEECSCYPNAGEITCVCRDNWHGSNRPWVSFNQNLEYQIGYICSGVFGDNPRPNDGTGSCGPV
SSNGAYGVKGFSFKYGNGVWIGRTKSTNSRSGFEMIWDPNGWTETDSSFSVKQDIVAITDWSGY
SGSFVMHPELTGLDCIVPCFWVELIRGRPKESTIWTSGSSISFCGVNSDTVGWSWPDGAELPFT
IDK

<u>N1-VN04-sNAp-354</u>

H5N1 A/Vietnam/1203/2004

VKLAGNSSLCPINGWAPLSKDNSVRIGSKGDVFVIREPFISCSHLECRQFFLTQGALLNDKHSN
GTVKDRSPHRTLMSVPIGSVPSPYNARFESIAWSASACHDGTSWLTIGITGPDNGAVAILKYNG
IITDTIKSWRNNILRTQESECACVNGSCFTVMTDGPSNGQASYKIFKMEKGKVVKSVELDAPNY
HYEECSCYPNAGEITCVCRDNWHGSNRPWVSFNQNLEYQIGYICSGVFGDNPRPNDGTGSCGPV
SSNGAYGVKGFSFKYGNGVWIGRTKSTNSRSGFEMIWDPNGWTETDSSFSVKQDIVAITDWSGY
SGSFVMHPELTGLDCIVPCFWVELIRGRPKESTIWTSGSSISFCGVNSDTVGWSWPDGAELPFT
IDK

<u>N1-WSN33-sNAp-155</u>

H1N1 A/WSN/1933

VILTGNSSLCPIRGWAPLSKDNGIRIGSKGDVFVIREPFISCSHLECRTFFLTQGALLNDKHSR
GTFKDRSPYRALMSVPVGSAPSPYNARFESIAWSASACHDGMGWLTIGITGPDDGAVAILKYNR
IITETIKSWRKNILRTQESECTCVNGSCFTIMTDGPSDGLASYKIFKIEKGKVTKSIELNAPNS
HYEECSCYPDTGKVMCVCRDNWHGSNRPWVSFDQNLDYKIGYICSGVFGDNPRPKDGTGSCGPV
SADGANGVKGFSYKYGNGVWIGRTKSDSSRHGFEMIWDPNGWTETDSRFSMRQDVVAITNRSGY
SGSFVMHPELTGLDCMVPCFWVELIRGLPEEDAIWTSGSIISFCGVNSDTVDWSWPDGAELPFT
IDK

<u>N1-WSN33-sNAp-366</u>

H1N1 A/WSN/1933

VILTGNSSLCPIRGWAPLSKDNSVRIGSKGDVFVIREPFISCSHLECRTFFLTQGALLNDKHSR
GTFKDRSPYRTLMSVPIGSVPSPYNARFESIAWSASACHDGMGWLTIGITGPDDGAVAILKYNG
IITETIKSWRKNILRTQESECTCVNGSCFTIMTDGPSDGLASYKIFKIEKGKVTKSIELNAPNS
HYEECSCYPDTGKVMCVCRDNWHGSNRPWVSFDQNLDYKIGYICSGVFGDNPRPKDGTGSCGPV
SADGANGVKGFSYKYGNGVWIGRTKSDSSRHGFEMIWDPNGWTETDSRFSMRQDVVAITNRSGY
SGSFVMHPELTGLDCMVPCFWVELIRGLPEEDAIWTSGSIISFCGVNSDTVDWSWPDGAELPFT
IDK

<u>N1-WSN33-sNAp-367</u>

H1N1 A/WSN/1933

VILTGNSSLCPIRGWAPLSKDNSVRIGSKGDVFVIREPFISCSHLECRQFFLTQGALLNDKHSR
GTFKDRSPYRTLMSVPIGSVPSPYNARFESIAWSASACHDGMGWLTIGITGPDDGAVAILKYNG
IITETIKSWRKNILRTQESECTCVNGSCFTIMTDGPSDGLASYKIFKIEKGKVTKSIELNAPNS
HYEECSCYPDTGKVMCVCRDNWHGSNRPWVSFDQNLDYKIGYICSGVFGDNPRPKDGTGSCGPV
SADGANGVKGFSYKYGNGVWIGRTKSDSSRHGFEMIWDPNGWTETDSRFSMRQDVVAITNRSGY
SGSFVMHPELTGLDCMVPCFWVELIRGLPEEDAIWTSGSIISFCGVNSDTVDWSWPDGAELPFT
IDK

N1-WSN33-sNAp-375

H1N1 A/WSN/1933

VILTGNSSLCPIRGWAPLAKDNSVRIGSKGDVFVIREPFISCSHLECRMFFLTQGALLNDKHSR
GTFKDRSPYRTLMSVPLGSVPSPYNARFESIAWSASACHDGMGWLTIGITGPDDGAVAILKYNG
IITETIKSWRKNILRTQESECTCVNGSCFTIMTDGPSDGLASYKIFKIEKGKVTKSIELNAPNS
HYEECSCYPDTGKVMCVCRDNWHGSNRPWVSFDQNLDYKIGYICSGVFGDNPRPKDGTGSCGPV
SADGANGVKGFSYKYGNGVWIGRTKSDSSRHGFEMIWDPNGWTETDSRFSMRQDVVAITNRSGY
SGSFVMHPELTGLDCMVPCFWVELIRGLPEEDAIWTSGSIIAFCGVNSDTVDWSWPDGAELPFT
IDK

N1-WSN33-sNAp-378

H1N1 A/WSN/1933

VILTGNSSLCPIRGWAPLAKDNSVRIGSKGDVFVIREPFISCSPLECRMFFLTQGALLNDKHSR
GTFKDRSPYRTLMSVPLGSVPSPYNARFESIAWSASACHDGMGWLTIGITGPDDGAVAILKYNG
IITETIKSWRKNILRTQESECACVNGSCFTVMTDGPSDGLASYKIFKIEKGKITKSIEMNAPNS
HYEECSCYPDTGKVMCVCRDNWHGSNRPWVSFDQNLDYKIGYICSGVFGDNPRPKDGTGSCGPV
SADGANGVKGFSFKYGNGVWIGRTKSDSSRHGFEMIWDPNGWTETDSRFSMRQDIVAITNRSGY
SGSFVMHPELTGLDCIVPCFWVELIRGLPEEDAIWTSGSIIAFCGVNSDTVDWSWPDGAELPFT
IDK

N1-CA09-sNAp-155-T466A

H1N1 A/California/07/2009

VKLAGNSSLCPVSGWAPLSKDNSVRIGSKGDVFVIREPFISCSPLECRTFFLTQGALLNDKHSN
GTIKDRSPYRTLMSVPIGSVPSPYNARFESIAWSASACHDGINWLTIGITGPDNGAVAILKYNG
IITDTIKSWRNNILRTQESECACVNGSCFTVMTDGPSNGQASYKIFRIEKGKIVKSVEMNAPNY
HYEECSCYPDSSEITCVCRDNWHGSNRPWVSFNQNLEYQIGYICSGIFGDNPRPNDKTGSCGPV
SSNGANGVKGFSFKYGNGVWIGRTKSISSRNGFEMIWDPNGWTGTDNNFSIKQDIVGINEWSGY
SGSFVMHPELTGLDCIVPCFWVELIRGRPKENTIWTSGSSISFCGVNSDTVGWSWPDGAELPFA
IDK

All membrane-anchored NA constructs shown below were tested without any added sequence features:

<u>N1-CA09-WT full-length with C-terminal myc tag</u>

H1N1 A/California/07/2009

```
MNPNQKIITIGSVCMTIGMANLILQIGNIISIWISHSIQLGNQNQIETCNQSVITYENNTWVNQ
TYVNISNTNFAAGQSVVSVKLAGNSSLCPVSGWAIYSKDNSVRIGSKGDVFVIREPFISCSPLE
CRTFFLTQGALLNDKHSNGTIKDRSPYRTLMSCPIGEVPSPYNSRFESVAWSASACHDGINWLT
IGISGPDNGAVAVLKYNGIITDTIKSWRNNILRTQESECACVNGSCFTVMTDGPSNGQASYKIF
RIEKGKIVKSVEMNAPNYHYEECSCYPDSSEITCVCRDNWHGSNRPWVSFNQNLEYQIGYICSG
IFGDNPRPNDKTGSCGPVSSNGANGVKGFSYKYGNGVWIGRTKSISSRNGFEMIWDPNGWTGTD
NNFSIKQDIVGINEWSGYSGSFVQHPELTGLDCIRPCFWVELIRGRPKENTIWTSGSSISFCGV
NSDTVGWSWPDGAELPFTIDKGGSGSGGEQKLISEEDL
```

<u>N1-CA09-sNAp-130 full-length with C-terminal myc tag</u>

H1N1 A/California/07/2009

```
MNPNQKIITIGSVCMTIGMANLILQIGNIISIWISHSIQLGNQNQIETCNQSVITYENNTWVNQ
TYVNISNTNFAAGQSVVSVKLAGNSSLCPVSGWAPLSKDNSVRIGSKGDVFVIREPFISCSPLE
CRTFFLTQGALLNDKHSNGTIKDRSPYRTLMSVPIGSVPSPYNARFESIAWSASACHDGINWLT
IGITGPDNGAVAILKYNGIITDTIKSWRNNILRTQESECACVNGSCFTVMTDGPSNGQASYKIF
RIEKGKIVKSVEMNAPNYHYEECSCYPDSSEITCVCRDNWHGSNRPWVSFNQNLEYQIGYICSG
IFGDNPRPNDKTGSCGPVSSNGANGVKGFSFKYGNGVWIGRTKSISSRNGFEMIWDPNGWTGTD
NNFSIKQDIVGINEWSGYSGSFVMHPELTGLDCIVPCFWVELIRGRPKENTIWTSGSSISFCGV
NSDTTGWSWPDGAELPFTIDKGGSGSGGEQKLISEEDL
```
