# Supplementary material for: Structure-based design of stabilized recombinant influenza neuraminidase tetramers
Source: Nat Commun. 2022 Apr 5;13:1825. doi: 10.1038/s41467-022-29416-z (PMC8983682; doi:10.1038/s41467-022-29416-z)
Supplement: Supplementary file 4 — Supplementary Data 2 [file 41467_2022_29416_MOESM4_ESM.pdf]

N1-CA09-WT

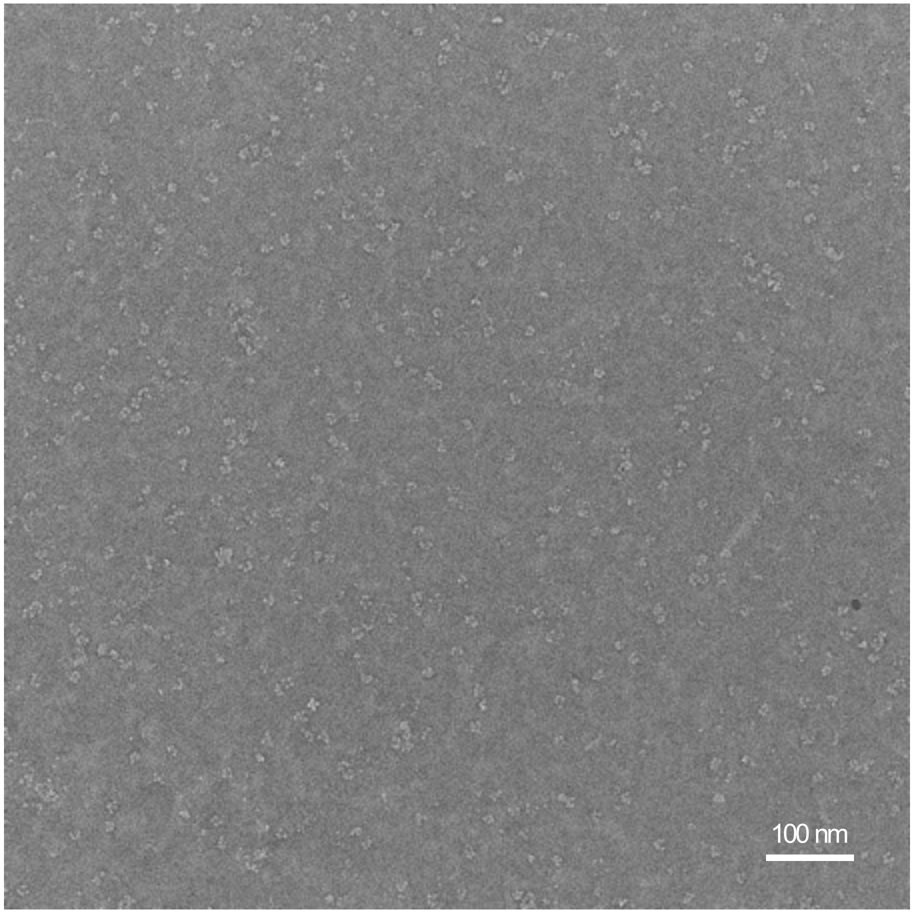

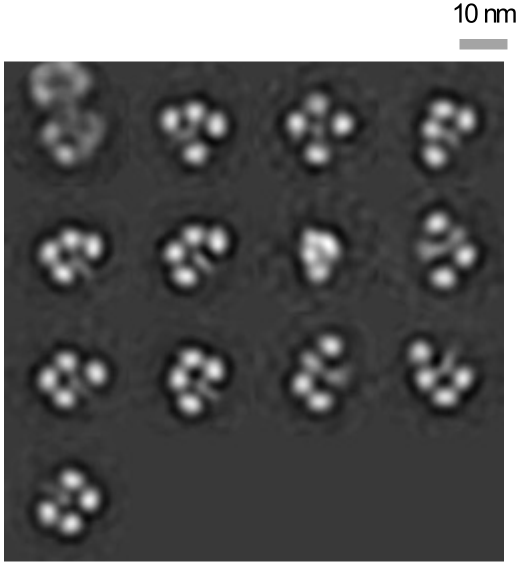

Total particles picked: 5271
Total particles clearly in closed state: 0
Total particles clearly in open state: 3974

N1-MI15-WT

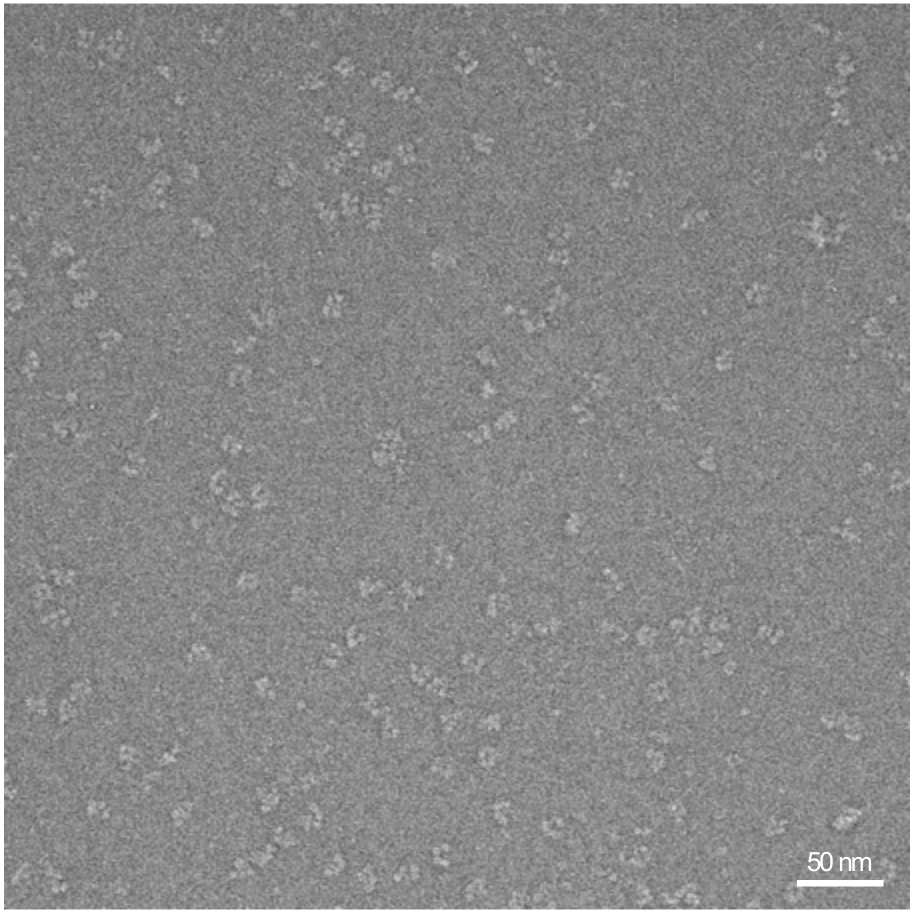

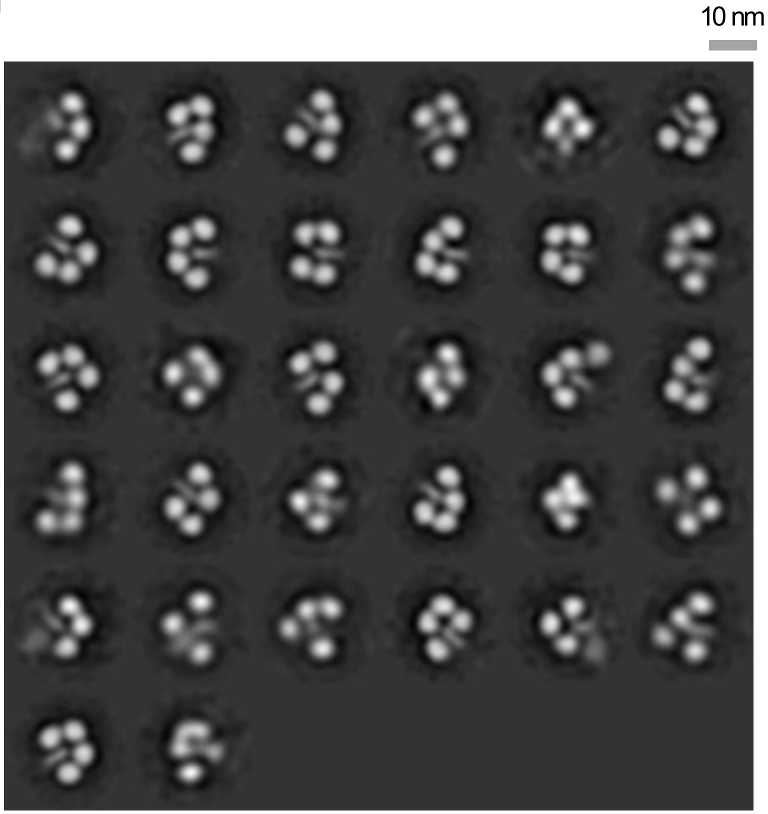

<u>Total particles picked: 11230</u>
Total particles clearly in closed state: 0
Total particles clearly in open state: 10076

N1-NC99-WT

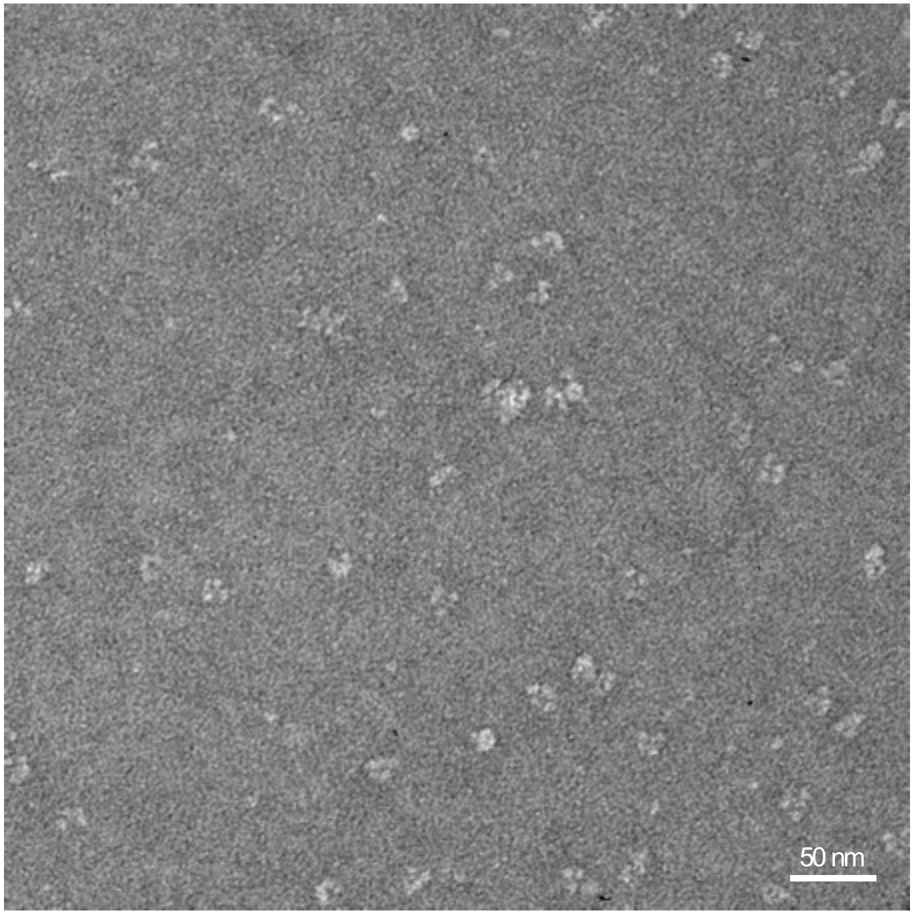

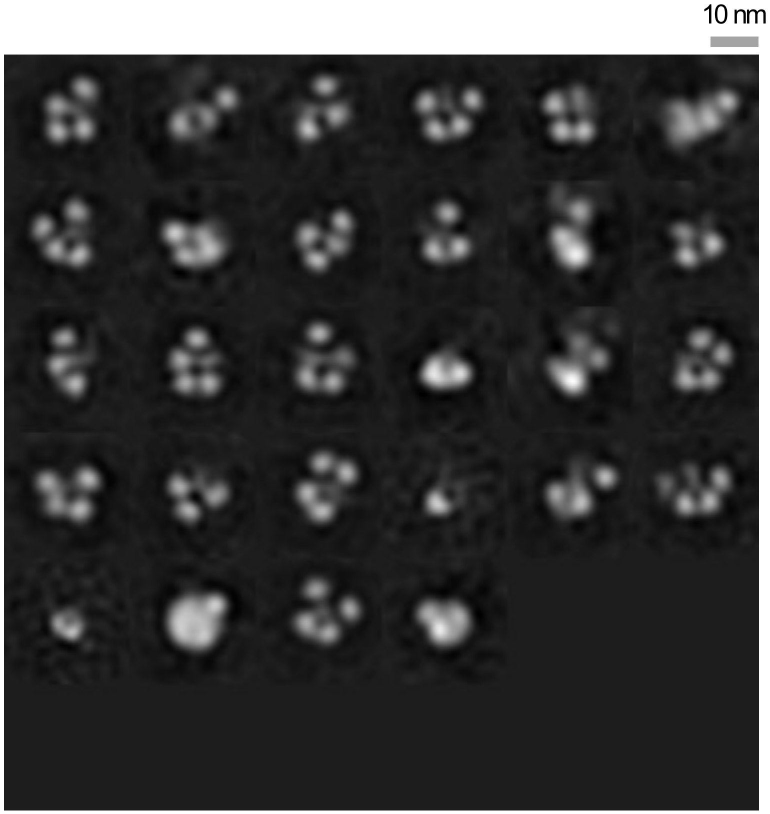

Total particles picked: 5273:
Total particles clearly in closed state: 101
Total particles clearly in open state: 3452

N1-WSN33-WT

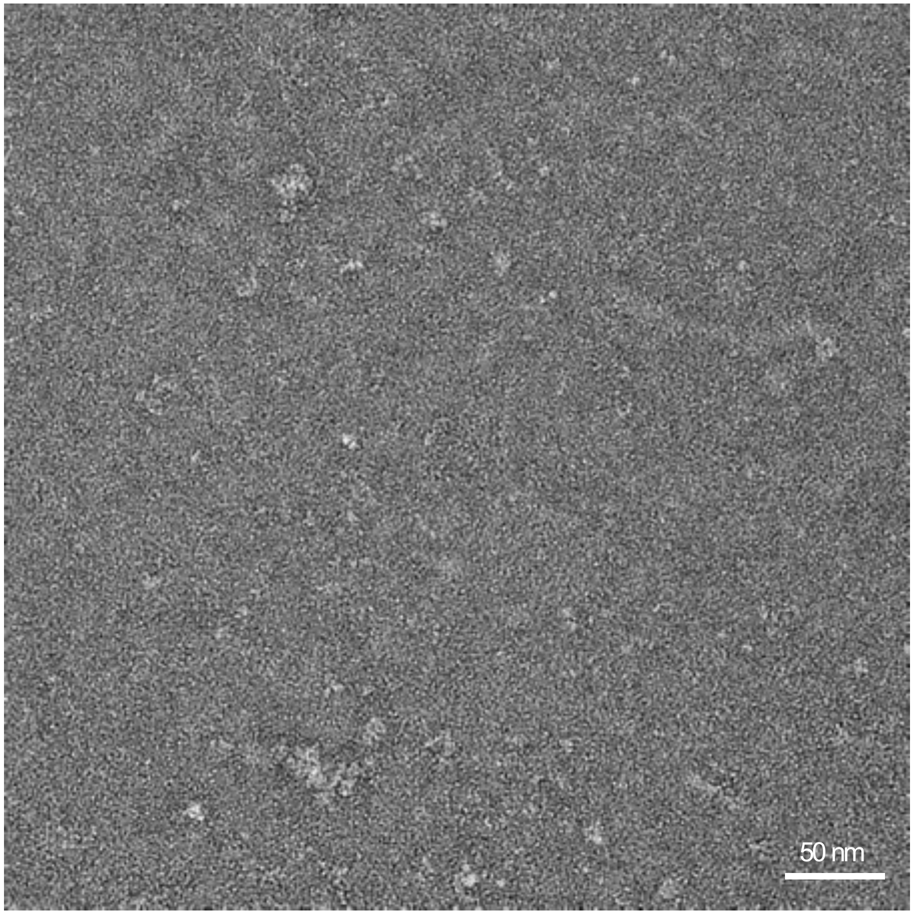

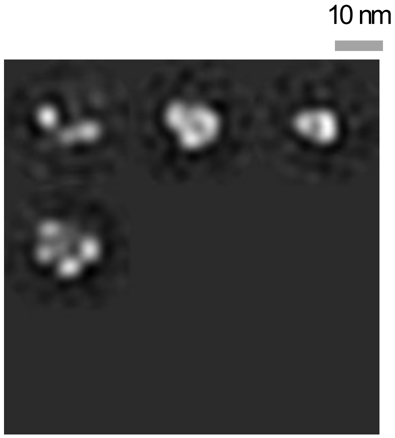

<u>Total particles picked: 2320</u>
Total particles clearly in closed state: 0
Total particles clearly in open state: 238

N1-BV18-WT

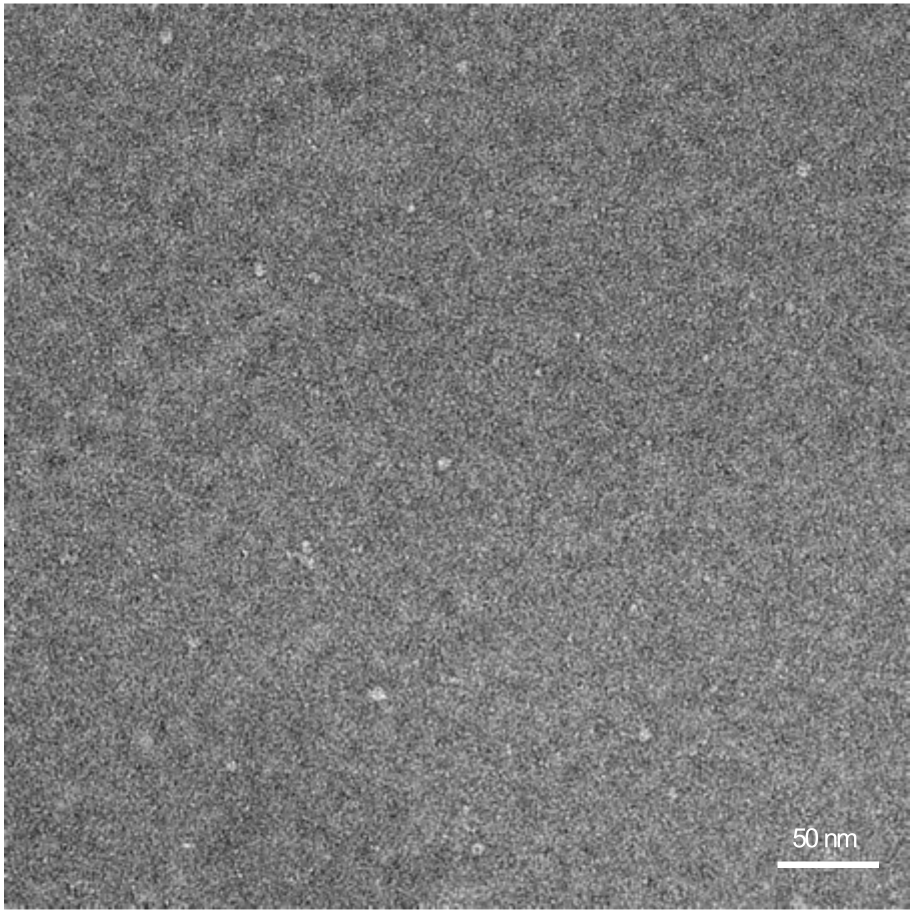

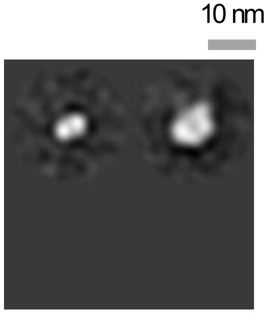

Total particles picked: 2281
No clear tetramers observed to quantify

N1-VN04-WT

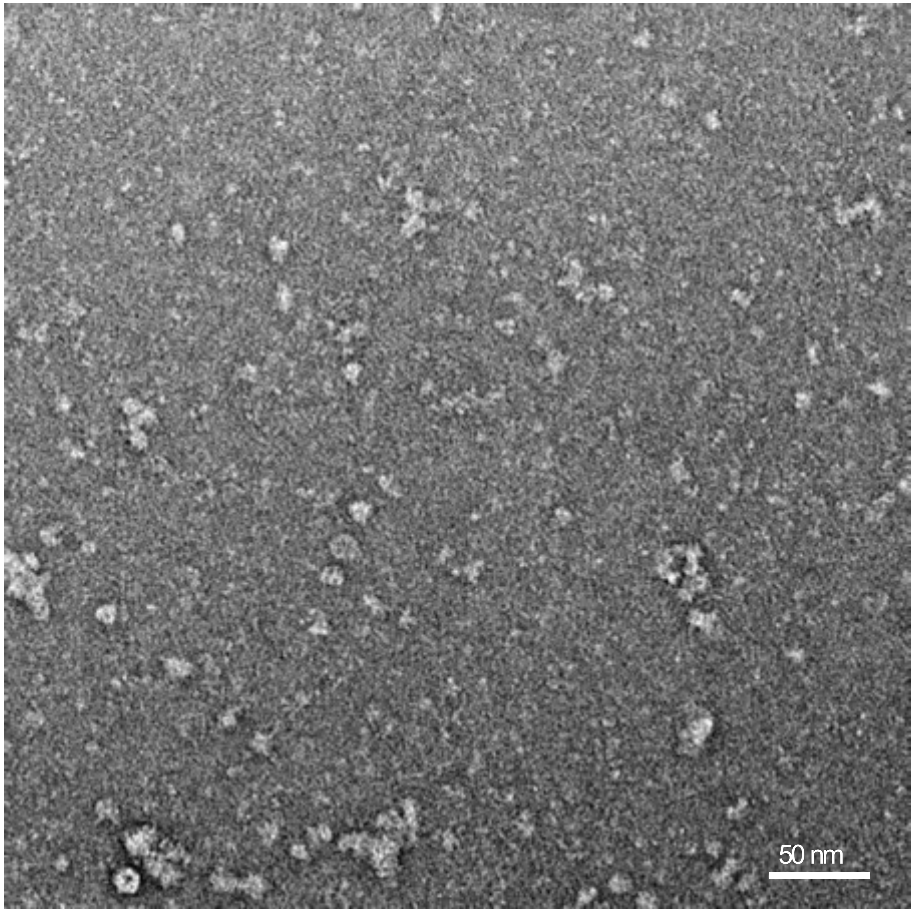

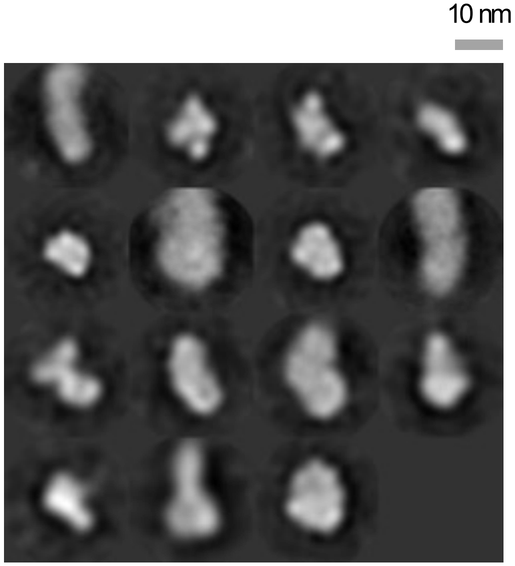

Total particles picked: 5722
No clear tetramers observed to quantify

N6-SI14-WT

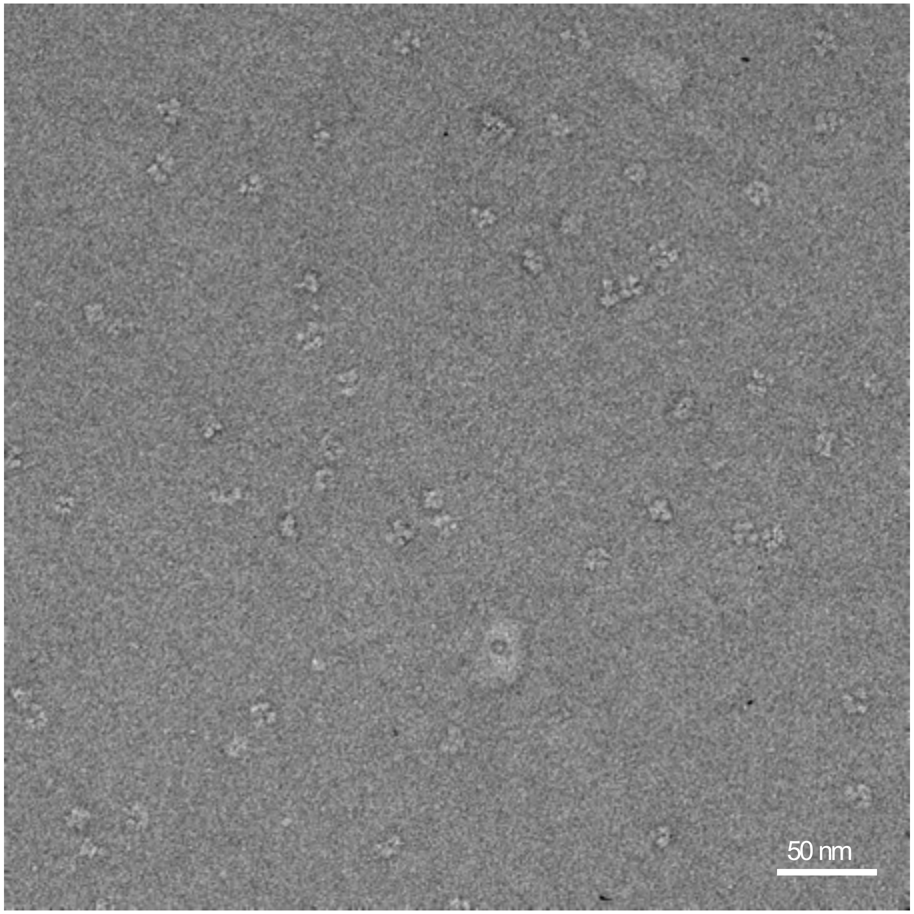

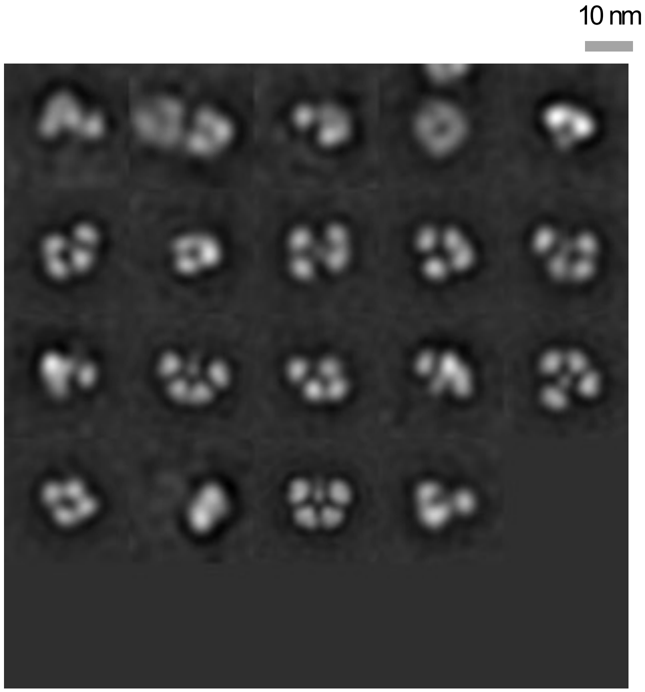

Total particles picked: 3306
Total particles clearly in closed state: 116
Total particles clearly in open state: 1708

N2-MO99-WT

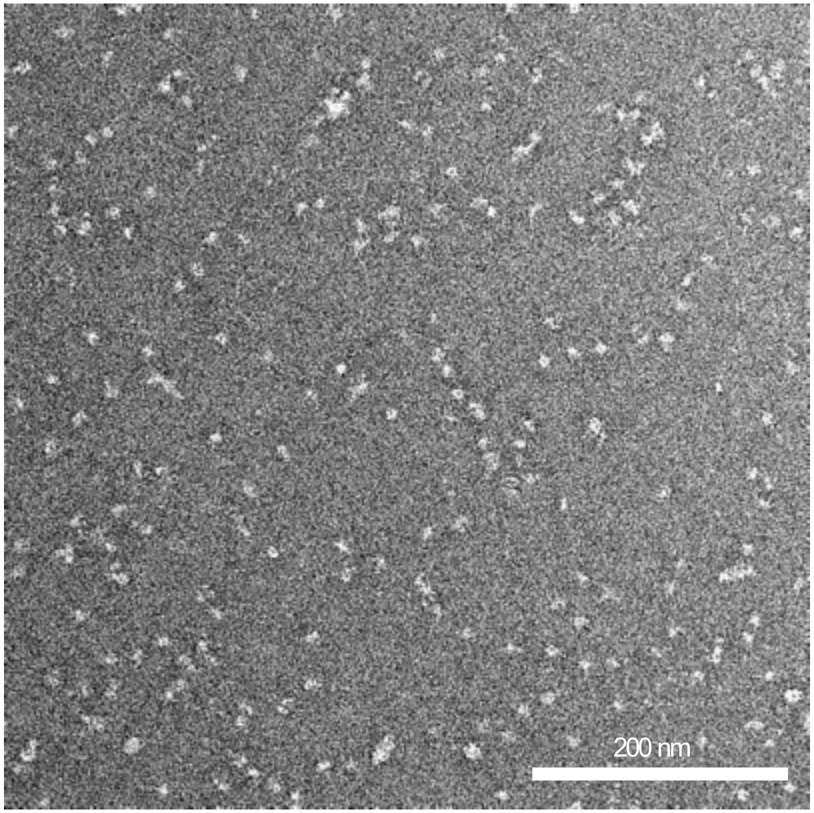

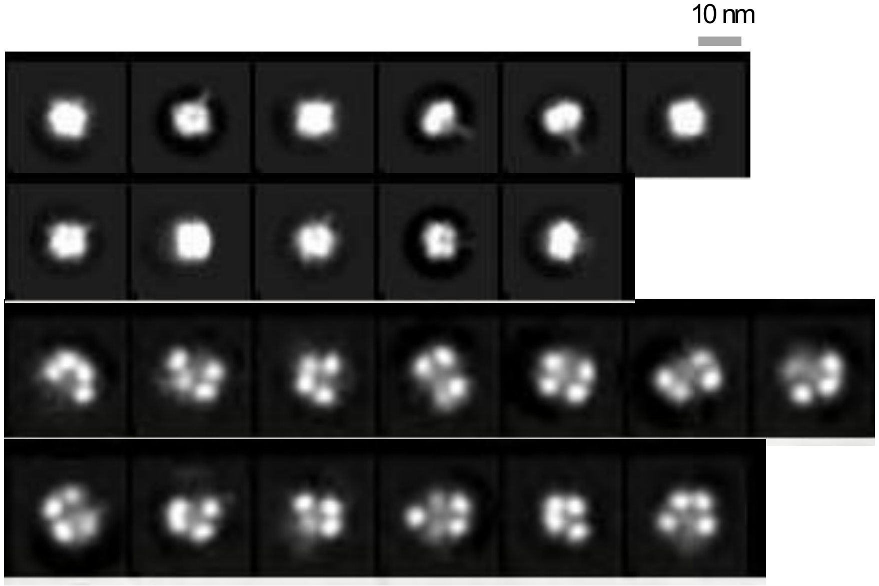

Total particles picked: 60706
Total particles clearly in closed state: 24213
Total particles clearly in open state: 6424

N4-DB16-WT

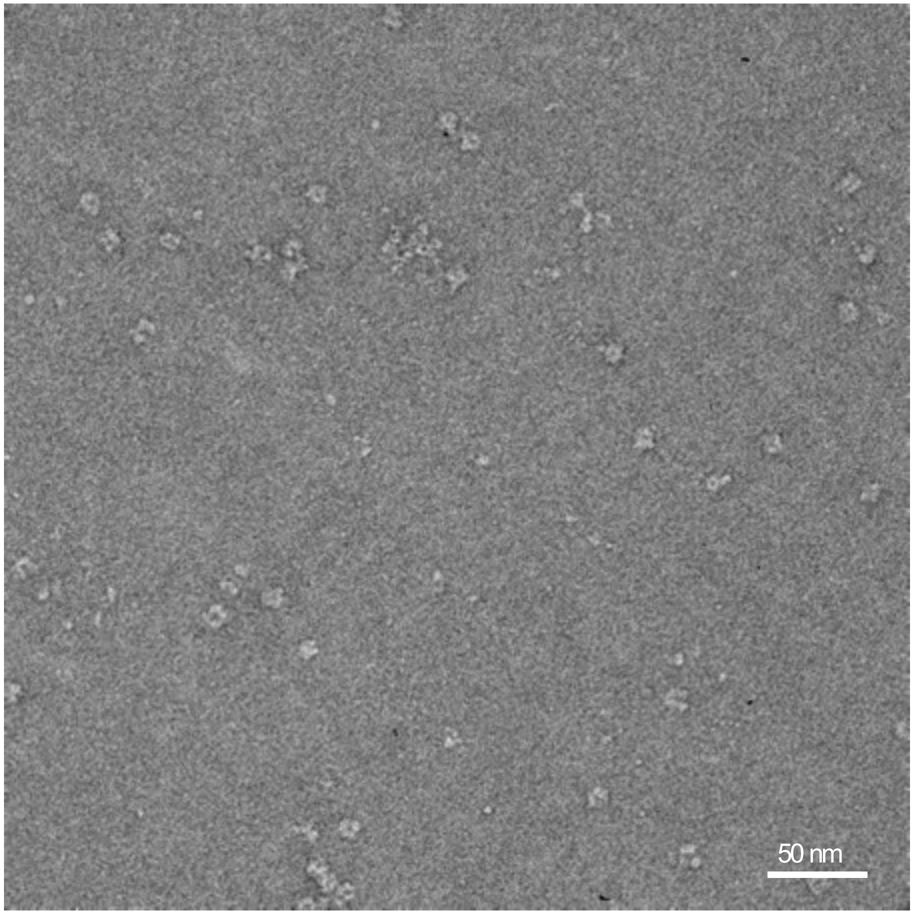

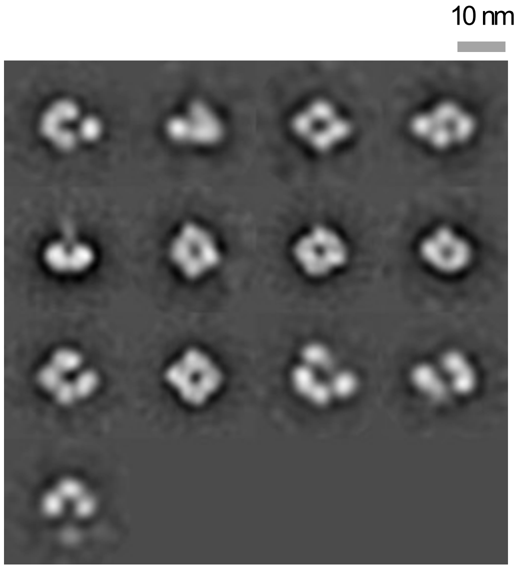

Total particles picked: 2604
Total particles clearly in closed state: 1395
Total particles clearly in open state: 619

N7-NE03-WT

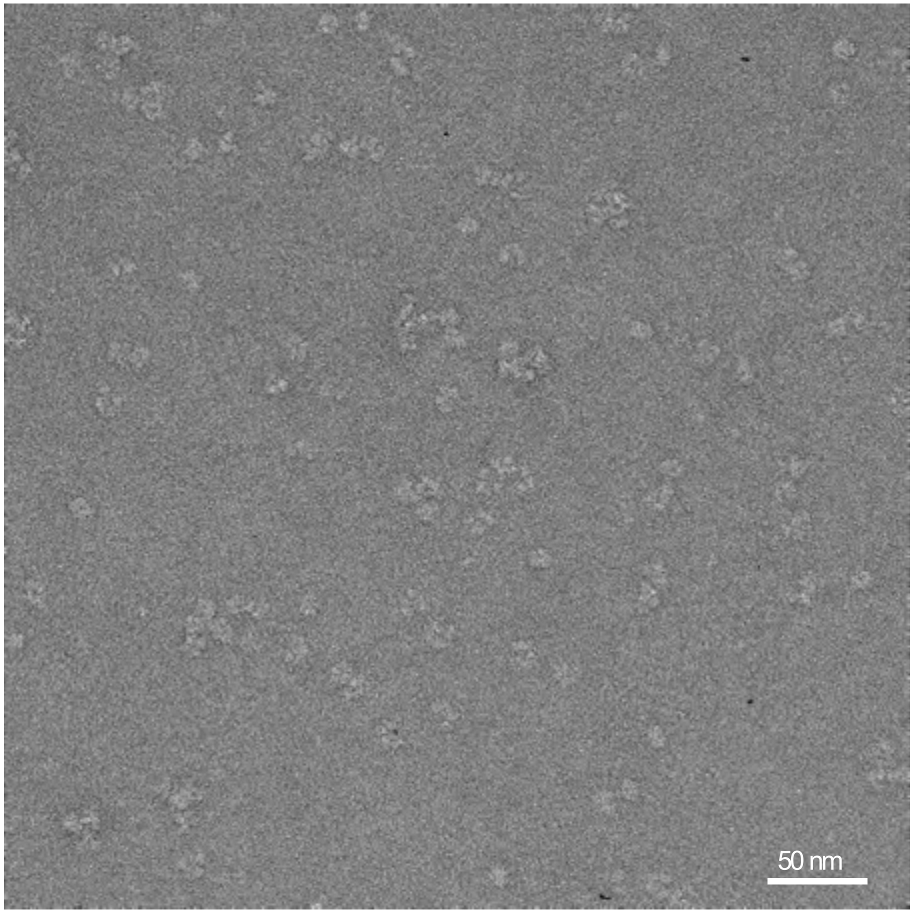

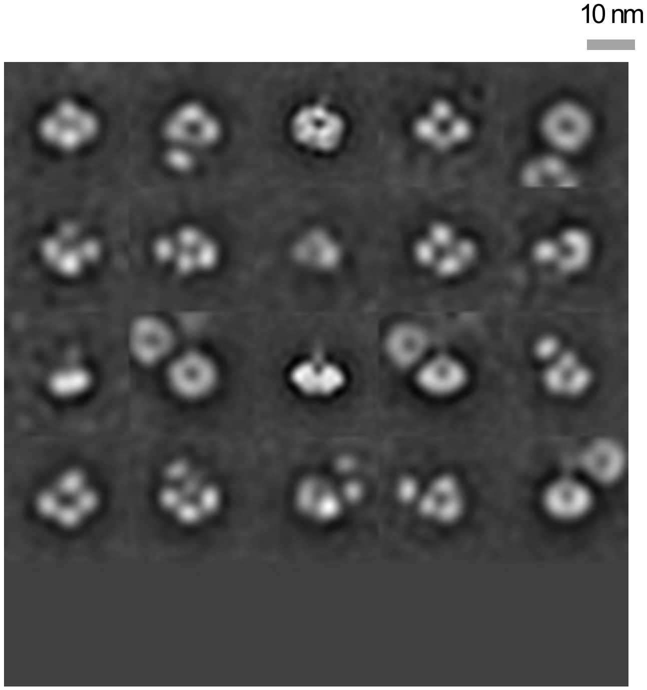

<u>Total particles picked: 3933</u>
Total particles clearly in closed state: 1413
Total particles clearly in open state: 570

N8-JD13-WT

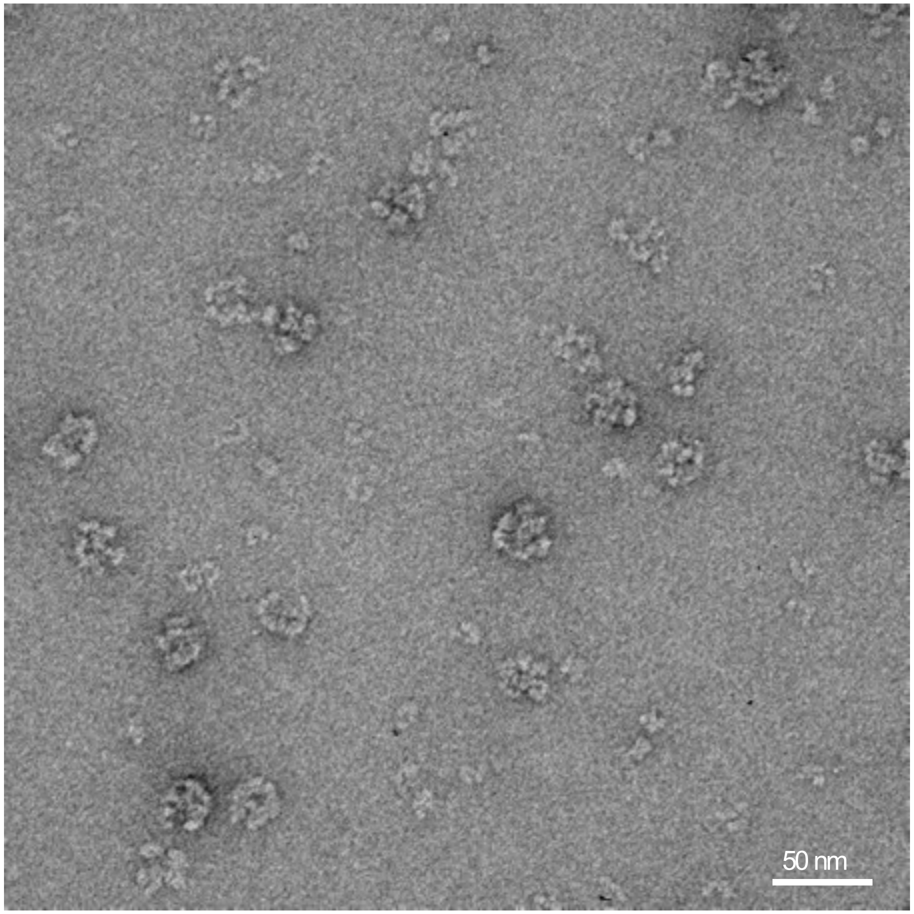

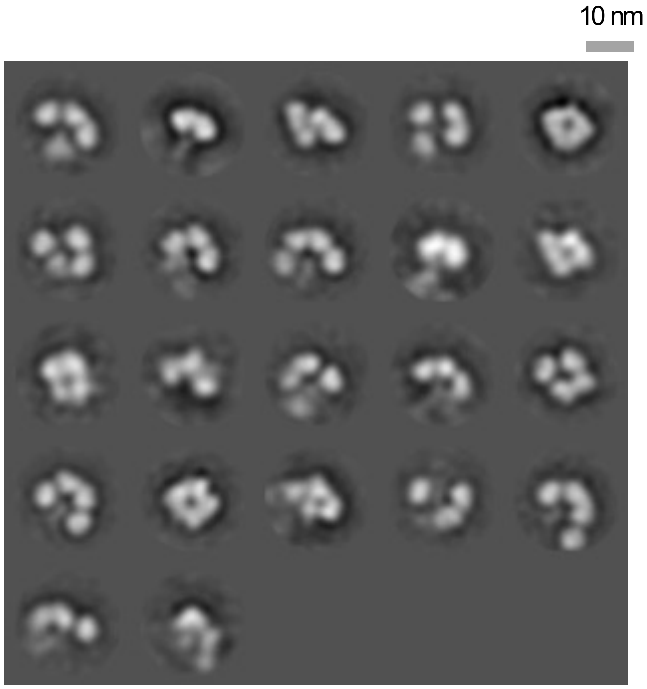

Total particles picked: 7599
Total particles clearly in closed state: 1688
Total particles clearly in open state: 3304

N9-AN13-WT

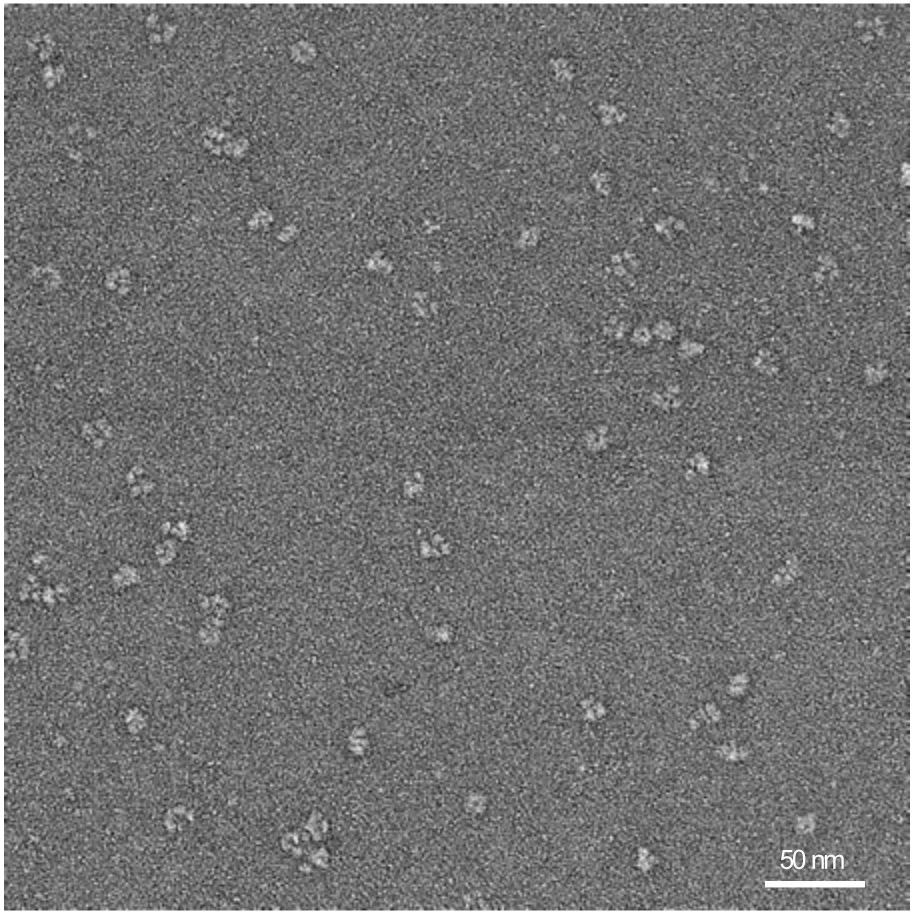

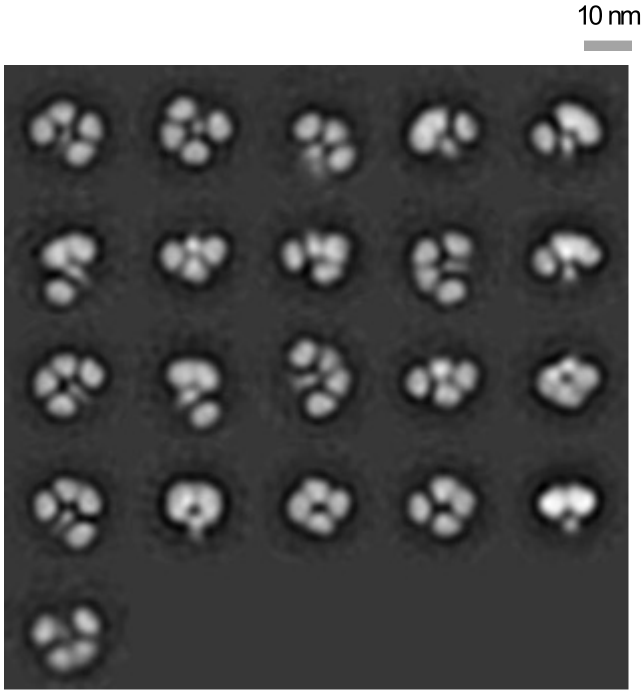

<u>Total particles picked: 8020</u>
Total particles clearly in closed state: 1435
Total particles clearly in open state: 5262

N2-WI05-WT

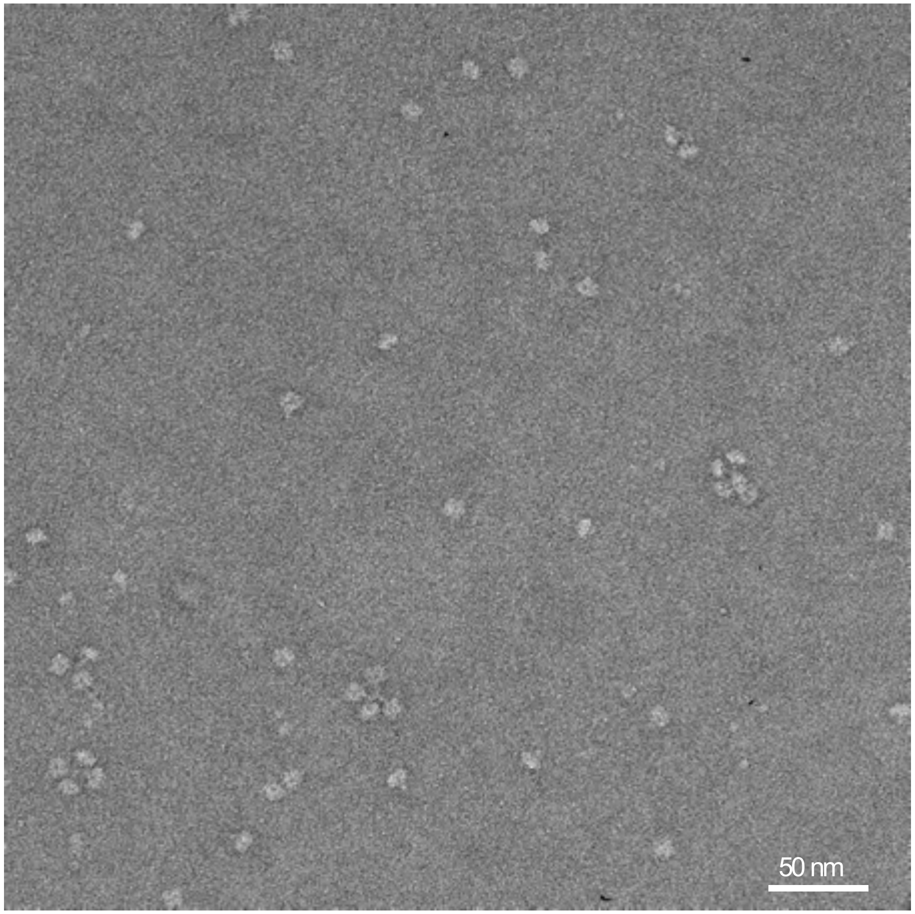

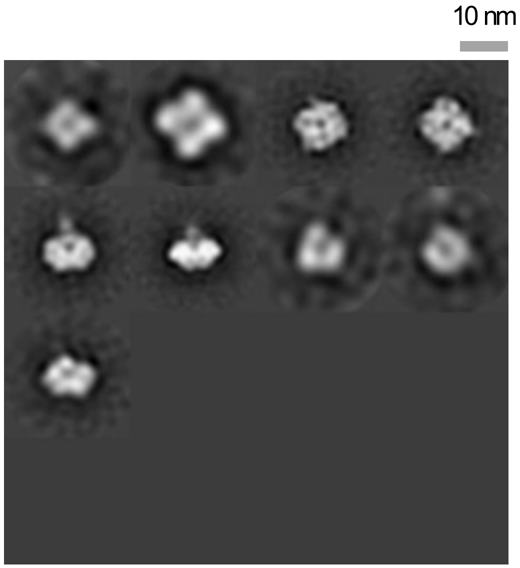

<u>Total particles picked: 2386</u>
Total particles clearly in closed state: 2117
Total particles clearly in open state: 0

N2-IN11-WT

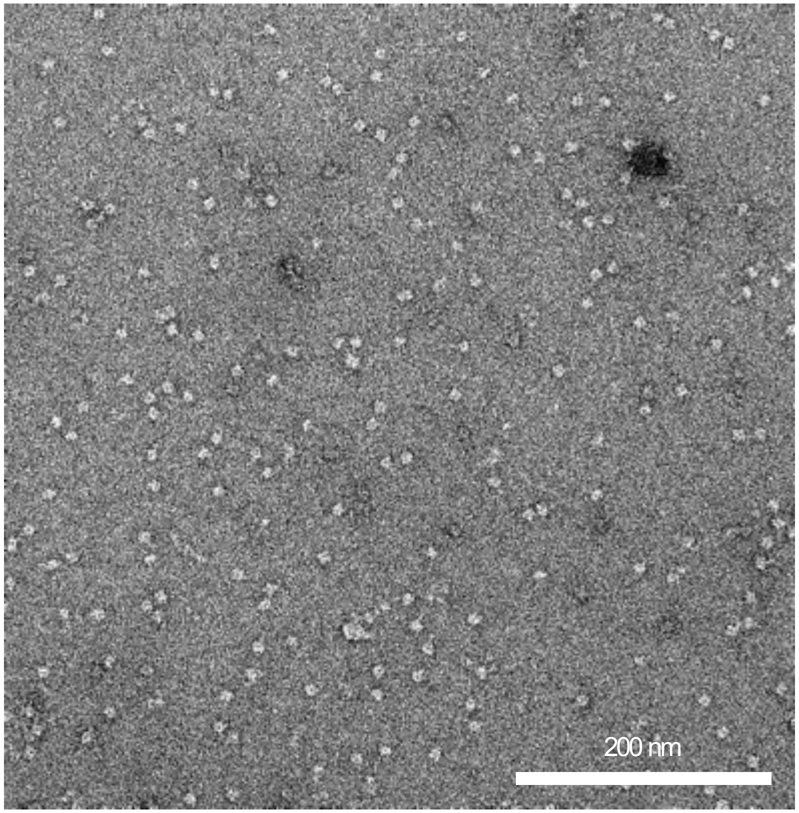

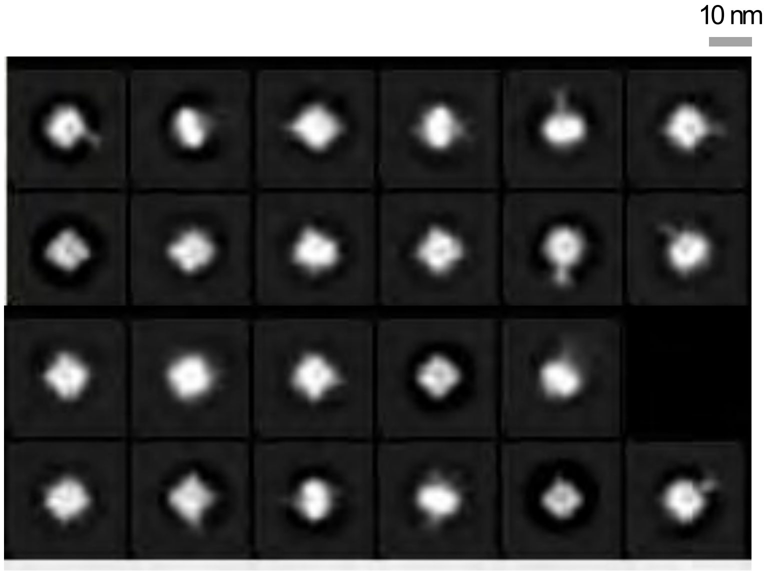

Total particles picked: 62551
Total particles clearly in closed state: 22866
Total particles clearly in open state: 0

N3-MI06-WT

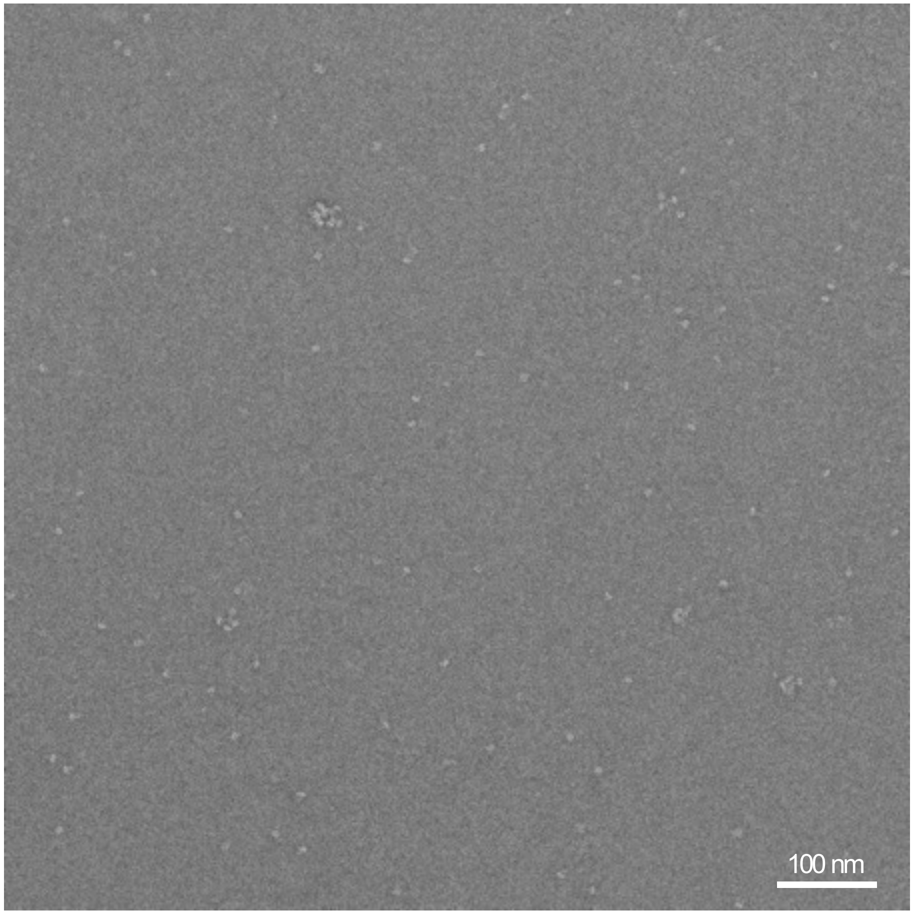

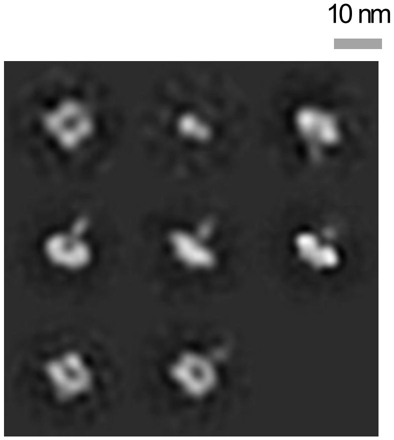

<u>Total particles picked: 6166</u>
Total particles clearly in closed state: 3477
Total particles clearly in open state: 0

N5-DB16-WT

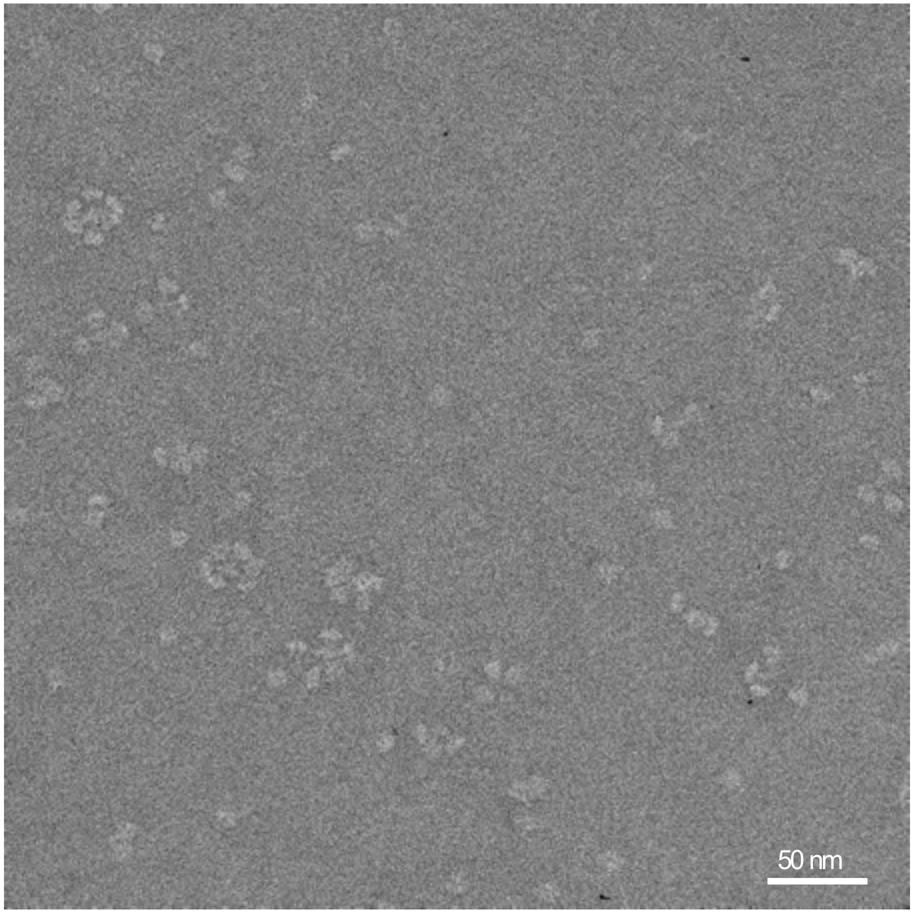

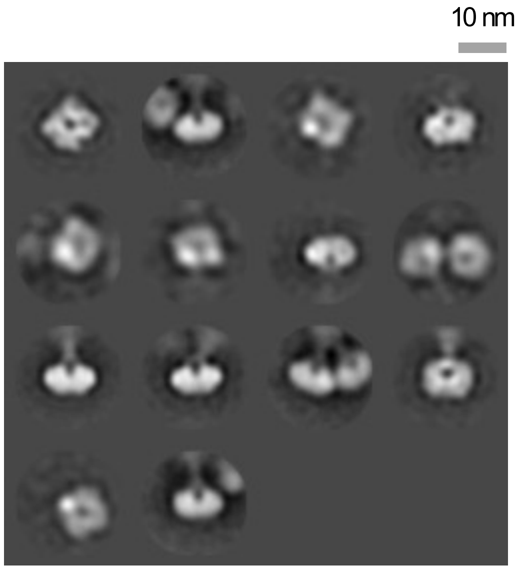

<u>Total particles picked: 8042</u>
Total particles clearly in closed state: 5576
Total particles clearly in open state: 0

B-CO17-WT

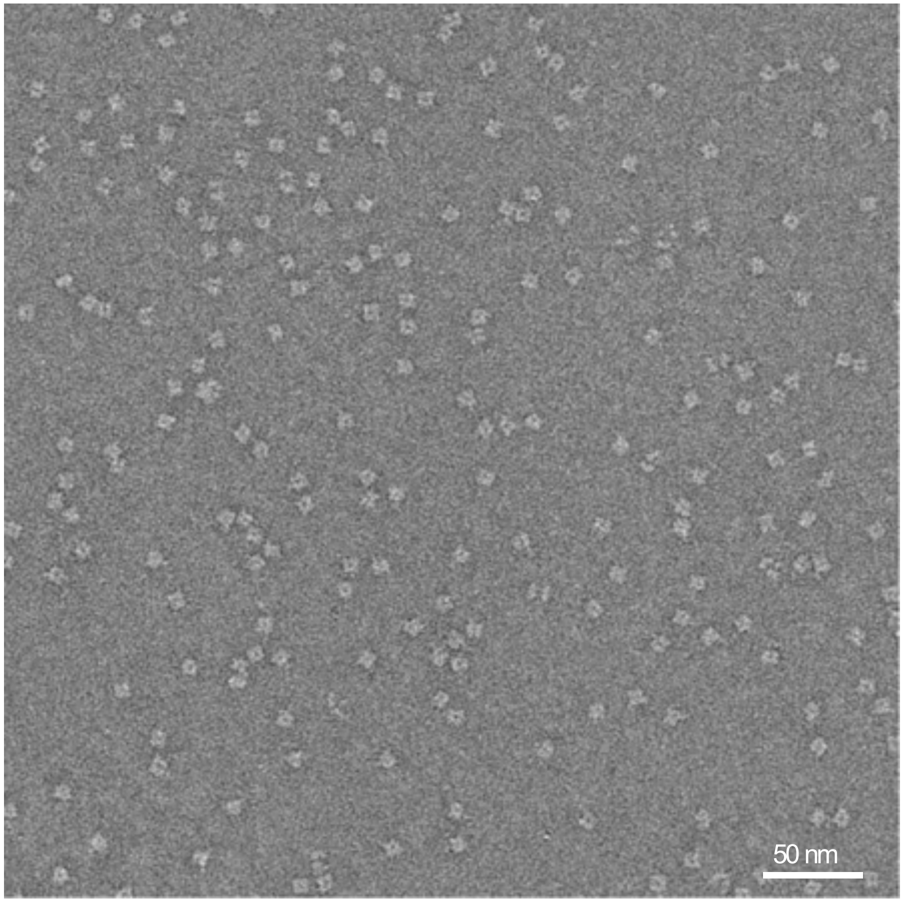

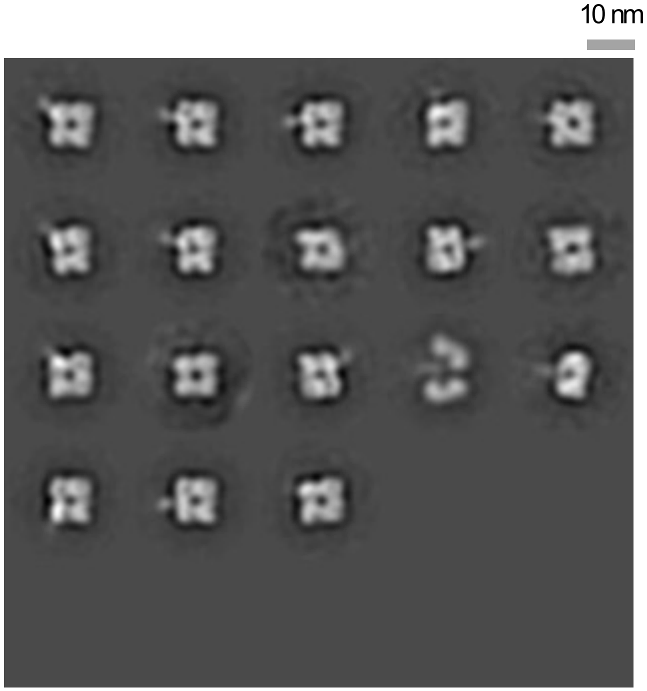

<u>Total particles picked: 12896</u>
Total particles clearly in closed state: 12328
Total particles clearly in open state: 425

B-PH13-WT

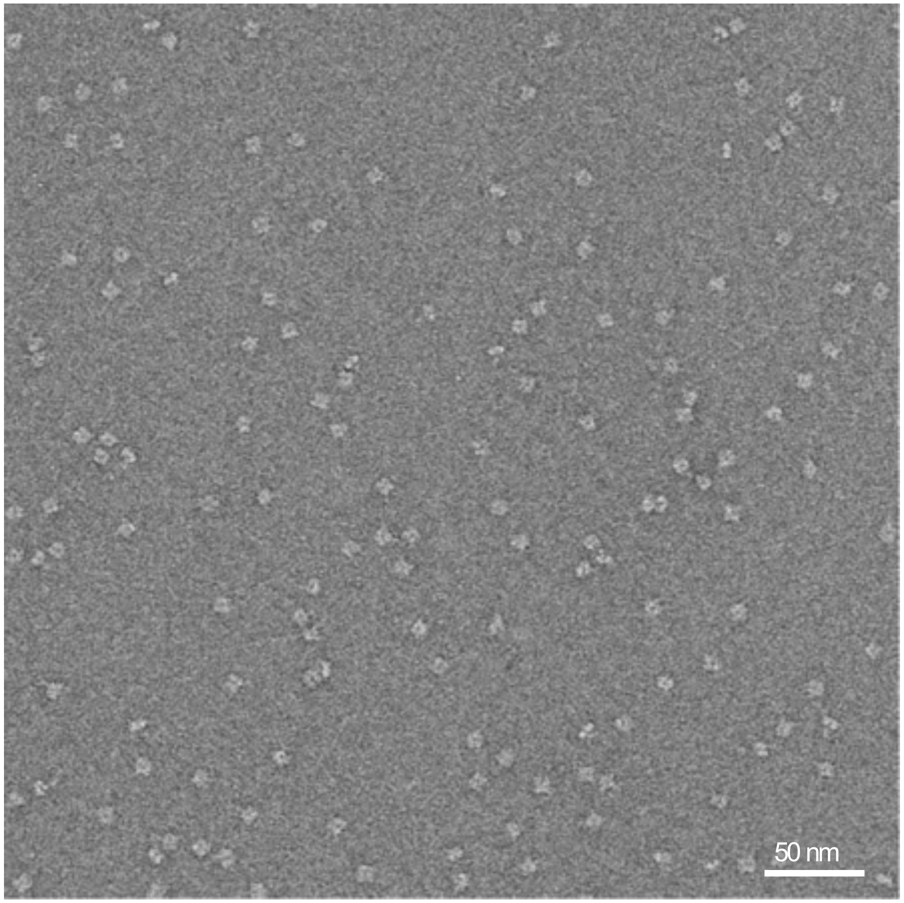

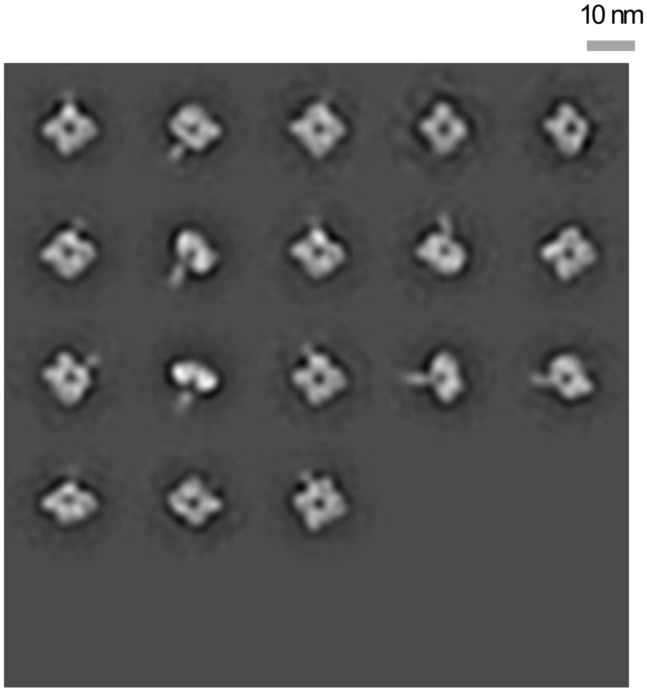

<u>Total particles picked: 9972</u>

Total particles clearly in closed state: 9817

Total particles clearly in open state: 0

N9-AN13-Y170H

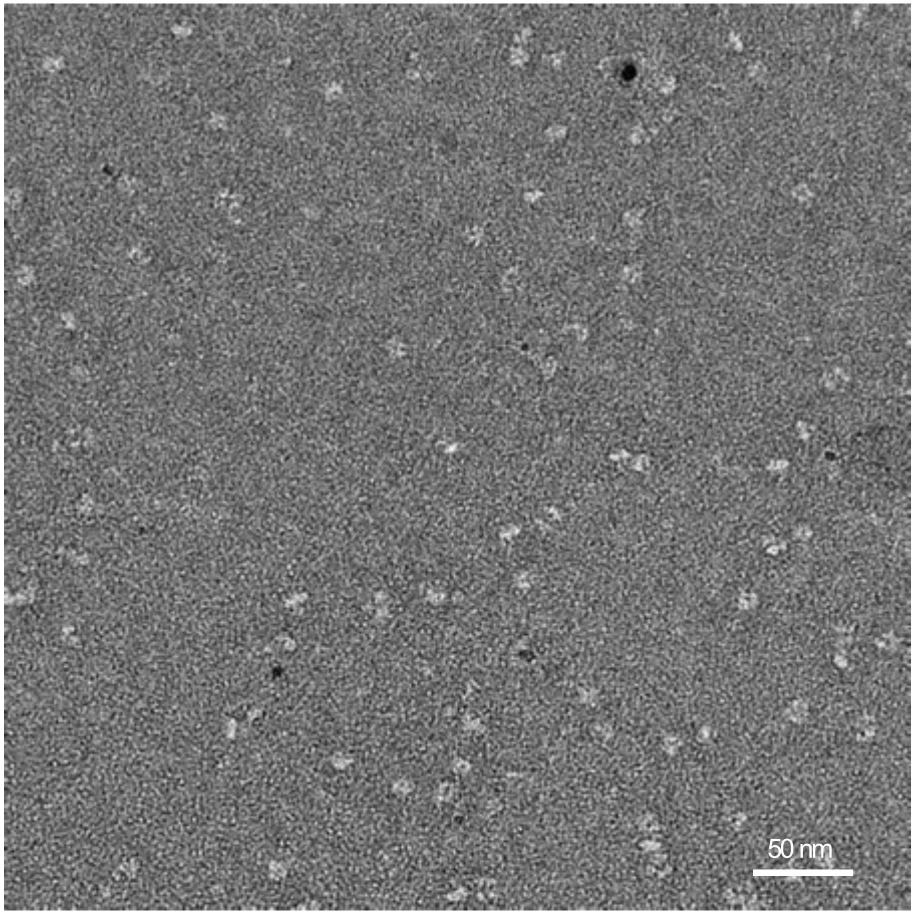

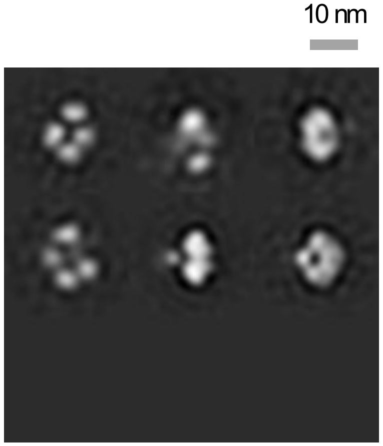

Total particles picked: 8592
Total particles clearly in closed state: 3059
Total particles clearly in open state: 1164

N2-WI05-WT, obtained from BEI Resources

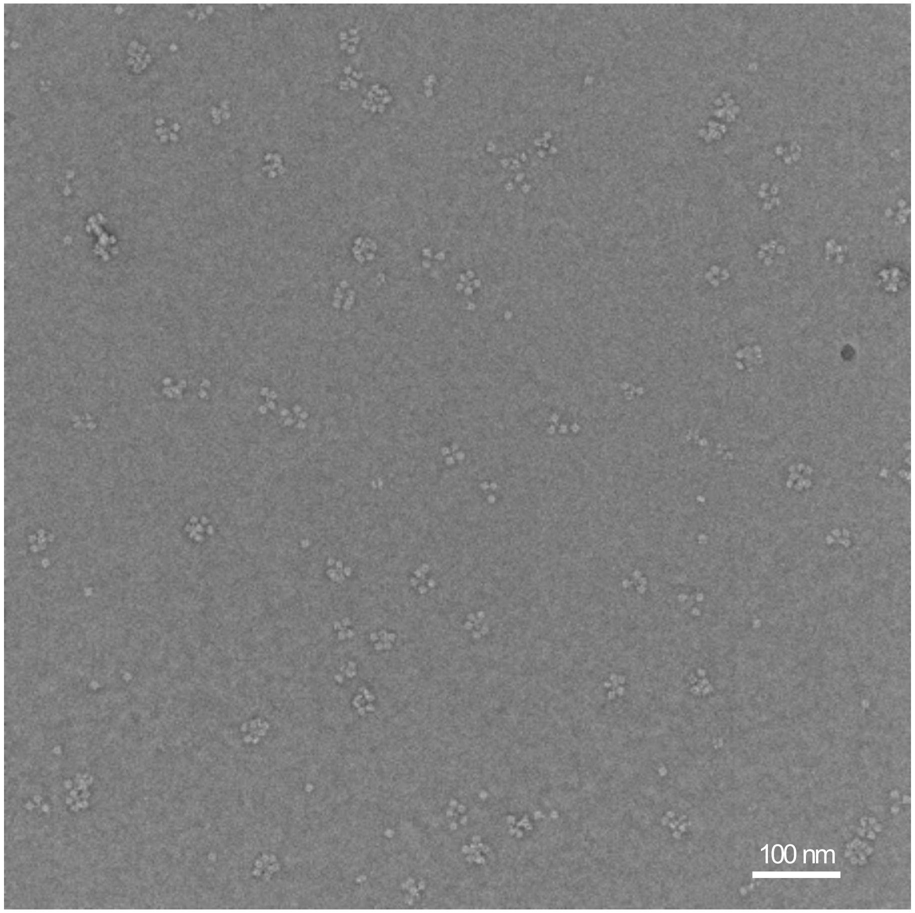

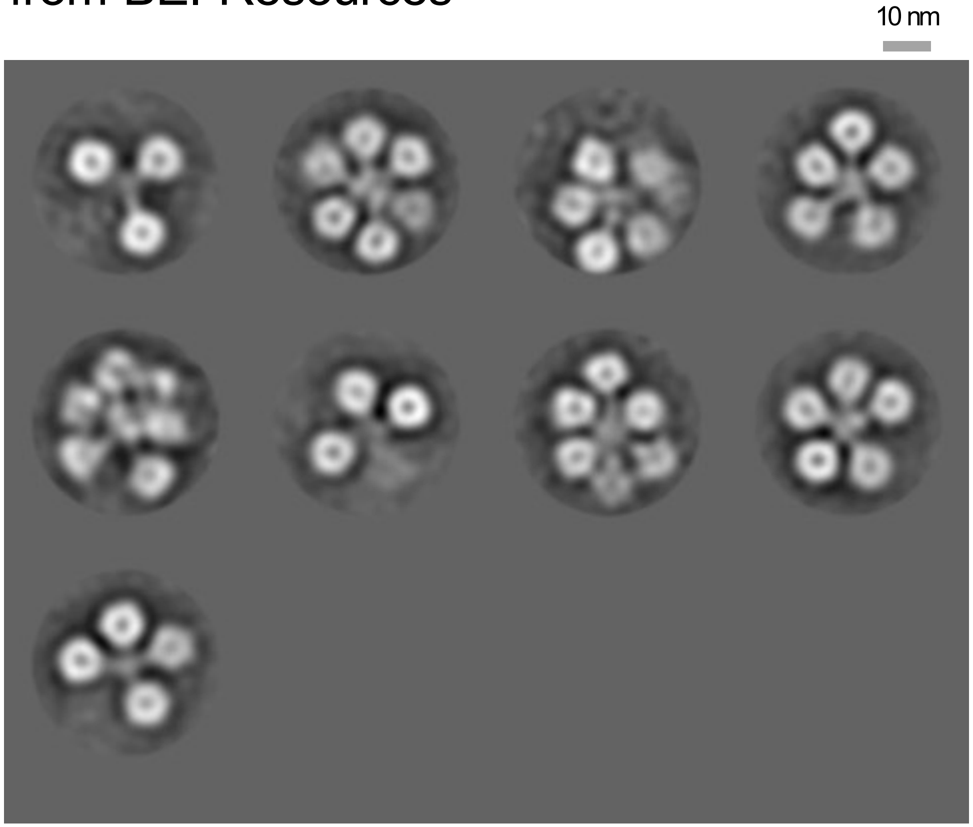

<u>Total particles picked: 1190</u>

No tetramers quantified alone, however tetramers in clusters appear predominantly closed

N1-NC99-WT, obtained from BEI Resources

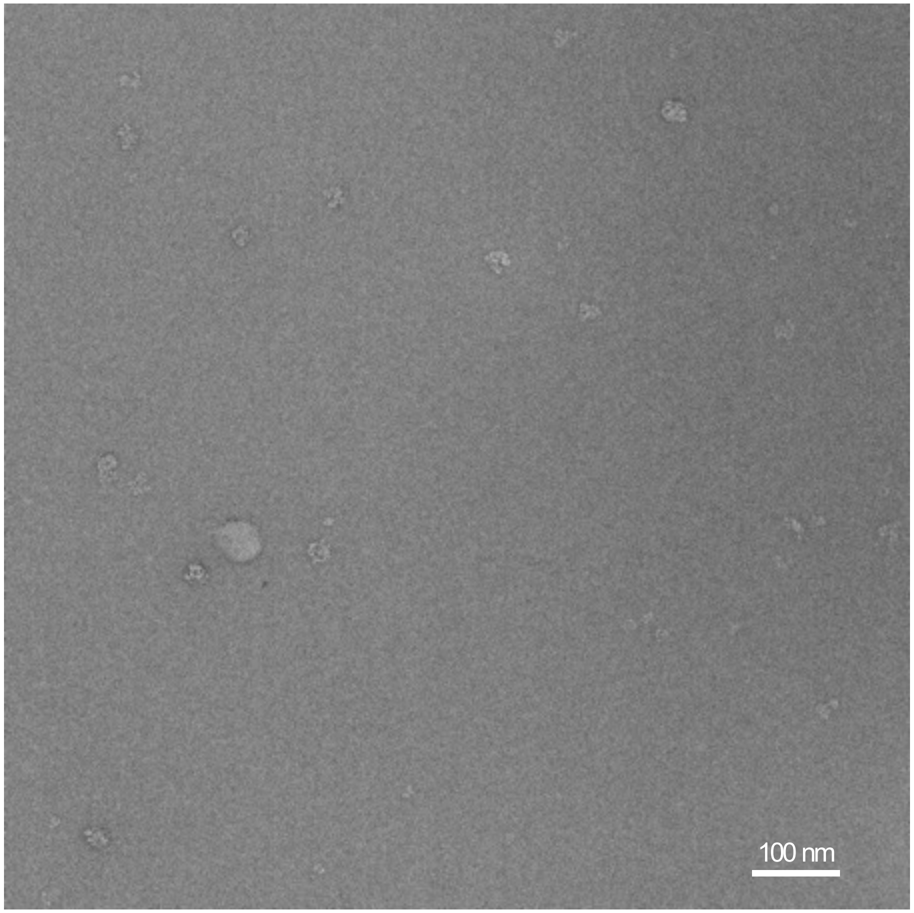

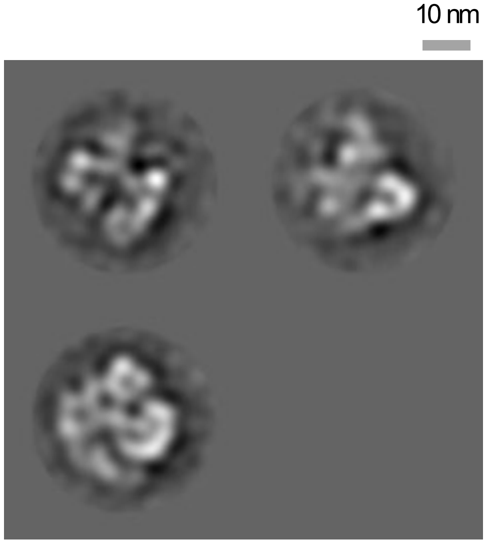

<u>Total particles picked: 1325</u>

No tetramers quantified alone, however no clear tetramers were present in clusters

N1-CA09-WT, obtained from BEI Resources

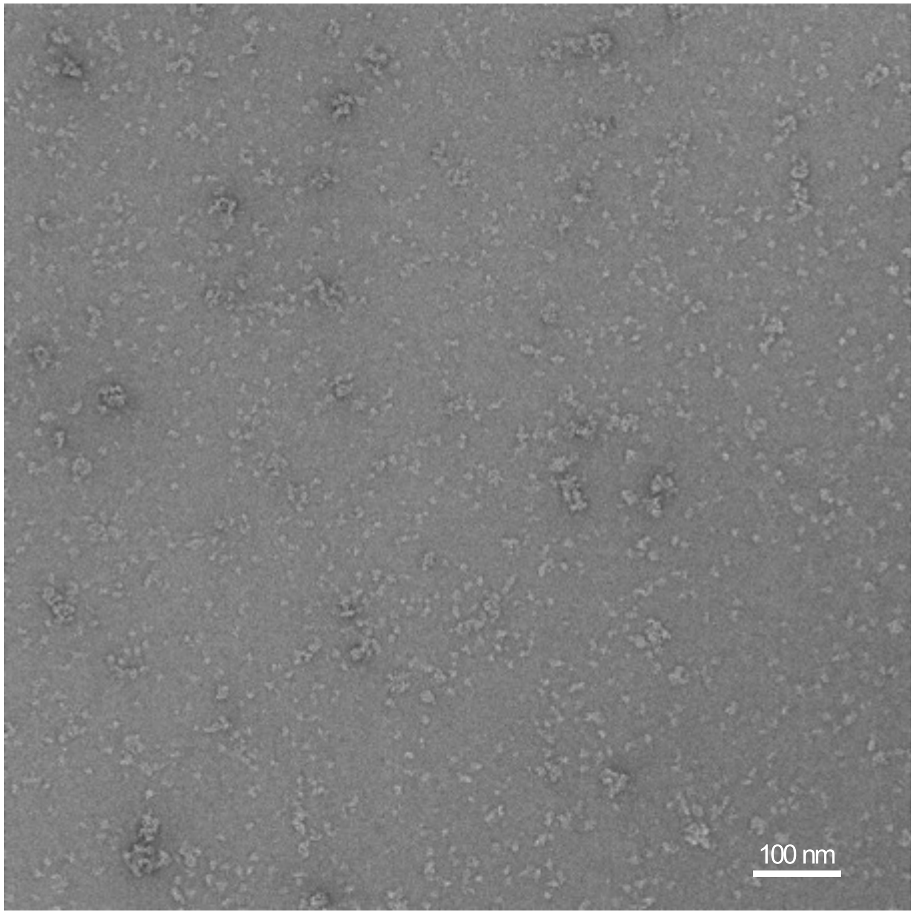

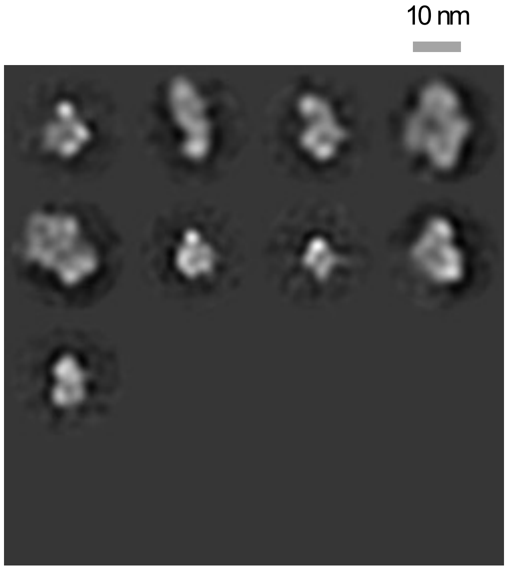

Total particles picked: 6382
No clear tetramers observed to quantify

N1-CA09-WT with 1mM $CaCl_2$ added after purification

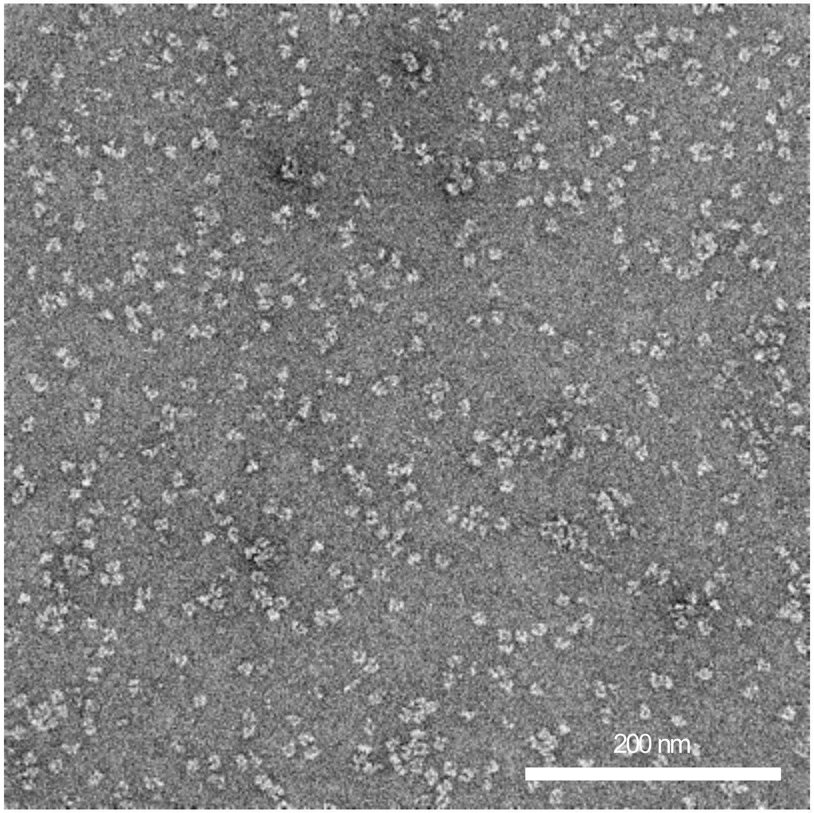

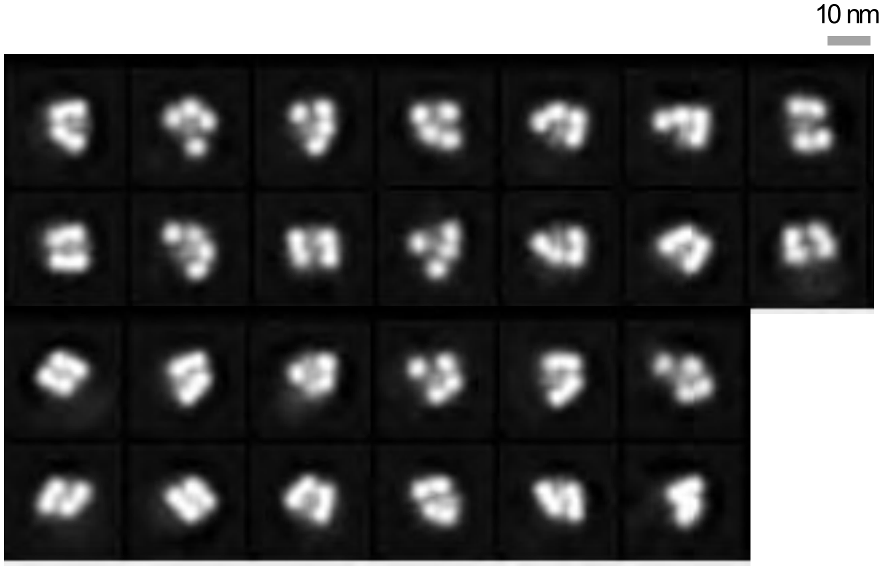

<u>Total particles picked: 56046</u>
Total particles clearly in closed state: 738
Total particles clearly in open state: 46410

N1-CA09-WT with 1mM $CaCl_2$ added throughout purification process

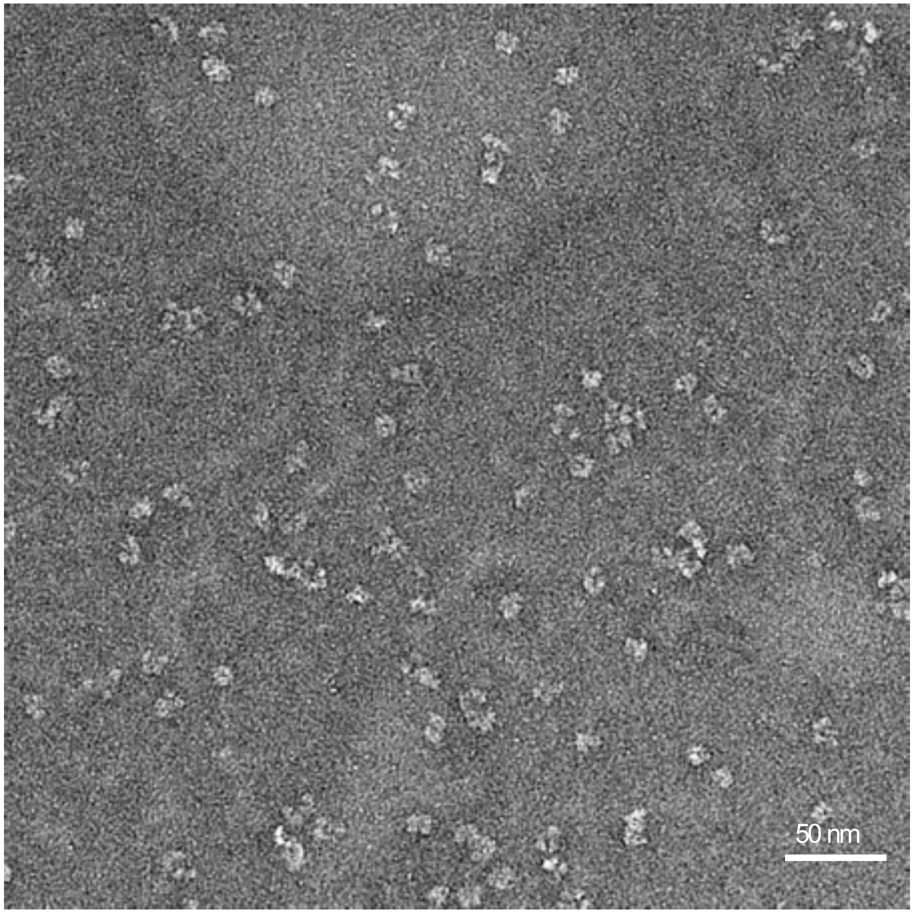

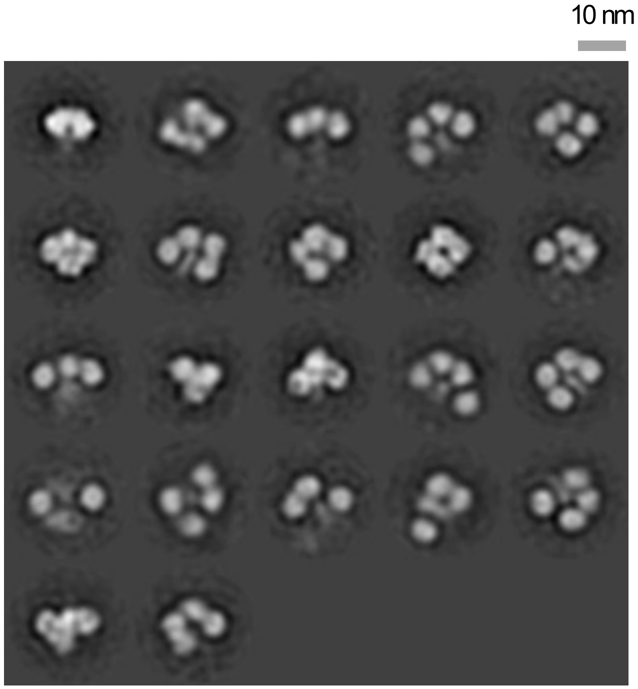

<u>Total particles picked: 6847</u>

Total particles clearly in closed state: 1051

Total particles clearly in open state: 4600

N1-MI15-WT with 1mM $CaCl_2$ added throughout purification process

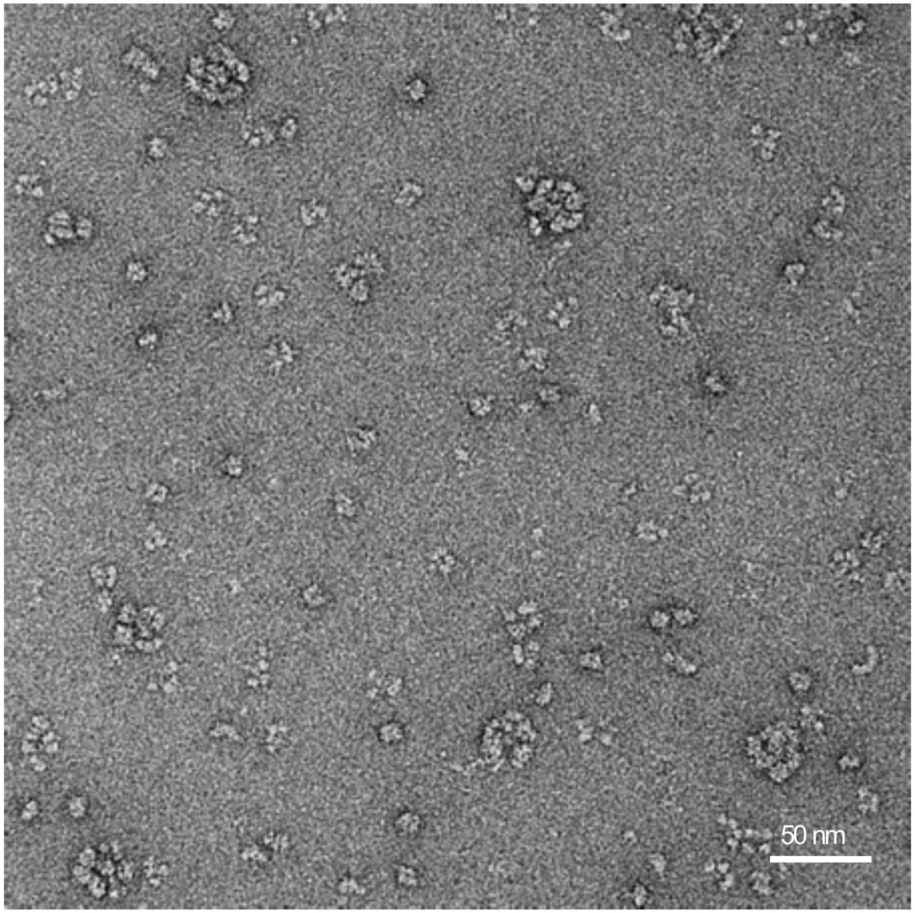

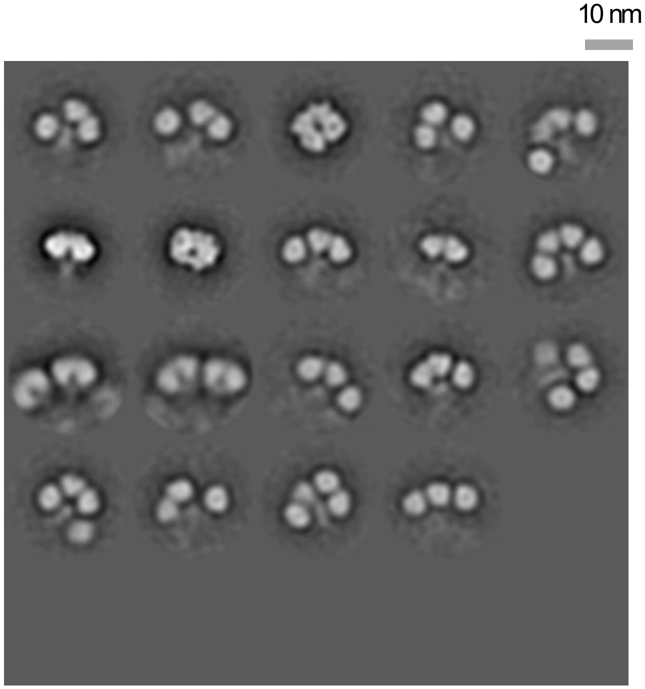

Total particles picked: 7756
Total particles clearly in closed state: 1760
Total particles clearly in open state: 4127

N1-CA09-WT + Stalk

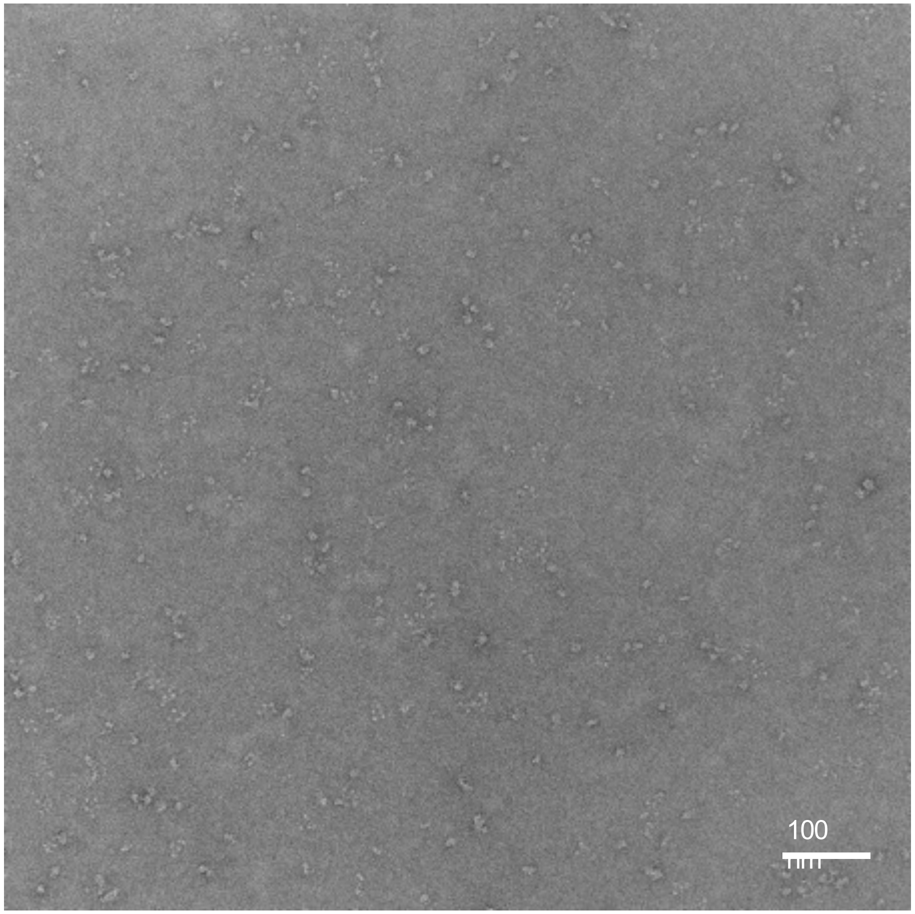

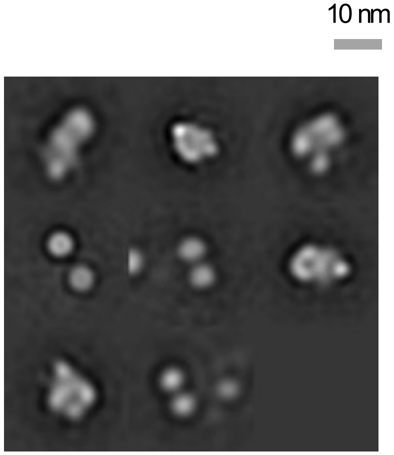

<u>Total particles picked: 8619</u>
Total particles clearly in closed state: 0
Total particles clearly in open state: 0
Classes appeared largely disordered

N1-CA09-WT + Stalk with 1mM $CaCl_2$ added throughout purification process

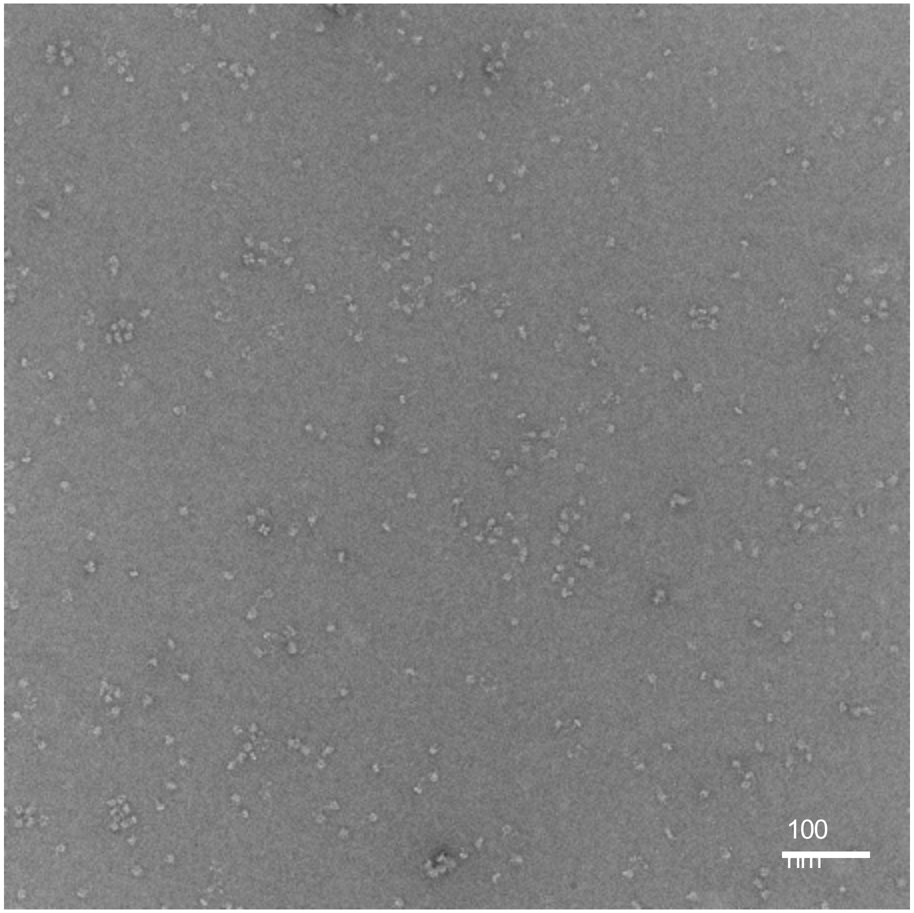

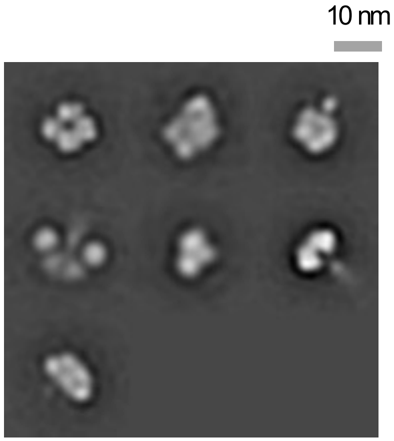

<u>Total particles picked: 9097</u>
Total particles clearly in closed state: 3335
Total particles clearly in open state: 389
Many particles appeared otherwise disordered

N1-CA09-sNAp-94

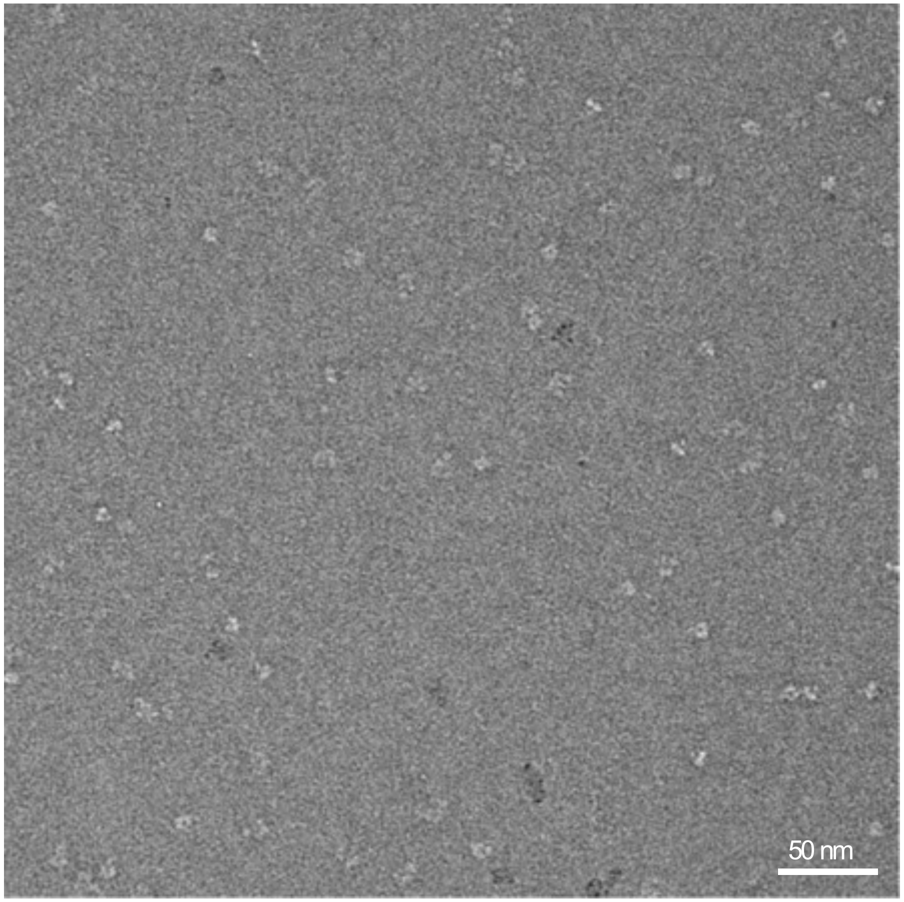

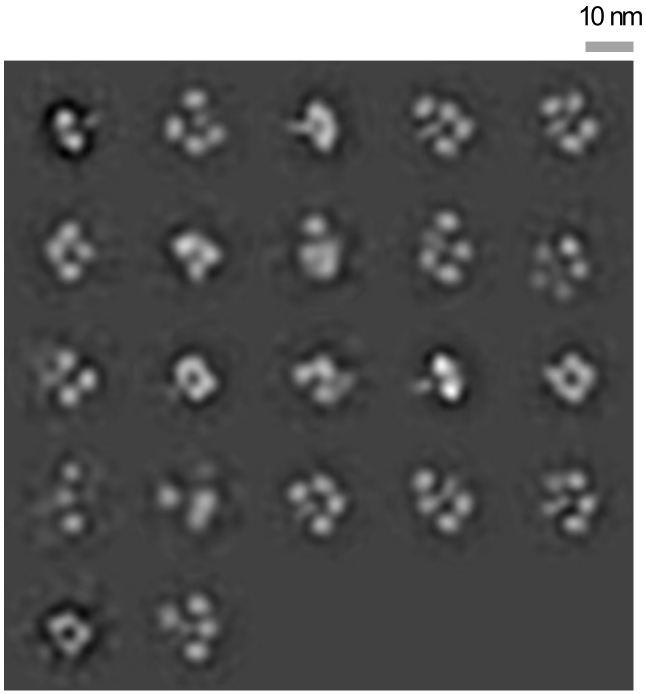

Total particles picked: 6596

Total particles clearly in closed state: 2471

Total particles clearly in open state: 2585

N1-CA09-sNAp-114

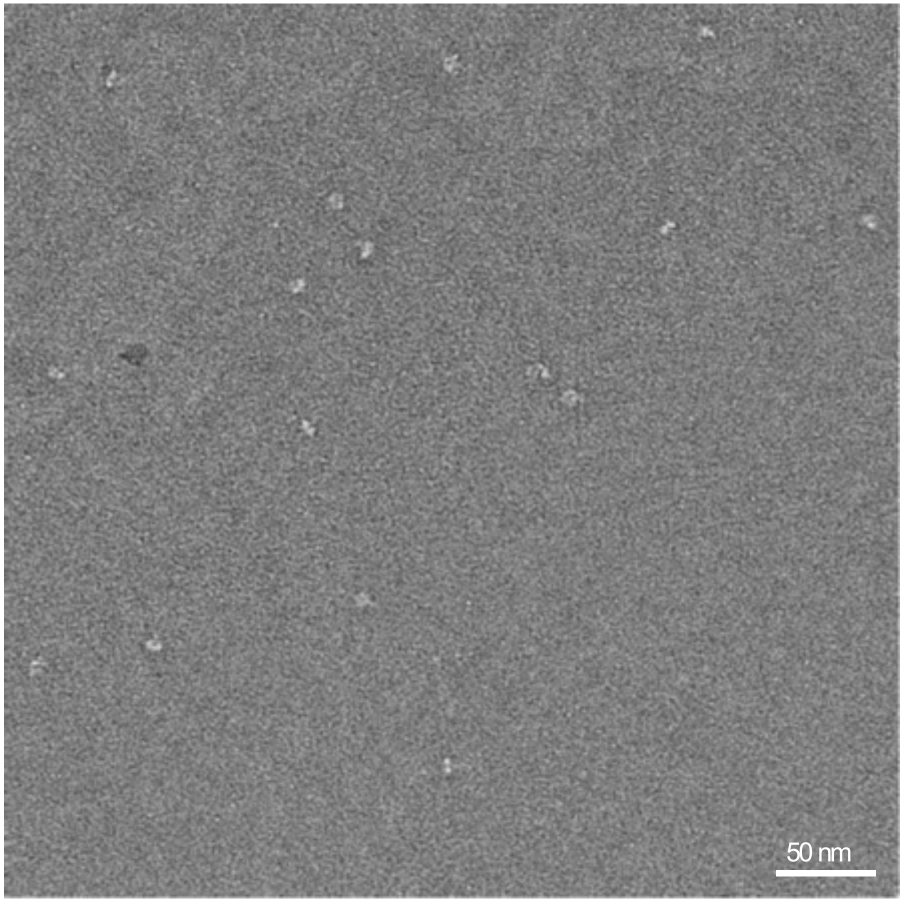

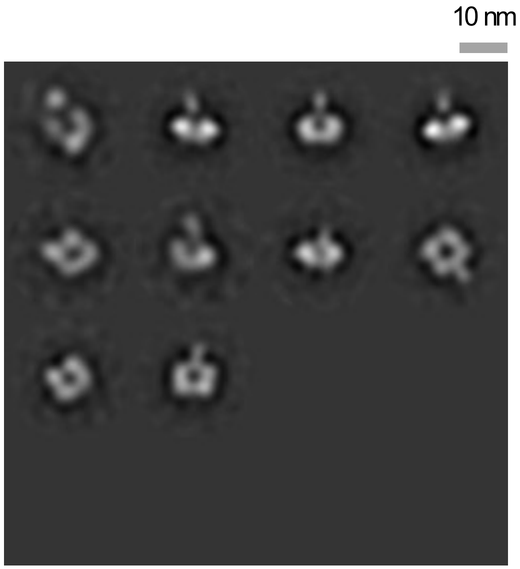

<u>Total particles picked: 3155</u>
Total particles clearly in closed state: 1647
Total particles clearly in open state: 41

N1-CA09-sNAp-130

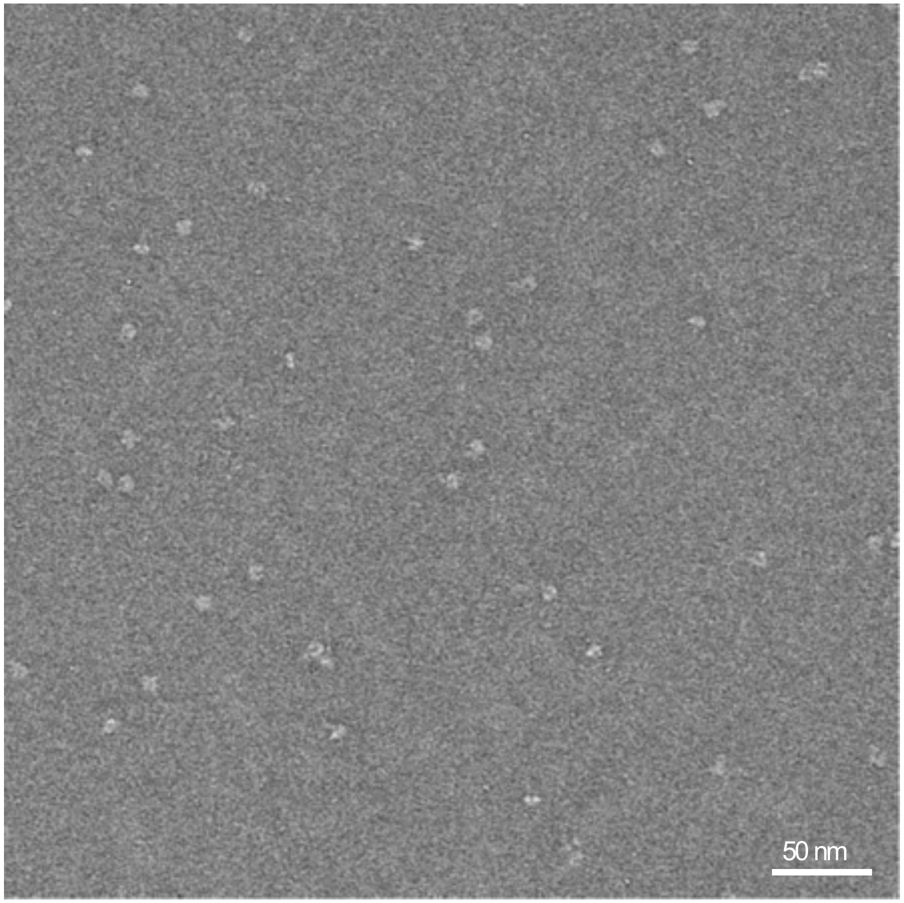

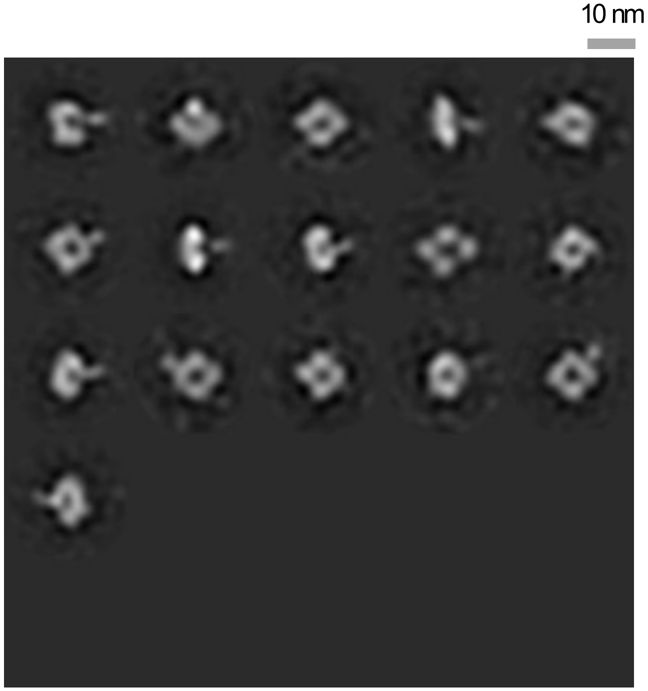

Total particles picked: 4334
Total particles clearly in closed state: 3152
Total particles clearly in open state: 0

N1-CA09-sNAp-155

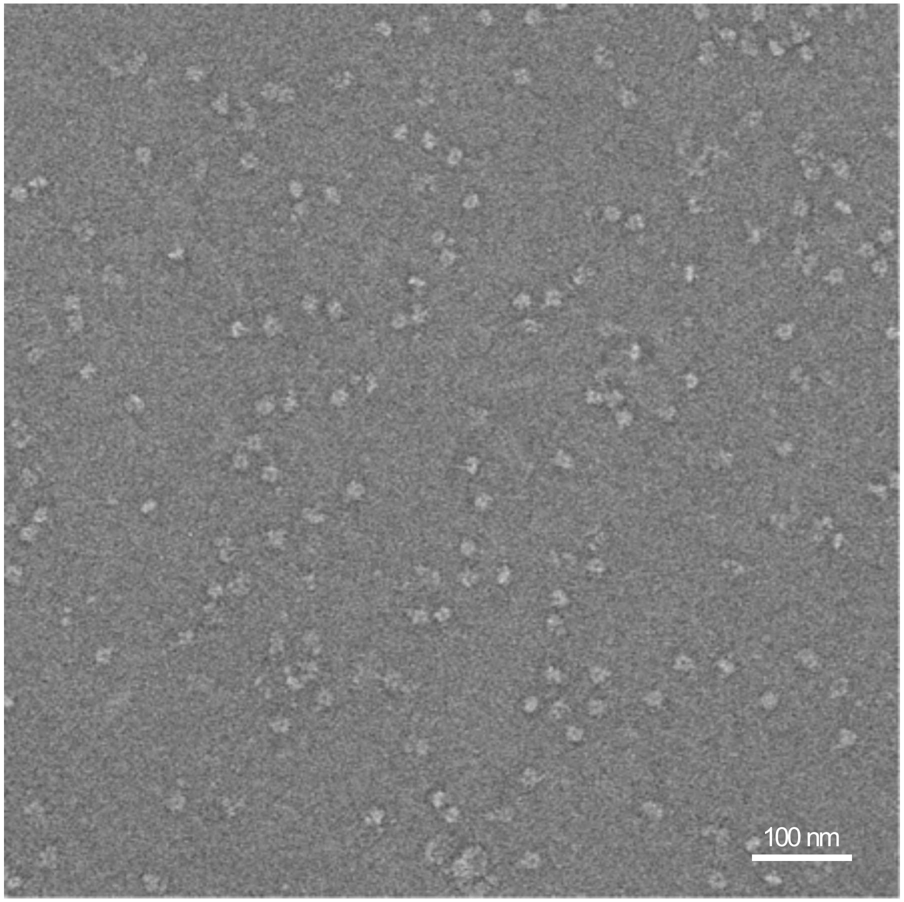

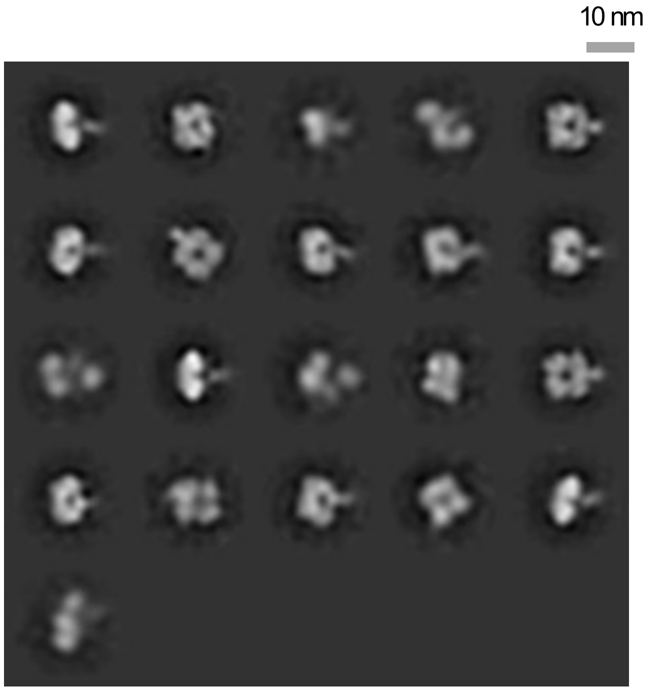

Total particles picked: 9724
Total particles clearly in closed state: 7603
Total particles clearly in open state: 1152

N1-CA09-sNAp-155, time course study - Day 0

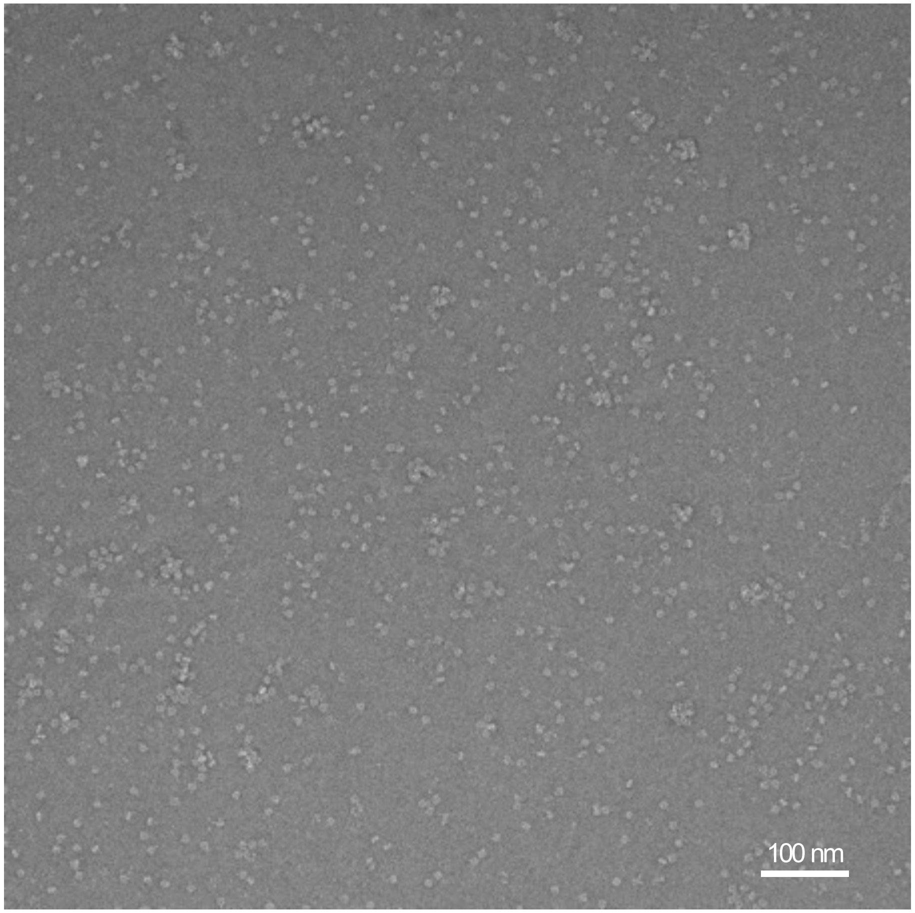

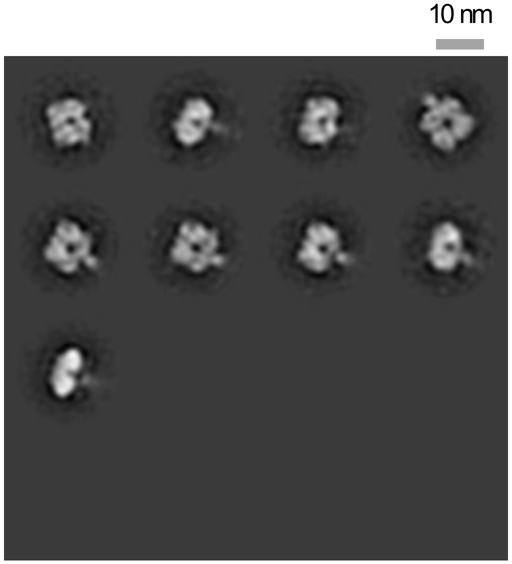

<u>Total particles picked: 5727</u>

Total particles clearly in closed state: 4853

Total particles clearly in open state: 0

N1-CA09-sNAp-155, time course study - Day 6

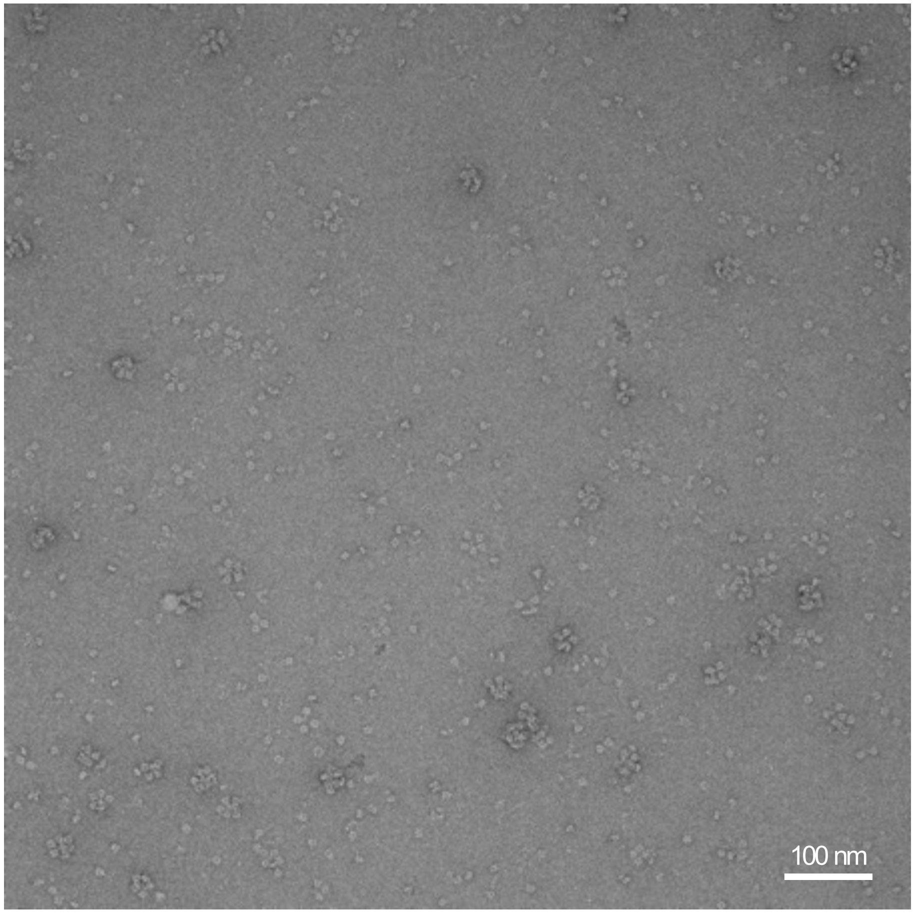

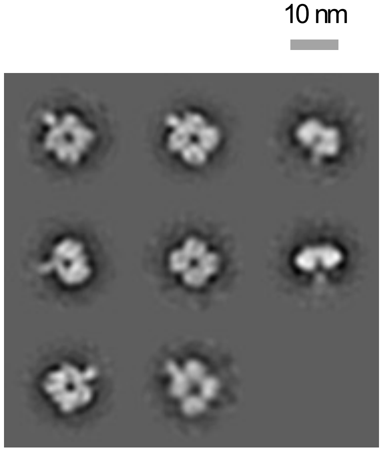

<u>Total particles picked: 4503</u>
Total particles clearly in closed state: 3205
Total particles clearly in open state: 0

N1-CA09-sNAp-155, time course study - Day 10

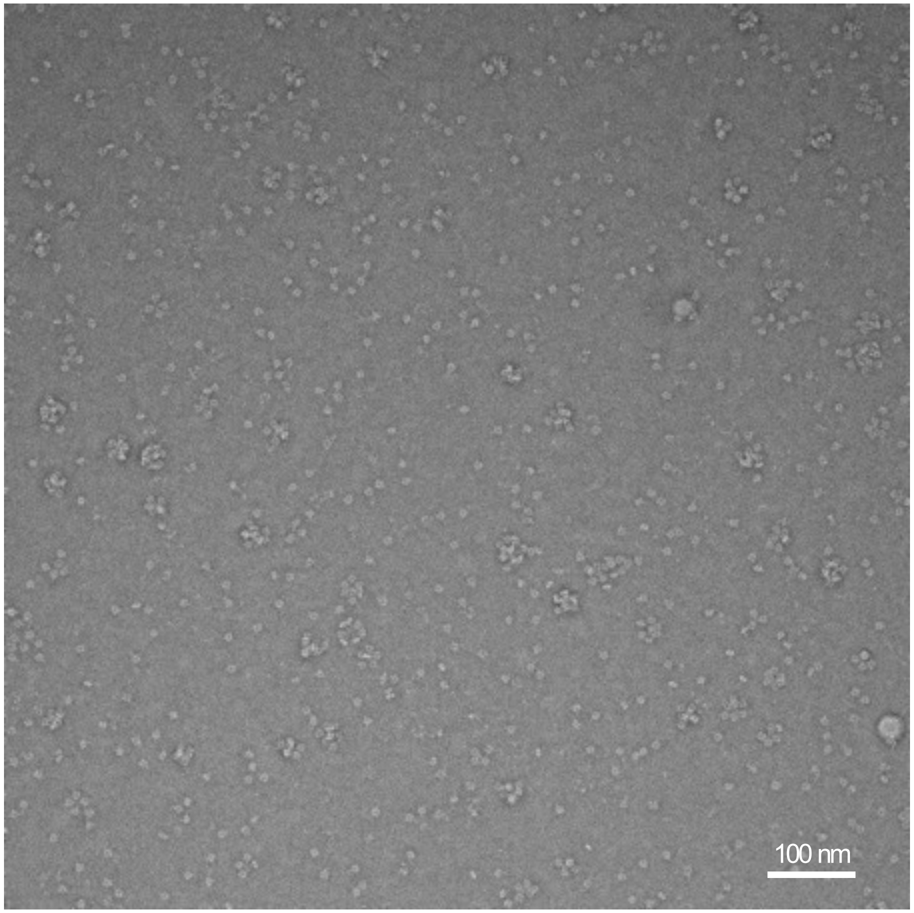

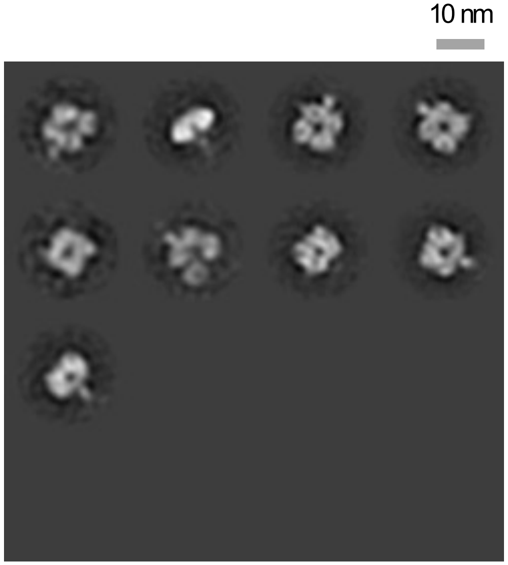

Total particles picked: 5179

Total particles clearly in closed state: 4010

Total particles clearly in open state: 0

N1-CA09-sNAp-155, time course study - Day 15

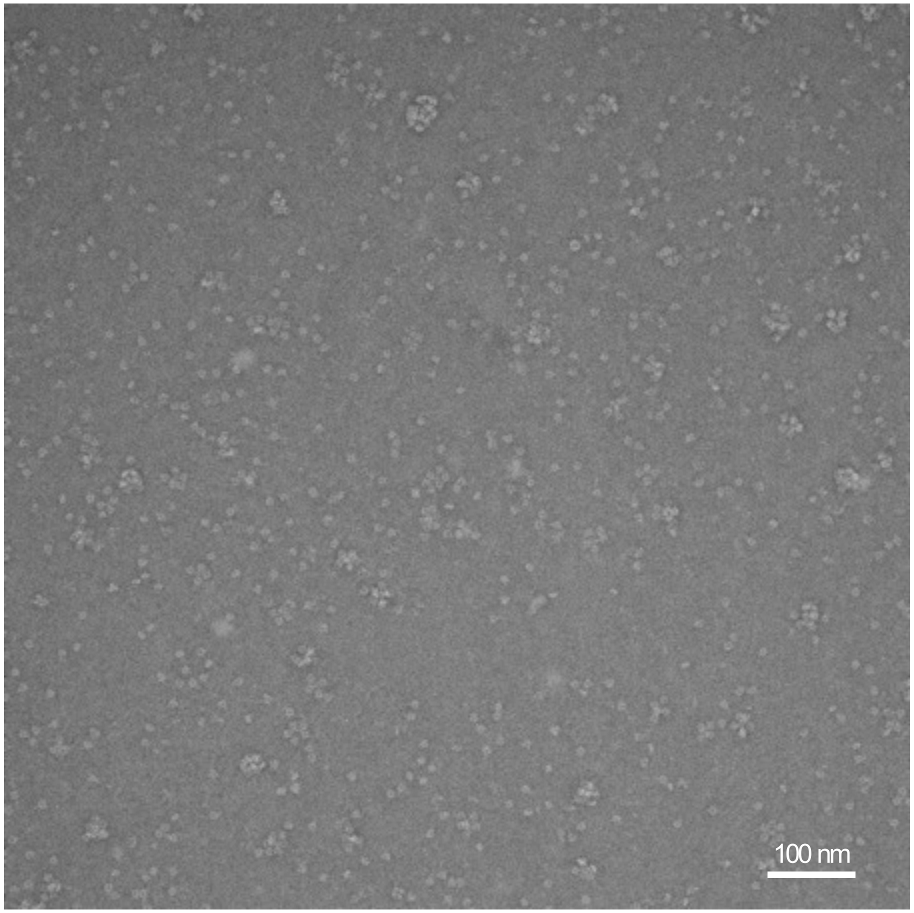

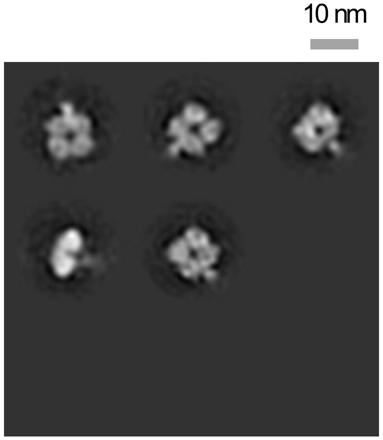

Total particles picked: 6941
Total particles clearly in closed state: 4670
Total particles clearly in open state: 0

N1-CA09-sNAp-131

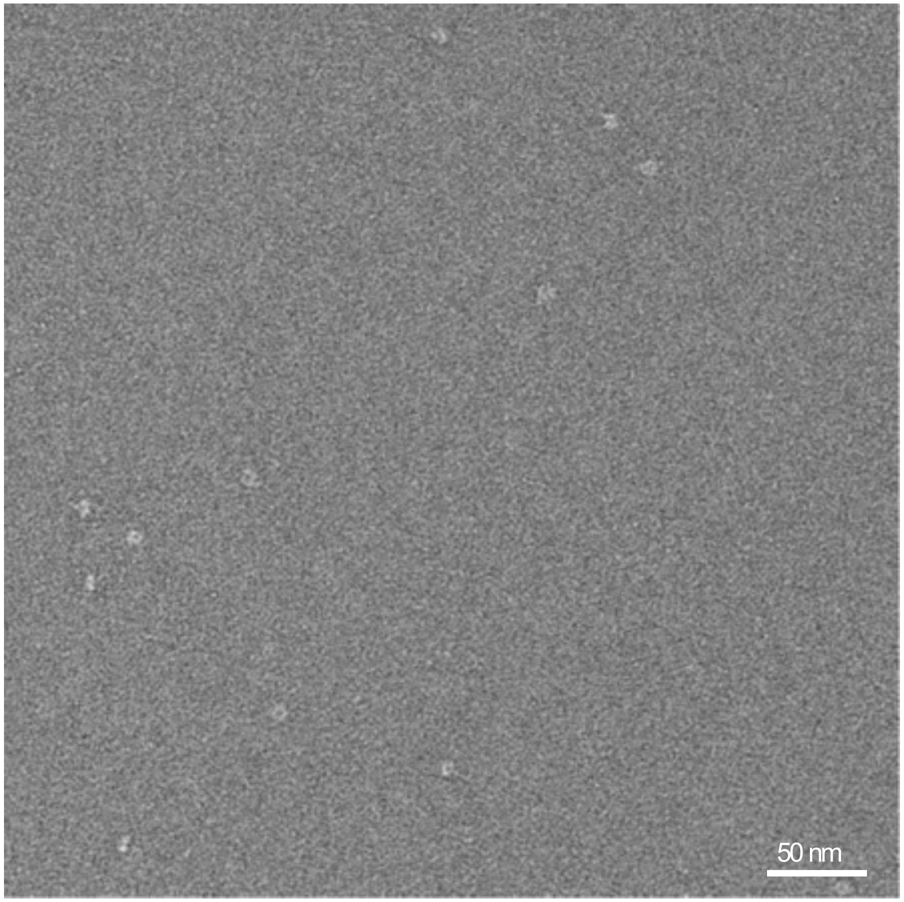

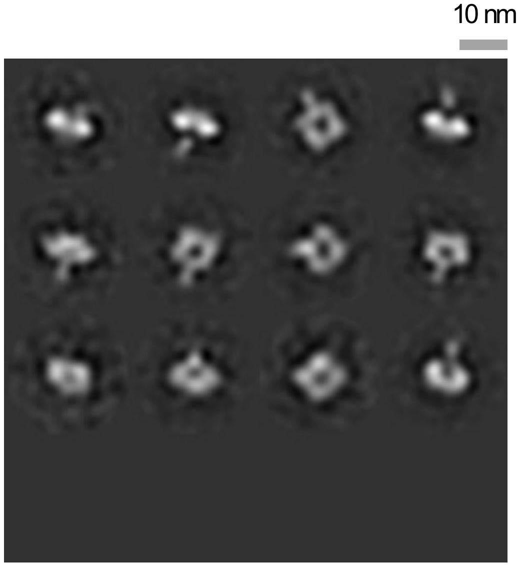

<u>Total particles picked: 2004</u>
Total particles clearly in closed state: 1508
Total particles clearly in open state: 0

N1-CA09-sNAp-134

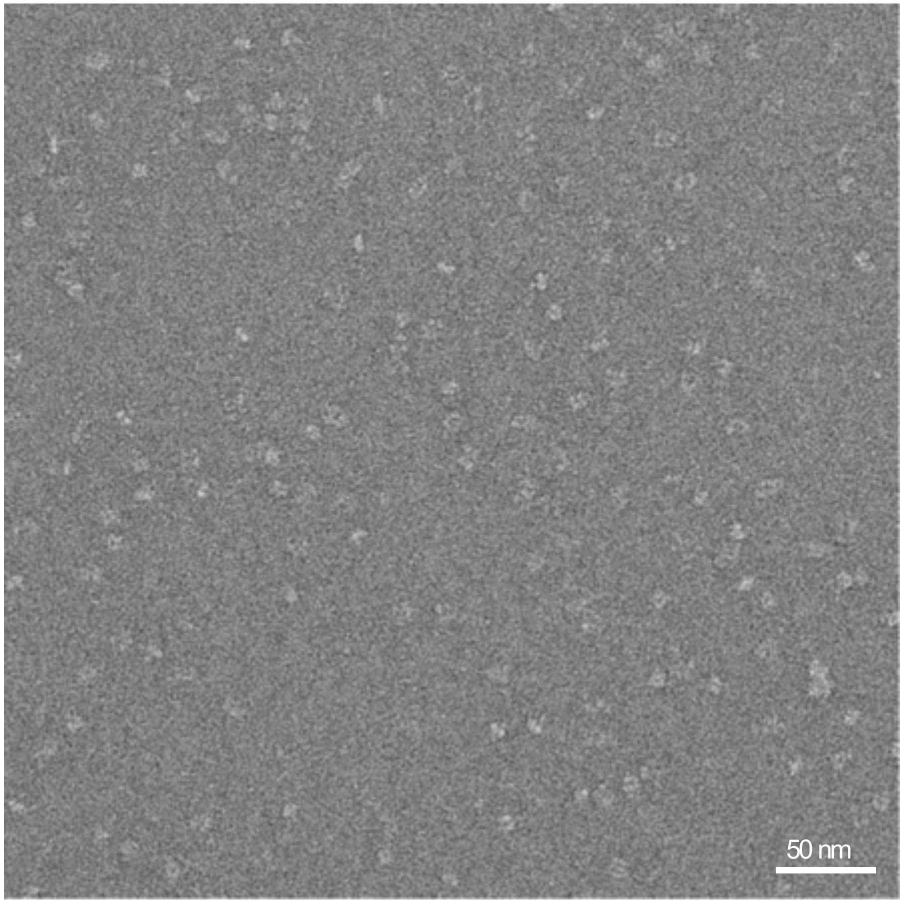

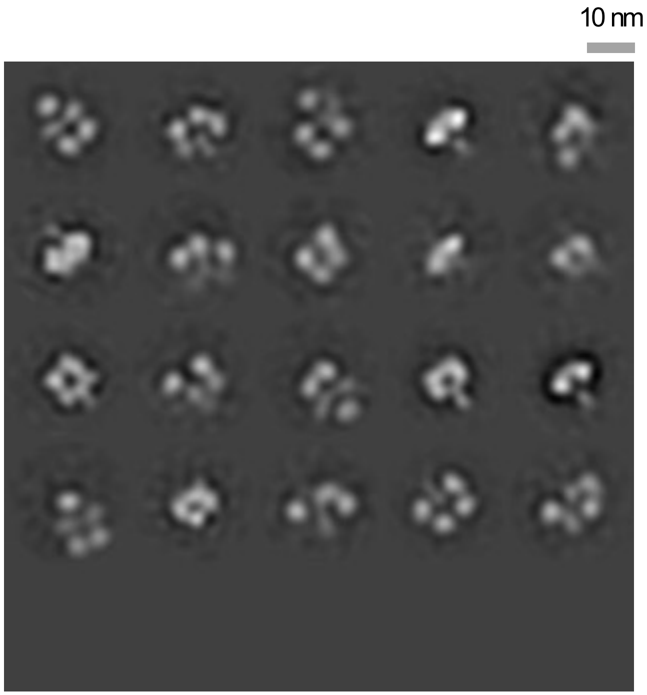

Total particles picked: 16073
Total particles clearly in closed state: 5389
Total particles clearly in open state: 4932

N1-CA09-sNAp-130 + Stalk

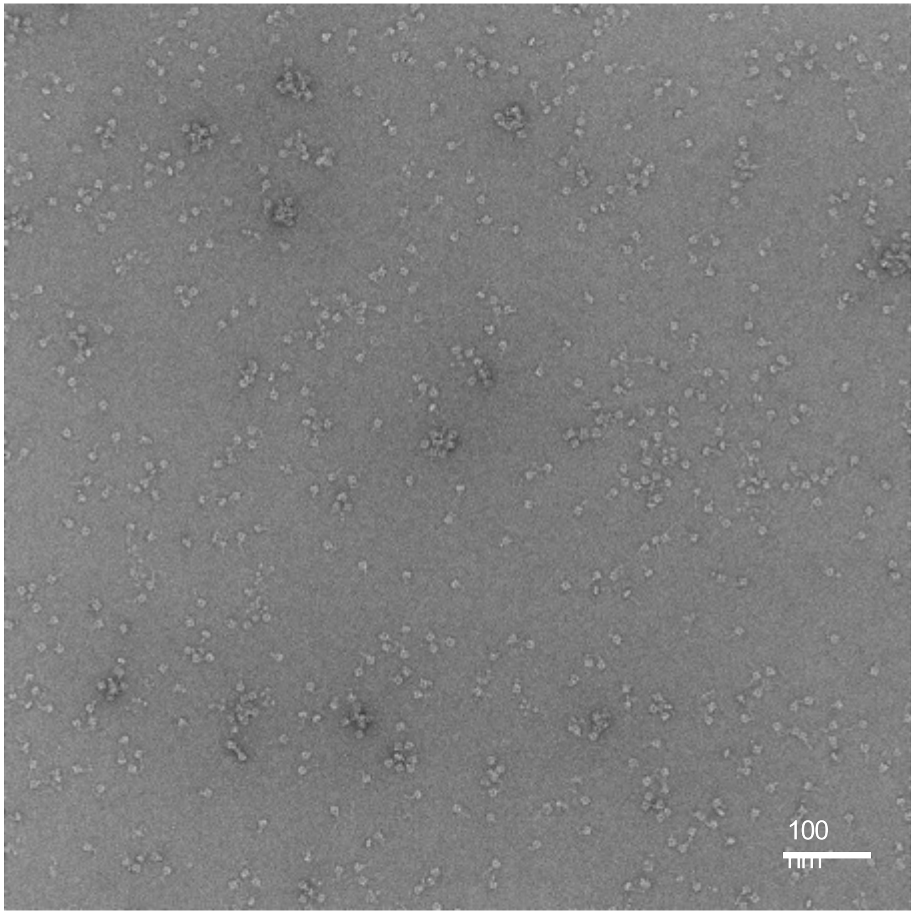

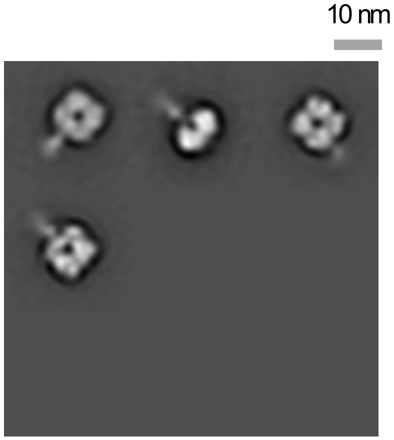

<u>Total particles picked: 12656</u>
Total particles clearly in closed state: 7405
Total particles clearly in open state: 0

N8-JD13-sNAp-282

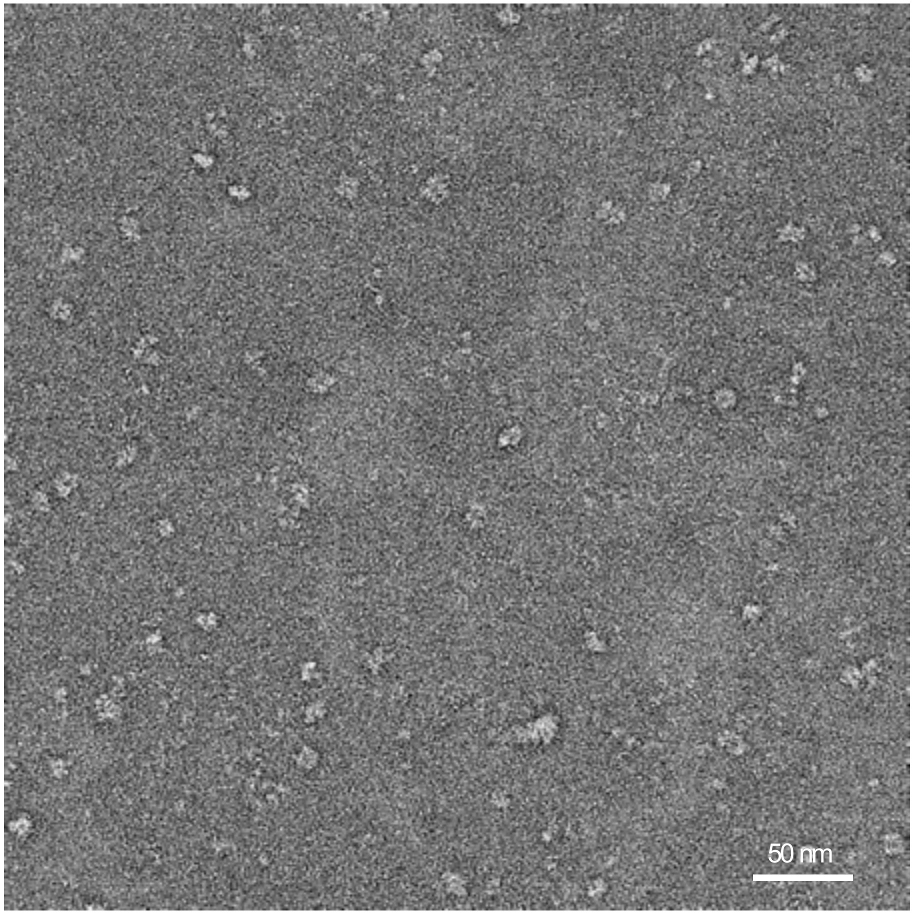

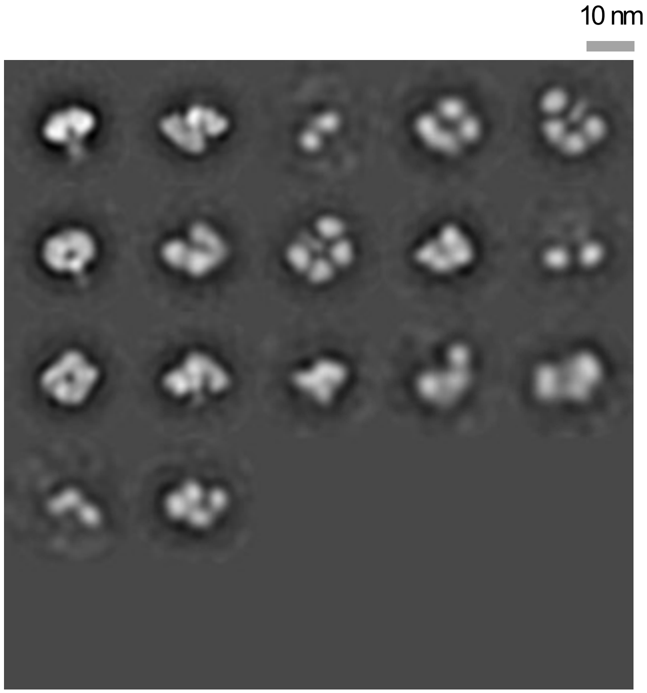

<u>Total particles picked: 6305</u>

Total particles clearly in closed state: 2049

Total particles clearly in open state: 1054

N8-JD13-sNAp-285

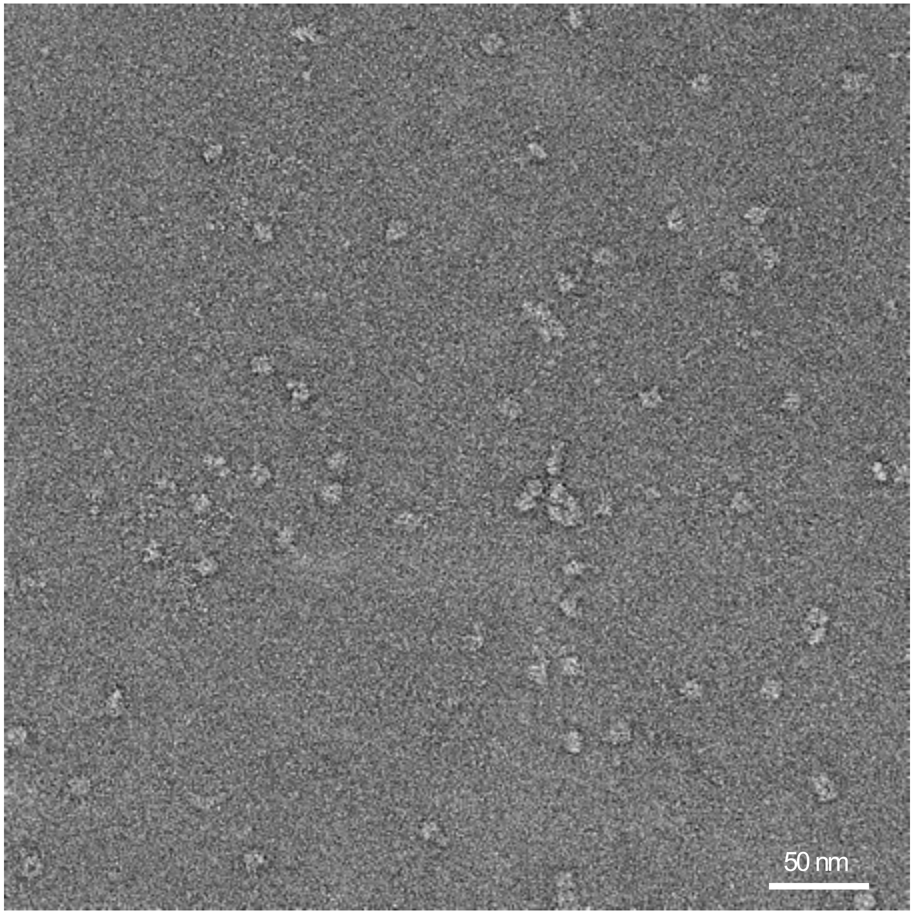

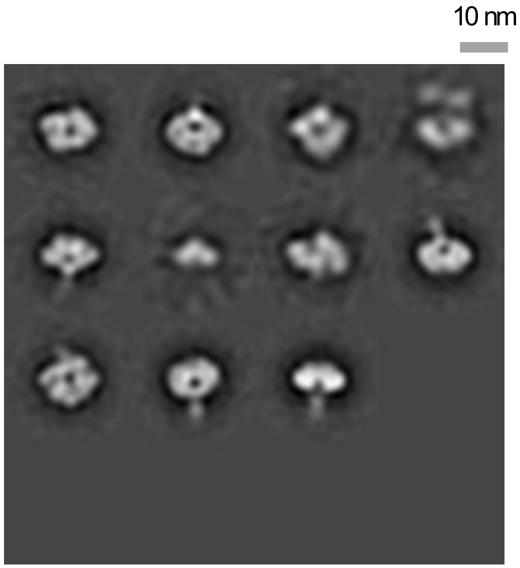

<u>Total particles picked: 7923</u>
Total particles clearly in closed state: 5493
Total particles clearly in open state: 0

N8-JD13-sNAp-285, time course study - Day 0

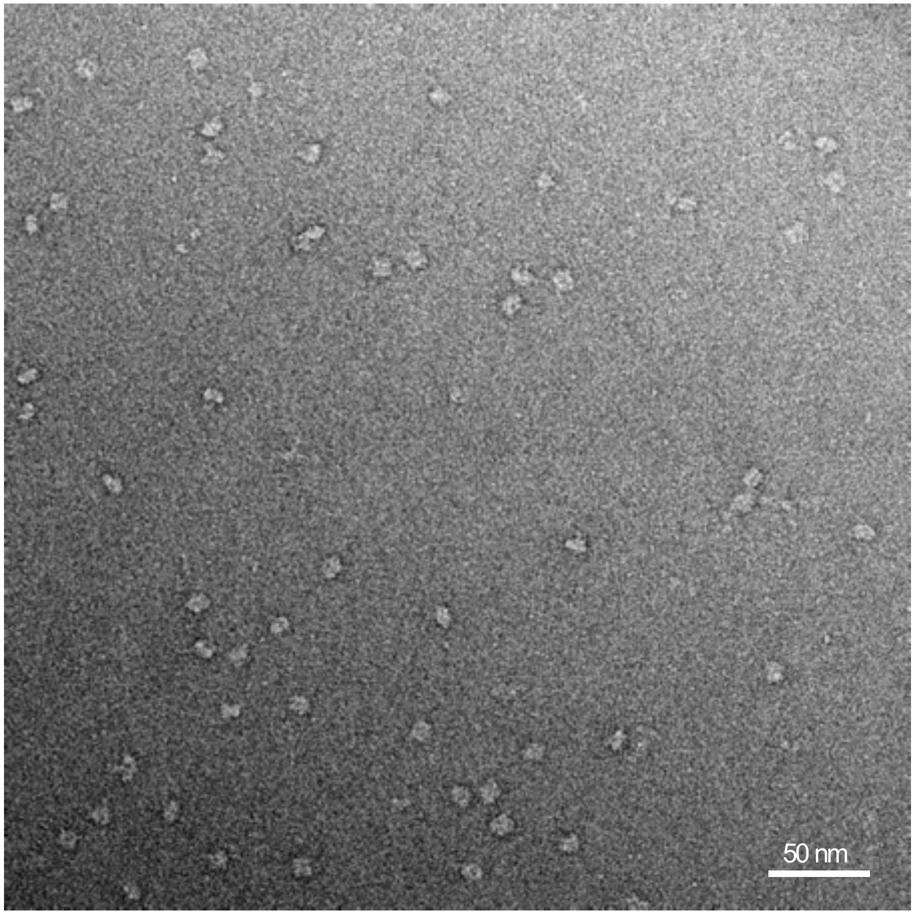

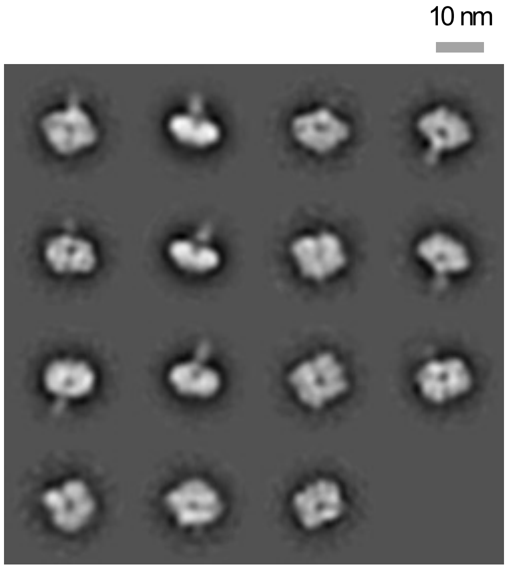

<u>Total particles picked: 7275</u>

Total particles clearly in closed state: 6055

Total particles clearly in open state: 201

N8-JD13-sNAp-285, time course study - Day 6

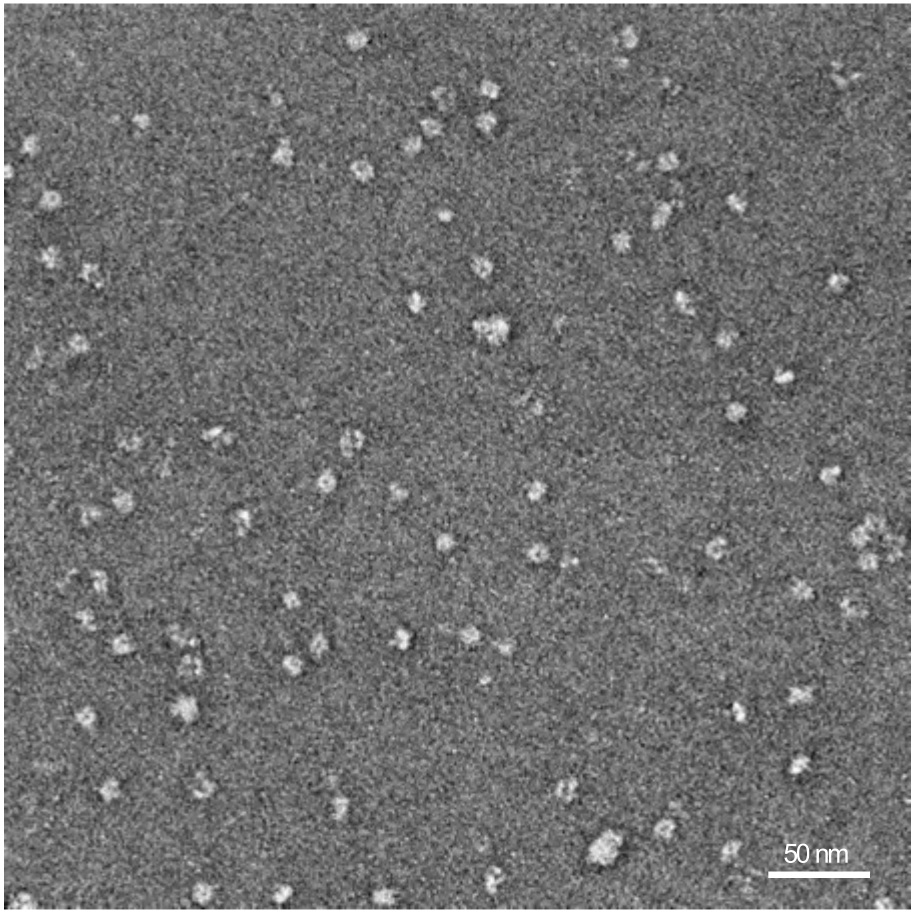

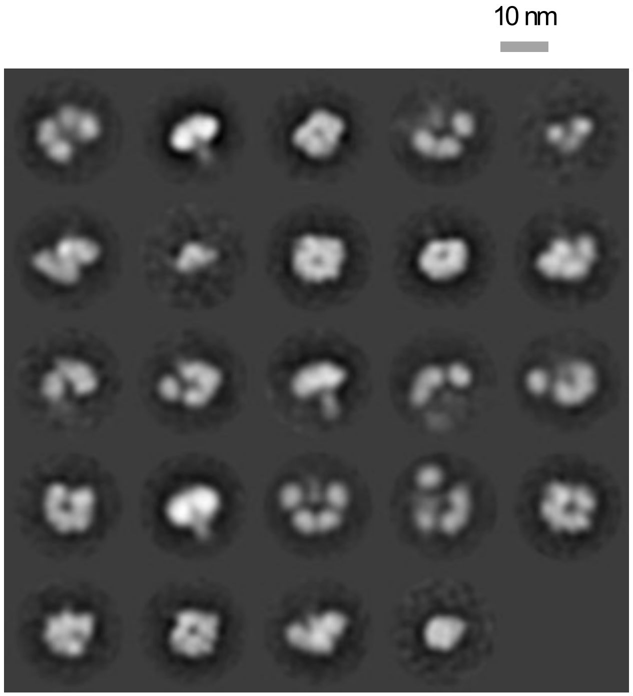

<u>Total particles picked: 7000</u>

Total particles clearly in closed state: 3477

Total particles clearly in open state: 1634

N2-WI05-desNAp-156

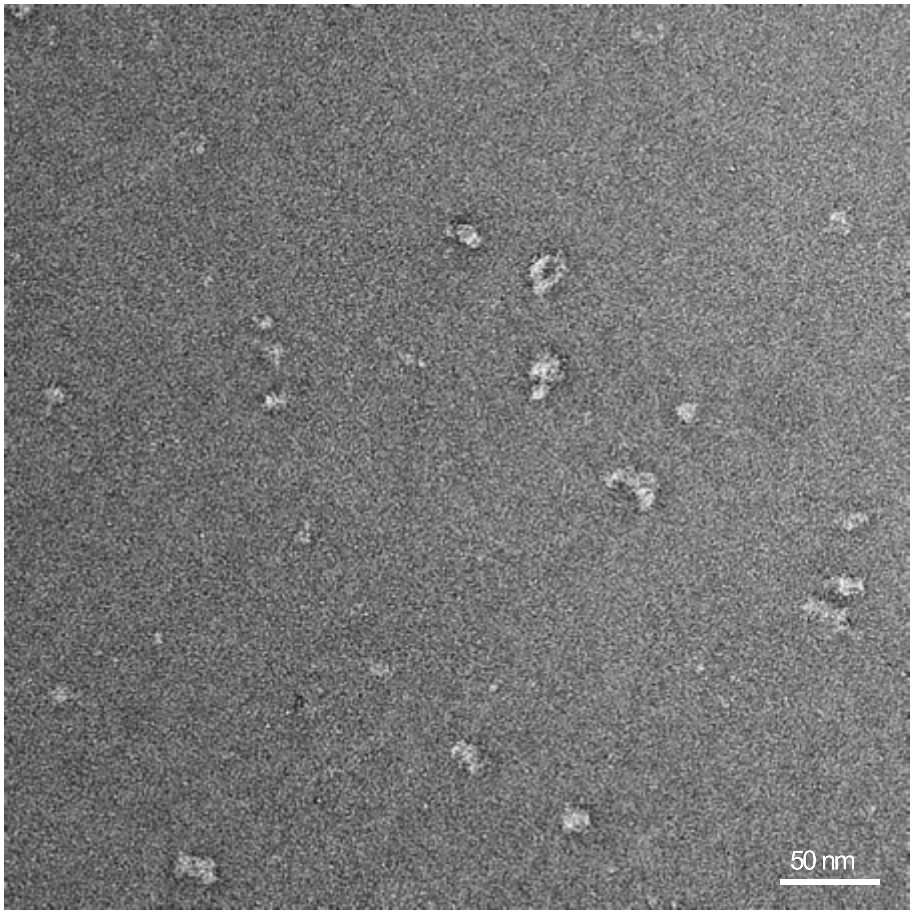

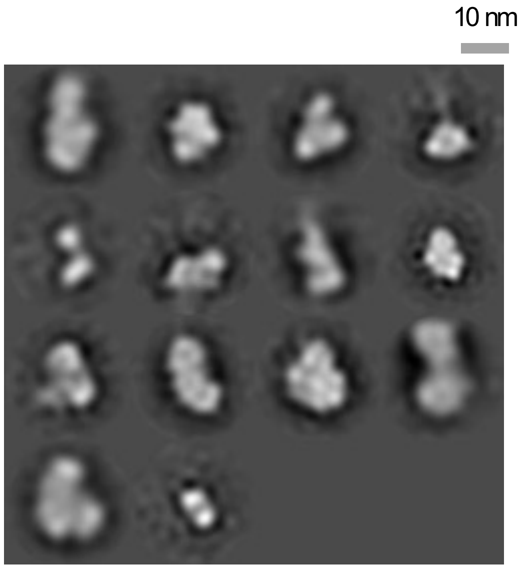

Total particles picked: 5248
No clear tetramers observed to quantify

N2-WI05-desNAp-157

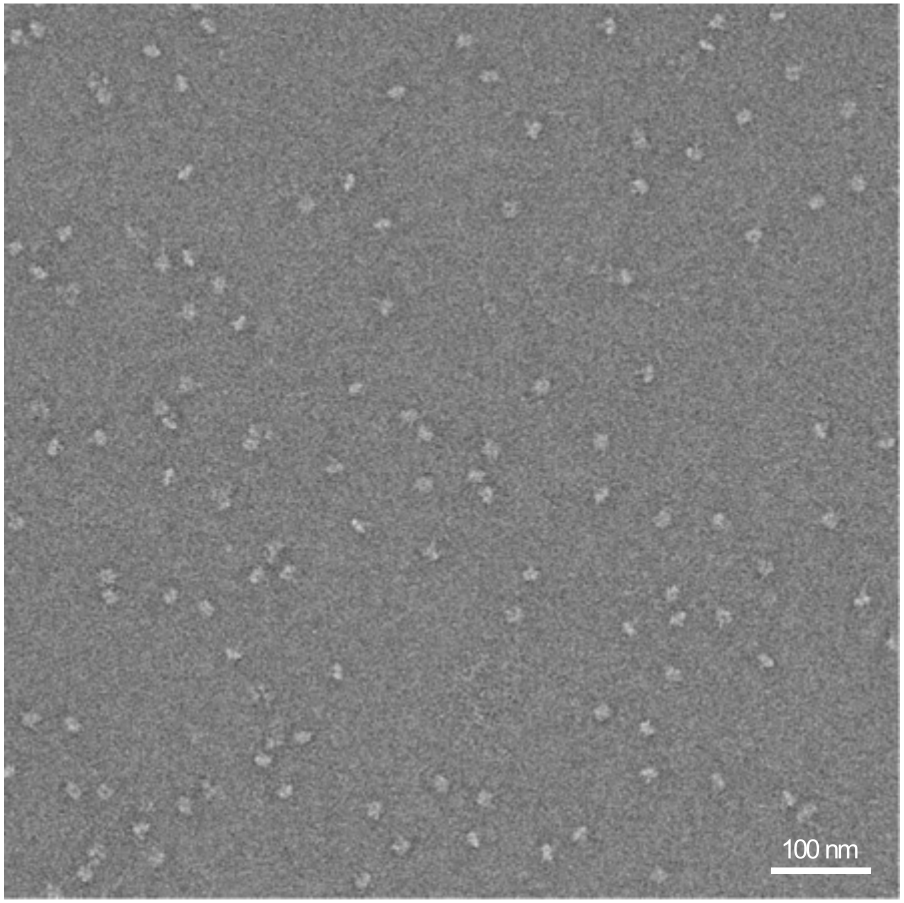

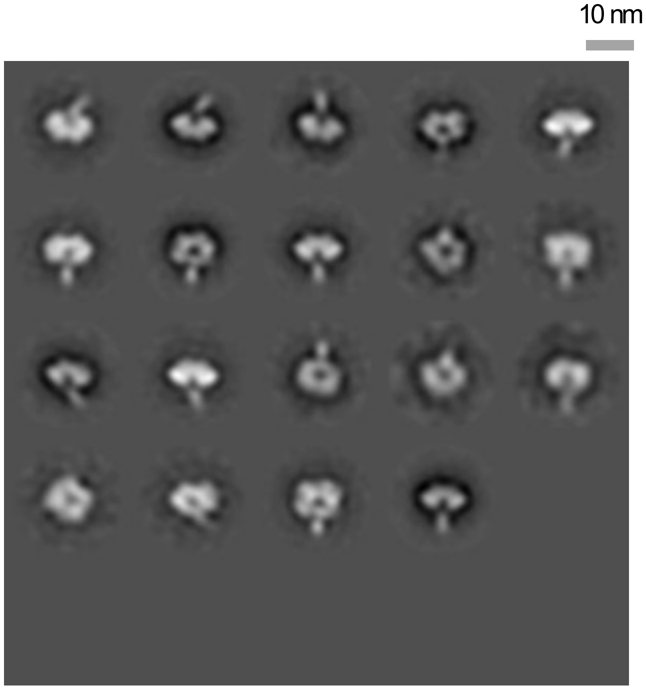

Total particles picked: 7203
Total particles clearly in closed state: 6190
Total particles clearly in open state: 75

N2-WI05-desNAp-158

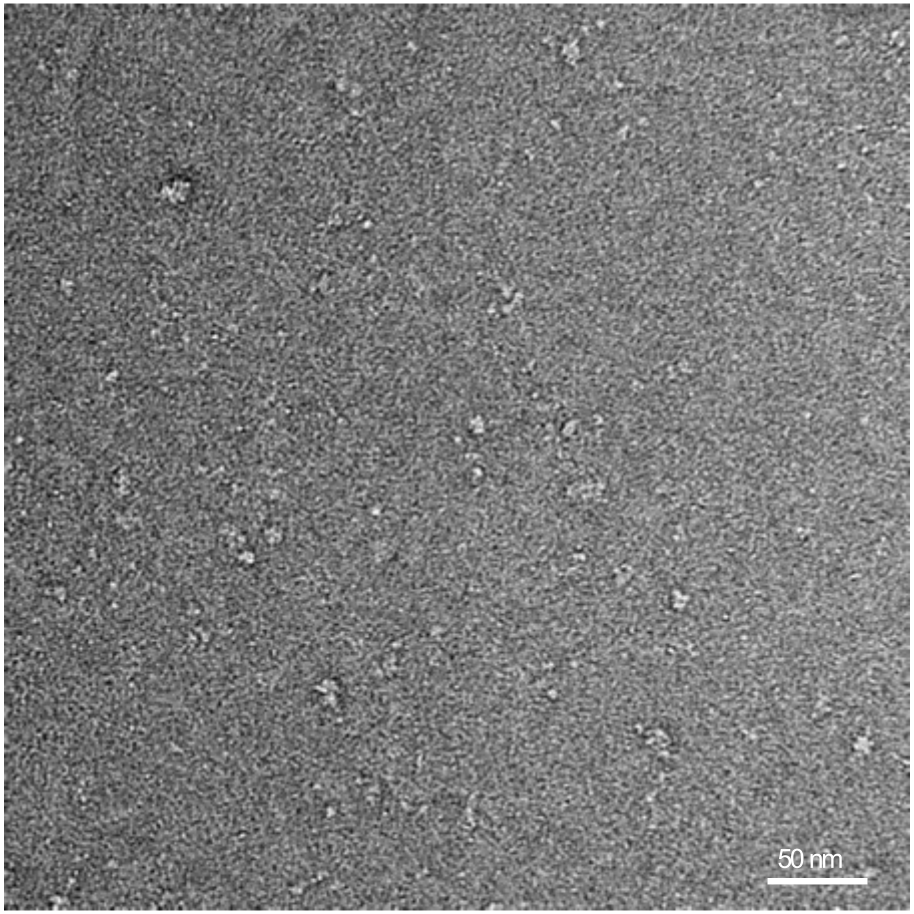

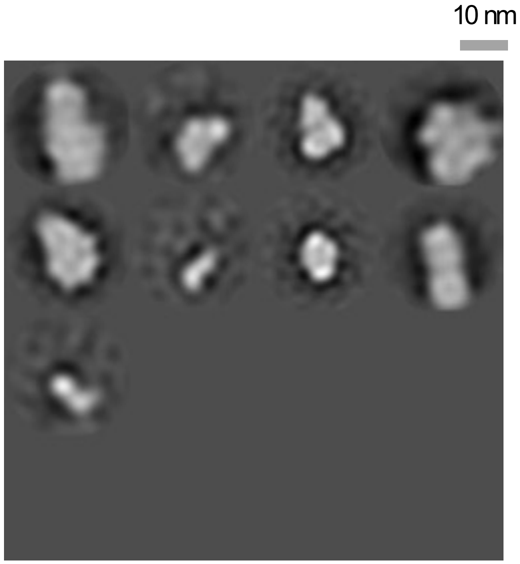

Total particles picked: 5916
No clear tetramers observed to quantify

N2-WI05-desNAp-249

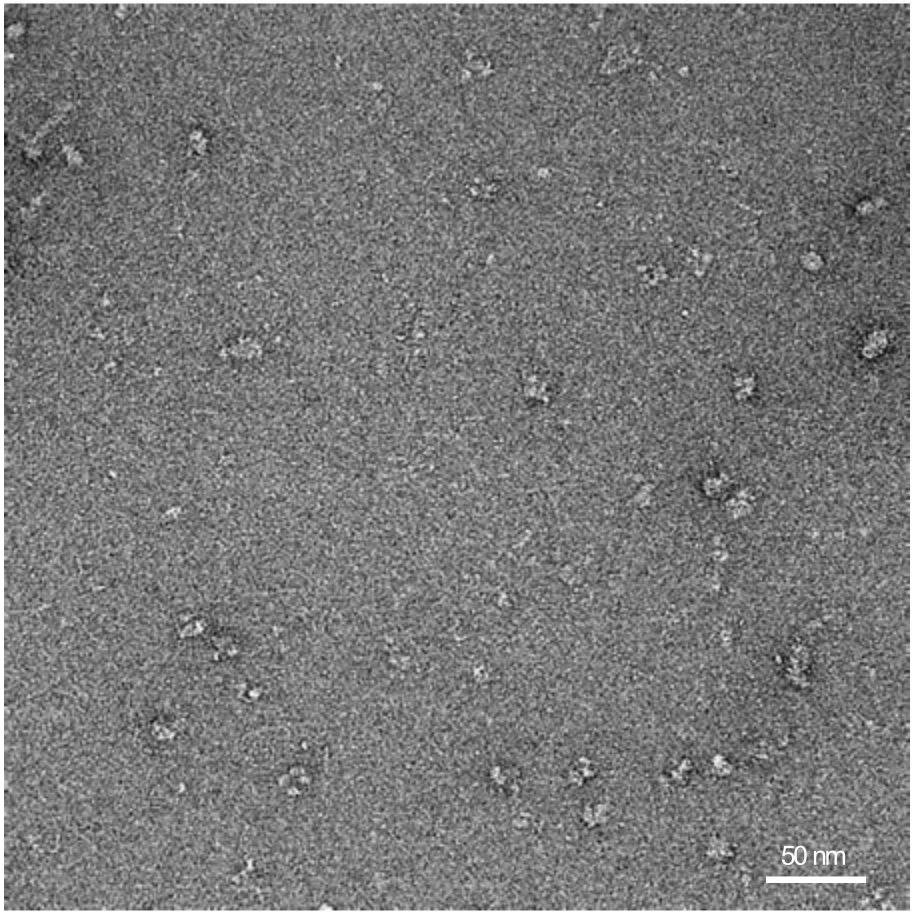

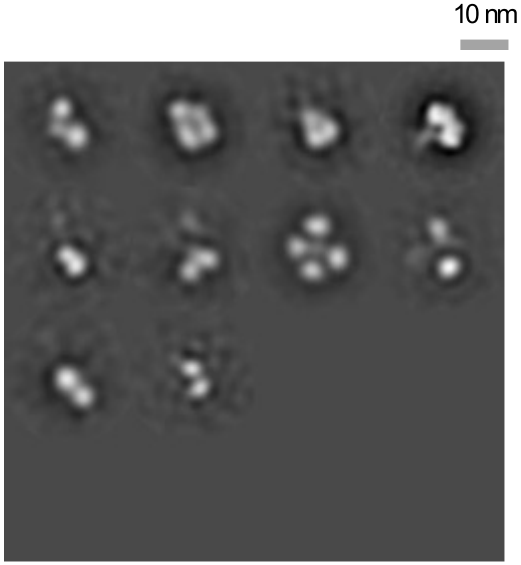

<u>Total particles picked: 8258</u>

Total particles clearly in closed state: 923

Total particles clearly in open state: 981

N2-WI05-desNAp-255

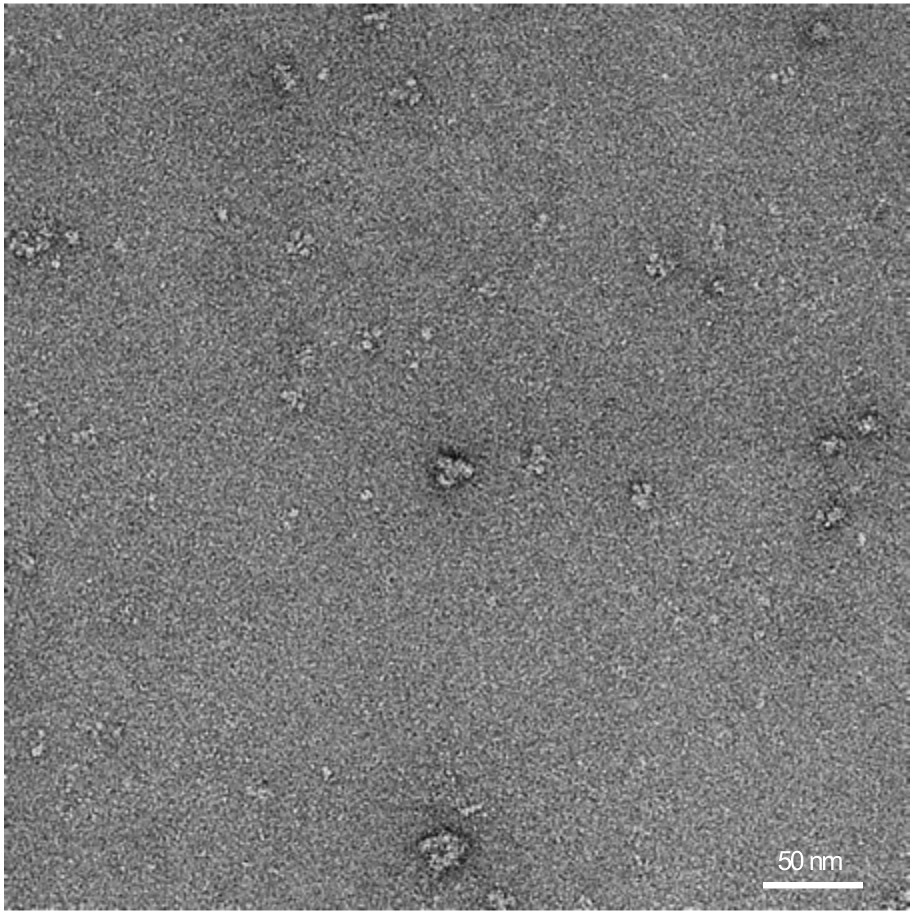

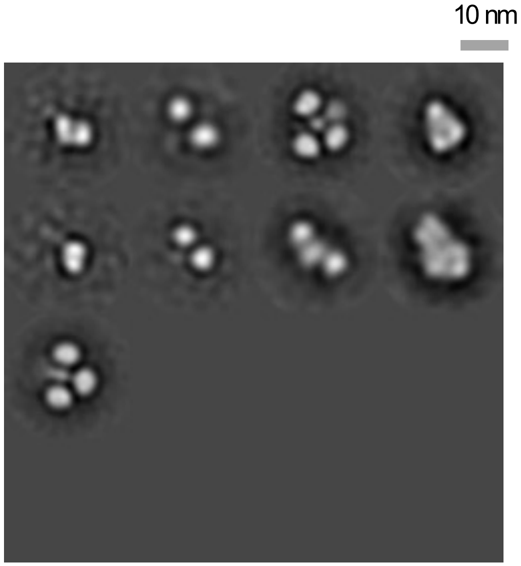

<u>Total particles picked: 6458</u>
Total particles clearly in closed state: 0
Total particles clearly in open state: 1215

N1-MI15-sNAp-155

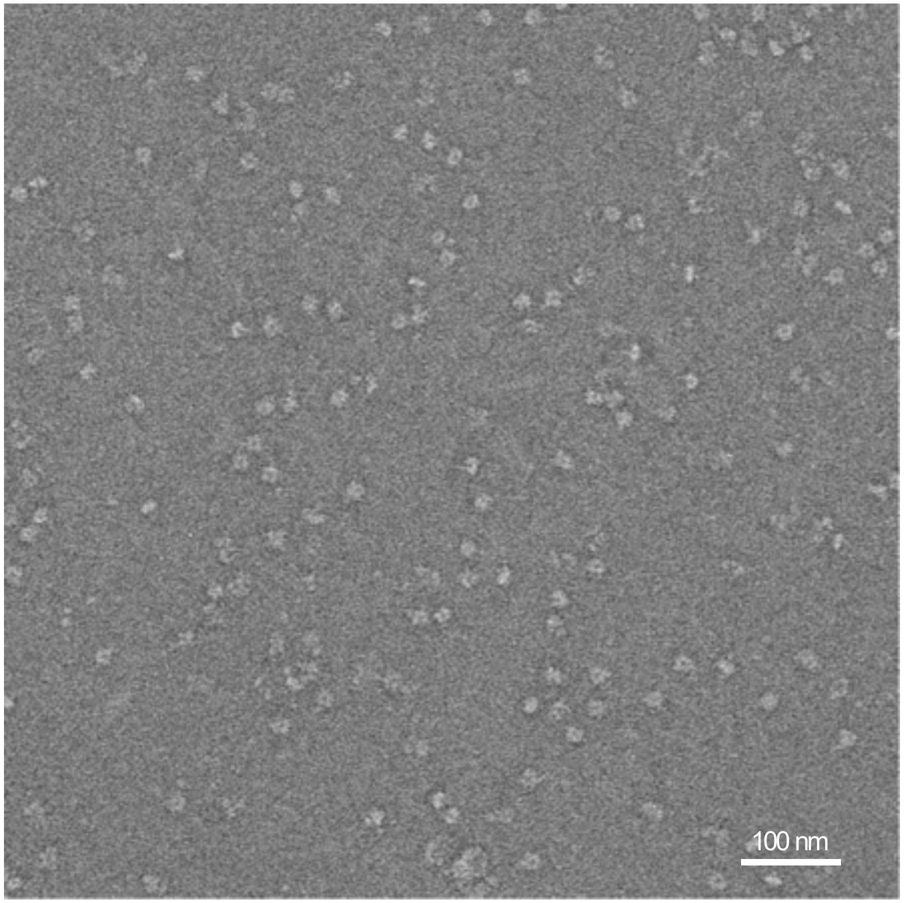

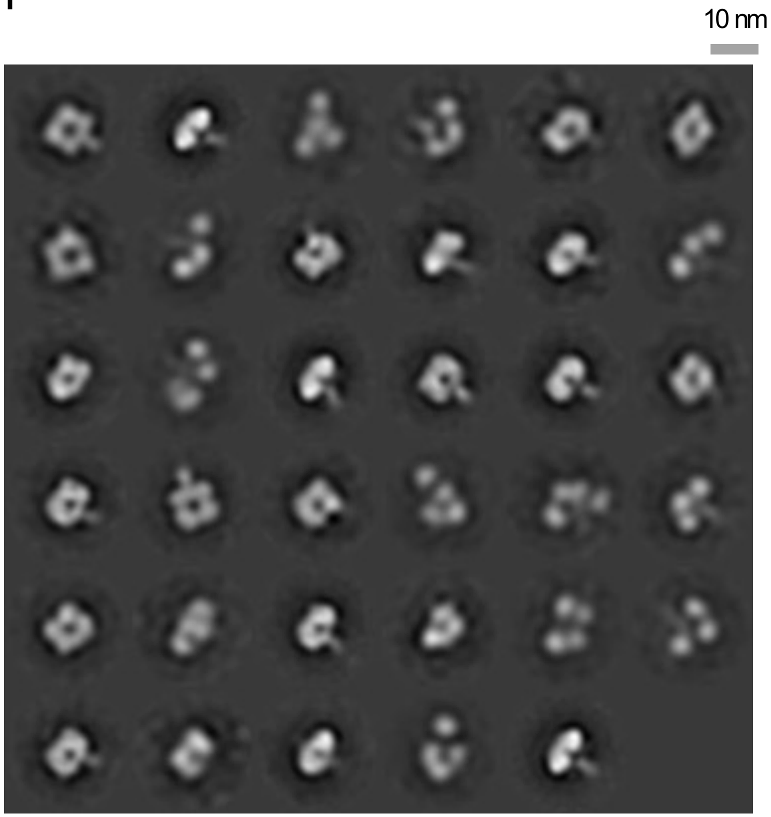

Total particles picked: 9724
Total particles clearly in closed state: 7603
Total particles clearly in open state: 1152

N1-MI15-sNAp-174

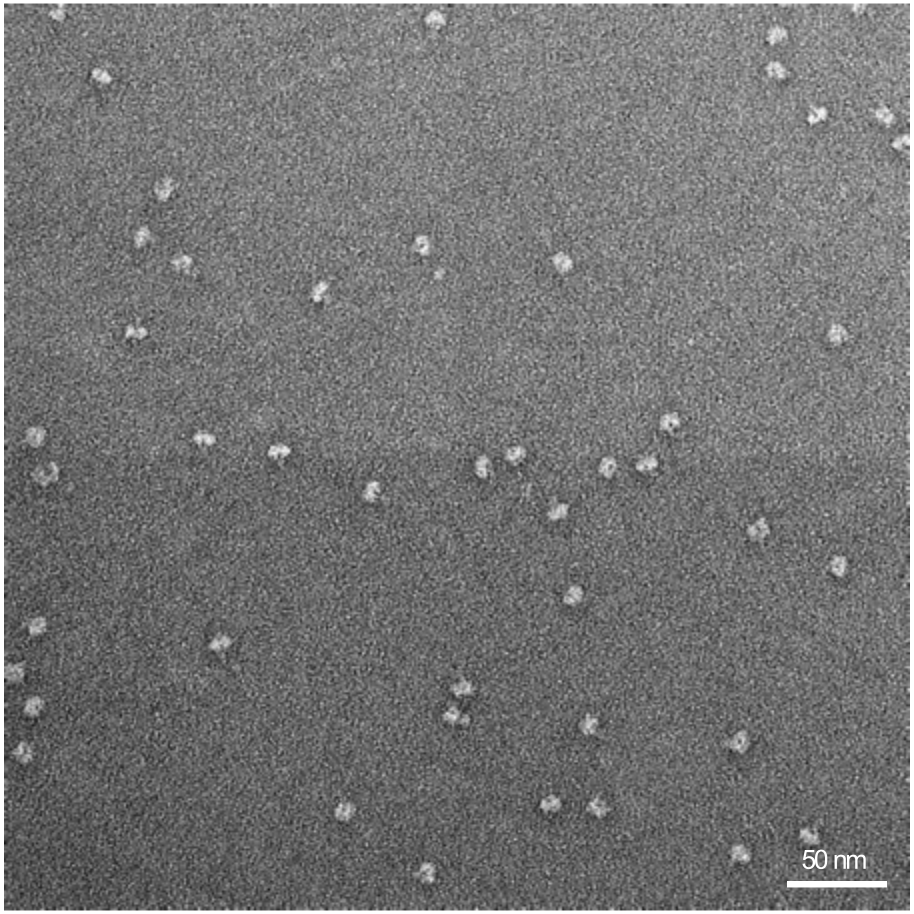

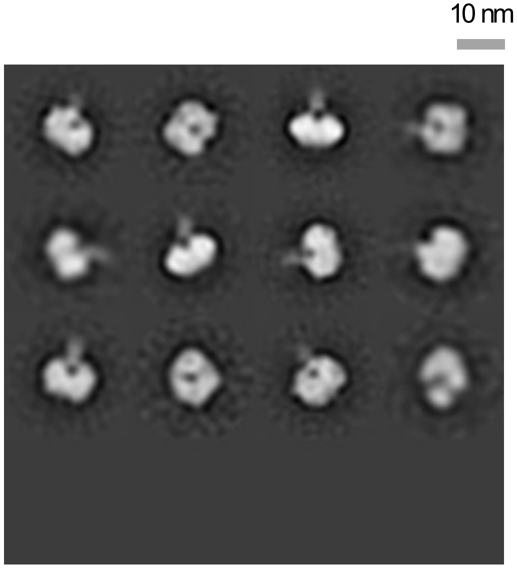

Total particles picked: 3241
Total particles clearly in closed state: 2875
Total particles clearly in open state: 178

N1-MI15-sNAp-174, time course study - Day 0

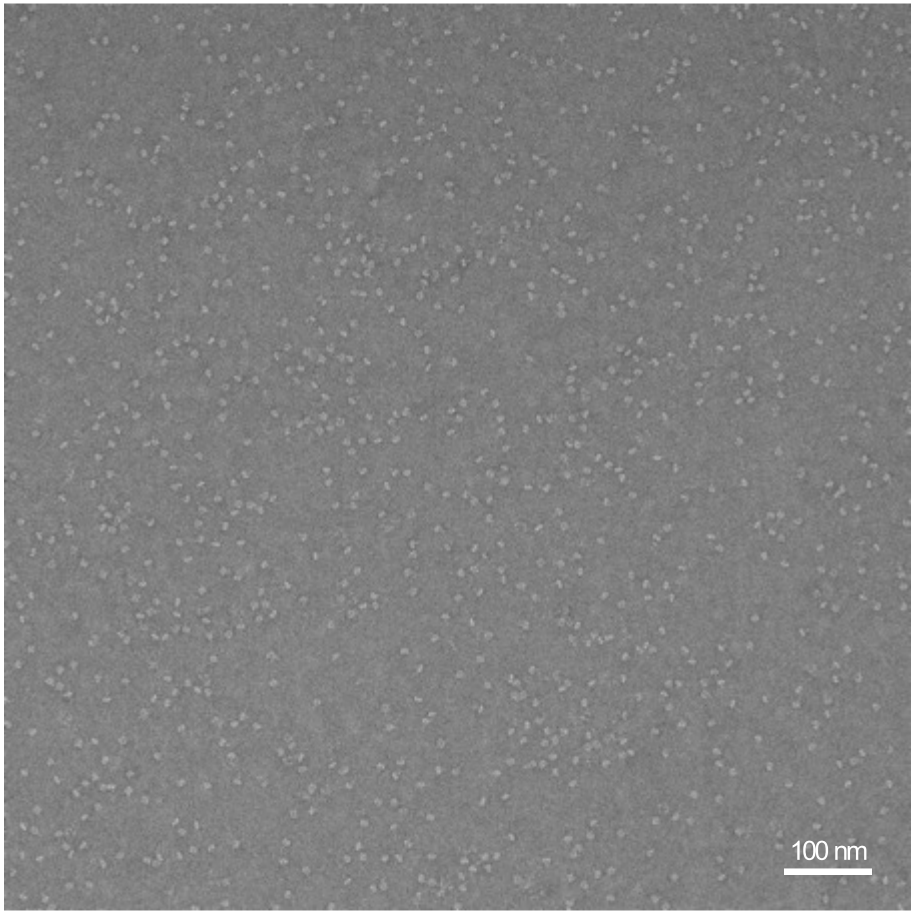

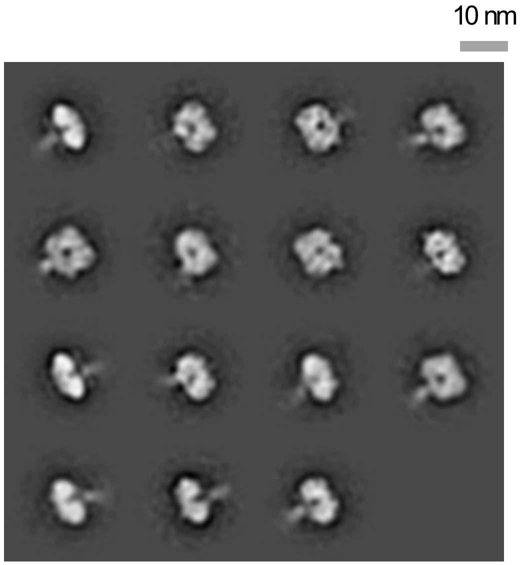

<u>Total particles picked: 6698</u>
Total particles clearly in closed state: 6300
Total particles clearly in open state: 280

N1-MI15-sNAp-174, time course study - Day 6

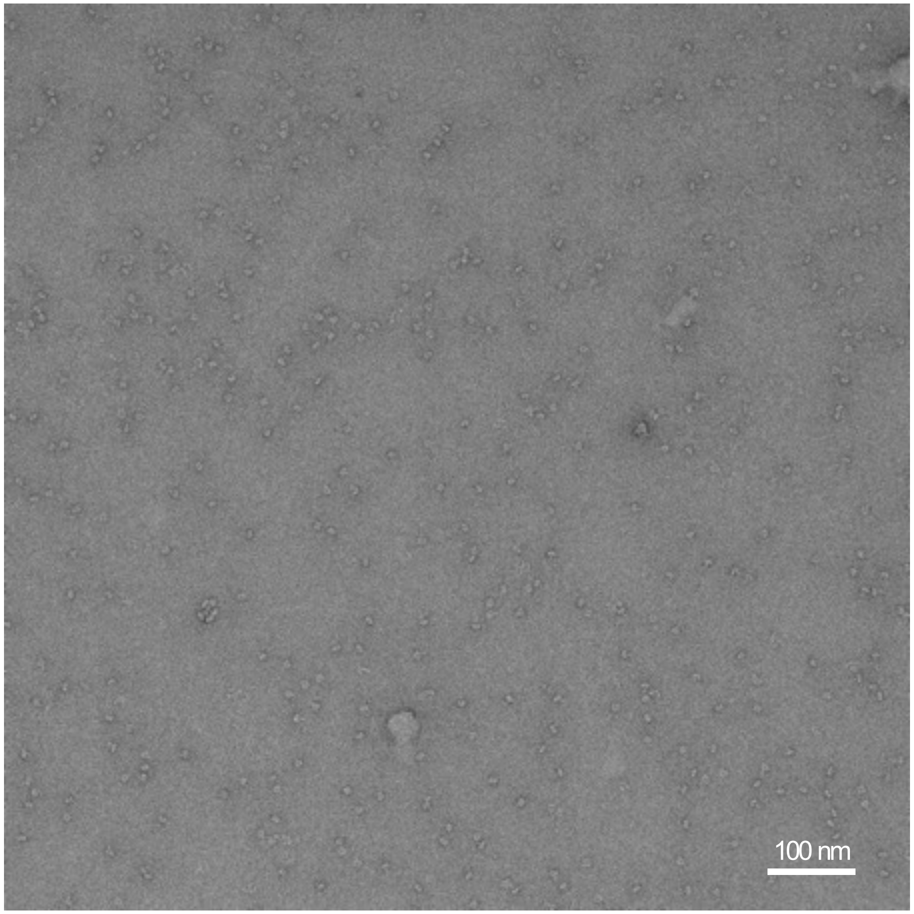

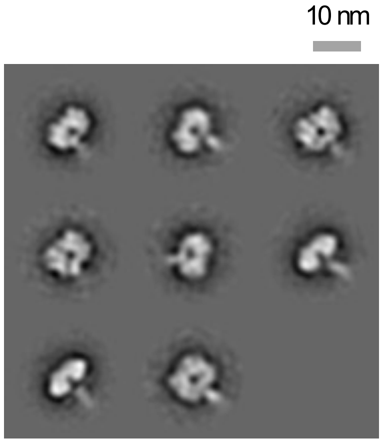

<u>Total particles picked: 3549</u>
Total particles clearly in closed state: 2702
Total particles clearly in open state: 93

N1-MI15-sNAp-174, time course study - Day 10

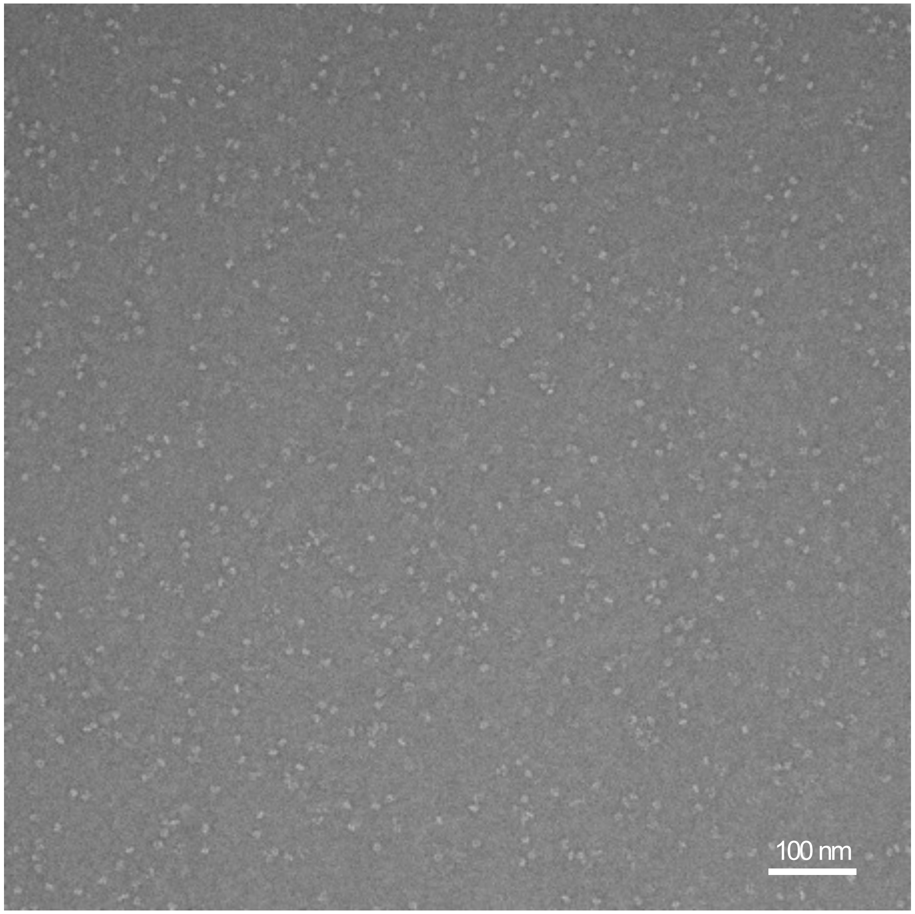

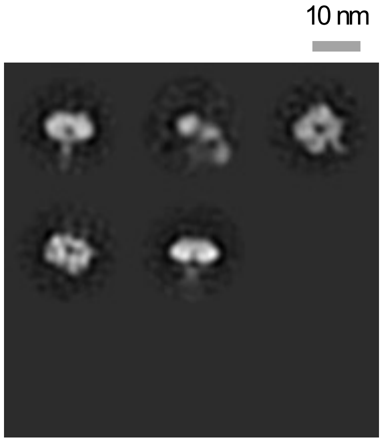

<u>Total particles picked: 5314</u>
Total particles clearly in closed state: 2108
Total particles clearly in open state: 330

N1-MI15-sNAp-174, time course study - Day 15

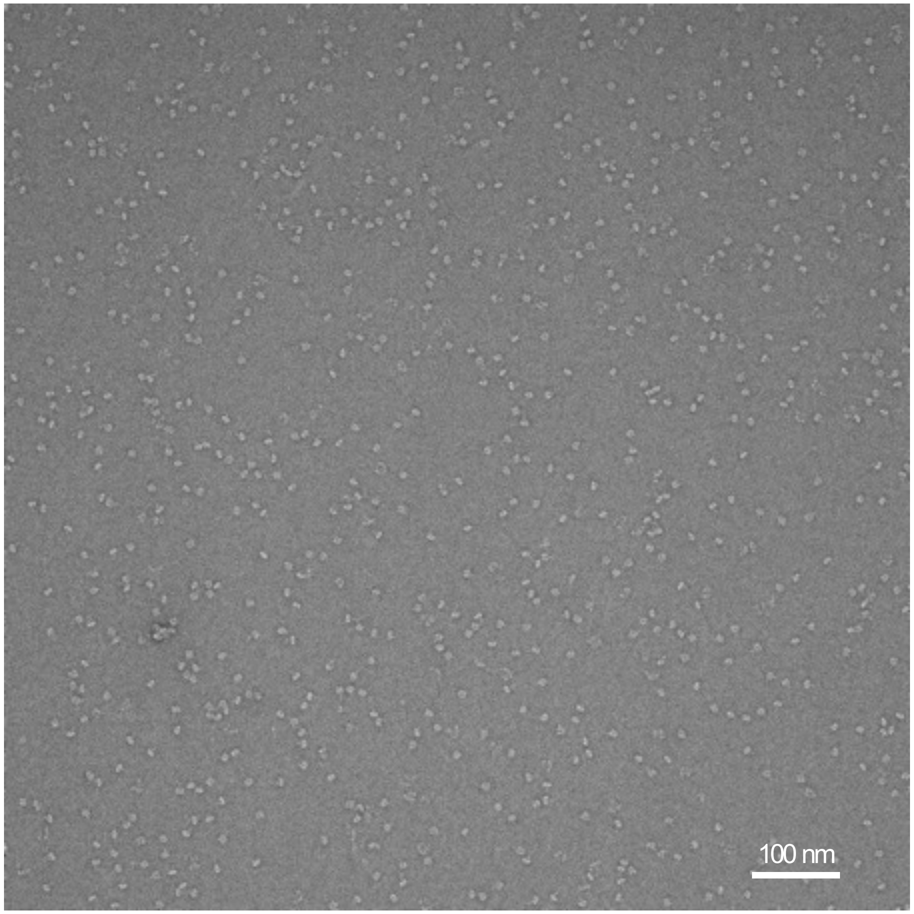

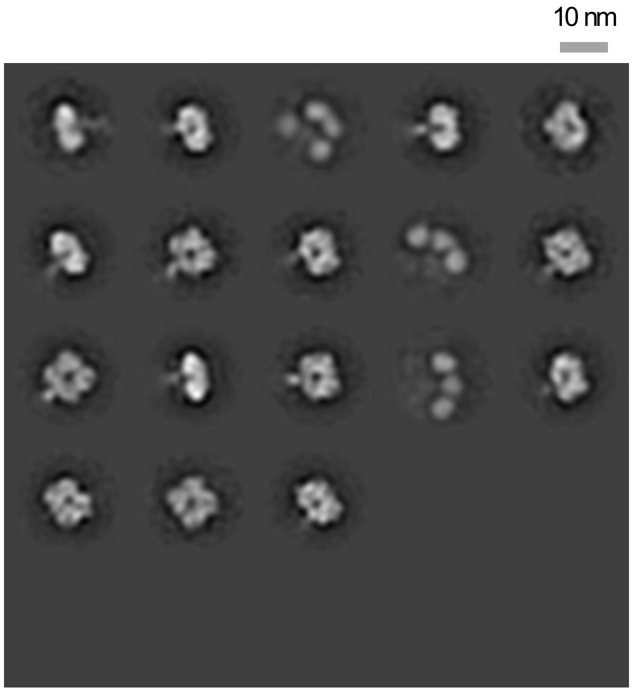

<u>Total particles picked: 6564</u>

Total particles clearly in closed state: 5639

Total particles clearly in open state: 797

N1-MI15-sNAp-165

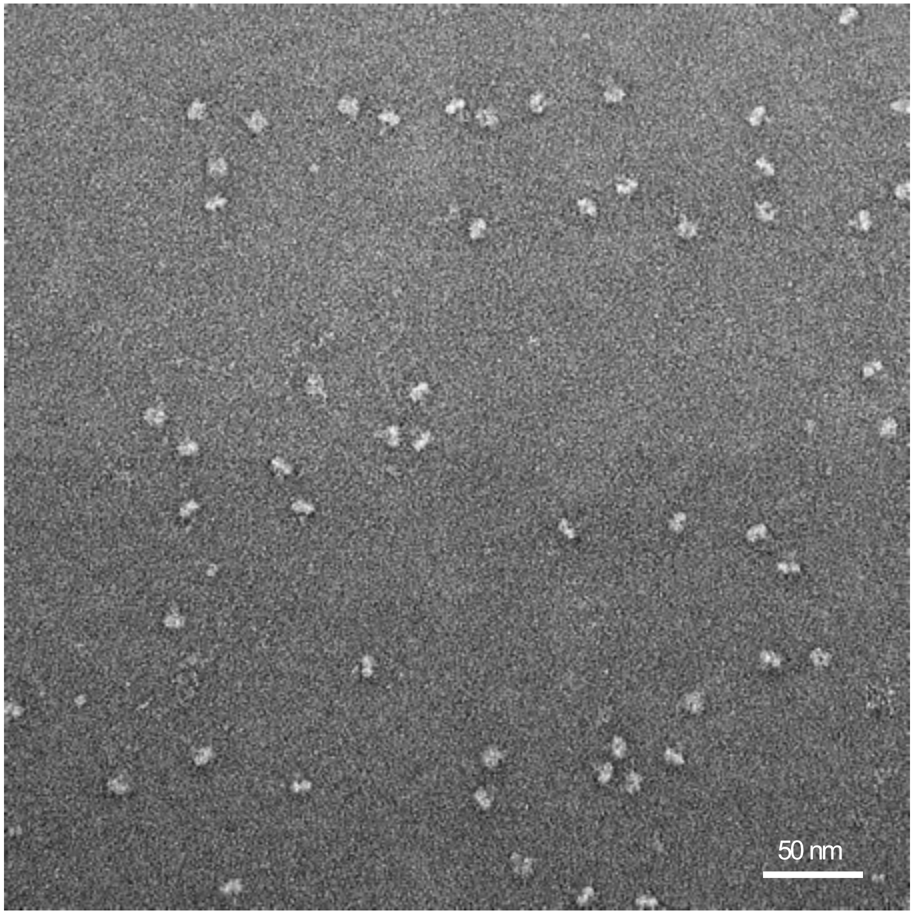

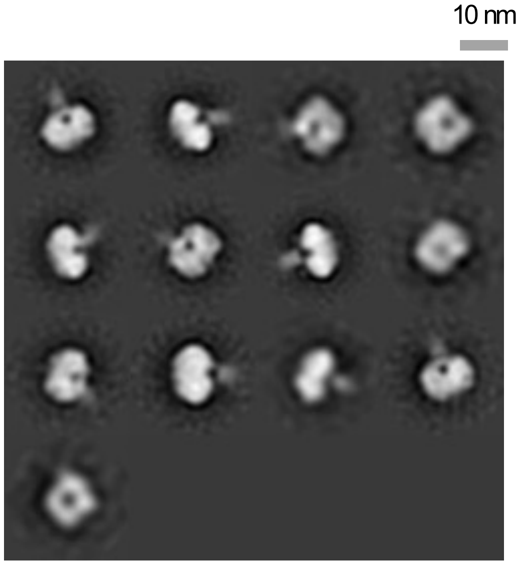

Total particles picked: 4557
Total particles clearly in closed state: 4202
Total particles clearly in open state: 0

N1-MI15-sNAp-176

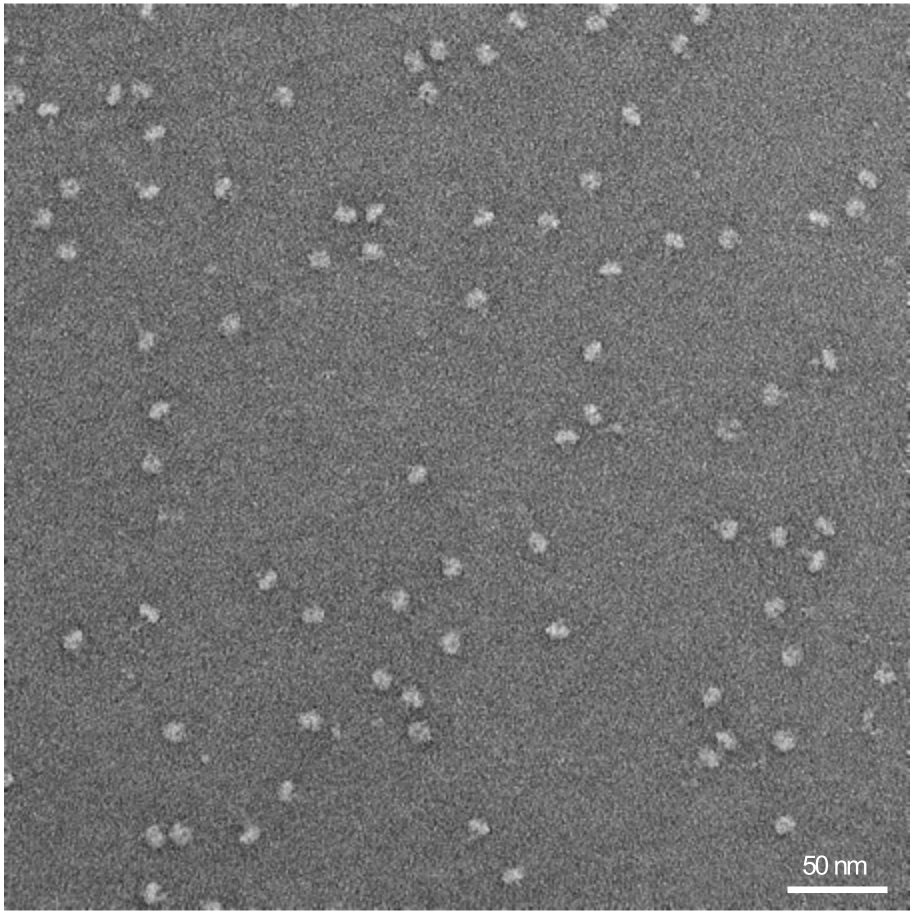

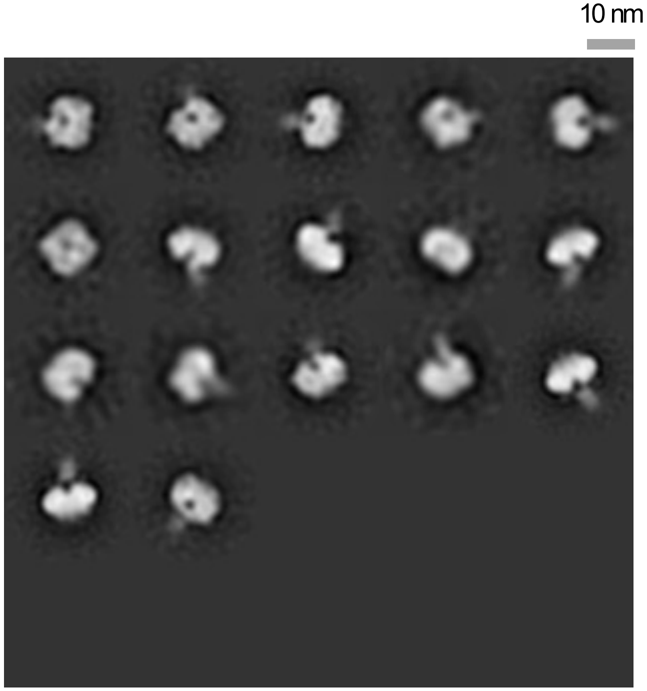

Total particles picked: 6859
Total particles clearly in closed state: 6507
Total particles clearly in open state: 0

N1-MI15-sNAp-183

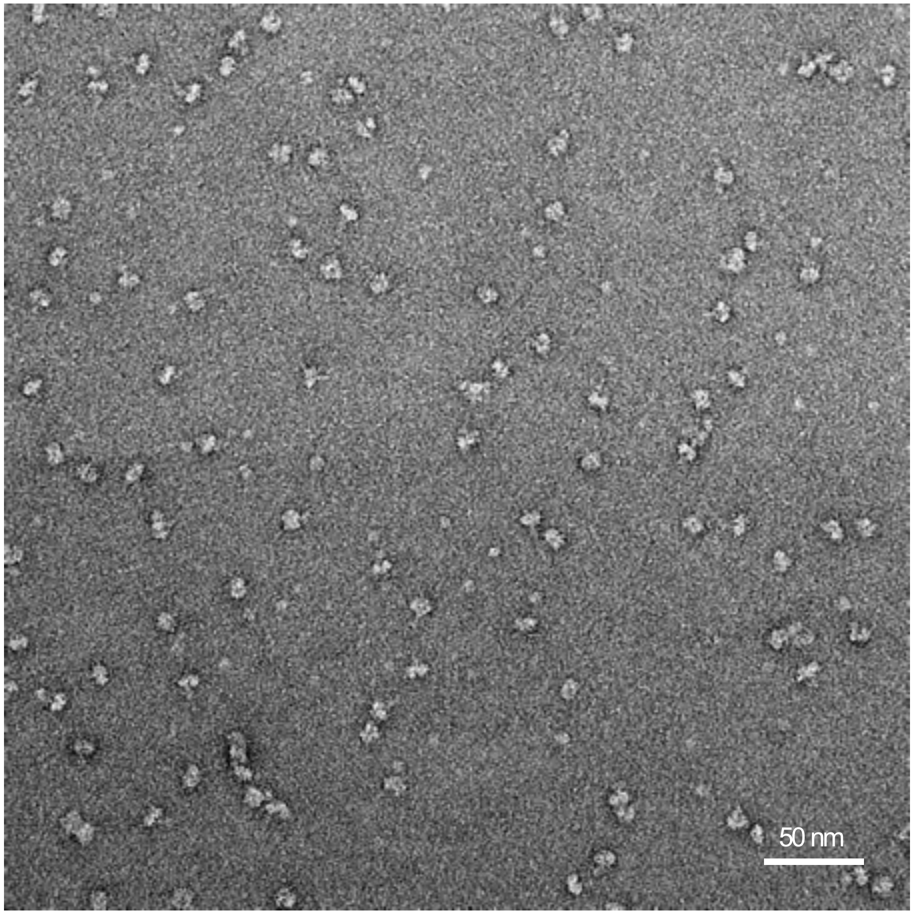

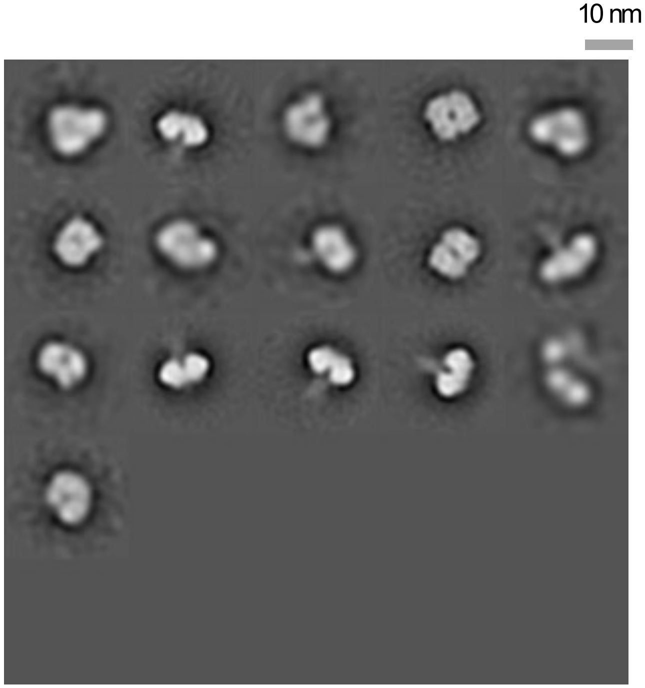

<u>Total particles picked: 7247</u>
Total particles clearly in closed state: 5602
Total particles clearly in open state: 238

N1-VN04-sNAp-155

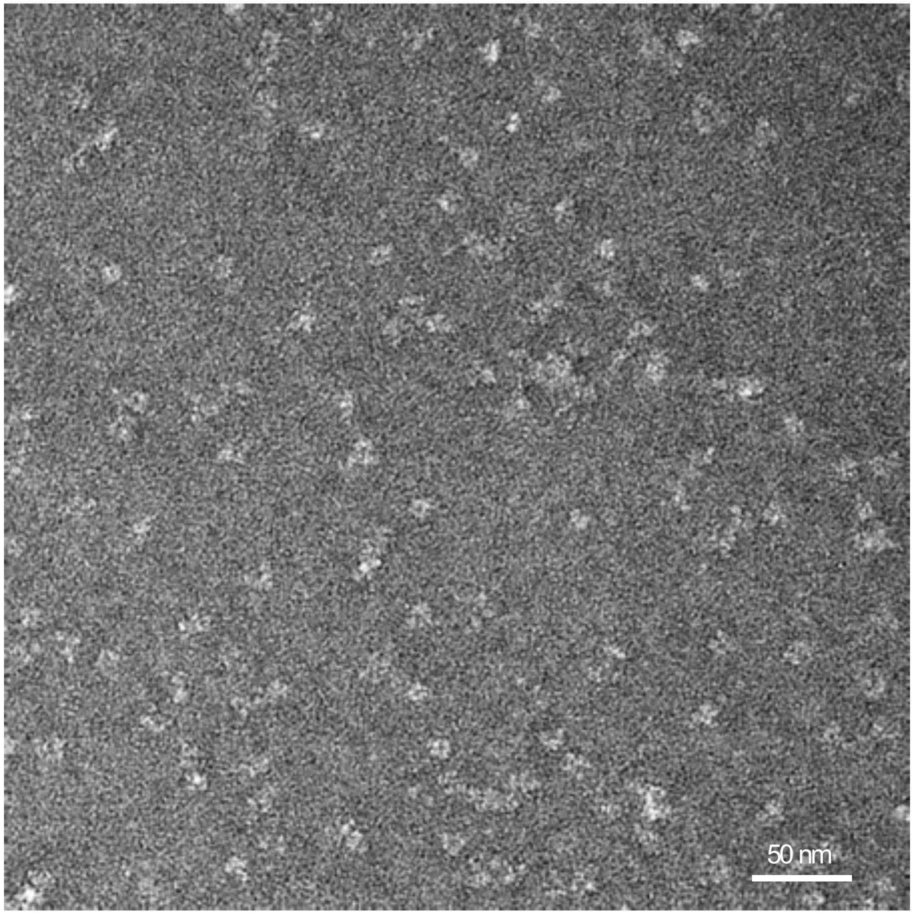

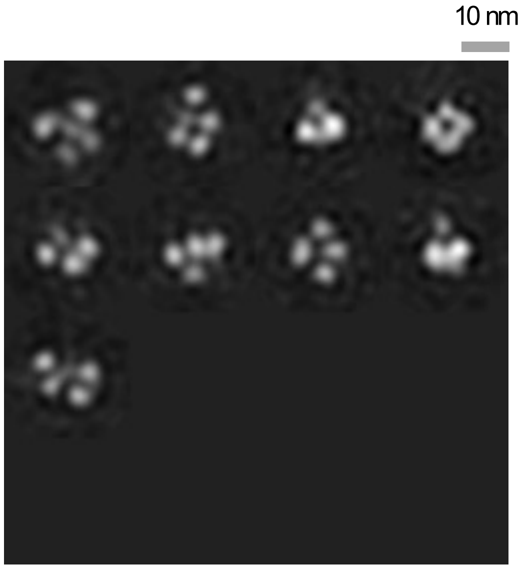

<u>Total particles picked: 3469</u>
Total particles clearly in closed state: 766
Total particles clearly in open state: 1578

N1-VN04-sNAp-354

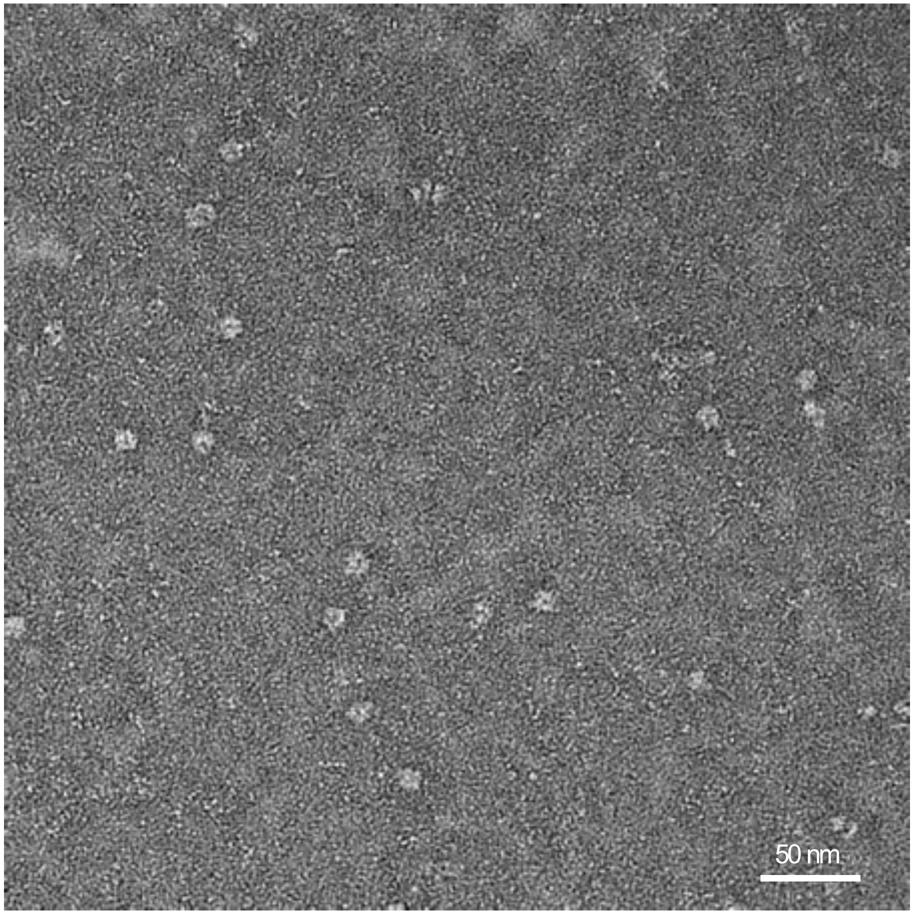

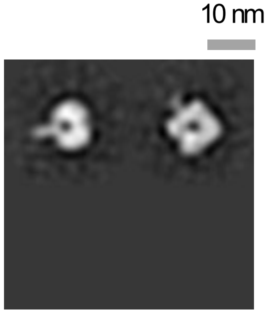

Total particles picked: 2408
Total particles clearly in closed state: 760
Total particles clearly in open state: 0

N1-WSN33-sNAp-155

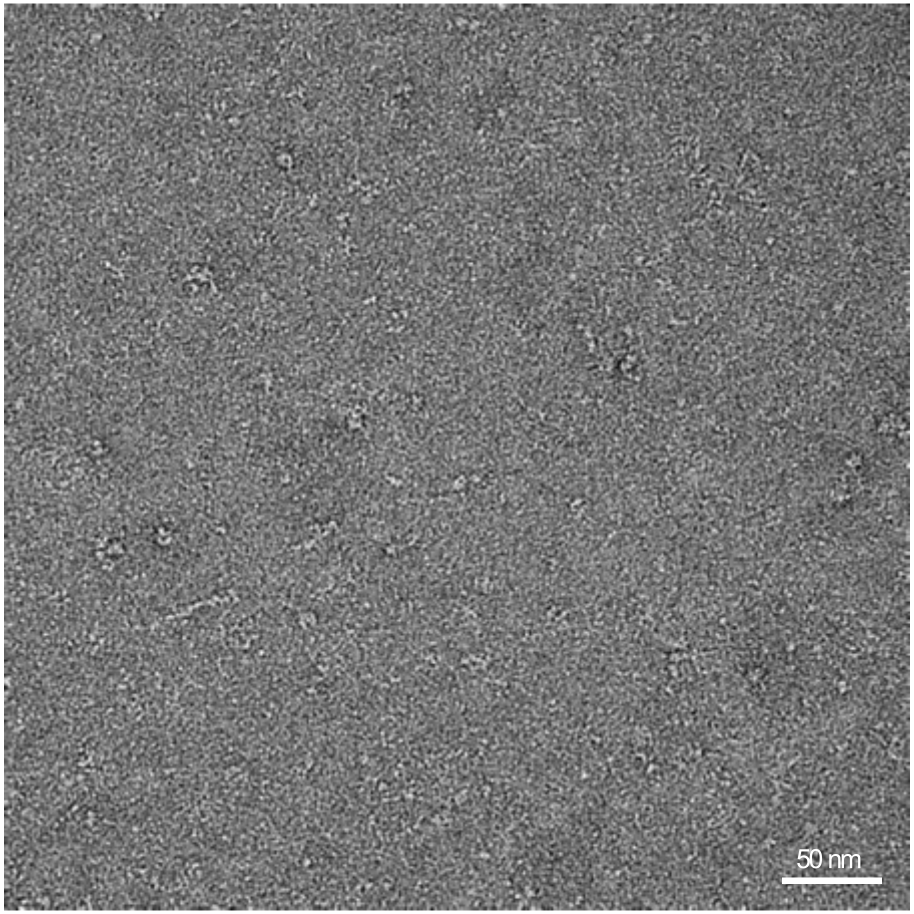

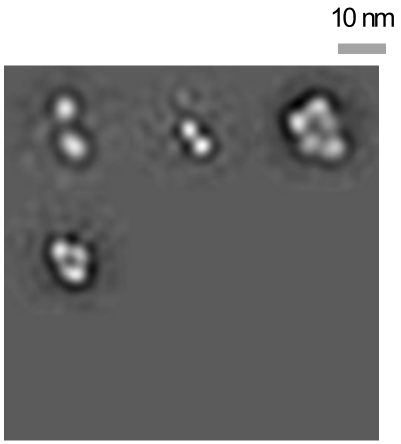

<u>Total particles picked: 9809</u>
No clear tetramers observed to quantify

N1-WSN33-sNAp-366

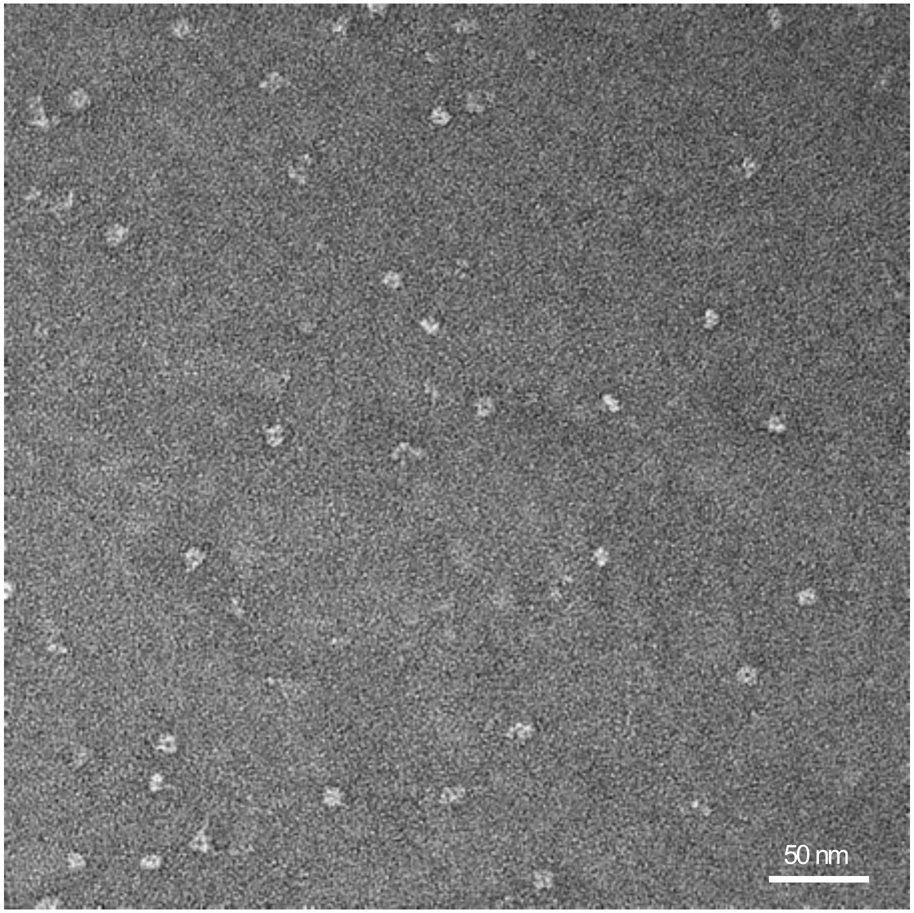

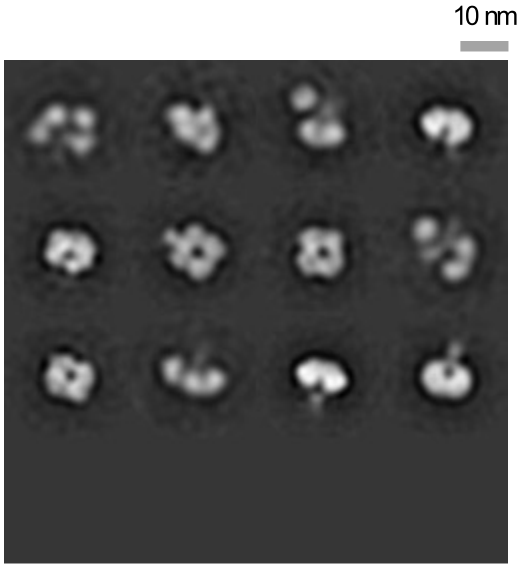

Total particles picked: 5272
Total particles clearly in closed state: 2963
Total particles clearly in open state: 662

N1-WSN33-sNAp-367

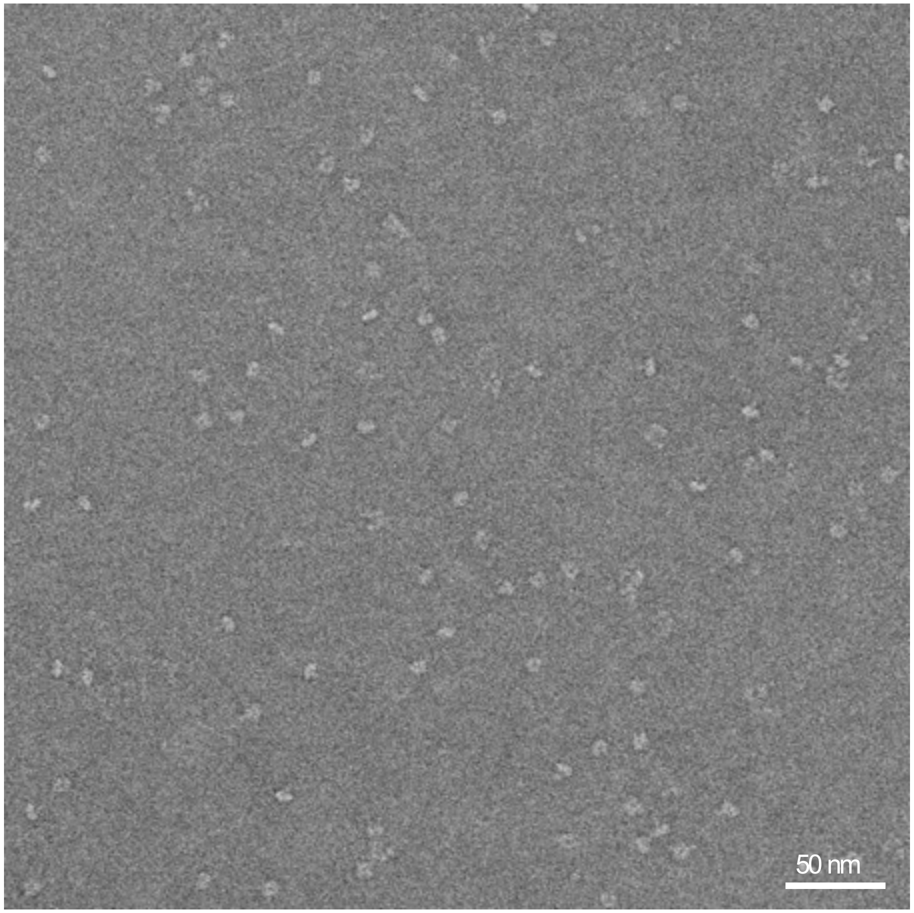

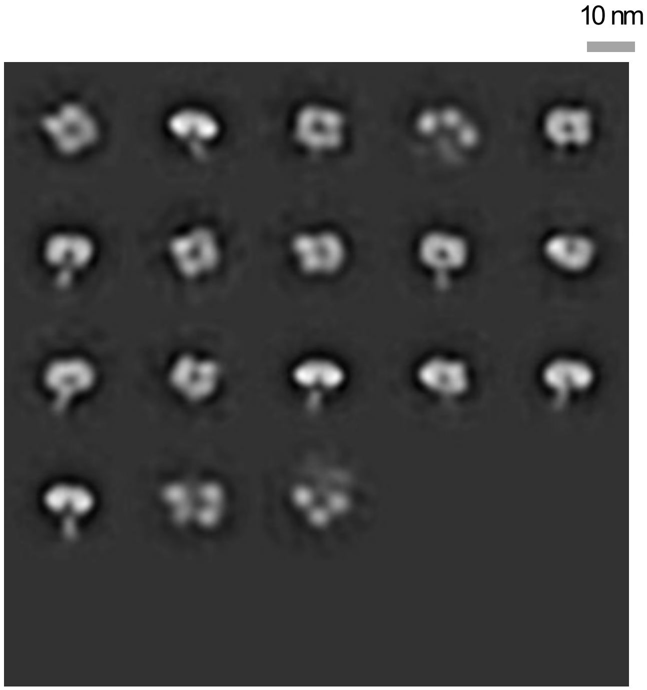

<u>Total particles picked: 16395</u>
Total particles clearly in closed state: 12678
Total particles clearly in open state: 856

N1-WSN33-sNAp-375

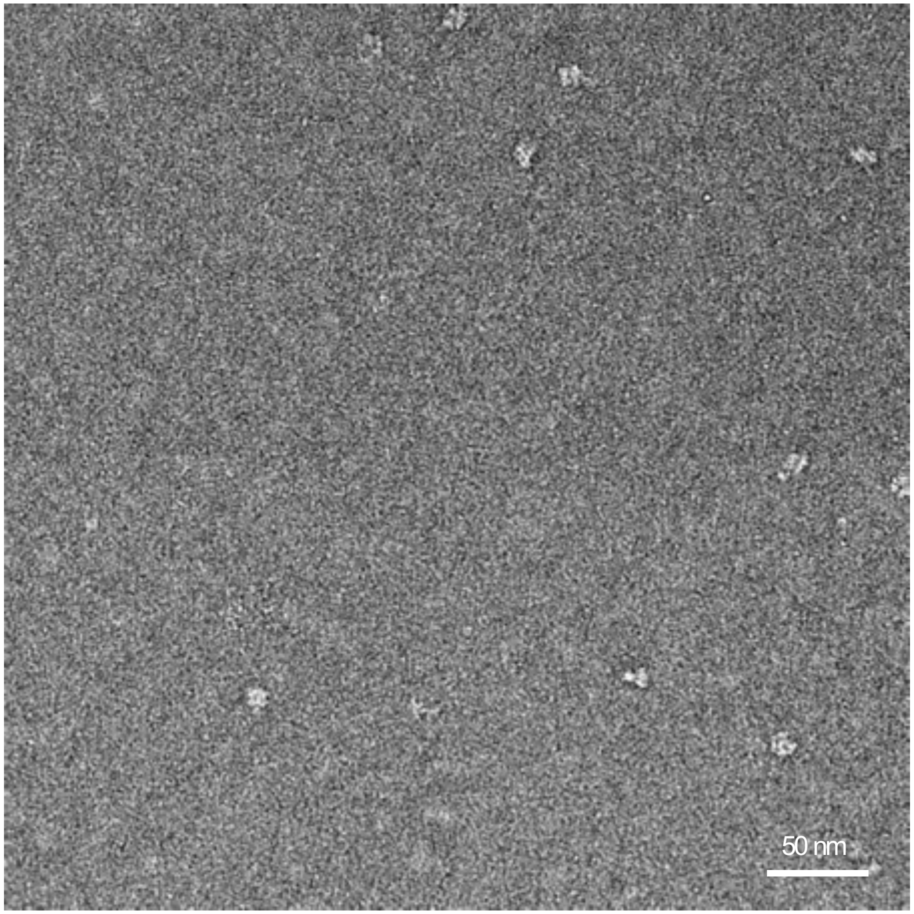

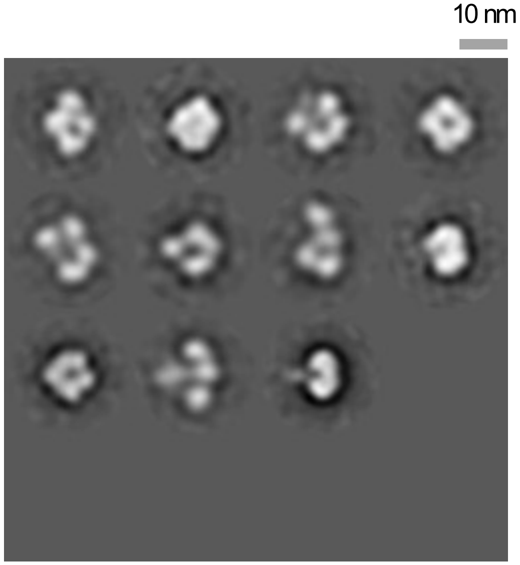

<u>Total particles picked: 1198</u>
Total particles clearly in closed state: 453
Total particles clearly in open state: 374

N1-WSN33-sNAp-378

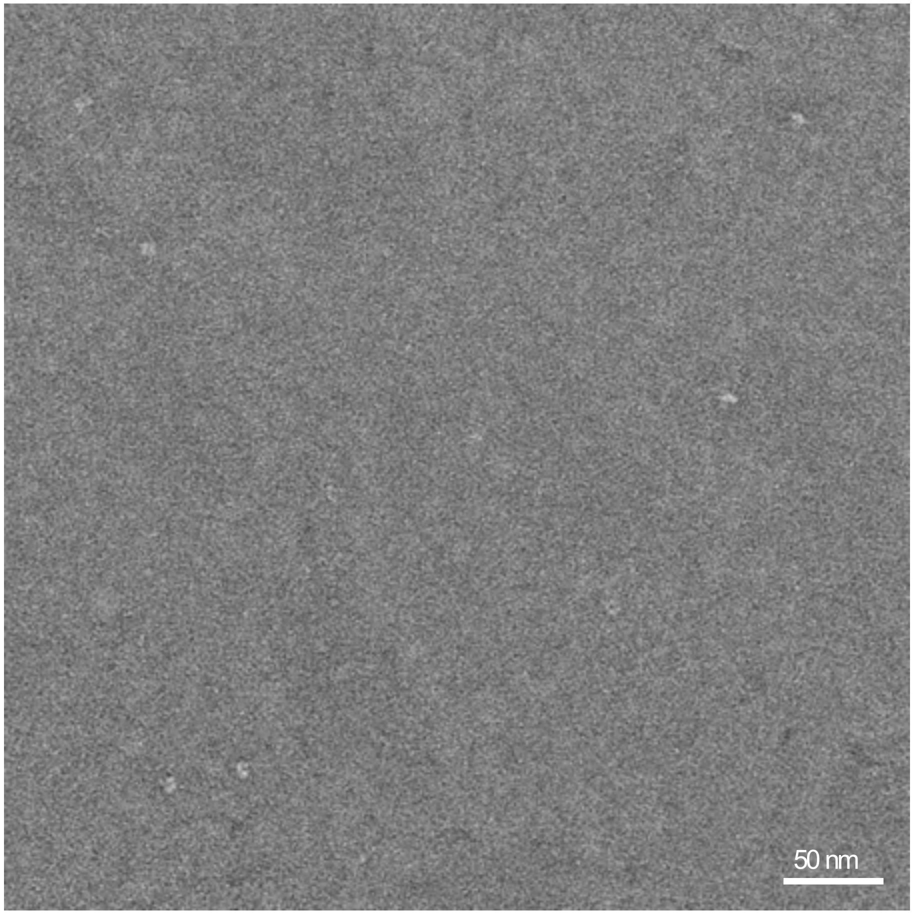

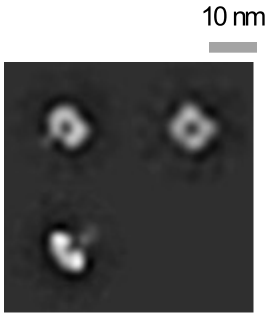

Total particles picked: 4065
Total particles clearly in closed state: 1213
Total particles clearly in open state: 0

N1-CA09-sNAp-155-T466A

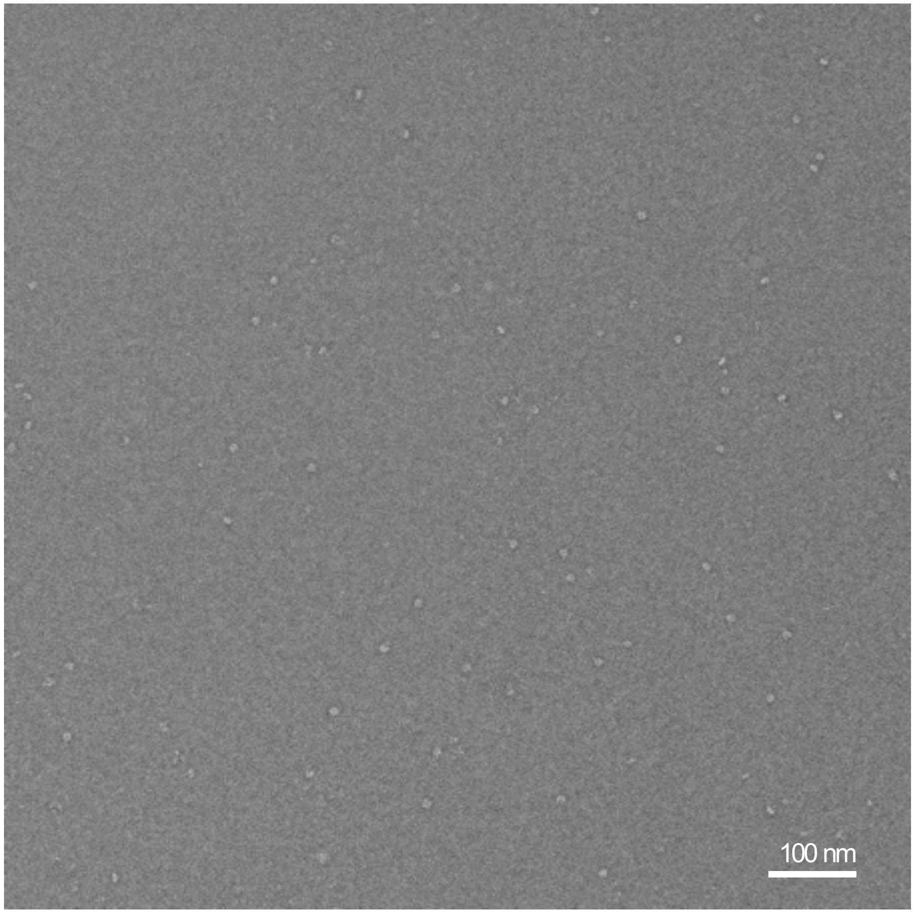

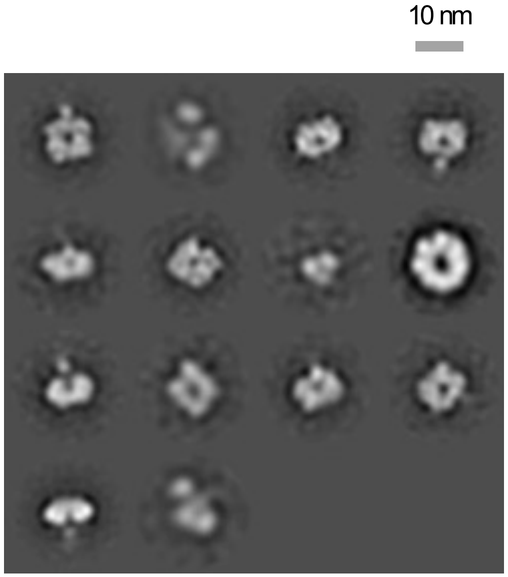

<u>Total particles picked: 4544</u>
Total particles clearly in closed state: 2339
Total particles clearly in open state: 390
