# Supplementary material for: Structure-based design of stabilized recombinant influenza neuraminidase tetramers
Source: Nat Commun. 2022 Apr 5;13:1825. doi: 10.1038/s41467-022-29416-z (PMC8983682; doi:10.1038/s41467-022-29416-z)
Supplement: Supplementary file 5 — Supplementary Data 3 [file 41467_2022_29416_MOESM5_ESM.zip › code_directions (Contains Supplementary Data 3)/README.docx]

Directions for design scripts used in Ellis and Lederhofer et al: "Structure-based design of stabilized recombinant influenza neuraminidase tetramers"

All scripts can be executed using Rosetta modeling software, which can be licensed and downloaded from: https://www.rosettacommons.org/software/license-and-download

Scripts have been tested and validated with the Rosetta versions below:

v2017.18-dev59451

v2019.21-dev60746

v2019.45-dev61026

v2021.14-dev61623

No non-standard hardware is required

The following directions and examples assume that the Rosetta Scripts version v2021.14-dev61623 is installed in the following location:

/software/rosetta/versions/v2021.14-dev61623/

A general input is provided below using the zip file that accompanies these protocols:

/software/rosetta/versions/v2021.14-dev61623/bin/rosetta_scripts -parser:protocol Supplementary_Item_3.xml -s 4b7q-aligned.pdb -native 4b7q-aligned.pdb -beta -nstruct NSTRUCT -parser:script_vars symfile="C4_Z.sym" outpath="output/" resfile="RESFILE.resfile" -overwrite 1 -ex1 -ex2 -unmute all -out:suffix "_RESFILE" -out:level 300 -out::path::all output/ > output/RESFILE.log

In which RESFILE should be replaced with the name of the resfile used to guide design, and NSTRUCT should be replaced with an integer that determines the number of independent trajectories performed. A local "output/" directory should be created to accept results.

EXAMPLES:

Example 1 runs the design protocol using example1.resfile once. This resfile is meant to make the mutations I99P and S196T and not allow Rosetta to choose residue identities.

/software/rosetta/versions/v2021.14-dev61623/bin/rosetta_scripts -parser:protocol Supplementary_Item_3.xml -s 4b7q-aligned.pdb -native 4b7q-aligned.pdb -beta -nstruct 1 -parser:script_vars symfile="C4_Z.sym" outpath="output/" resfile="example1.resfile" -overwrite 1 -ex1 -ex2 -unmute all -out:suffix "_example1" -out:level 300 -out::path::all output/ > output/example1.log

Example 2 runs the design protocol using example2.resfile three times. This resfile allows Rosetta to choose either I or P at position 99, I or M at position 177, and S or T at position 196.

/software/rosetta/versions/v2021.14-dev61623/bin/rosetta_scripts -parser:protocol Supplementary_Item_3.xml -s 4b7q-aligned.pdb -native 4b7q-aligned.pdb -beta -nstruct 3 -parser:script_vars symfile="C4_Z.sym" outpath="output/" resfile="example2.resfile" -overwrite 1 -ex1 -ex2 -unmute all -out:suffix "_example2" -out:level 300 -out::path::all output/ > output/example2.log

Runtime is minimal and is expected to be anywhere from less than 1 min to up to 30 min depending on computing power used.

Expected outputs from examples can be found in the "output/" folder in the related zip file.
