# Supplementary material for: Structure-based design of stabilized recombinant influenza neuraminidase tetramers
Source: Nat Commun. 2022 Apr 5;13:1825. doi: 10.1038/s41467-022-29416-z (PMC8983682; doi:10.1038/s41467-022-29416-z)
Supplement: Supplementary file 6 — Reporting Summary [file 41467_2022_29416_MOESM6_ESM.pdf]

nature portfolio

Corresponding author(s): Neil King & Masaru Kanekiyo

Last updated by author(s): Mar 14, 2022

Reporting Summary

Nature Portfolio wishes to improve the reproducibility of the work that we publish. This form provides structure for consistency and transparency in reporting. For further information on Nature Portfolio policies, see our Editorial Policies and the Editorial Policy Checklist.

Statistics

For all statistical analyses, confirm that the following items are present in the figure legend, table legend, main text, or Methods section.

n/a	Confirmed	
☐	☒	The exact sample size (*n*) for each experimental group/condition, given as a discrete number and unit of measurement
☐	☒	A statement on whether measurements were taken from distinct samples or whether the same sample was measured repeatedly
☒	☐	The statistical test(s) used AND whether they are one- or two-sided<br>*Only common tests should be described solely by name; describe more complex techniques in the Methods section.*
☒	☐	A description of all covariates tested
☒	☐	A description of any assumptions or corrections, such as tests of normality and adjustment for multiple comparisons
☐	☒	A full description of the statistical parameters including central tendency (e.g. means) or other basic estimates (e.g. regression coefficient) AND variation (e.g. standard deviation) or associated estimates of uncertainty (e.g. confidence intervals)
☒	☐	For null hypothesis testing, the test statistic (e.g. *F*, *t*, *r*) with confidence intervals, effect sizes, degrees of freedom and *P* value noted<br>*Give P values as exact values whenever suitable.*
☒	☐	For Bayesian analysis, information on the choice of priors and Markov chain Monte Carlo settings
☒	☐	For hierarchical and complex designs, identification of the appropriate level for tests and full reporting of outcomes
☒	☐	Estimates of effect sizes (e.g. Cohen's *d*, Pearson's *r*), indicating how they were calculated

Our web collection on statistics for biologists contains articles on many of the points above.

Software and code

Policy information about availability of computer code

Data collection	EM data collection: Gatan Digital Micrograph software; Leginon (v3.5)
Data analysis	All data with the following exceptions were analyzed with Matplotlib (v3.3.3) and GraphPad Prism (v8.4.3).<br>EM analysis: Coot (v0.9) Relion (v1.4 and v3.1); CTFFIND4 (v4.1.14); DoG Picker (v0.2); CryoSPARC (v3.1.0); Warp (v1.0.6); Phenix (v1.17.1-3660)<br>Structure rendering: ChimeraX (v1.0.0); Chimera (v1.6.2)<br>Sequence logos: WebLogo (v3)<br>Biolayer interferometry: Octet Analysis (v11)<br>FACS analyses: FlowJo (v10)<br>Figure compilation: Inkscape (v1.0.0)<br>Some images were used from BioRender.

For manuscripts utilizing custom algorithms or software that are central to the research but not yet described in published literature, software must be made available to editors and reviewers. We strongly encourage code deposition in a community repository (e.g. GitHub). See the Nature Portfolio guidelines for submitting code & software for further information.

Data

Policy information about availability of data

All manuscripts must include a data availability statement. This statement should provide the following information, where applicable:

- Accession codes, unique identifiers, or web links for publicly available datasets
- A description of any restrictions on data availability
- For clinical datasets or third party data, please ensure that the statement adheres to our policy

All images and data were generated and analyzed by the authors, and will be made available by the corresponding authors (N.P.K., and M.K.) upon reasonable request. Raw negative-stain EM images and complete set of 2D class averages are provided in Supplementary Item 2. Structural models and density maps are deposited in the Protein Data Bank and Electron Microscopy Data Bank under accession numbers PDB 7U2Q and EMD-26318 (N1-CA09-sNAp-155) and PDB 7U2T and EMD-26319 (N1-MI15-sNAp-174).

Field-specific reporting

Please select the one below that is the best fit for your research. If you are not sure, read the appropriate sections before making your selection.

[x] Life sciences [ ] Behavioural & social sciences [ ] Ecological, evolutionary & environmental sciences

For a reference copy of the document with all sections, see nature.com/documents/nr-reporting-summary-flat.pdf

Life sciences study design

All studies must disclose on these points even when the disclosure is negative.

Sample size	Sample size calculation has not been performed. For the experiments, sample sizes were determined based on our previous studies utilizing similar experimental techniques.
Data exclusions	No data have been excluded.
Replication	All analyses for negative-stain EM, antibody binding, biochemical and biophysical characterization have been performed at least twice. All attempts at replication were successful. Cryo-EM experiments have been performed once, this is standard in the structural field.
Randomization	No randomization have been performed. This is not relevant to the study.
Blinding	Experimenters were blinded to experimental conditions whenever possible. Collection of NS-EM were blinded. Readout of the structural, biochemical and biophysical characterizations were not performed in a blinded manner, as these experiments often require subtle real-time adjustment to ensure optimal data collection.

Reporting for specific materials, systems and methods

We require information from authors about some types of materials, experimental systems and methods used in many studies. Here, indicate whether each material, system or method listed is relevant to your study. If you are not sure if a list item applies to your research, read the appropriate section before selecting a response.

Materials & experimental systems

n/a	Involved in the study	
[ ]	[x]	Antibodies
[ ]	[x]	Eukaryotic cell lines
[x]	[ ]	Palaeontology and archaeology
[x]	[ ]	Animals and other organisms
[x]	[ ]	Human research participants
[x]	[ ]	Clinical data
[x]	[ ]	Dual use research of concern

Methods

n/a	Involved in the study	
[x]	[ ]	ChIP-seq
[ ]	[x]	Flow cytometry
[x]	[ ]	MRI-based neuroimaging

Antibodies

Antibodies used	Antibody 1G01 used in the study was constructed by cloning antibody heavy and light chains into respective mammalian expression vectors. Recombinant 1G01 was produced in mammalian cells (Expi293 cells) by transient transfection of expression vectors and purified by protein A affinity chromatography. Sequences, specificity and function of the antibody was verified prior to use in the study. Antibody 1G01 (human); and CD6 (mouse, Kerafast, Catalog EFD001) were used in biolayer interferometry experiments with a starting concentration of 400 nM and 100 nM respectively. Anti-myc tag antibody (9E10) labelled with Alexa Fluor 488 (Abcam, Catalog ab202008) was used at 1:400 dilution in the FACS experiments.

Validation	All the antibodies used in the study were tested for their reactivity and specificity by ELISA and BLI using a set of recombinant NAs, or virus neutralization assays prior to use in the study.<br>CD6 mAb was validated by vendor: https://www.kerafast.com/item/1757/anti-influenza-a-h1n1-neuraminidase-na-cd6-antibody<br>Anti-myc tag antibody labelled with Alexa Fluor 488 (9E10) was validated by vendor: https://www.abcam.com/alexa-fluor-488-myc-tag-antibody-9e10-ab202008.html

Eukaryotic cell lines

Policy information about cell lines

Cell line source(s)	Expi293F cells (ThermoFisher, Catalog A14527); MDCK-SIAT1-PB1 cells (Creanga et al., Nat Commun. 2021). The parental MDCK-SIAT1 cells were purchased from Millipore Sigma (Catalog 05071502), HEK-293-PB1 (a gift from Dr. Jesse Bloom, Fred Hutchinson Cancer Research Center)
Authentication	Commercial cell lines were authenticated by manufacturers and no further authentications were performed by the authors; MDCK-SIAT-PB1 cells were not authenticated. All cells used in the studies were not extensively passaged.
Mycoplasma contamination	Tested negative (monthly).
Commonly misidentified lines (See ICLAC register)	No commonly misidentified cell lines were used in this study.

Flow Cytometry

Plots

Confirm that:

- [x] The axis labels state the marker and fluorochrome used (e.g. CD4-FITC).
- [x] The axis scales are clearly visible. Include numbers along axes only for bottom left plot of group (a 'group' is an analysis of identical markers).
- [x] All plots are contour plots with outliers or pseudocolor plots.
- [x] A numerical value for number of cells or percentage (with statistics) is provided.

Methodology

Sample preparation	Expi293F cells were transfected with plasmids encoding NA constructs by using PEI-MAX 24 h prior to the experiements. Cells were washed and resuspended in 25 mM HEPES, 150 mM NaCl, 2.5% glycerol, 1% BSA, plus or minus 5 mM EDTA.
Instrument	Attune NxT flow cytometer for data acuisition
Software	FlowJo v10
Cell population abundance	Percent positivity for subpopulation is indicated in each plot.
Gating strategy	FSC-A and SSC-A gating to exclude cell debris; SSC-H and SSC-A gating to select singlet cells; ZombieViolet gating to exclude dead cells; and anti-myc (9E10)-AF488 gating to select myc-positive cells. Within this population, anti-NA (1G01 or CD6)-AF680 binding was assessed.

- [x] Tick this box to confirm that a figure exemplifying the gating strategy is provided in the Supplementary Information.
